# A Higher-Order Generalized Singular Value Decomposition for Comparison of Global mRNA Expression from Multiple Organisms

**DOI:** 10.1371/journal.pone.0028072

**Published:** 2011-12-22

**Authors:** Sri Priya Ponnapalli, Michael A. Saunders, Charles F. Van Loan, Orly Alter

**Affiliations:** 1 Department of Electrical and Computer Engineering, University of Texas at Austin, Texas, United States of America; 2 Department of Management Science and Engineering, Stanford University, Stanford, California, United States of America; 3 Department of Computer Science, Cornell University, Ithaca, New York, United States of America; 4 Scientific Computing and Imaging (SCI) Institute and Departments of Bioengineering and Human Genetics, University of Utah, Salt Lake City, Utah, United States of America; Wayne State University, United States of America

## Abstract

The number of high-dimensional datasets recording multiple aspects of a single phenomenon is increasing in many areas of science, accompanied by a need for mathematical frameworks that can compare multiple large-scale matrices with different row dimensions. The only such framework to date, the generalized singular value decomposition (GSVD), is limited to two matrices. We mathematically define a higher-order GSVD (HO GSVD) for *N*≥2 matrices 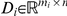, each with full column rank. Each matrix is exactly factored as *D_i_* = *U_i_*Σ*_i_V^T^*, where *V*, identical in all factorizations, is obtained from the eigensystem *SV* = *V*Λ of the arithmetic mean *S* of all pairwise quotients 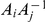 of the matrices 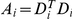, *i*≠*j*. We prove that this decomposition extends to higher orders almost all of the mathematical properties of the GSVD. The matrix *S* is nondefective with *V* and Λ real. Its eigenvalues satisfy *λ_k_*≥1. Equality holds if and only if the corresponding eigenvector *v_k_* is a right basis vector of equal significance in all matrices *D_i_* and *D_j_*, that is *σ_i,k_*/*σ_j,k_* = 1 for all *i* and *j*, and the corresponding left basis vector *u_i,k_* is orthogonal to all other vectors in *U_i_* for all *i*. The eigenvalues *λ_k_* = 1, therefore, define the “common HO GSVD subspace.” We illustrate the HO GSVD with a comparison of genome-scale cell-cycle mRNA expression from *S. pombe*, *S. cerevisiae* and human. Unlike existing algorithms, a mapping among the genes of these disparate organisms is not required. We find that the approximately common HO GSVD subspace represents the cell-cycle mRNA expression oscillations, which are similar among the datasets. Simultaneous reconstruction in the common subspace, therefore, removes the experimental artifacts, which are dissimilar, from the datasets. In the simultaneous sequence-independent classification of the genes of the three organisms in this common subspace, genes of highly conserved sequences but significantly different cell-cycle peak times are correctly classified.

## Introduction

In many areas of science, especially in biotechnology, the number of high-dimensional datasets recording multiple aspects of a single phenomenon is increasing. This is accompanied by a fundamental need for mathematical frameworks that can compare multiple large-scale matrices with different row dimensions. For example, comparative analyses of global mRNA expression from multiple model organisms promise to enhance fundamental understanding of the universality and specialization of molecular biological mechanisms, and may prove useful in medical diagnosis, treatment and drug design [1]. Existing algorithms limit analyses to subsets of homologous genes among the different organisms, effectively introducing into the analysis the assumption that sequence and functional similarities are equivalent (e.g., [2]). However, it is well known that this assumption does not always hold, for example, in cases of nonorthologous gene displacement, when nonorthologous proteins in different organisms fulfill the same function [3]. For sequence-independent comparisons, mathematical frameworks are required that can distinguish and separate the similar from the dissimilar among multiple large-scale datasets tabulated as matrices with different row dimensions, corresponding to the different sets of genes of the different organisms. The only such framework to date, the generalized singular value decomposition (GSVD) [4]–[7], is limited to two matrices.

It was shown that the GSVD provides a mathematical framework for sequence-independent comparative modeling of DNA microarray data from two organisms, where the mathematical variables and operations represent biological reality [7], [8]. The variables, significant subspaces that are common to both or exclusive to either one of the datasets, correlate with cellular programs that are conserved in both or unique to either one of the organisms, respectively. The operation of reconstruction in the subspaces common to both datasets outlines the biological similarity in the regulation of the cellular programs that are conserved across the species. Reconstruction in the common and exclusive subspaces of either dataset outlines the differential regulation of the conserved relative to the unique programs in the corresponding organism. Recent experimental results [9] verify a computationally predicted genome-wide mode of regulation that correlates DNA replication origin activity with mRNA expression [10], [11], demonstrating that GSVD modeling of DNA microarray data can be used to correctly predict previously unknown cellular mechanisms.

We now define a higher-order GSVD (HO GSVD) for the comparison of 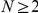 datasets. The datasets are tabulated as 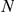 real matrices 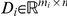, each with full column rank, with different row dimensions and the same column dimension, where there exists a one-to-one mapping among the columns of the matrices. Like the GSVD, the HO GSVD is an exact decomposition, i.e., each matrix is exactly factored as 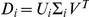, where the columns of 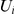 and 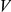 have unit length and are the left and right basis vectors respectively, and each 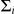 is diagonal and positive definite. Like the GSVD, the matrix 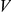 is identical in all factorizations. In our HO GSVD, the matrix 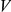 is obtained from the eigensystem 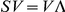 of the arithmetic mean 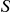 of all pairwise quotients 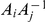 of the matrices 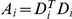, or equivalently of all 
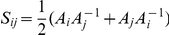
, 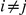.

To clarify our choice of 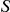, we note that in the GSVD, defined by Van Loan [5], the matrix 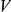 can be formed from the eigenvectors of the unbalanced quotient 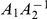 (Section 1 in Appendix S1). We observe that this 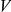 can also be formed from the eigenvectors of the balanced arithmetic mean 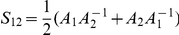. We prove that in the case of 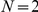, our definition of 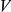 by using the eigensystem of 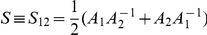 leads algebraically to the GSVD (Theorems S1–S5 in Appendix S1), and therefore, as Paige and Saunders showed [6], can be computed in a stable way. We also note that in the GSVD, the matrix 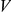 does not depend upon the ordering of the matrices 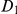 and 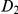. Therefore, we define our HO GSVD for 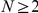 matrices by using the balanced arithmetic mean 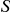 of all pairwise arithmetic means 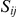, each of which defines the GSVD of the corresponding pair of matrices 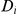 and 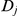, noting that 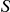 does not depend upon the ordering of the matrices 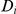 and 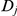.

We prove that 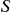 is nondefective (it has 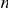 independent eigenvectors), and that its eigensystem is real (Theorem 1). We prove that the eigenvalues of 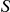 satisfy 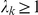 (Theorem 2). As in our GSVD comparison of two matrices [7], we interpret the 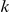
th diagonal of 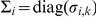 in the factorization of the 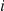 th matrix 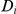 as indicating the significance of the 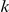
th right basis vector 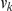 in 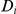 in terms of the overall information that 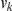 captures in 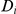. The ratio 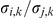 indicates the significance of 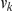 in 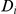 relative to its significance in 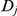. We prove that an eigenvalue of 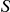 satisfies 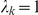 if and only if the corresponding eigenvector 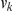 is a right basis vector of equal significance in all 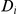 and 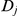, that is, 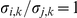 for all 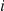 and 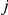, and the corresponding left basis vector 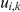 is orthonormal to all other vectors in 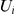 for all 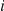. We therefore mathematically define, in analogy with the GSVD, the “common HO GSVD subspace” of the 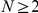 matrices to be the subspace spanned by the right basis vectors 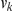 that correspond to the 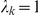 eigenvalues of 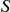 (Theorem 3). We also show that each of the right basis vectors 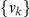 that span the common HO GSVD subspace is a generalized singular vector of all pairwise GSVD factorizations of the matrices 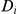 and 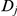 with equal corresponding generalized singular values for all 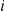 and 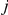 (Corollary 1).

Recent research showed that several higher-order generalizations are possible for a given matrix decomposition, each preserving some but not all of the properties of the matrix decomposition [12]–[14] (see also Theorem S6 and Conjecture S1 in Appendix S1). Our new HO GSVD extends to higher orders all of the mathematical properties of the GSVD except for complete column-wise orthogonality of the left basis vectors that form the matrix 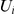 for all 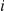, i.e., in each factorization.

We illustrate the HO GSVD with a comparison of cell-cycle mRNA expression from *S. pombe* [15], [16], *S. cerevisiae* [17] and human [18]. Unlike existing algorithms, a mapping among the genes of these disparate organisms is not required (Section 2 in Appendix S1). We find that the common HO GSVD subspace represents the cell-cycle mRNA expression oscillations, which are similar among the datasets. Simultaneous reconstruction in this common subspace, therefore, removes the experimental artifacts, which are dissimilar, from the datasets. Simultaneous sequence-independent classification of the genes of the three organisms in the common subspace is in agreement with previous classifications into cell-cycle phases [19]. Notably, genes of highly conserved sequences across the three organisms [20], [21] but significantly different cell-cycle peak times, such as genes from the ABC transporter superfamily [22]–[28], phospholipase B-encoding genes [29], [30] and even the B cyclin-encoding genes [31], [32], are correctly classified.

## Methods

### HO GSVD Construction

**Figure 1:**
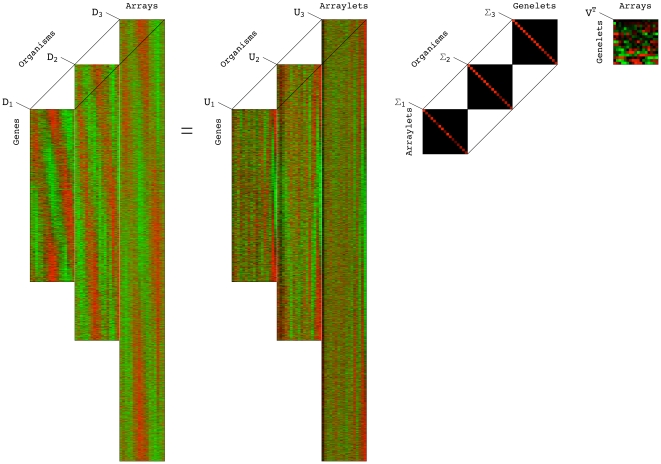
Higher-order generalized singular value decomposition (HO GSVD). In this raster display of Equation (1) with overexpression (red), no change in expression (black), and underexpression (green) centered at gene- and array-invariant expression, the *S. pombe*, S. cerevisiae and human global mRNA expression datasets are tabulated as organism-specific genes
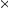
17-arrays matrices 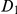, 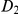 and 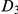. The underlying assumption is that there exists a one-to-one mapping among the 17 columns of the three matrices but not necessarily among their rows. These matrices are transformed to the reduced diagonalized matrices 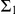, 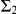 and 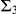, each of 17-“arraylets,” i.e., left basis vectors
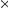
17-“genelets,” i.e., right basis vectors, by using the organism-specific genes
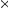
17-arraylets transformation matrices 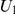, 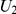 and 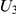 and the shared 17-genelets
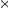
17-arrays transformation matrix 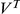. We prove that with our particular 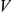 of Equations (2)–(4), this decomposition extends to higher orders all of the mathematical properties of the GSVD except for complete column-wise orthogonality of the arraylets, i.e., left basis vectors that form the matrices 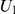, 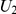 and 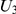. We therefore mathematically define, in analogy with the GSVD, the “common HO GSVD subspace” of the 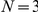 matrices to be the subspace spanned by the genelets, i.e., right basis vectors 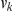 that correspond to higher-order generalized singular values that are equal, 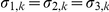, where, as we prove, the corresponding arraylets, i.e., the left basis vectors 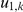, 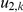 and 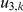, are orthonormal to all other arraylets in 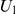, 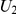 and 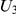. We show that like the GSVD for two organisms [7], the HO GSVD provides a sequence-independent comparative mathematical framework for datasets from more than two organisms, where the mathematical variables and operations represent biological reality: Genelets of common significance in the multiple datasets, and the corresponding arraylets, represent cell-cycle checkpoints or transitions from one phase to the next, common to *S. pombe*, *S. cerevisiae* and human. Simultaneous reconstruction and classification of the three datasets in the common subspace that these patterns span outline the biological similarity in the regulation of their cell-cycle programs. Notably, genes of significantly different cell-cycle peak times [19] but highly conserved sequences [20], [21] are correctly classified.

Suppose we have a set of 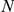 real matrices 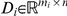 each with full column rank. We define a HO GSVD of these 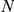 matrices as
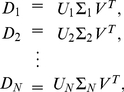
(1)where each 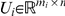 is composed of normalized left basis vectors, each 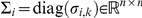 is diagonal with 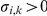, and 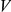, identical in all matrix factorizations, is composed of normalized right basis vectors. As in the GSVD comparison of global mRNA expression from two organisms [7], in the HO GSVD comparison of global mRNA expression from 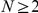 organisms, the shared right basis vectors 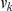 of Equation (1) are the “genelets” and the 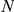 sets of left basis vectors 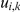 are the 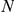 sets of “arraylets” (Figure 1 and Section 2 in Appendix S1). We obtain 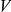 from the eigensystem of 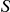, the arithmetic mean of all pairwise quotients 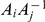 of the matrices 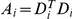, or equivalently of all 
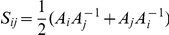
, 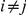:
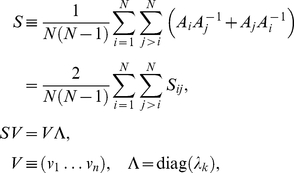
(2)with 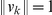. We prove that 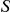 is nondefective, i.e., 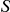 has 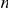 independent eigenvectors, and that its eigenvectors 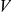 and eigenvalues 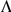 are real (Theorem 1). We prove that the eigenvalues of 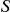 satisfy 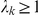 (Theorem 2).

Given 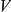, we compute matrices 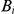 by solving 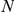 linear systems:
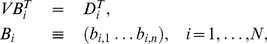
(3)and we construct 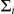 and 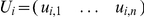 by normalizing the columns of 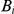:
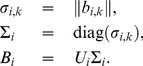
(4)


### HO GSVD Interpretation

In this construction, the rows of each of the 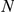 matrices 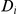 are superpositions of the same right basis vectors, the columns of 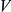 (Figures S1 and S2 and Section 1 in Appendix S1). As in our GSVD comparison of two matrices, we interpret the 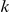
th diagonals of 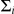, the “higher-order generalized singular value set” 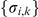, as indicating the significance of the 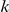
th right basis vector 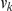 in the matrices 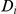, and reflecting the overall information that 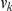 captures in each 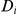 respectively. The ratio 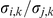 indicates the significance of 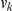 in 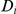 relative to its significance in 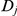. A ratio of 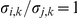 for all 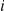 and 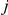 corresponds to a right basis vector 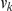 of equal significance in all 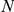 matrices 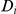. GSVD comparisons of two matrices showed that right basis vectors of approximately equal significance in the two matrices reflect themes that are common to both matrices under comparison [7]. A ratio of 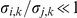 indicates a basis vector 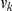 of almost negligible significance in 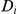 relative to its significance in 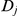. GSVD comparisons of two matrices showed that right basis vectors of negligible significance in one matrix reflect themes that are exclusive to the other matrix.

We prove that an eigenvalue of 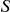 satisfies 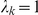 if and only if the corresponding eigenvector 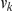 is a right basis vector of equal significance in all 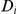 and 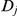, that is, 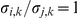 for all 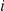 and 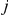, and the corresponding left basis vector 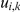 is orthonormal to all other vectors in 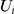 for all 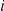. We therefore mathematically define, in analogy with the GSVD, the “common HO GSVD subspace” of the 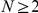 matrices to be the subspace spanned by the right basis vectors 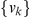 corresponding to the eigenvalues of 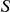 that satisfy 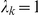 (Theorem 3).

It follows that each of the right basis vectors 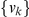 that span the common HO GSVD subspace is a generalized singular vector of all pairwise GSVD factorizations of the matrices 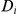 and 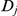 with equal corresponding generalized singular values for all 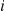 and 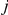 (Corollary 1). Since the GSVD can be computed in a stable way [6], we note that the common HO GSVD subspace can also be computed in a stable way by computing all pairwise GSVD factorizations of the matrices 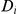 and 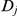. This also suggests that it may be possible to formulate the HO GSVD as a solution to an optimization problem, in analogy with existing variational formulations of the GSVD [33]. Such a formulation may lead to a stable numerical algorithm for computing the HO GSVD, and possibly also to a higher-order general Gauss-Markov linear statistical model [34]–[36].

We show, in a comparison of 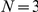 matrices, that the approximately common HO GSVD subspace of these three matrices reflects a theme that is common to the three matrices under comparison (Section 2).

### HO GSVD Mathematical Properties

#### Theorem 1


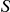
 *is nondefective (it has* 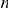 *independent eigenvectors) and its eigensystem is real.*

*Proof.* From Equation (2) it follows that
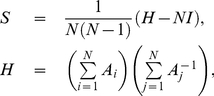
(5)and the eigenvectors of 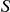 equal the eigenvectors of 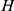.

Let the SVD of the matrices 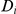 appended along the 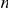-columns axis be
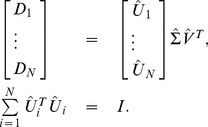
(6)Since the matrices 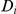 are real and with full column rank, it follows from the SVD of 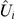 that the symmetric matrices 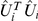 are real and positive definite, and their inverses exist. It then follows from Equations (5) and (6) that 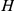 is similar to 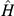,
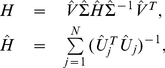
(7)and the eigenvalues of 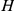 equal the eigenvalues of 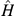.

A sum of real, symmetric and positive definite matrices, 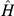 is also real, symmetric and positive definite; therefore, its eigensystem
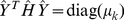
(8)is real with 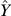 orthogonal and 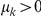. Without loss of generality let 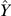 be orthonormal, such that 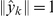. It follows from the similarity of 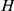 with 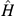 that the eigensystem of 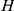 can be written as 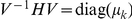, with the real and nonsingular 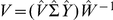, where 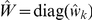 and 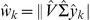 such that 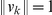 for all 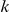.

Thus, from Equation (5), 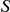 is nondefective with real eigenvectors 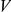. Also, the eigenvalues of 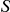 satisfy
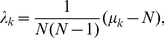
(9)where 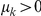 are the eigenvalues of 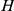 and 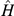. Thus, the eigenvalues of 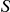 are real. □

#### Theorem 2

*The eigenvalues of* 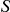 *satisfy* 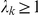 *.*

*Proof.* Following Equation (9), asserting that the eigenvalues of 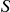 satisfy 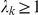 is equivalent to asserting that the eigenvalues of 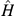 satisfy 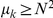.

From Equations (6) and (7), the eigenvalues of 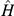 satisfy
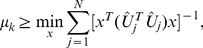
(10)under the constraint that
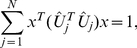
(11)where 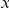 is a real unit vector, and where it follows from the Cauchy-Schwarz inequality [37] (see also [4], [34], [38]) for the real nonzero vectors 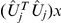 and 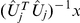 that for all 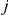


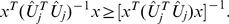
(12)With the constraint of Equation (11), which requires the sum of the 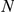 positive numbers 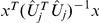 to equal one, the lower bound on the eigenvalues of 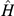 in Equation (10) is at its minimum when the sum of the inverses of these numbers is at its minimum, that is, when the numbers equal
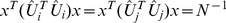
(13)for all 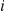 and 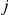. Thus, the eigenvalues of 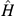 satisfy 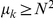. □

#### Theorem 3

***The common HO GSVD subspace.*** *An eigenvalue of* 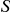 *satisfies* 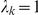 *if and only if the corresponding eigenvector* 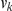 *is a right basis vector of equal significance in all* 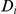 *and* 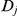 *, that is,* 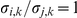 *for all* 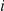 *and* 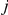 *, and the corresponding left basis vector* 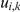 *is orthonormal to all other vectors in* 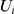 *for all* 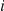 *. The “common HO GSVD subspace” of the* 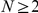 *matrices is, therefore, the subspace spanned by the right basis vectors* 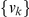 *corresponding to the eigenvalues of* 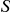 *that satisfy* 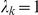 *.*

*Proof.* Without loss of generality, let 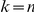. From Equation (12) and the Cauchy-Schwarz inequality, an eigenvalue of 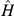 equals its minimum lower bound 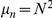 if and only if the corresponding eigenvector 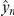 is also an eigenvector of 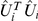 for all 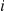 [37], where, from Equation (13), the corresponding eigenvalue equals 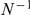,
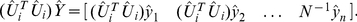
(14)


Given the eigenvectors 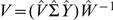 of 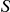, we solve Equation (3) for each 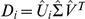 of Equation (6), and obtain
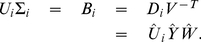
(15)


Following Equations (14) and (15), where 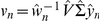 corresponds to a minimum eigenvalue 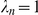, and since 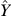 is orthonormal, we obtain
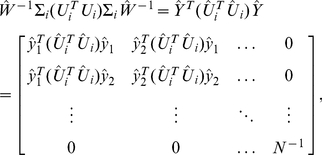
(16)with zeroes in the 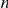
th row and the 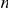
th column of the matrix above everywhere except for the diagonal element. Thus, an eigenvalue of 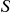 satisfies 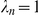 if and only if the corresponding left basis vectors 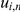 are orthonormal to all other vectors in 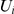.

The corresponding higher-order generalized singular values are 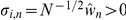. Thus 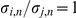 for all 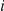 and 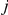, and the corresponding right basis vector 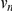 is of equal significance in all matrices 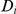 and 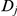. □

#### Corollary 1

*An eigenvalue of* 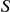 *satisfies* 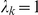 *if and only if the corresponding right basis vector* 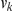 *is a generalized singular vector of all pairwise GSVD factorizations of the matrices* 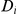 *and* 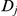 *with equal corresponding generalized singular values for all* 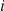 *and* 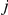 *.*

*Proof.* From Equations (12) and (13), and since the pairwise quotients 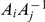 are similar to 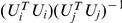 with the similarity transformation of 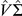 for all 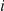 and 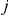, it follows that an eigenvalue of 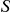 satisfies 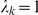 if and only if the corresponding right basis vector 
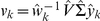
 is also an eigenvector of each of the pairwise quotients 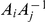 of the matrices 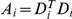 with equal corresponding eigenvalues, or equivalently of all 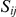 with all eigenvalues at their minimum of one,
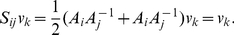
(17)We prove (Theorems S1–S5 in Appendix S1) that in the case of 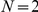 matrices our definition of 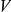 by using the eigensystem of 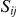 leads algebraically to the GSVD, where an eigenvalue of 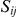 equals its minimum of one if and only if the two corresponding generalized singular values are equal, such that the corresponding generalized singular vector 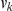 is of equal significance in both matrices 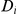 and 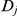. Thus, it follows that each of the right basis vectors 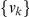 that span the common HO GSVD subspace is a generalized singular vector of all pairwise GSVD factorizations of the matrices 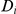 and 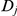 with equal corresponding generalized singular values for all 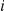 and 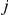. □

Note that since the GSVD can be computed in a stable way [6], the common HO GSVD subspace we define (Theorem 3) can also be computed in a stable way by computing all pairwise GSVD factorizations of the matrices 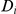 and 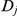 (Corollary 1). It may also be possible to formulate the HO GSVD as a solution to an optimization problem, in analogy with existing variational formulations of the GSVD [33]. Such a formulation may lead to a stable numerical algorithm for computing the HO GSVD, and possibly also to a higher-order general Gauss-Markov linear statistical model [34]–[36].

## Results

### HO GSVD Comparison of Global mRNA Expression from Three Organisms

Consider now the HO GSVD comparative analysis of global mRNA expression datasets from the 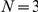 organisms *S. pombe*, *S. cerevisiae* and human (Section 2.1 in Appendix S1, Mathematica Notebooks S1 and S2, and Datasets S1, S2 and S3). The datasets are tabulated as matrices of 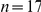 columns each, corresponding to DNA microarray-measured mRNA expression from each organism at 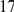 time points equally spaced during approximately two cell-cycle periods. The underlying assumption is that there exists a one-to-one mapping among the 17 columns of the three matrices but not necessarily among their rows, which correspond to either 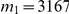-*S. pombe* genes, 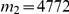-*S. cerevisiae* genes or 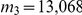-human genes. The HO GSVD of Equation (1) transforms the datasets from the organism-specific genes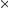 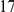-arrays spaces to the reduced spaces of the 17-“arraylets,” i.e., left basis vactors
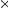
17-“genelets,” i.e., right basis vectors, where the datasets 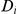 are represented by the diagonal nonnegative matrices 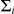, by using the organism-specific genes
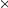
17-arraylets transformation matrices 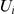 and the one shared 17-genelets
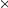
17-arrays transformation matrix 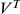 (Figure 1).

**Figure 2:**
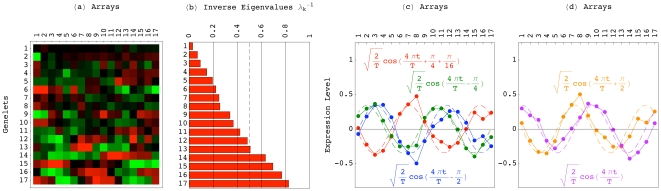
Genelets or right basis vectors. (*a*) Raster display of the expression of the 17 genelets, i.e., HO GSVD patterns of expression variation across time, with overexpression (red), no change in expression (black) and underexpression (green) around the array-, i.e., time-invariant expression. (*b*) Bar chart of the corresponding inverse eigenvalues 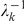, showing that the 13th through the 17th genelets correspond to 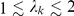. (*c*) Line-joined graphs of the 13th (red), 14th (blue) and 15th (green) genelets in the two-dimensional subspace that approximates the five-dimensional HO GSVD subspace (Figure S4 and Section 2.4), normalized to zero average and unit variance. (*d*) Line-joined graphs of the projected 16th (orange) and 17th (violet) genelets in the two-dimensional subspace. The five genelets describe expression oscillations of two periods in the three time courses.

Following Theorem 3, the approximately common HO GSVD subspace of the three datasets is spanned by the five genelets 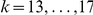 that correspond to 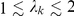. We find that these five genelets are approximately equally significant with 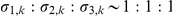 in the *S. pombe*, *S. cerevisiae* and human datasets, respectively (Figure 2 *a* and *b*). The five corresponding arraylets in each dataset are 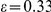-orthonormal to all other arraylets (Figure S3 in Appendix S1).

### Common HO GSVD Subspace Represents Similar Cell-Cycle Oscillations

The expression variations across time of the five genelets that span the approximately common HO GSVD subspace fit normalized cosine functions of two periods, superimposed on time-invariant expression (Figure 2 *c* and *d*). Consistently, the corresponding organism-specific arraylets are enriched [39] in overexpressed or underexpressed organism-specific cell cycle-regulated genes, with 24 of the 30 *P*-values 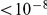 (Table 1 and Section 2.2 in Appendix S1). For example, the three 17th arraylets, which correspond to the 0-phase 17th genelet, are enriched in overexpressed G2 *S. pombe* genes, G2/M and M/G1 *S. cerevisiae* genes and S and G2 human genes, respectively, representing the cell-cycle checkpoints in which the three cultures are initially synchronized.

**Table 1:** Arraylets or left basis vectors.

		Overexpression		Underexpression	
Dataset	Arraylet	Annotation	*P*-value	Annotation	*P*-value
*S. pombe*	13	G2	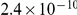	G1	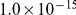
	14	M	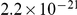	G2	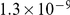
	15	M	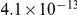	S	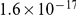
	16	G2	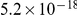	G1	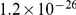
	17	G2	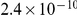	S	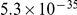
*S. cerevisiae*	13	S/G2	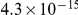	M/G1	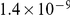
	14	M/G1	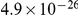	G2/M	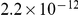
	15	G1	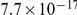	S	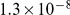
	16	G2/M	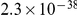	G1	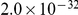
	17	G2/M	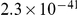	G1	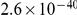
Human	13	G1/S	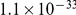	G2	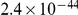
	14	M/G1	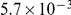	G2	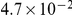
	15	G2	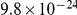	None	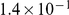
	16	G1/S	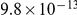	G2	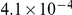
	17	G2	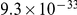	M/G1	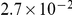

Probabilistic significance of the enrichment of the arraylets, i.e., HO GSVD patterns of expression variation across the *S. pombe*, *S. cerevisiae* and human genes, that span the common HO GSVD subspace in each dataset, in over- or underexpressed cell cycle-regulated genes. The *P*-value of each enrichment is calculated as described [39] (Section 2.2 in Appendix S1) assuming hypergeometric distribution of the annotations (Datasets S1, S2, S3) among the genes, including the 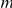 = 100 genes most over- or underexpressed in each arraylet.

**Figure 3:**
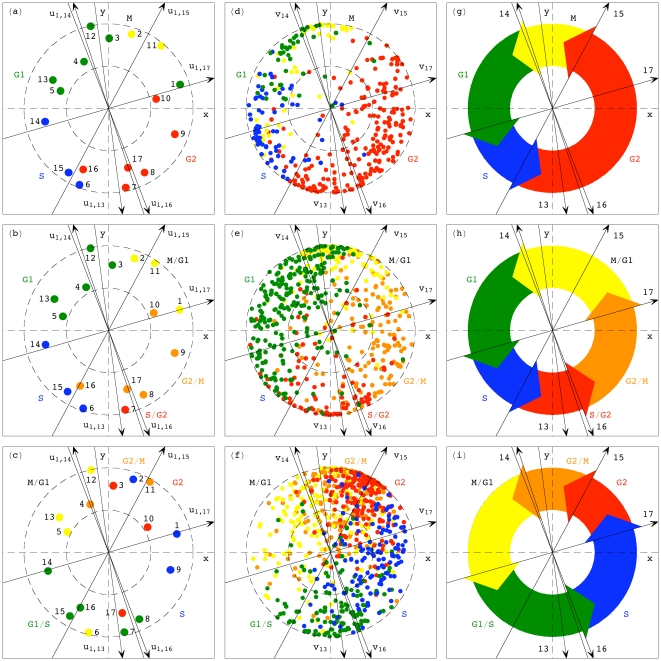
Common HO GSVD subspace represents similar cell-cycle oscillations. (*a*–*c*) *S. pombe*, *S. cerevisiae* and human array expression, projected from the five-dimensional common HO GSVD subspace onto the two-dimensional subspace that approximates it (Sections 2.3 and 2.4 in Appendix S1). The arrays are color-coded according to their previous cell-cycle classification [15]–[18]. The arrows describe the projections of the 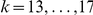 arraylets of each dataset. The dashed unit and half-unit circles outline 100% and 50% of added-up (rather than canceled-out) contributions of these five arraylets to the overall projected expression. (*d*–*f*) Expression of 380, 641 and 787 cell cycle-regulated genes of *S. pombe*, *S. cerevisiae* and human, respectively, color-coded according to previous classifications. (*g*–*i*) The HO GSVD pictures of the *S. pombe*, *S. cerevisiae* and human cell-cycle programs. The arrows describe the projections of the 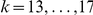 shared genelets and organism-specific arraylets that span the common HO GSVD subspace and represent cell-cycle checkpoints or transitions from one phase to the next.

Simultaneous sequence-independent reconstruction and classification of the three datasets in the common subspace outline cell-cycle progression in time and across the genes in the three organisms (Sections 2.3 and 2.4 in Appendix S1). Projecting the expression of the 17 arrays of either organism from the corresponding five-dimensional arraylets subspace onto the two-dimensional subspace that approximates it (Figure S4 in Appendix S1), 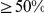 of the contributions of the arraylets add up, rather than cancel out (Figure 3 *a*–*c*). In these two-dimensional subspaces, the angular order of the arrays of either organism describes cell-cycle progression in time through approximately two cell-cycle periods, from the initial cell-cycle phase and back to that initial phase twice. Projecting the expression of the genes, 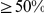 of the contributions of the five genelets add up in the overall expression of 343 of the 380 *S. pombe* genes classified as cell cycle-regulated, 554 of the 641 *S. cerevisiae* cell-cycle genes, and 632 of the 787 human cell-cycle genes (Figure 3 *d*–*f*). Simultaneous classification of the genes of either organism into cell-cycle phases according to their angular order in these two-dimensional subspaces is consistent with the classification of the arrays, and is in good agreement with the previous classifications of the genes (Figure 3 *g*–*i*). With all 3167 *S. pombe*, 4772 *S. cerevisiae* and 13,068 human genes sorted, the expression variations of the five arraylets from each organism approximately fit one-period cosines, with the initial phase of each arraylet (Figures S5, S6, S7 in Appendix S1) similar to that of its corresponding genelet (Figure 2). The global mRNA expression of each organism, reconstructed in the common HO GSVD subspace, approximately fits a traveling wave, oscillating across time and across the genes.

Note also that simultaneous reconstruction in the common HO GSVD subspace removes the experimental artifacts and batch effects, which are dissimilar, from the three datasets. Consider, for example, the second genelet. With 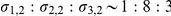 in the *S. pombe*, *S. cerevisiae* and human datasets, respectively, this genelet is almost exclusive to the *S. cerevisiae* dataset. This genelet is anticorrelated with a time decaying pattern of expression (Figure 2*a*). Consistently, the corresponding *S. cerevisiae*-specific arraylet is enriched in underexpressed *S. cerevisiae* genes that were classified as up-regulated by the *S. cerevisiae* synchronizing agent, the 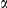-factor pheromone, with the *P*-value 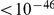. Reconstruction in the common subspace effectively removes this *S. cerevisiae*-approximately exclusive pattern of expression variation from the three datasets.

### Simultaneous HO GSVD Classification of Homologous Genes of Different Cell-Cycle Peak Times

Notably, in the simultaneous sequence-independent classification of the genes of the three organisms in the common subspace, genes of significantly different cell-cycle peak times [19] but highly conserved sequences [20], [21] are correctly classified (Section 2.5 in Appendix S1).

**Figure 4:**
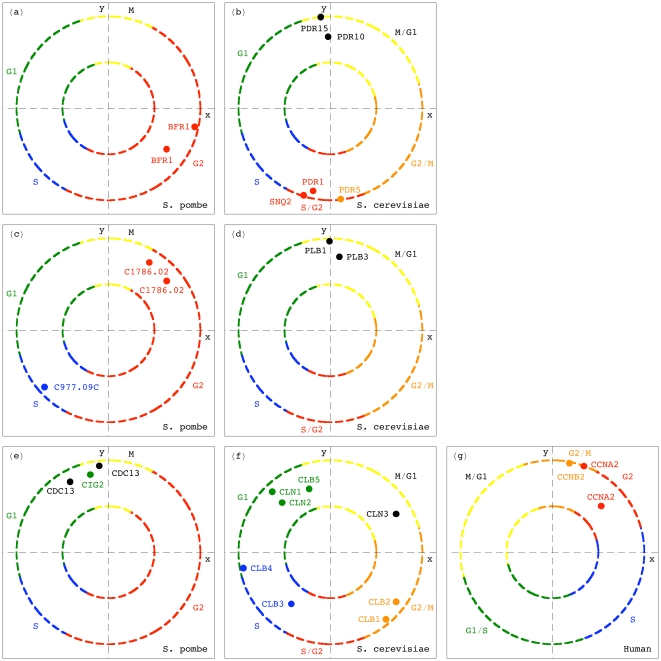
Simultaneous HO GSVD classification of homologous genes of different cell-cycle peak times. (*a*) The *S. pombe* gene *BFR1*, and (*b*) its closest *S. cerevisiae* homologs. (*c*) The *S. pombe* and (*d*) *S. cerevisiae* closest homologs of the *S. cerevisiae* gene *PLB1*. (*e*) The *S. pombe* cyclin-encoding gene *CIG2* and its closest *S. pombe*, (*f*) *S. cerevisiae* and (*g*) human homologs.

For example, consider the G2 *S. pombe* gene *BFR1* (Figure 4*a*), which belongs to the evolutionarily highly conserved ATP-binding cassette (ABC) transporter superfamily [22]. The closest homologs of *BFR1* in our *S. pombe*, *S. cerevisiae* and human datasets are the *S. cerevisiae* genes *SNQ2*, *PDR5*, *PDR15* and *PDR10* (Table S1*a* in Appendix S1). The expression of *SNQ2* and *PDR5* is known to peak at the S/G2 and G2/M cell-cycle phases, respectively [17]. However, sequence similarity does not imply similar cell-cycle peak times, and *PDR15* and *PDR10*, the closest homologs of *PDR5*, are induced during stationary phase [23], which has been hypothesized to occur in G1, before the Cdc28-defined cell-cycle arrest [24]. Consistently, we find *PDR15* and *PDR10* at the M/G1 to G1 transition, antipodal to (i.e., half a cell-cycle period apart from) *SNQ2* and *PDR5*, which are projected onto S/G2 and G2/M, respectively (Figure 4*b*). We also find the transcription factor *PDR1* at S/G2, its known cell-cycle peak time, adjacent to *SNQ2* and *PDR5*, which it positively regulates and might be regulated by, and antipodal to *PDR15*, which it negatively regulates [25]–[28].

Another example is the *S. cerevisiae* phospholipase B-encoding gene *PLB1* [29], which peaks at the cell-cycle phase M/G1 [30]. Its closest homolog in our *S. cerevisiae* dataset, *PLB3*, also peaks at M/G1 [17] (Figure 4*d*). However, among the closest *S. pombe* and human homologs of *PLB1* (Table S1*b* in Appendix S1), we find the *S. pombe* genes *SPAC977.09c* and *SPAC1786.02*, which expressions peak at the almost antipodal *S. pombe* cell-cycle phases S and G2, respectively [19] (Figure 4*c*).

As a third example, consider the *S. pombe* G1 B-type cyclin-encoding gene *CIG2* [31], [32] (Table S1*c* in Appendix S1). Its closest *S. pombe* homolog, *CDC13*, peaks at M [19] (Figure 4*e*). The closest human homologs of *CIG2*, the cyclins *CCNA2* and *CCNB2*, peak at G2 and G2/M, respectively (Figure 4*g*). However, while periodicity in mRNA abundance levels through the cell cycle is highly conserved among members of the cyclin family, the cell-cycle peak times are not necessarily conserved [1]: The closest homologs of *CIG2* in our *S. cerevisiae* dataset, are the G2/M promoter-encoding genes *CLB1,2* and *CLB3,4*, which expressions peak at G2/M and S respectively, and *CLB5*, which encodes a DNA synthesis promoter, and peaks at G1 (Figure 4*f*).

## Discussion

We mathematically defined a higher-order GSVD (HO GSVD) for two or more large-scale matrices with different row dimensions and the same column dimension. We proved that our new HO GSVD extends to higher orders almost all of the mathematical properties of the GSVD: The eigenvalues of 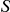 are always greater than or equal to one, and an eigenvalue of one corresponds to a right basis vector of equal significance in all matrices, and to a left basis vector in each matrix factorization that is orthogonal to all other left basis vectors in that factorization. We therefore mathematically defined, in analogy with the GSVD, the common HO GSVD subspace of the 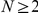 matrices to be the subspace spanned by the right basis vectors that correspond to the eigenvalues of 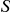 that equal one.

The only property that does not extend to higher orders in general is the complete column-wise orthogonality of the normalized left basis vectors in each factorization. Recent research showed that several higher-order generalizations are possible for a given matrix decomposition, each preserving some but not all of the properties of the matrix decomposition [12]–[14]. The HO GSVD has the interesting property of preserving the exactness and diagonality of the matrix GSVD and, in special cases, also partial or even complete column-wise orthogonality. That is, all 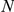 matrix factorizations in Equation (1) are exact, all 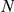 matrices 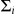 are diagonal, and when one or more of the eigenvalues of 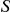 equal one, the corresponding left basis vectors in each factorization are orthogonal to all other left basis vectors in that factorization.

The complete column-wise orthogonality of the matrix GSVD [5] enables its stable computation [6]. We showed that each of the right basis vectors that span the common HO GSVD subspace is a generalized singular vector of all pairwise GSVD factorizations of the matrices 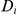 and 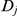 with equal corresponding generalized singular values for all 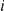 and 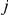. Since the GSVD can be computed in a stable way, the common HO GSVD subspace can also be computed in a stable way by computing all pairwise GSVD factorizations of the matrices 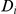 and 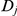. That is, the common HO GSVD subspace exists also for 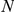 matrices 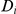 that are not all of full column rank. This also means that the common HO GSVD subspace can be formulated as a solution to an optimization problem, in analogy with existing variational formulations of the GSVD [33].

It would be ideal if our procedure reduced to the stable computation of the matrix GSVD when 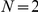. To achieve this ideal, we would need to find a procedure that allows a computation of the HO GSVD, not just the common HO GSVD subspace, for 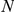 matrices 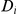 that are not all of full column rank. A formulation of the HO GSVD, not just the common HO GSVD subspace, as a solution to an optimization problem may lead to a stable numerical algorithm for computing the HO GSVD. Such a formulation may also lead to a higher-order general Gauss-Markov linear statistical model [34]–[36].

It was shown that the GSVD provides a mathematical framework for sequence-independent comparative modeling of DNA microarray data from two organisms, where the mathematical variables and operations represent experimental or biological reality [7], [8]. The variables, subspaces of significant patterns that are common to both or exclusive to either one of the datasets, correlate with cellular programs that are conserved in both or unique to either one of the organisms, respectively. The operation of reconstruction in the subspaces common to both datasets outlines the biological similarity in the regulation of the cellular programs that are conserved across the species. Reconstruction in the common and exclusive subspaces of either dataset outlines the differential regulation of the conserved relative to the unique programs in the corresponding organism. Recent experimental results [9] verify a computationally predicted genome-wide mode of regulation [10], [11], and demonstrate that GSVD modeling of DNA microarray data can be used to correctly predict previously unknown cellular mechanisms.

Here we showed, comparing global cell-cycle mRNA expression from the three disparate organisms *S. pombe*, *S. cerevisiae* and human, that the HO GSVD provides a sequence-independent comparative framework for two or more genomic datasets, where the variables and operations represent biological reality. The approximately common HO GSVD subspace represents the cell-cycle mRNA expression oscillations, which are similar among the datasets. Simultaneous reconstruction in the common subspace removes the experimental artifacts, which are dissimilar, from the datasets. In the simultaneous sequence-independent classification of the genes of the three organisms in this common subspace, genes of highly conserved sequences but significantly different cell-cycle peak times are correctly classified.

Additional possible applications of our HO GSVD in biotechnology include comparison of multiple genomic datasets, each corresponding to (*i*) the same experiment repeated multiple times using different experimental protocols, to separate the biological signal that is similar in all datasets from the dissimilar experimental artifacts; (*ii*) one of multiple types of genomic information, such as DNA copy number, DNA methylation and mRNA expression, collected from the same set of samples, e.g., tumor samples, to elucidate the molecular composition of the overall biological signal in these samples; (*iii*) one of multiple chromosomes of the same organism, to illustrate the relation, if any, between these chromosomes in terms of their, e.g., mRNA expression in a given set of samples; and (*iv*) one of multiple interacting organisms, e.g., in an ecosystem, to illuminate the exchange of biological information in these interactions.

## Supporting Information

Appendix S1 A PDF format file, readable by Adobe Acrobat Reader.
(PDF)

Mathematica Notebook S1 **Higher-order generalized singular value decomposition (HO GSVD) of global mRNA expression datasets from three different organisms.** A Mathematica 5.2 code file, executable by Mathematica 5.2 and readable by Mathematica Player, freely available at http://www.wolfram.com/products/player/.
(NB) Click here for additional data file.

Mathematica Notebook S2 **HO GSVD of global mRNA expression datasets from three different organisms.** A PDF format file, readable by Adobe Acrobat Reader.
(PDF)

Dataset S1
***S. pombe*** **global mRNA expression.** A tab-delimited text format file, readable by both Mathematica and Microsoft Excel, reproducing the relative mRNA expression levels of 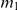 = 3167 *S. pombe* gene clones at 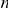 = 17 time points during about two cell-cycle periods from Rustici *et al.* [15] with the cell-cycle classifications of Rustici *et al.* or Oliva *et al.* [16].(TXT)

Dataset S2
***S. cerevisiae*** **global mRNA expression.** A tab-delimited text format file, readable by both Mathematica and Microsoft Excel, reproducing the relative mRNA expression levels of 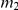 = 4772 *S. cerevisiae* open reading frames (ORFs), or genes, at 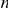 = 17 time points during about two cell-cycle periods, including cell-cycle classifications, from Spellman *et al.* [17].(TXT)

Dataset S3
**Human global mRNA expression.** A tab-delimited text format file, readable by both Mathematica and Microsoft Excel, reproducing the relative mRNA expression levels of 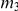 = 13,068 human genes at 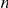 = 17 time points during about two cell-cycle periods, including cell-cycle classifications, from Whitfield *et al.* [18].(TXT)
