# Supplementary material for: A Higher-Order Generalized Singular Value Decomposition for Comparison of Global mRNA Expression from Multiple Organisms
Source: PLoS One. 2011 Dec 22;6(12):e28072. doi: 10.1371/journal.pone.0028072 (PMC3245232; doi:10.1371/journal.pone.0028072)
Supplement: Appendix S1 — A PDF format file, readable by Adobe Acrobat Reader. (PDF) [file pone.0028072.s001.pdf]

1. Mathematical Framework: HO GSVD

Comparative analyses of large-scale datasets promise to enhance our fundamental understanding of the data by distinguishing the similar from the dissimilar among these data. For example, comparative analyses of global mRNA expression from multiple model organisms promise to enhance fundamental understanding of the universality and specialization of molecular biological mechanisms, and may prove useful in medical diagnosis, treatment and drug design [1]. Existing algorithms limit analyses to subsets of homologous genes among the different organisms, effectively introducing into the analysis the assumption that sequence and functional similarities are equivalent [2]. For sequence-independent comparisons [3], mathematical frameworks are required that can distinguish the similar from the dissimilar among multiple large-scale datasets tabulated as matrices with the same column dimension and different row dimensions, corresponding to the different sets of genes of the different organisms. The only such framework to date, that of the generalized singular value decomposition (GSVD) [4–6] is limited to two matrices.

Recently we showed that the GSVD provides a comparative mathematical framework for global mRNA expression datasets from two different organisms, tabulated as two matrices with the same column dimension and different row dimensions, where the mathematical variables and operations represent biological reality [7]. In this application, one matrix tabulates DNA microarray-measured genome-scale mRNA expression from the yeast *S. cerevisiae*, sampled at n time points at equal time intervals during the cell-cycle program. This matrix is of size m_1-*S. cerevisiae* genes $\times$ n-DNA microarrays. The second matrix tabulates data from the HeLa human cell line, sampled at the same number of time points, also at equal time intervals, and is of size m_2-human genes $\times$ n-arrays. The underlying assumption of the GSVD as a comparative mathematical framework for the two matrices is that there exists a one-to-one mapping among the columns of the matrices, but not necessarily among their rows. The GSVD factors each matrix into a product of an organism-specific matrix of size m_1-*S. cerevisiae* genes or m_2-human genes $\times$ n-"arraylets," i.e., left basis vectors, an organism-specific diagonal matrix of size n-arraylets $\times$ n-"genelets," i.e., right basis vectors, and a shared matrix of size n-genelets $\times$ n-arrays.

We showed that the mathematical variables of the GSVD, i.e., the patterns of the genelets and the two sets of arraylets, represent either the similar or the dissimilar among the biological programs that compose the *S. cerevisiae* and human datasets. Genelets of common significance in both datasets, and the corresponding arraylets, represent cell-cycle checkpoints that are common to *S. cerevisiae* and human. Simultaneous reconstruction and classification of both the *S. cerevisiae* and human data in the common subspace that these patterns span outlines the biological similarity in the regulation of their cell-cycle programs. Patterns almost exclusive to either dataset correlate with either the *S. cerevisiae* or the human exclusive synchronization responses. Reconstruction of either dataset in the subspaces of the common vs. exclusive patterns represents differential gene expression in the *S. cerevisiae* and human conserved cell-cycle programs vs. their unique synchronization-response programs, respectively. Notably, relations such as these between the expression profiles of the *S. cerevisiae* genes *KAR4* and *CIK1*, which are known to be correlated in response to the synchronizing agent, the α-factor pheromone, yet anticorrelated during cell division, are correctly depicted.

We now define a higher-order GSVD (HO GSVD) of $N \geq 2$ datasets, tabulated as N real matrices D_i with the same column dimension and, in general, different row dimensions. In our form of the HO GSVD, each data matrix D_i is assumed to have full column rank and is factored as the product $D_i = U_i \Sigma_i V^T$, where U_i is the same shape as D_i (rectangular), Σ_i is diagonal and positive definite, and V is square and nonsingular. The columns of U_i have unit length; we call them the "left basis vectors" for D_i (a different set for each i). The columns of V also have unit length; we call them "right basis vectors," and they are the same in all factorizations. In the application of the HO GSVD to a comparison of global mRNA expression from $N \geq 2$ organisms, the right basis vectors are the genelets and the N sets of left basis vectors are the N sets of arraylets. We use the notation:

$$U_i \equiv (u_{i,1} \ \ldots \ u_{i,n}), \qquad \|u_{i,k}\| = 1, \quad (1)$$
$$\Sigma_i \equiv \operatorname{diag}(\sigma_{i,k}), \qquad \sigma_{i,k} > 0, \quad (2)$$
$$V \equiv (v_1 \ \ldots \ v_n), \qquad \|v_k\| = 1. \quad (3)$$

We call $\{\sigma_{i,k}\}$ the "higher-order generalized singular value set." They are weights in the following sums of rank-one matrices of unit norm:

$$D_i = U_i \Sigma_i V^T = \sum_k \sigma_{i,k} u_{i,k} v_k^T, \quad \|u_{i,k} v_k^T\| = 1. \quad (4)$$

Hence we regard the kth values $\sigma_{i,k}$ as indicating the significance of the kth right basis vector v_k in the matrices D_i (reflecting the overall information that v_k captures in each D_i in turn). To obtain the factorizations, we work with the matrices $A_i = D_i^T D_i$ and the matrix sum S, defined as the arithmetic mean of all pairwise quotients $A_i A_j^{-1}$, or equivalently of all $S_{ij} = \frac{1}{2}(A_i A_j^{-1} + A_j A_i^{-1})$, $i \neq j$. The eigensystem $SV = V\Lambda$ is used to define V, and the factors U_i and Σ_i are computed from D_i and V.

To clarify our choice of S, we note that in the GSVD, defined by Van Loan [4], the matrix V can be formed from the eigenvectors of the unbalanced quotient $A_1 A_2^{-1}$ (Supplementary Section 1.1). We observe that this V can also be formed from the eigenvectors of the balanced arithmetic mean $S_{12} = \frac{1}{2}(A_1 A_2^{-1} + A_2 A_1^{-1})$. We prove that in the case of $N = 2$, our definition of V by using the eigensystem of $S \equiv S_{12} = \frac{1}{2}(A_1 A_2^{-1} + A_2 A_1^{-1})$ leads algebraically to the GSVD and therefore, as Paige and

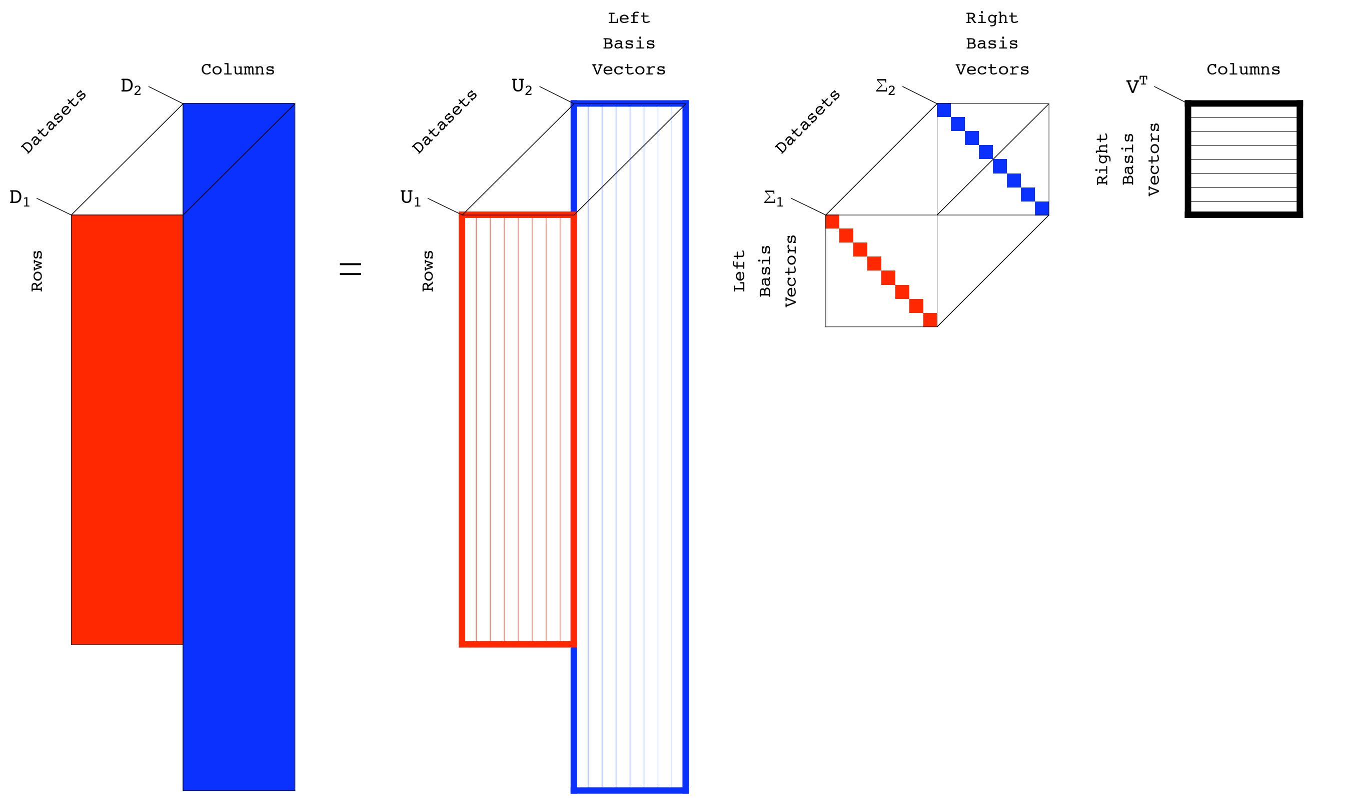

Supplementary Figure S1. The GSVD of two matrices D_1 and D_2 is reformulated as a linear transformation of the two matrices from the two rows $\times$ columns spaces to two reduced and diagonalized left basis vectors $\times$ right basis vectors spaces. The right basis vectors are shared by both datasets. Each right basis vector corresponds to two left basis vectors.

Saunders showed [5], can be computed in a stable way. We also note that in the GSVD, the matrix V is invariant under the exchange of the two matrices D_1 and D_2.

Therefore, we define our HO GSVD for $N \geq 2$ matrices by using the balanced arithmetic mean S of all pairwise arithmetic means S_{ij}, each of which defines the GSVD of the corresponding pair of matrices D_i and D_j, noting that S is invariant under the exchange of any two matrices D_i and D_j.

We also show that the existing SVD and GSVD decompositions are in some sense special cases of our HO GSVD (Supplementary Section 1.2). Finally, we conjecture a role for our exact HO GSVD in iterative approximation algorithms (Supplementary Section 1.3).

1.1. The Matrix GSVD

1.1.1. ***Construction of the matrix GSVD.*** Suppose we have two real matrices $D_1 \in \mathbb{R}^{m_1 \times n}$ and $D_2 \in \mathbb{R}^{m_2 \times n}$ each with full column rank. Van Loan [4] defined the GSVD of D_1 and D_2 as

$$\begin{aligned} U_1^T D_1 X &\equiv \Sigma_1, \\ U_2^T D_2 X &\equiv \Sigma_2, \end{aligned} \tag{5}$$

where each $U_i \in \mathbb{R}^{m_i \times n}$ has orthonormal columns, $X \in \mathbb{R}^{n \times n}$ is nonsingular, and the $\Sigma_i = \mathrm{diag}(\sigma_{i,k}) \in \mathbb{R}^{n \times n}$ are diagonal with $\sigma_{i,k} > 0$ $(i = 1, 2)$. Paige and Saunders [5] showed that the GSVD can be computed in a stable way by orthogonal transformations. In the full column-rank case it takes the form

$$\begin{aligned} D_1 &\equiv U_1 \Sigma_1 V^T, \\ D_2 &\equiv U_2 \Sigma_2 V^T, \end{aligned} \tag{6}$$

where U_1, U_2 and $\begin{bmatrix} \Sigma_1 \\ \Sigma_2 \end{bmatrix}$ have orthonormal columns [6], and V is square and nonsingular.

We work with the form of Supplementary Equation (6), but we find it useful and more similar to the standard SVD [6] if we assume that the columns of V are scaled to have unit length, with the columns of Σ_i scaled accordingly. Note that the ratios $\sigma_{1,k}/\sigma_{2,k}$ are not altered by the scaling.

In place of the methods of Van Loan [4] and Paige and Saunders [5], we construct the GSVD of Supplementary Equation (6) as follows. We obtain V from the eigensystem of S, the arithmetic mean of the quotients $A_1 A_2^{-1}$ and $A_2 A_1^{-1}$ of the matrices $A_1 = D_1^T D_1$ and $A_2 = D_2^T D_2$:

$$\begin{aligned} S &\equiv S_{12} = \tfrac{1}{2}(A_1 A_2^{-1} + A_2 A_1^{-1}), \\ SV &= V\Lambda, \\ V &\equiv (v_1 \ \ldots \ v_n), \qquad \Lambda = \mathrm{diag}(\lambda_k), \end{aligned} \tag{7}$$

with $\|v_k\| = 1$. Given V, we compute matrices B_i by solving two linear systems

$$\begin{aligned} VB_i^T &= D_i^T, \\ B_i &\equiv (b_{i,1} \ \ldots \ b_{i,n}), \qquad i = 1, 2, \end{aligned} \tag{8}$$

and we construct Σ_i and $U_i = (u_{i,1} \ \ldots \ u_{i,n})$ by normalizing the columns of B_i:

$$\begin{aligned} \sigma_{i,k} &= \|b_{i,k}\|, \\ \Sigma_i &= \mathrm{diag}(\sigma_{i,k}), \\ B_i &= U_i \Sigma_i. \end{aligned} \tag{9}$$

We prove below that S is nondefective (it has n independent eigenvectors) and its eigensystem is real.

From Supplementary Equations (8) and (9) we have $D_i = B_i V^T = U_i \Sigma_i V^T$ as in Supplementary Equation (6). We see that the rows of both D_1 and D_2 are superpositions of the same right basis vectors, the columns of V (Supplementary Figure S1). This is the construction that we generalize in Equations (1)–(4) to compute our HO GSVD.

1.1.2. ***Interpretation of the GSVD construction.*** In our GSVD comparison of two matrices, we interpreted the kth diagonals of Σ_1 and Σ_2, i.e., the "generalized singular value pair" $(\sigma_{1,k}, \sigma_{2,k})$, as indicating the significance of the kth right basis vector v_k in the matrices D_1 and D_2, and reflecting the overall information that v_k captures in D_1 and D_2 respectively [7]. The ratio $\sigma_{1,k}/\sigma_{2,k}$ indicates the significance of v_k in D_1 relative to its significance in D_2.

A ratio of $\sigma_{1,k}/\sigma_{2,k} = 1$ corresponds to a basis vector v_k of equal significance in D_1 and D_2. GSVD comparisons of two matrices showed that right basis vectors of approximately equal significance in both matrices reflect themes that are common to the two matrices under comparison [7].

A ratio of $\sigma_{1,k}/\sigma_{2,k} \gg 1$ corresponds to a basis vector v_k of almost negligible significance in D_2 relative to its significance in D_1. Likewise, a ratio of $\sigma_{1,k}/\sigma_{2,k} \ll 1$ indicates a basis vector v_k of almost negligible significance in D_1 relative to its significance in D_2. GSVD comparisons of two matrices showed that right basis vectors of negligible significance in one matrix reflect themes that are exclusive to the other matrix.

1.1.3. ***Mathematical properties of Λ and V in the GSVD construction.*** Note that our GSVD construction in Supplementary Equations (7)–(9) is well defined for any square nonsingular V. We now show that our particular V leads algebraically to the GSVD of Supplementary Equation (6), ignoring the rescaled columns of Σ_i and V. Recall that $A_i = D_i^T D_i$ and $D_i = U_i \Sigma_i V^T$ with Σ_i diagonal.

In practice we would prefer not to form A_i or S directly. Instead we may work with the QR factorizations $D_i = Q_i R_i$, where $Q_i^T Q_i = I$ and R_i is upper triangular and nonsingular. Define another triangular matrix R and its SVD to be

$$R \equiv R_1 R_2^{-1} = \widetilde{U} \widetilde{\Sigma} \widetilde{V}^T, \tag{10}$$

where $\widetilde{U}$ and $\widetilde{V}$ are square and orthogonal, and $\widetilde{\Sigma} = \mathrm{diag}(\widetilde{\sigma}_k)$, $\widetilde{\sigma}_k > 0$. We then have

$$\begin{aligned} S &= \tfrac{1}{2}[(R_1^T R_1)(R_2^T R_2)^{-1} + (R_2^T R_2)(R_1^T R_1)^{-1}], \\ R_1^{-T} S R_1^T &= \tfrac{1}{2}[R_1 (R_2^T R_2)^{-1} R_1^T + R_1^{-T} (R_2^T R_2) R_1^{-1}] \\ &= \tfrac{1}{2}[RR^T + (RR^T)^{-1}] \\ &= \widetilde{U} \Lambda \widetilde{U}^T, \end{aligned} \tag{11}$$

where $\Lambda \equiv \frac{1}{2}(\widetilde{\Sigma}^2 + \widetilde{\Sigma}^{-2}) \equiv \mathrm{diag}(\lambda_k)$. Thus

$$S(R_1^T \widetilde{U}) = (R_1^T \widetilde{U}) \Lambda, \tag{12}$$

$$SV = V\Lambda, \qquad V \equiv R_1^T \widetilde{U} D, \tag{13}$$

where the diagonal matrix D normalizes $R_1^T \widetilde{U}$ so that V has columns of unit length.

Supplementary Theorem S1. *The matrices U_1 and U_2 constructed in Supplementary Equation (9) have orthonormal columns ($U_1^T U_1 = U_2^T U_2 = I$).*

Proof. From Supplementary Equation (8) we have $VB_i^T B_i V^T = D_i^T D_i$, so that

$$\begin{aligned} (R_1^T \widetilde{U} D) B_i^T B_i (D \widetilde{U}^T R_1) &= R_i^T R_i \\ \Rightarrow \quad D B_1^T B_1 D &= I, \\ D B_2^T B_2 D &= \widetilde{U}^T R_1^{-T} (R_2^T R_2) R_1^{-1} \widetilde{U} = \widetilde{\Sigma}^{-2}, \end{aligned}$$

with help from Supplementary Equations (10), (12) and (13). We see that both $B_i^T B_i$ are diagonal, and the quantities in Supplementary Equation (9) must be

$$U_1 = B_1 D, \qquad \Sigma_1 = D^{-1}, \tag{14}$$

$$U_2 = B_2 D \widetilde{\Sigma}, \qquad \Sigma_2 = \widetilde{\Sigma}^{-1} D^{-1}, \tag{15}$$

with U_i column-wise orthonormal. □

Supplementary Theorem S2. *The eigenvalues of S satisfy $\lambda_k \geq 1$, and S has a full set of n eigenvectors (it is nondefective). Also, the eigenvectors are real.*

Proof. The equivalence transformation of Supplementary Equation (11) shows that S has the same eigenvalues as $\widetilde{U} \Lambda \widetilde{U}^T$, namely Λ. From the definition of Λ and $\widetilde{\Sigma}$, the eigenvalues are $\lambda_k = \frac{1}{2}(\widetilde{\sigma}_k^2 + 1/\widetilde{\sigma}_k^2) \geq 1$. Also, in the eigensystem of S in Supplementary Equation (13), $V = R_1^T \widetilde{U} D$ is a product of real nonsingular matrices and hence is real and nonsingular. □

Supplementary Theorem S3. *An eigenvalue $\lambda_k = 1$ corresponds to a right basis vector v_k of equal significance in both matrices D_1 and D_2. That is, $\sigma_{1,k}/\sigma_{2,k} = 1$.*

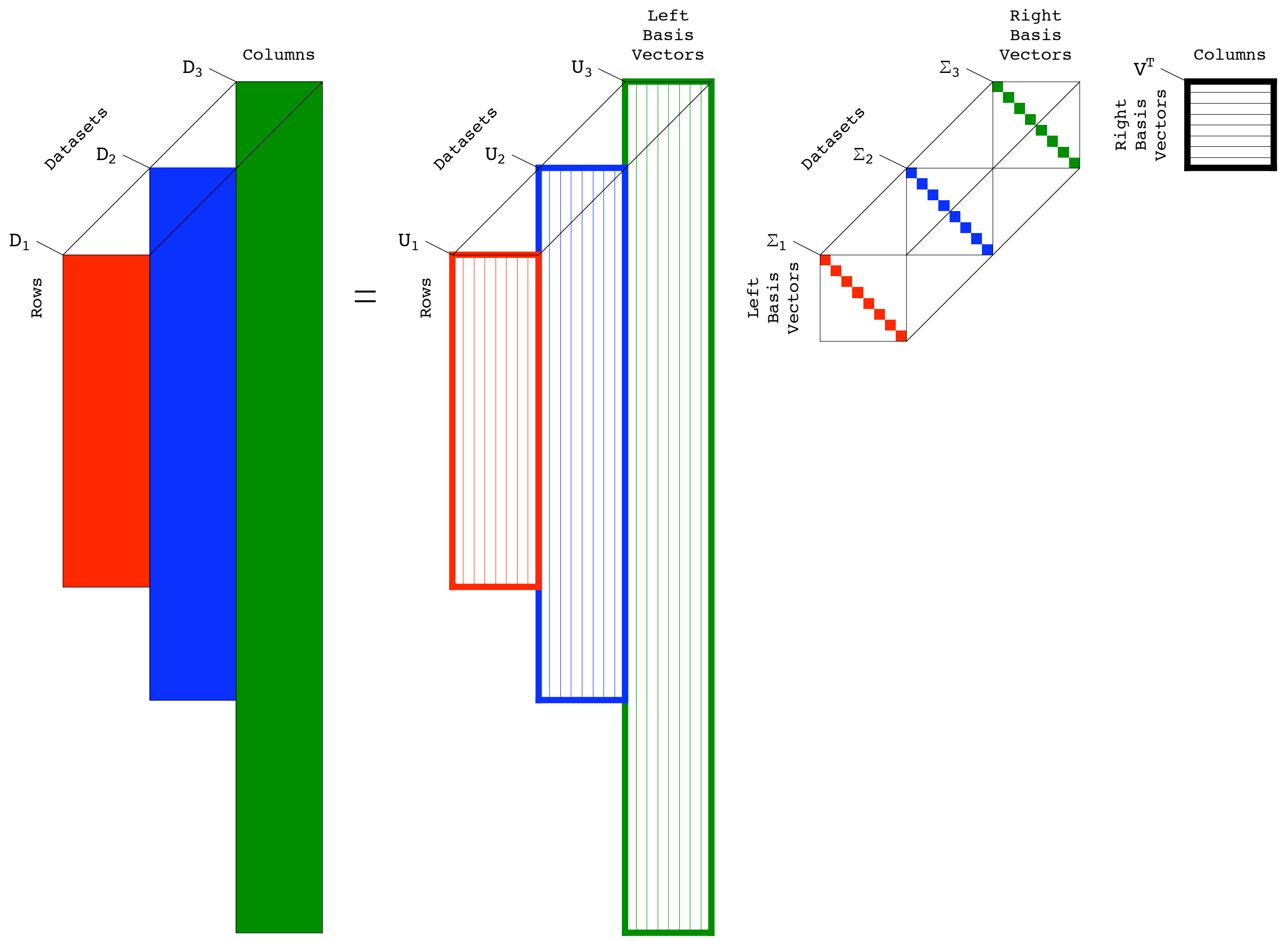

Supplementary Figure S2. The higher-order GSVD (HO GSVD) of three matrices D_1, D_2, and D_3 is a linear transformation of the three matrices from the three rows × columns spaces to three reduced and diagonalized left basis vectors × right basis vectors spaces. The right basis vectors are shared by all three datasets. Each right basis vector corresponds to three left basis vectors.

Proof. From the orthonormality of U_1 and U_2 of Equations (14) and (15), and our GSVD construction of Equations (7)–(9), we have $\lambda_k = (\sigma_{1,k}^2/\sigma_{2,k}^2 + \sigma_{2,k}^2/\sigma_{1,k}^2)/2$. Therefore, $\lambda_k = 1$ can occur only if $\sigma_{1,k}/\sigma_{2,k} = 1$. In other words, $\lambda_k = 1$ if the kth right basis vector v_k is equally significant in D_1 and D_2. □

Note that the GSVD is a generalization of the SVD in that if one of the matrices is the identity matrix, the GSVD reduces to the SVD of the other matrix.

In Equations (1)–(4), we now define a HO GSVD and in Theorems 1–3 and Corollary 1 we show that this new decomposition extends to higher orders all of the mathematical properties of the GSVD except for complete column-wise orthogonality of the left basis vectors that form the matrices U_i for all i. We proceed in the same way as in Supplementary Equations (7)–(9).

1.2. The Matrix GSVD and SVD as Special Cases

Let us now show that the GSVD and the standard SVD are special cases of our HO GSVD.

Supplementary Theorem S4. *Suppose matrices D and D_j are real and have full column rank. The HO GSVD of N matrices satisfying $D_i = D$ for all $i \neq j$ reduces to the GSVD of the two matrices D and D_j.*

Proof. Substituting $D_i = D$ for all $i \neq j$ in Equation (2), we obtain the matrix sum $S = \frac{1}{N}[AA_j^{-1} + A_jA^{-1} + (N-2)I]$, with $A = D^TD$. The eigenvectors of S are the same as the eigenvectors V of $\frac{1}{2}(AA_j^{-1} + A_jA^{-1})$, and Supplementary Theorem S2 shows that V exists. Solving two linear systems for B and B_j and normalizing the solutions,

$$
\begin{aligned}
VB^T &= D_i^T = D^T, \qquad & B &= U\Sigma, \\
VB_j^T &= D_j^T, \qquad & B_j &= U_j\Sigma_j,
\end{aligned}
$$

reduces the HO GSVD of Equation (1) to the GSVD of

D and D_j of Supplementary Equation (6):

$$D_i = D = U\Sigma V^T, \qquad i \neq j,$$
$$D_j = U_j\Sigma_j V^T.$$

Supplementary Theorem S1 shows that the columns of U and U_j are orthonormal. □

Supplementary Theorem S5. *Suppose the matrix D_j is real and has full column rank. The HO GSVD of N matrices satisfying $D_i = I$ for all $i \neq j$ reduces to the SVD of D_j.*

Proof. Substituting $D_i = I$ for all $i \neq j$ in Supplementary Equation (2), we obtain the matrix sum $S = \frac{1}{N}[A_j + A_j^{-1} + (N-2)I]$. The symmetry of S implies that its eigenvectors V exist and are orthonormal. Computing the matrix B_j from

$$VB_j^T = D_j^T, \qquad B_j = U_j\Sigma_j,$$

gives $D_j = U_j\Sigma_j V^T$, and Supplementary Theorem S1 shows that the columns of U_j are orthonormal. Hence the factorization must be the SVD of D_j [6]. □

1.3. Role in Approximation Algorithms

Recent research showed that several higher-order generalizations are possible for a given matrix decomposition, each preserving only some but not all of the properties of the matrix decomposition [12, 13].

Our HO GSVD preserves the exactness as well as the diagonality of the matrix GSVD, i.e., all N matrix factorizations in Equation (1) are exact and all N matrices Σ_i are diagonal. In general, our HO GSVD does not preserve the orthogonality of the matrix GSVD, i.e., the matrices U_i in Equation (1) are not necessarily column-wise orthonormal. For some applications, however, one might want to preserve the orthogonality instead of the exactness of the matrix GSVD. An iterative approximation algorithm might be used to compute for a set of $N > 2$ real matrices $D_i \in \mathbb{R}^{m_i \times n}$, each with full column rank, an approximate decomposition

$$\begin{aligned} D_1 &\approx U_1\Sigma_1 V^T, \\ D_2 &\approx U_2\Sigma_2 V^T, \\ &\vdots \\ D_N &\approx U_N\Sigma_N V^T, \end{aligned} \tag{16}$$

where each $U_i \in \mathbb{R}^{m_i \times n}$ is composed of orthonormal columns, each $\Sigma_i = \text{diag}(\sigma_{i,k}) \in \mathbb{R}^{n \times n}$ is diagonal with $\sigma_{i,k} > 0$, and V is identical in all N matrix factorizations.

If there exist an exact decomposition of Equation (1) where the matrices U_i are column-wise orthonormal, then it is reasonable to expect that the iterative approximation algorithm will converge to that exact decomposition. More than that, when the iterative approximation algorithm is initialized with the exact decomposition, it is reasonable to expect convergence in just one iteration. We show below that if there exist an exact decomposition of Equation (1) in which the matrices U_i are column-wise orthonormal, our HO GSVD leads algebraically to that exact decomposition.

Supplementary Theorem S6. *If there exist an exact HO GSVD of Equation (1) where the matrices U_i are column-wise orthonormal, then our particular V leads algebraically to the exact decomposition.*

Proof. If there exist an exact decomposition with column-wise orthonormal U_i for all i, then $A_i = D_i^T D_i = V\Sigma_i^2 V^T$ and the right basis vectors, i.e., the columns of V, simultaneously diagonalize all pairwise quotients $A_i A_j^{-1} V = V\Sigma_i^2\Sigma_j^{-2}$ as well as their arithmetic mean $SV = V\Lambda$.

Therefore, the V of the eigensystem of S in Equation (2) is equivalent to the V of the HO GSVD of Equation (1) with column-wise orthonormal U_i for all i. □

We conjecture, therefore, the following role for our exact HO GSVD in iterative approximation algorithms.

Supplementary Conjecture S1. *An iterative approximation algorithm will converge to the optimal approximate decomposition of Supplementary Equation (16) in a significantly reduced number of iterations when initialized with our exact HO GSVD, rather than with random U_i, Σ_i and V.*

2. Biological Application: Comparison of Global mRNA Expression Datasets from Three Different Organisms

2.1. *S. pombe*, *S. cerevisiae* and Human Datasets

Rustici *et al.* [15] monitored mRNA levels in the yeast *Schizosaccharomyces pombe* over about two cell-cycle periods, in a culture synchronized initially by the *cdc25-22* block-release late in the cell-cycle phase G_2, relative to reference mRNA from an asynchronous culture, at 15min intervals for 240min. The *S. pombe* dataset we analyze (Supplementary Dataset S1) tabulates the ratios of gene expression levels for the m_1=3167 gene clones with no missing data in at least 14 of the n=17 arrays. Of these, the mRNA expression of 380 gene clones was classified as cell cycle-regulated by Rustici *et al.* or Oliva *et al.* [16].

Spellman *et al.* [17] monitored mRNA expression in the yeast *Saccharomyces cerevisiae* over about two cell-cycle periods, in a culture synchronized initially by the α-factor pheromone in the cell-cycle phase M/G_1, relative to reference mRNA from an asynchronous culture, at 7min intervals for 112min. The *S. cerevisiae* dataset we analyze (Supplementary Dataset S2) tabulates the ratios of gene expression levels for the m_2=4772 open reading frames (ORFs), or genes, with no missing data in at least 14 of the n=17 arrays. Of these, the mRNA expression of 641 ORFs was traditionally or microarray-classified as cell cycle-regulated.

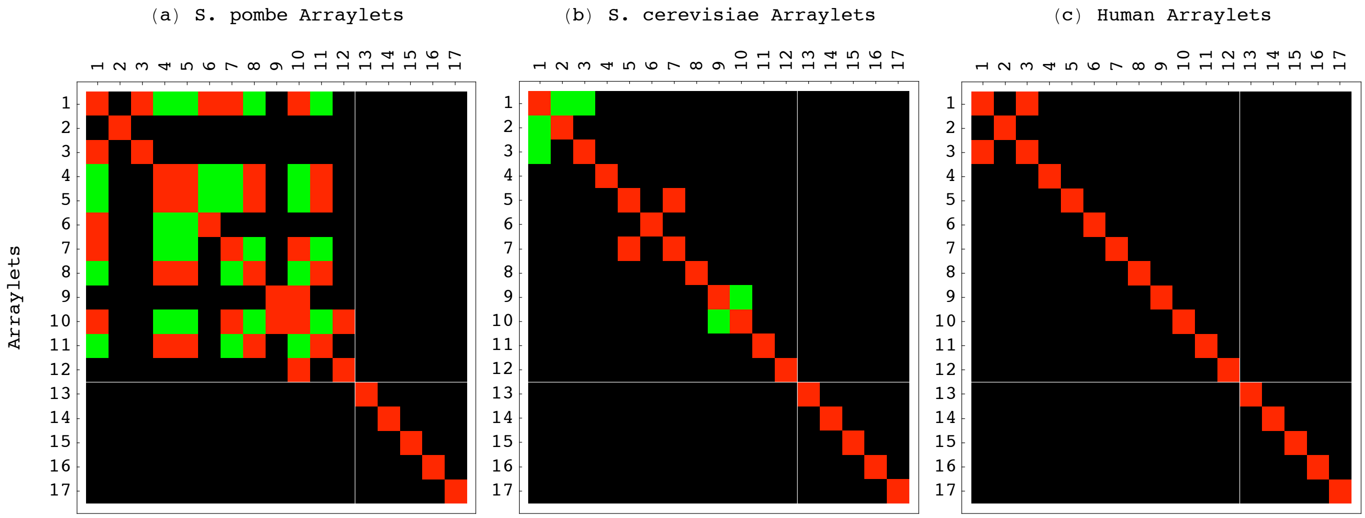

Supplementary Figure S3. Correlations among the n=17 arraylets in each organism. Raster displays of $U_i^T U_i$, with correlations $\geq \epsilon = 0.33$ (red), $\leq -\epsilon$ (green) and $\in (-\epsilon, \epsilon)$ (black), show that the arraylets $u_{i,k}$ with $k = 13, \ldots, 17$ that correspond to $1 \lesssim \lambda_k \lesssim 2$, are $\epsilon = 0.33$-orthonormal to all other arraylets in each dataset. The corresponding five genelets v_k are approximately equally significant with $\sigma_{1,k} : \sigma_{2,k} : \sigma_{3,k} \sim 1 : 1 : 1$ in the *S. pombe*, *S. cerevisiae* and human datasets, respectively (Figure 2). Following Theorem 3, therefore, these genelets span the approximately "common HO GSVD subspace" for the three datasets.

Whitfield *et al.* [18] monitored mRNA levels in the human HeLa cell line over about two cell-cycle periods, in a culture synchronized initially by a double thymidine-block in S-phase, relative to reference mRNA from an asynchronous culture, at 2hr intervals for 34hr. The human dataset we analyze (Supplementary Dataset S3) tabulates the ratios of gene expression levels for the m_3=13,068 gene clones with no missing data in at least 14 of the n=17 arrays. Of these, the mRNA expression of 787 gene clones was classified as cell cycle-regulated.

Of the 53,839, 81,124 and 222,156 elements in the *S. pombe*, *S. cerevisiae* and human data matrices, 2420, 2936 and 14,680 elements, respectively, i.e., ∼5%, are missing valid data. SVD [6] is used to estimate the missing data as described [7]. In each of the data matrices, SVD of the expression patterns of the genes with no missing data uncovered 17 orthogonal patterns of gene expression, i.e., "eigengenes." The five most significant of these patterns, in terms of the fraction of the mRNA expression that they capture, are used to estimate the missing data in the remaining genes. For each of the three data matrices, the five most significant eigengenes and their corresponding fractions are almost identical to those computed after the missing data are estimated, with the corresponding correlations >0.95 (Supplementary Mathematica Notebooks S1 and S2). This suggests that the five most significant eigengenes, as computed for the genes with no missing data, are valid patterns for estimation of missing data. This also indicates that this SVD estimation of missing data is robust to variations in the data selection cutoffs.

We compute the HO GSVD of the three data matrices after missing data estimation by using Equations (1)–(4). Following Theorem 3, we find that the approximately common HO GSVD subspace of the three datasets is spanned by the five genelets v_k $k = 13, \ldots, 17$ (Figure 2 and Supplementary Figure S3).

2.2. Common Subspace Interpretation

In analogy with the GSVD [7], a common HO GSVD genelet and the $N = 3$ corresponding arraylets, which are approximately orthonormal to all other arraylets in the corresponding datasets (Theorem 3), are inferred to represent a biological process common to the three organisms and the corresponding cellular states when a consistent biological or experimental theme is reflected in the interpretations of the patterns of the genelet and the arraylets.

A genelet v_k is associated with a biological process or an experimental artifact when its pattern of expression variation across the arrays, i.e., across time, is interpretable. An arraylet $u_{i,k}$ is parallel- and antiparallel-associated with the most likely parallel and antiparallel cellular states according to the annotations of the two groups of m genes each, with largest and smallest levels of expression in this arraylet among all m_i genes, respectively. The P-value of a given association, i.e., $P(y; m, m_i, z)$, is calculated assuming hypergeometric probability distribution of the z annotations among the m_i genes, and of the subset of $y \subseteq z$ annotations among the subset of m genes, as described [39],

$$P(y; m, m_i, z) = \binom{m_i}{m}^{-1} \sum_{x=y}^{m} \binom{z}{x} \binom{m_i - z}{m - x}. \quad (17)$$

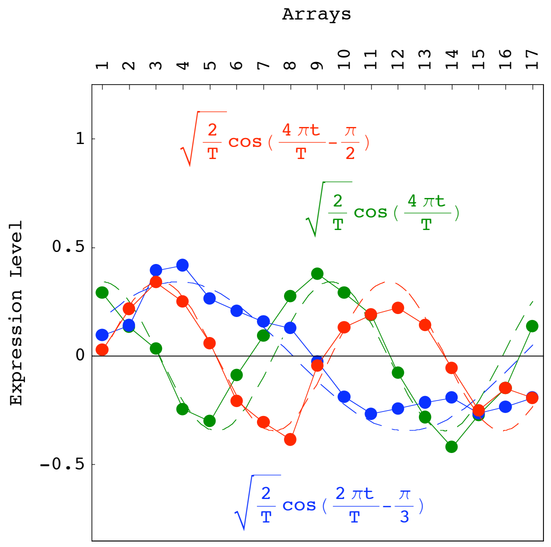

Supplementary Figure S4. The three-dimensional least-squares approximation of the five-dimensional approximately common HO GSVD subspace. Line-joined graphs of the first (red), second (blue) and third (green) most significant orthonormal vectors in the least squares approximation of the genelets v_k with $k = 13, \ldots, 17$, which span the common HO GSVD subspace. We approximate this five-dimensional subspace with the two orthonormal vectors x (green) and y (red), which fit normalized cosine functions of two periods, and 0- and $-\pi/2$-initial phases, i.e., normalized zero-phase cosine and sine functions of two periods, respectively.

We find that the approximately common HO GSVD subspace represents cell-cycle mRNA expression in the three disparate organisms (Figure 2 and Table 1).

2.3. HO GSVD Data Reconstruction

The decoupling of the HO GSVD genelets and N sets of arraylets, i.e., the diagonality of the matrices Σ_i in Equation (1), allows simultaneous reconstruction of the $N = 3$ datasets in the common HO GSVD subspace without eliminating genes or arrays.

In analogy with the GSVD [7], given a common HO GSVD subspace that is spanned by the K genelets $\{v_k\}$ where $k = n - K + 1, \ldots, n$, we reconstruct each dataset in terms of only these genelets and the corresponding $\{u_{i,k}\}$ arraylets,

$$D_i = U_i \Sigma_i V^T = \sum_{k=1}^{n} \sigma_{i,k} u_{i,k} v_k^T \rightarrow \sum_{k=n-K+1}^{n} \sigma_{i,k} u_{i,k} v_k^T. \qquad (18)$$

Note that this reconstruction is mathematically equivalent to setting to zero the higher-order generalized singular values $\{\sigma_{i,k}\}$ in Σ_i for all $k \neq n - K + 1, \ldots, n$, and then multiplying the matrices $U_i \Sigma_i V^T$ to obtain the reconstructed D_i.

2.4. Simultaneous HO GSVD Classification

Identifying the subset of genelets and the corresponding arraylets that span the approximately common HO GSVD subspace allows simultaneous classification of the genes and arrays of the three datasets by similarity in their expression of these genelets or corresponding arraylets, respectively, rather than by their overall expression, as described [7].

We least squares-approximate the K=5-dimensional subspace spanned by the five genelets v_k with $k = 13, \ldots, 17$, with the two-dimensional space spanned by two of the three orthonormal vectors x, y and $z \in \mathbb{R}^n$ that maximize the norm $\sum_{k=n-K+1}^{n} (\|v_k^T x\|^2 + \|v_k^T y\|^2 + \|v_k^T z\|^2)$ (Supplementary Figure S4). Since the common HO GSVD subspace represents cell-cycle mRNA expression, the two vectors that we select to approximate the common subspace, x and y, describe expression oscillations of two periods in the three time courses.

We plot the projection of each gene of the dataset D_i from the K-genelets subspace onto y, i.e., $e_m^T \sum_{k=n-K+1}^{n} (\sigma_{i,k} u_{i,k} v_k^T) y / a_m$ where e_{m_i} is a unit m_i-vector, along the y-axis vs. that onto x along the x-axis, normalized by its ideal amplitude a_m, where the contribution of each genelet to the overall projected expression of the gene adds up rather than cancels out, $a_m^2 = \sum_k \sum_j |\sigma_{i,k} \sigma_{i,j} (e_{m_i}^T u_{i,k} u_{i,j}^T e_{m_i}) v_k^T (x x^T + y y^T) v_j|$. In this plot, the distance of each gene from the origin is the amplitude of its normalized projection. A unit amplitude indicates that the genelets add up; a zero amplitude indicates that they cancel out. The angular distance of each gene from the x-axis is its phase in the progression of expression across the genes from x to y and back to x, going through the projection of each genelet v_k in this subspace, i.e., $(x x^T + y y^T) v_k$. Sorting the genes according to these angular distances gives the angular order, or classification, of the genes.

Similarly, we plot the projection of each array from the K-arraylets subspace onto $\sum_{k=n-K+1}^{n} (u_{i,k} v_k^T) y$, i.e., $y^T \sum_{k=n-K+1}^{n} (\sigma_{i,k} v_k v_k^T) e_n$ where e_n is a unit n-vector, along the y-axis vs. that onto $\sum_{k=n-K+1}^{n} (u_{i,k} v_k^T) x$ along the x-axis, normalized by its ideal amplitude a_n, where the contribution of each arraylet to the overall projected expression of the array adds up rather than cancels out, $a_n^2 = \sum_k \sum_j |\sigma_{i,k} \sigma_{i,j} (e_n^T v_k v_j^T e_n) v_k^T (x x^T + y y^T) v_j|$. We sort the arrays according to their angular distances from the x-axis.

For classification, we set to zero the arithmetic mean of each genelet across the arrays, i.e., time, and that of each arraylet across the genes, such that the expression of each gene and array is centered at its time- or gene-invariant level, respectively.

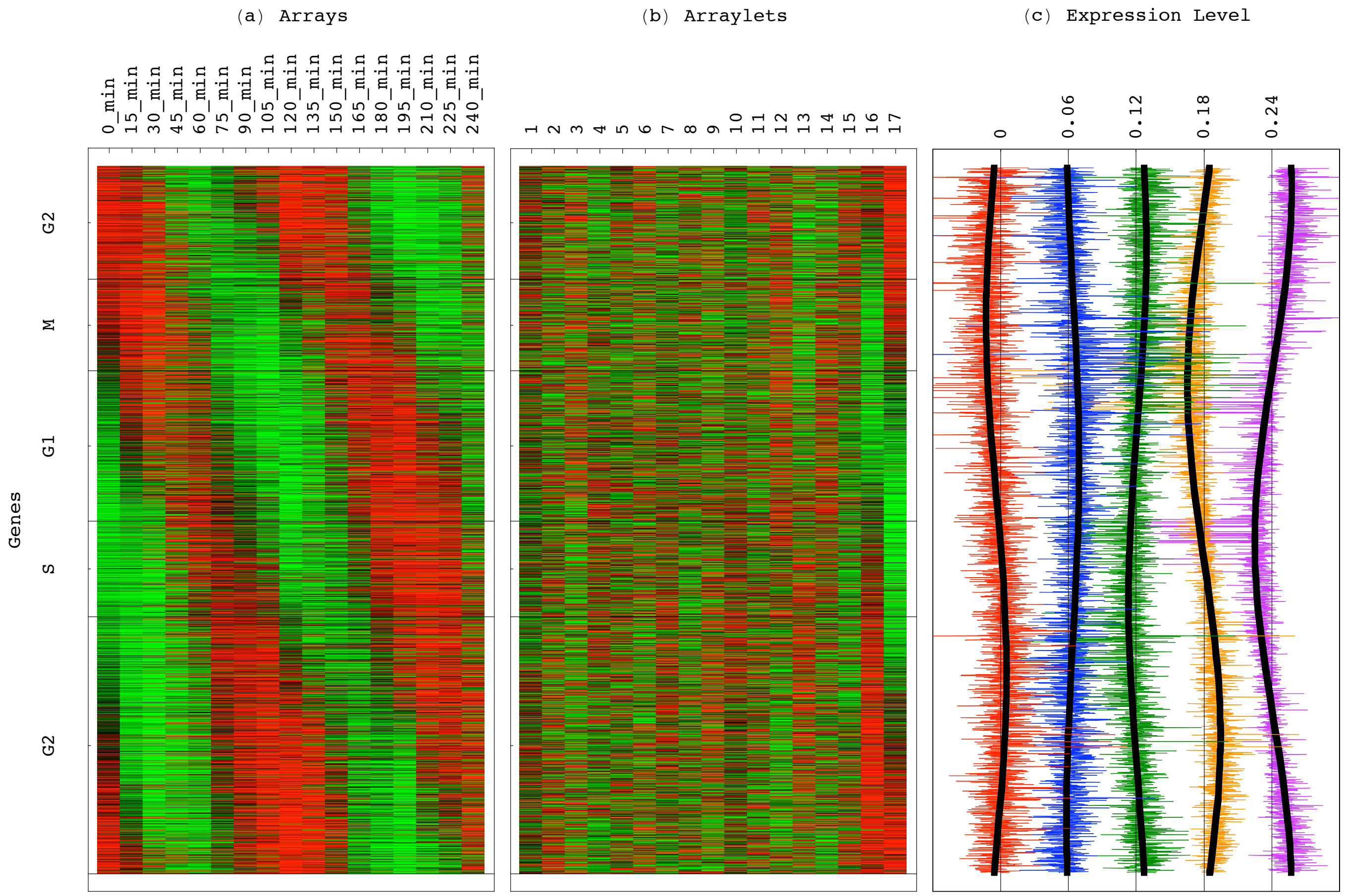

Supplementary Figure S5. *S. pombe* global mRNA expression reconstructed in the five-dimensional common HO GSVD subspace with genes sorted according to their phases in the two-dimensional subspace that approximates it (Supplementary Sections 2.3 and 2.4). (*a*) Expression of the sorted 3167 genes in the 17 arrays, centered at their gene- and array-invariant levels, showing a traveling wave of expression. (*b*) Expression of the sorted genes in the 17 arraylets, centered at their arraylet-invariant levels. Arraylets $k = 13, \ldots, 17$ display the sorting. (*c*) Line-joined graphs of the 13th (red), 14th (blue), 15th (green), 16th (orange) and 17th (violet) arraylets fit one-period cosines with initial phases similar to those of the corresponding genelets (Figure 2).

With all 3167 *S. pombe*, 4772 *S. cerevisiae* and 13,068 human genes sorted, the expression variations of the five $k = 13, \ldots, 17$ arraylets from each organism approximately fit one period cosines, with the initial phase of each arraylet similar to that of its corresponding genelet. The global mRNA expression of each organism, reconstructed in the common HO GSVD subspace, approximately fits a traveling wave, oscillating across time and across the genes (Supplementary Figures S5–S7).

2.5. Sequence-Independence of the Classification Our new HO GSVD provides a comparative mathematical framework for $N \geq 2$ large-scale DNA microarray datasets from N organisms tabulated as N matrices that does not require a one-to-one mapping between the genes of the different organisms. The HO GSVD, therefore, can be used to identify genes of common function across different organisms independently of the sequence similarity among these genes, and to study, e.g., nonorthologous gene displacement [3]. The HO GSVD can also be used to identify homologous genes, of similar DNA or protein sequences in one organism or across multiple organisms, that have different cellular functions.

We examine, for example, the HO GSVD classifications of genes of significantly different cell-cycle peak times [19] but highly conserved sequences [20, 21]. We consider three subsets of genes, the closest *S. pombe*, *S. cerevisiae* and human homologs of (*i*) the *S. pombe* gene *BFR1*, which belongs to the evolutionarily highly conserved ATP-binding cassette (ABC) transporter superfamily [22–28], (*ii*) the *S. cerevisiae* phospholipase B-encoding gene *PLB1* [29, 30], and (*iii*) the *S. pombe* strongly regulated S-phase cyclin-encoding gene *CIG2* [31, 32] (Supplementary Table S1). We find, notably, that these genes are correctly classified (Figure 4).

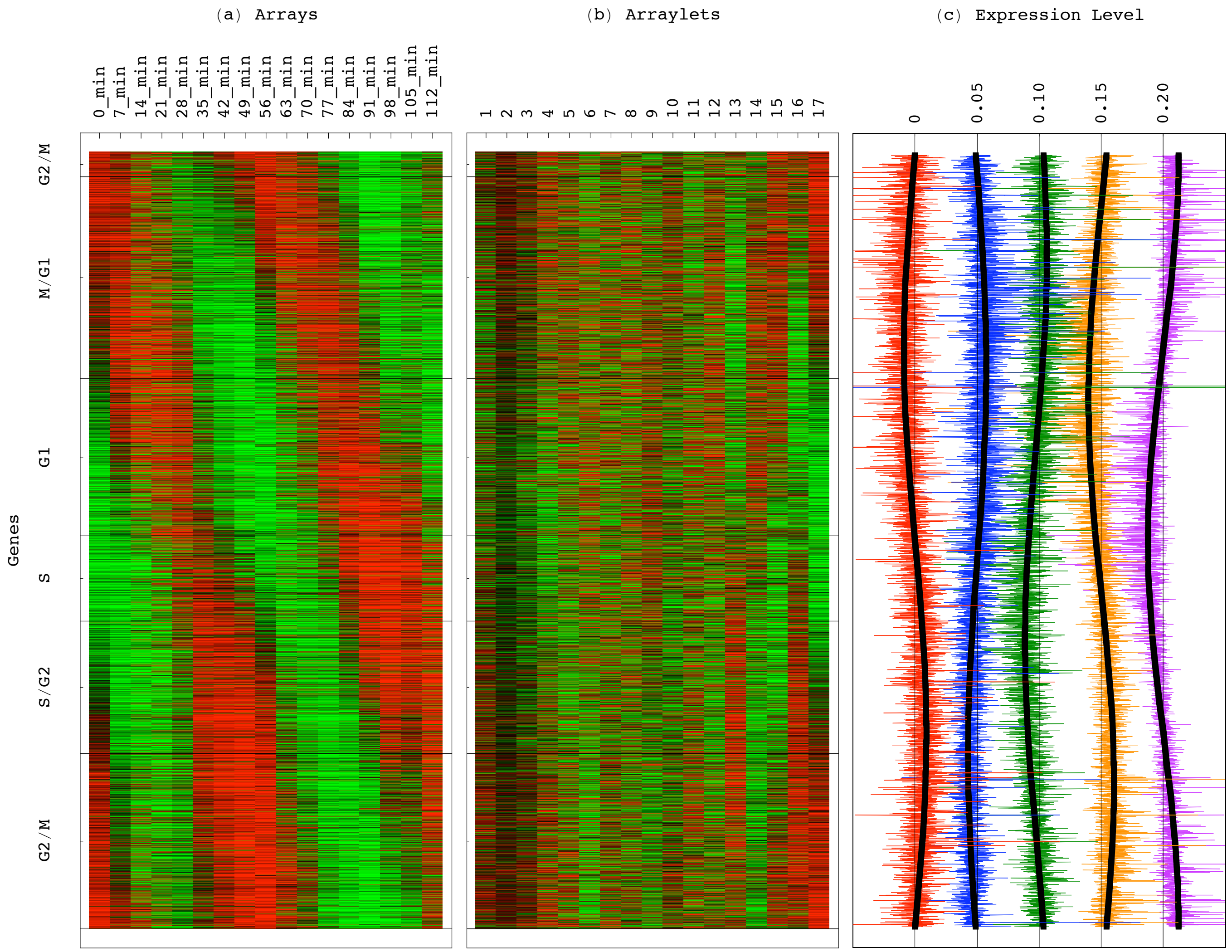

Supplementary Figure S6. *S. cerevisiae* global mRNA expression reconstructed in the five-dimensional common HO GSVD subspace with genes sorted according to their phases in the two-dimensional subspace that approximates it. (*a*) Expression of the sorted 4772 genes in the 17 arrays, centered at their gene- and array-invariant levels, showing a traveling wave of expression. (*b*) Expression of the sorted genes in the 17 arraylets, centered at their arraylet-invariant levels. Arraylets $k = 13, \ldots, 17$ display the sorting. (*c*) Line-joined graphs of the 13th (red), 14th (blue), 15th (green), 16th (orange) and 17th (violet) arraylets fit one-period cosines with initial phases similar to those of the corresponding genelets (Figure 2).

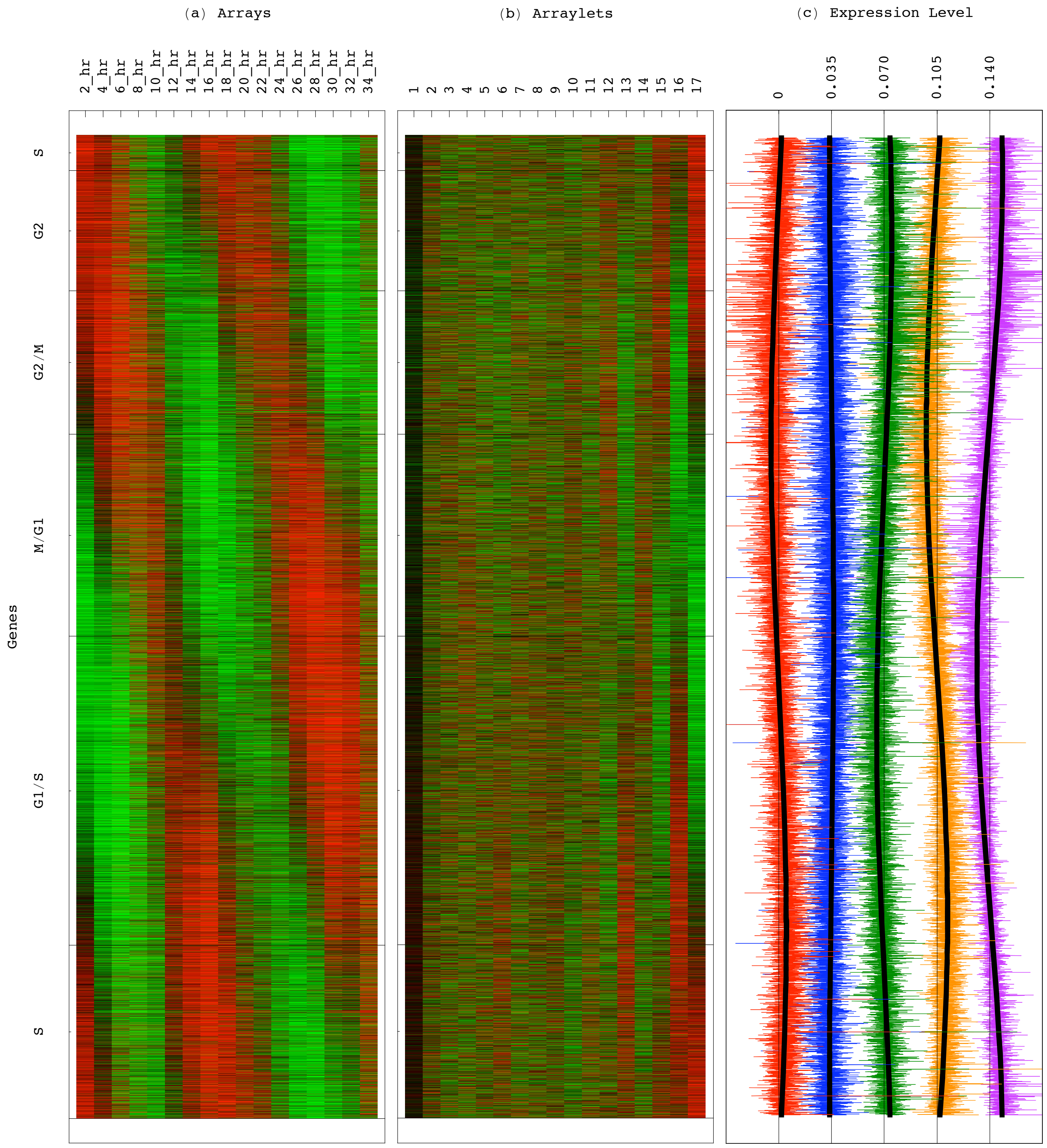

Supplementary Figure S7. Human global mRNA expression reconstructed in the five-dimensional common HO GSVD subspace with genes sorted according to their phases in the two-dimensional subspace that approximates it. (*a*) Expression of the sorted 13,068 genes in the 17 arrays, centered at their gene- and array-invariant levels, showing a traveling wave of expression. (*b*) Expression of the sorted genes in the 17 arraylets, centered at their arraylet-invariant levels. Arraylets $k = 13, \ldots, 17$ display the sorting. (*c*) Line-joined graphs of the 13th (red), 14th (blue), 15th (green), 16th (orange) and 17th (violet) arraylets fit one-period cosines with initial phases similar to those of the corresponding genelets.

	Query Gene	Gene	Organism	RefSeq ID	Bit Score	E-value
(a)	Bfr1	Snq2	*S. cerevisiae*	NP_010294.1	1149	0
	S. pombe	Pdr5	*S. cerevisiae*	NP_014796.1	1103	0
	NP_587932.3	Pdr18	*S. cerevisiae*	NP_014468.1	1097	0
		Pdr15	*S. cerevisiae*	NP_010694.1	1093	0
		Pdr12	*S. cerevisiae*	NP_015267.1	1070	0
		Pdr10	*S. cerevisiae*	NP_014973.1	1029	0
(b)	Plb1	Plb2	*S. cerevisiae*	NP_013719.1	825	0
	S. cerevisiae	Plb3	*S. cerevisiae*	NP_014632.1	813	0
	NP_013721.1	SPAC977.09c	*S. pombe*	NP_592772.1	385	7×10^{-107}
		SPAC1A6.03c	*S. pombe*	NP_593194.1	372	7×10^{-103}
		SPCC1450.09c	*S. pombe*	NP_588308.1	369	8×10^{-102}
		SPAC1786.02	*S. pombe*	NP_594024.1	355	1×10^{-97}
(c)	Cig2	Cdc13	*S. pombe*	NP_595171.1	346	3×10^{-95}
	S. pombe	Clb2	*S. cerevisiae*	NP_015444.1	248	1×10^{-65}
	NP_593889.1	Cig1	*S. pombe*	NP_588110.2	241	1×10^{-63}
		Clb1	*S. cerevisiae*	NP_011622.1	234	2×10^{-61}
		Clb4	*S. cerevisiae*	NP_013311.1	222	5×10^{-58}
		Clb3	*S. cerevisiae*	NP_010126.1	221	2×10^{-57}
		Ccnb2	Human	NP_004692.1	202	5×10^{-52}
		Clb6	*S. cerevisiae*	NP_011623.1	183	4×10^{-46}
		Clb5	*S. cerevisiae*	NP_015445.1	179	5×10^{-45}
		Ccnb1	Human	NP_114172.1	174	1×10^{-43}
		Rem1	*S. pombe*	NP_595798.1	160	2×10^{-39}
		Ccna1 (isoform c)	Human	NP_001104516.1	159	7×10^{-39}
		Ccna1 (isoform a)	Human	NP_003905.1	158	1×10^{-38}
		Ccna1 (isoform b)	Human	NP_001104515.1	157	1×10^{-38}
		Ccna2	Human	NP_001228.1	145	6×10^{-35}

Supplementary Table S1. The closest *S. pombe*, *S. cerevisiae* and human homologs of (*a*) the *S. pombe* gene *BFR1*, (*b*) the *S. cerevisiae* gene *PLB1*, and (*c*) the *S. pombe* gene *CIG2*, as determined by an NCBI BLAST [20] of the protein sequence that corresponds to each gene against the NCBI RefSeq database [21].
