# Supplementary material for: A Higher-Order Generalized Singular Value Decomposition for Comparison of Global mRNA Expression from Multiple Organisms
Source: PLoS One. 2011 Dec 22;6(12):e28072. doi: 10.1371/journal.pone.0028072 (PMC3245232; doi:10.1371/journal.pone.0028072)

(* © Sri Priya Ponnapalli and Orly Alter 2011 *)

(* All Rights Reserved *)

```
<< LinearAlgebra`MatrixManipulation`;
<< NumericalMath`TrigFit`;
<< Graphics`Graphics`;
<< Graphics`Colors`;
<< Graphics`Arrow`;
Off[General::"spell"];
Off[General::"spell1"];
```

(* Estimate Missing S. pombe, S. cerevisiae and Human Cell Cycle Data Using SVD *)

(* Estimate Missing S. pombe Cell Cycle Data Using SVD *)

```
(* Read S. pombe Data *)

stream = "Desktop/HO_GSVD/Data/S_pombe.txt";
matrix = Import[stream, "Table"];
{genes, arrays} = Dimensions[matrix] - {1, 4}
Clear[stream];

{3167, 17}

genenames = TakeRows[
   TakeColumns[matrix, {1, 4}],
   {2, genes + 1}];
arraynames = TakeColumns[
   TakeRows[matrix, {1, 1}],
   {5, arrays + 4}];
matrix = TakeColumns[
   TakeRows[matrix, {2, genes + 1}],
   {5, arrays + 4}];
matrix = ToExpression[matrix];

(* Count Null Data *)

counter = Table[Dimensions[Position[matrix[[a]], Null]][[1]], {a, 1, genes}];

(* Locate Gene Position of Null Data *)

Clear[positions];
positions = Table[0, {a, 1, arrays + 1}];
Do[
  positions[[a]] = Flatten[
    Position[Flatten[counter], a - 1]],
  {a, 1, arrays + 1}];
numbers = Flatten[
   Table[
    Dimensions[positions[[a]]],
    {a, 1, Round[arrays * 0.25]}]];
```

(* Create Display Of Gene Position Of Null Data *)

```
framex = Table[{a, a - 1}, {a, 1, Round[arrays * 0.25]}];
framey = {500, 1000, 1500, 2000};
labelx = ColumnForm[{"Number of Arrays"}, Center];
labely = ColumnForm[{"Number of Genes"}, Center];
g = BarChart[numbers,
   Frame -> True,
   Axes -> False,
   FrameLabel -> {labelx, labely, None, None},
   FrameTicks -> {framex, framey, None, None},
   GridLines -> {None, None},
   PlotRange -> {{0.5, Round[arrays * 0.25] + 0.5}, {0, 2000}},
   DisplayFunction -> Identity];
g = FullGraphics[g];
g[[1, 2]] = g[[1, 2]] /.
   Text[labely, {b_, c_}, {1., 0.}] ->
    Text[labely, {b - 0.75, c}, {0, 0}, {0, 1}];
g[[1, 2]] = g[[1, 2]] /.
   Text[labelx, {b_, c_}, {0., 1.}] ->
    Text[labelx, {b, c - 400}, {0, 1}, {1, 0}];
```

(* Display Gene Position Of Null Data *)

```
Show[g, PlotRange -> All];
```

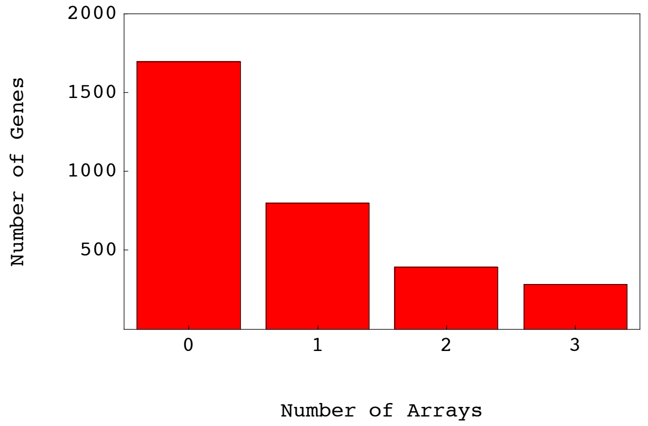

(* Select Genes by Number of Missing Data Points *)

```
matrix = AppendRows[Table[{counter[[a]]}, {a, 1, genes}], genenames, matrix];
matrix = Sort[matrix, OrderedQ[{#1, #2}] &];
fullgenenames = TakeColumns[
   TakeRows[matrix, {1, numbers[[1]]}],
   {2, 5}];
fullmatrix = TakeColumns[
   TakeRows[matrix, {1, numbers[[1]]}],
   {6, arrays + 5}];
missinggenenames1 = TakeColumns[
   TakeRows[matrix, {numbers[[1]] + 1, numbers[[1]] + numbers[[2]]}],
   {2, 5}];
missingmatrix1 = TakeColumns[
   TakeRows[matrix, {numbers[[1]] + 1, numbers[[1]] + numbers[[2]]}],
   {6, arrays + 5}];
missinggenenames2 = TakeColumns[
   TakeRows[matrix,
    {numbers[[1]] + numbers[[2]] + 1,
     numbers[[1]] + numbers[[2]] + numbers[[3]]}],
   {2, 5}];
missingmatrix2 = TakeColumns[
   TakeRows[matrix,
    {numbers[[1]] + numbers[[2]] + 1,
     numbers[[1]] + numbers[[2]] + numbers[[3]]}],
   {6, arrays + 5}];
```

```
missinggenenames3 = TakeColumns[
   TakeRows[matrix,
    {numbers[[1]] + numbers[[2]] + numbers[[3]] + 1,
     numbers[[1]] + numbers[[2]] + numbers[[3]] + numbers[[4]]}],
   {2, 5}];
missingmatrix3 = TakeColumns[
   TakeRows[matrix,
    {numbers[[1]] + numbers[[2]] + numbers[[3]] + 1,
     numbers[[1]] + numbers[[2]] + numbers[[3]] + numbers[[4]]}],
   {6, arrays + 5}];

(* Locate Array Position of Null Data *)

locator1 = Table[0, {numbers[[2]]}];
Do[
  locator1[[a]] = locator1[[a]] + Flatten[Position[missingmatrix1[[a]], Null]],
  {a, 1, numbers[[2]]}];
locator2 = Table[0, {numbers[[3]]}];
Do[
  locator2[[a]] = locator2[[a]] + Flatten[Position[missingmatrix2[[a]], Null]],
  {a, 1, numbers[[3]]}];
locator3 = Table[0, {numbers[[4]]}];
Do[
  locator3[[a]] = locator3[[a]] + Flatten[Position[missingmatrix3[[a]], Null]],
  {a, 1, numbers[[4]]}];

(* Sort Raw Data According to the Position of Missing Data Points for Each Gene *)

missingmatrix1 = AppendRows[locator1, missinggenenames1, missingmatrix1];
missingmatrix1 = Sort[missingmatrix1, OrderedQ[{#1, #2}] &];
locator1 = TakeColumns[missingmatrix1, {1, 1}];
missinggenenames1 = TakeColumns[missingmatrix1, {2, 5}];
missingmatrix1 = TakeColumns[missingmatrix1, {6, arrays + 5}];
missingmatrix2 = AppendRows[locator2, missinggenenames2, missingmatrix2];
missingmatrix2 = Sort[missingmatrix2, OrderedQ[{#1, #2}] &];
locator2 = TakeColumns[missingmatrix2, {1, 2}];
missinggenenames2 = TakeColumns[missingmatrix2, {3, 6}];
missingmatrix2 = TakeColumns[missingmatrix2, {7, arrays + 6}];
missingmatrix3 = AppendRows[locator3, missinggenenames3, missingmatrix3];
missingmatrix3 = Sort[missingmatrix3, OrderedQ[{#1, #2}] &];
locator3 = TakeColumns[missingmatrix3, {1, 3}];
missinggenenames3 = TakeColumns[missingmatrix3, {4, 7}];
missingmatrix3 = TakeColumns[missingmatrix3, {8, arrays + 7}];

(* Examine Subset of Genes with Full Data *)

(* Calculate SVD *)

{eigenarrays, eigenexpressions, eigengenes} = SingularValues[fullmatrix];
eigenarrays = Transpose[eigenarrays];
fractions = eigenexpressions^2 / Sum[eigenexpressions[[a]]^2, {a, 1, arrays}];
entropy = -N[Sum[fractions[[a]] * Log[fractions[[a]]], {a, 1, arrays}] / Log[arrays]];
entropy = N[Round[100 * entropy] / 100]

0.09

(* Create Fractions Bar Charts Displays *)

fractions[[2]]

0.0170902
```

```
limit = 0.018;
Clear[gridx, framex, framey];
gridx = Table[a, {a, 0, limit, N[limit / 3]}];
framex = gridx;
framex = Table[{gridx[[a]], framex[[a]]}, {a, 1, 4}];
gridx = Table[{gridx[[a]], RGBColor[0, 0, 0]}, {a, 1, 4}];
framey = Table[{a + 1, arrays - a - 6}, {a, 0, 12 - 3}];
table = Table[fractions[[arrays - a]], {a, 6, arrays - 2}];
g = BarChart[table,
   BarOrientation -> Horizontal,
   PlotRange -> {{0, limit * 1.0001}, {0.5, 12 - 2 + 0.5}},
   AspectRatio -> 1,
   Axes -> False,
   Frame -> True,
   FrameTicks -> {None, framey, framex, None},
   FrameLabel -> {None, None, None, None},
   GridLines -> {gridx, None},
   DisplayFunction -> Identity];
g = FullGraphics[g];
g[[1, 2]] = g[[1, 2]] /.
   Text[a_, {b_, c_}, {0., -1.}] ->
    Text[a, {b, c}, {0, -1}, {0, 1}];
g1 = Show[g,
   AspectRatio -> 1.25,
   PlotRange -> All,
   DisplayFunction -> Identity];

gridx = Table[a, {a, 0, 1, 0.2}];
framex = gridx;
framex = Table[{gridx[[a]], framex[[a]]}, {a, 1, 6}];
framex[[6]] = {1, "1"};
gridx = Table[{gridx[[a]], RGBColor[0, 0, 0]}, {a, 1, 6}];
framey = Table[{a + 1, arrays - a}, {a, 0, arrays - 1}];
labelx = ColumnForm[
   {"(b) Eigenexpression Fraction", StringJoin["d'1 = ", ToString[entropy]], " "},
   Center];
g = BarChart[
   Table[fractions[[arrays - a]], {a, 0, arrays - 1}],
   BarOrientation -> Horizontal,
   PlotRange -> {{0, 1.0001}, {0.5, arrays + 0.5}},
   AspectRatio -> 1,
   Axes -> False,
   Frame -> True,
   FrameTicks -> {None, framey, framex, None},
   FrameLabel -> {None, None, labelx, None},
   GridLines -> {gridx, None},
   DisplayFunction -> Identity];
g = FullGraphics[g];
g[[1, 2]] = g[[1, 2]] /.
   Text[labelx, {b_, c_}, {0., -1.}] →
    Text[labelx, {b, c + 3}, {0, -1}, {1, 0}];
g[[1, 2]] = g[[1, 2]] /.
   Text[a_, {b_, c_}, {0., -1.}] →
    Text[a, {b, c}, {0, -1}, {0, 1}];
g2 = Show[{g,
    Graphics[{RGBColor[1, 1, 0.8], Rectangle[{0.1, 0.6}, {0.98, 15.5}]}],
    Graphics[{Rectangle[{0.1, 0.6}, {0.98, 15.5}, g1]}]},
   AspectRatio -> 1.35,
   PlotRange -> All,
   DisplayFunction -> Identity];
```

```
(* Create Eigengenes 2D Red & Green Raster Display *)

contrast = 3.5;
displaying = Table[
   If[contrast * eigengenes[[i, j]] > 0,
    If[contrast * eigengenes[[i, j]] < 1, {contrast * eigengenes[[i, j]], 0}, {1, 0}],
    If[contrast * eigengenes[[i, j]] > -1, {0, -contrast * eigengenes[[i, j]]}, {0, 1}]],
   {i, 1, arrays}, {j, 1, arrays}];
framex = Table[{a - 0.5, arraynames[[1, a]]}, {a, 1, arrays}];
framey = Table[{a + 1 - 0.5, arrays - a}, {a, 0, arrays - 1}];
labely = "Eigengenes";
labelx = ColumnForm[{"(a) Arrays", " ", " "}, Center];

g = Show[
   Graphics[
    RasterArray[
     Table[
      RGBColor[displaying[[i, j, 1]], displaying[[i, j, 2]], 0],
      {i, arrays, 1, -1}, {j, 1, arrays}]]],
   AspectRatio -> 1,
   Frame -> True,
   FrameTicks -> {None, framey, framex, None},
   FrameLabel -> {None, labely, labelx, None},
   DisplayFunction -> Identity];
g = FullGraphics[g];
g[[1, 2]] = g[[1, 2]] /.
   Text[labely, {b_, c_}, {1., 0.}] ->
    Text[labely, {b - 3, c}, {0, 0}, {0, 1}];
g[[1, 2]] = g[[1, 2]] /.
   Text[labelx, {b_, c_}, {0., -1.}] ->
    Text[labelx, {b, c + 3}, {0, -1}, {1, 0}];
g[[1, 2]] = g[[1, 2]] /.
   Text[a_, {b_, c_}, {0., -1.}] ->
    Text[a, {b, c}, {0, -1}, {0, 1}];
g1 = Show[g,
   AspectRatio -> 1.05,
   PlotRange -> All,
   DisplayFunction -> Identity];

(* Create Selected Eigengenes Graph Display *)

p = Table[0, {5}];
color = {RGBColor[1, 0, 0], RGBColor[0, 0, 1], RGBColor[0, 0.5, 0],
   RGBColor[1, 0.5, 0], RGBColor[0.75, 0, 1]};
xlabels = {"(c) Arrays", " ", " ", "(d) Arrays", " "};
ylabels = {{" ", "Expression Level"}, {" ", " "}, {" ", " "}, {" ", " "}, {" ", " "}};
framex = Table[{a - 1, arraynames[[1, a]]}, {a, 1, arrays}];
framey = {-1, -0.5, 0, 0.5, 1};
flag = 0;
```

```
Do[{
  labelx = ColumnForm[{xlabels[[n]]}, Center],
  labely = ColumnForm[ylabels[[n]], Center],
  coordinates = Table[{a - 1, eigengenes[[n, a]]}, {a, 1, arrays}],
  points = Table[Point[coordinates[[a]]], {a, 1, arrays}],
  line = Line[coordinates],
  If[n == 4, flag = 0],
  g = Show[{
     Graphics[{color[[n]], PointSize[0.022], points}],
     Graphics[{color[[n]], line}]},
    If[flag == 0,
     {FrameLabel -> {None, labely, labelx, None},
      FrameTicks -> {None, framey, framex, None}},
     {FrameLabel -> {None, labely, labelx, None},
      FrameTicks -> {None, None, None, None}}],
    Frame -> True,
    GridLines -> {None, {{0, RGBColor[0, 0, 0]}}},
    PlotRange -> {-1.05, 1.05},
    DisplayFunction -> Identity],
  g = FullGraphics[g],
  g[[1, 2]] = g[[1, 2]] /.
    Text[labely, {b_, c_}, {1., 0.}] ->
     Text[labely, {b - 5.4, c}, {0, 0}, {0, 1}],
  g[[1, 2]] = g[[1, 2]] /.
    Text[labelx, {b_, c_}, {0., -1.}] ->
     Text[labelx, {b, c + 0.625}, {0, -1}, {1, 0}],
  g[[1, 2]] = g[[1, 2]] /.
    Text[a_, {b_, c_}, {0., -1.}] ->
     Text[a, {b, c}, {0, -1}, {0, 1}],
  flag = flag + 1,
  p[[n]] = Show[g,
    AspectRatio -> 1.05,
    PlotRange -> All,
    DisplayFunction -> Identity]},
 {n, 1, 5}]

(* Display Selected Eigengenes *)

g3 = Show[{p[[1]], p[[2]], p[[3]]}, DisplayFunction -> Identity];
g4 = Show[{p[[4]], p[[5]]}, DisplayFunction -> Identity];

(* Display Eigengenes, Fractions and Selected Eigengenes *)

Show[GraphicsArray[{g1, g2, g3, g4}],
  GraphicsSpacing -> -0.15];
```

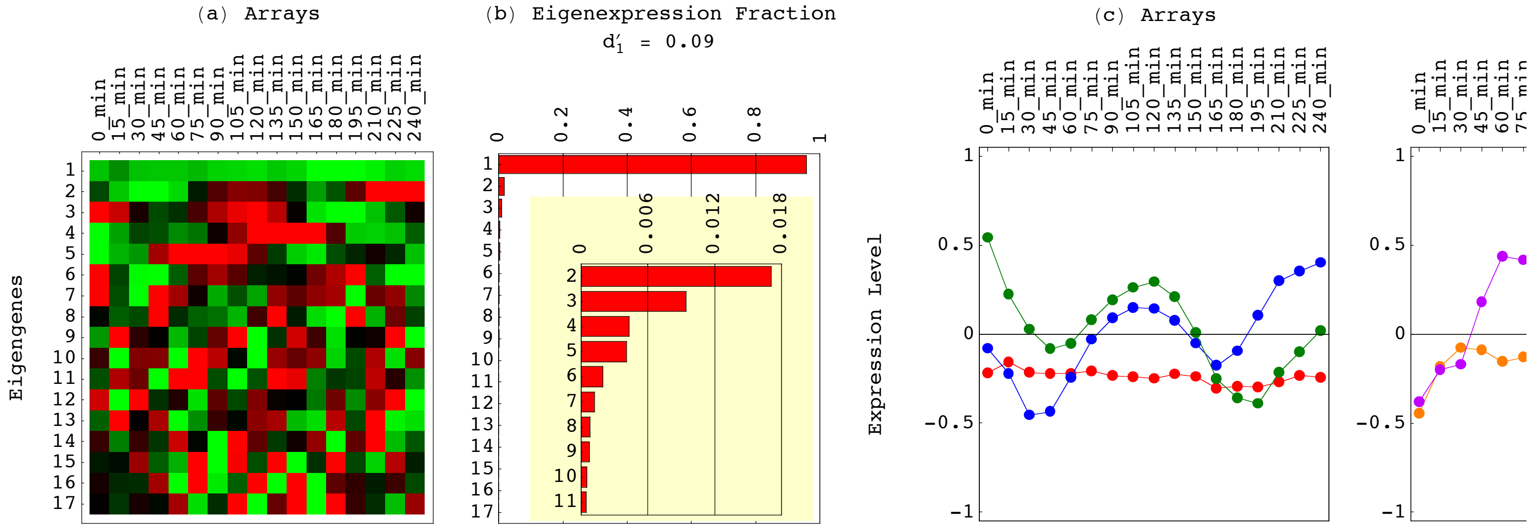

```
(* Choose Subset of Eigengenes for Estimation *)

eigengenes = TakeRows[eigengenes, {1, 5}];

(* Estimate Missing Data *)

Do[
 missingmatrix1[[a, locator1[[a, 1]]]] =
  N[Round[Flatten[Dot[Dot[
          Transpose[Drop[
            Transpose[{missingmatrix1[[a]]}],
            {locator1[[a, 1]]}]],
          PseudoInverse[Transpose[Drop[
             Transpose[eigengenes],
             {locator1[[a, 1]]}]]]],
         eigengenes]][[locator1[[a, 1]]]] * 100] / 100],
 {a, 1, numbers[[2]]}]

Do[Do[
  missingmatrix2[[a, locator2[[a, b]]]] =
   N[Round[Flatten[Dot[Dot[
           Transpose[Drop[Drop[
              Transpose[{missingmatrix2[[a]]}],
              {locator2[[a, 2]]}], {locator2[[a, 1]]}]],
           PseudoInverse[Transpose[Drop[Drop[
               Transpose[eigengenes],
               {locator2[[a, 2]]}], {locator2[[a, 1]]}]]]],
          eigengenes]][[locator2[[a, b]]]] * 100] / 100],
  {b, 1, 2}],
 {a, 1, numbers[[3]]}]

Do[Do[
  missingmatrix3[[a, locator3[[a, b]]]] =
   N[Round[Flatten[Dot[Dot[
           Transpose[Drop[Drop[Drop[
               Transpose[{missingmatrix3[[a]]}],
               {locator3[[a, 3]]}], {locator3[[a, 2]]}], {locator3[[a, 1]]}]],
           PseudoInverse[Transpose[Drop[Drop[Drop[
                Transpose[eigengenes],
                {locator3[[a, 3]]}], {locator3[[a, 2]]}], {locator3[[a, 1]]}]]]],
          eigengenes]][[locator3[[a, b]]]] * 100] / 100],
  {b, 1, 3}],
 {a, 1, numbers[[4]]}]

genenames = AppendColumns[
   fullgenenames,
   missinggenenames1,
   missinggenenames2,
   missinggenenames3];
matrix = AppendColumns[
   fullmatrix,
   missingmatrix1,
   missingmatrix2,
   missingmatrix3];
{genes, arrays} = Dimensions[matrix];
matrix1 = matrix;
genenames1 = genenames;
arraynames1 = arraynames;
{genes1, arrays1} = Dimensions[matrix1]

{3167, 17}
```

```
(* Examine S. pombe Data After Missing Data Estimation *)

(* Calculate SVD *)

{eigenarrays, eigenexpressions, eigengenes} = SingularValues[matrix];
eigenarrays = Transpose[eigenarrays];
fractions = eigenexpressions^2 / Sum[eigenexpressions[[a]]^2, {a, 1, arrays}];
entropy = -N[Sum[fractions[[a]] * Log[fractions[[a]]], {a, 1, arrays}] / Log[arrays]];
entropy = N[Round[100 * entropy] / 100]
```

0.08

```
(* Create Fractions Bar Charts Displays *)

fractions[[2]]
```

0.015115

```
limit = 0.018;
Clear[gridx, framex, framey];
gridx = Table[a, {a, 0, limit, N[limit / 3]}];
framex = gridx;
framex = Table[{gridx[[a]], framex[[a]]}, {a, 1, 4}];
gridx = Table[{gridx[[a]], RGBColor[0, 0, 0]}, {a, 1, 4}];
framey = Table[{a + 1, arrays - a - 6}, {a, 0, 12 - 3}];
table = Table[fractions[[arrays - a]], {a, 6, arrays - 2}];
g = BarChart[table,
   BarOrientation -> Horizontal,
   PlotRange -> {{0, limit * 1.0001}, {0.5, 12 - 2 + 0.5}},
   AspectRatio -> 1,
   Axes -> False,
   Frame -> True,
   FrameTicks -> {None, framey, framex, None},
   FrameLabel -> {None, None, None, None},
   GridLines -> {gridx, None},
   DisplayFunction -> Identity];
g = FullGraphics[g];
g[[1, 2]] = g[[1, 2]] /.
   Text[a_, {b_, c_}, {0., -1.}] ->
    Text[a, {b, c}, {0, -1}, {0, 1}];
g1 = Show[g,
   AspectRatio -> 1.25,
   PlotRange -> All,
   DisplayFunction -> Identity];

gridx = Table[a, {a, 0, 1, 0.2}];
framex = gridx;
framex = Table[{gridx[[a]], framex[[a]]}, {a, 1, 6}];
framex[[6]] = {1, "1"};
gridx = Table[{gridx[[a]], RGBColor[0, 0, 0]}, {a, 1, 6}];
framey = Table[{a + 1, arrays - a}, {a, 0, arrays - 1}];
labelx = ColumnForm[
   {"(b) Eigenexpression Fraction", StringJoin["d₁ = ", ToString[entropy]], " "},
   Center];
g = BarChart[
   Table[fractions[[arrays - a]], {a, 0, arrays - 1}],
   BarOrientation -> Horizontal,
   PlotRange -> {{0, 1.0001}, {0.5, arrays + 0.5}},
   AspectRatio -> 1,
   Axes -> False,
   Frame -> True,
   FrameTicks -> {None, framey, framex, None},
   FrameLabel -> {None, None, labelx, None},
   GridLines -> {gridx, None},
   DisplayFunction -> Identity];
```

```
g = FullGraphics[g];
g[[1, 2]] = g[[1, 2]] /.
   Text[labelx, {b_, c_}, {0., -1.}] ->
    Text[labelx, {b, c + 3}, {0, -1}, {1, 0}];
g[[1, 2]] = g[[1, 2]] /.
   Text[a_, {b_, c_}, {0., -1.}] ->
    Text[a, {b, c}, {0, -1}, {0, 1}];
g2 = Show[{g,
    Graphics[{RGBColor[1, 1, 0.8], Rectangle[{0.1, 0.6}, {0.98, 15.5}]}],
    Graphics[{Rectangle[{0.1, 0.6}, {0.98, 15.5}, g1]}]},
   AspectRatio -> 1.35,
   PlotRange -> All,
   DisplayFunction -> Identity];

(* Create Eigengenes 2 D Red & Green Raster Display *)

contrast = 3.5;
displaying = Table[
   If[contrast * eigengenes[[i, j]] > 0,
    If[contrast * eigengenes[[i, j]] < 1, {contrast * eigengenes[[i, j]], 0}, {1, 0}],
    If[contrast * eigengenes[[i, j]] > -1, {0, -contrast * eigengenes[[i, j]]}, {0, 1}]],
   {i, 1, arrays}, {j, 1, arrays}];
framex = Table[{a - 0.5, arraynames[[1, a]]}, {a, 1, arrays}];
framey = Table[{a + 1 - 0.5, arrays - a}, {a, 0, arrays - 1}];
labely = "Eigengenes";
labelx = ColumnForm[{"(a) Arrays", " ", " "}, Center];

g = Show[
   Graphics[
    RasterArray[
     Table[
      RGBColor[displaying[[i, j, 1]], displaying[[i, j, 2]], 0],
      {i, arrays, 1, -1}, {j, 1, arrays}]]],
   AspectRatio -> 1,
   Frame -> True,
   FrameTicks -> {None, framey, framex, None},
   FrameLabel -> {None, labely, labelx, None},
   DisplayFunction -> Identity];
g = FullGraphics[g];
g[[1, 2]] = g[[1, 2]] /.
   Text[labely, {b_, c_}, {1., 0.}] ->
    Text[labely, {b - 3, c}, {0, 0}, {0, 1}];
g[[1, 2]] = g[[1, 2]] /.
   Text[labelx, {b_, c_}, {0., -1.}] ->
    Text[labelx, {b, c + 3}, {0, -1}, {1, 0}];
g[[1, 2]] = g[[1, 2]] /.
   Text[a_, {b_, c_}, {0., -1.}] ->
    Text[a, {b, c}, {0, -1}, {0, 1}];
g1 = Show[g,
   AspectRatio -> 1.05,
   PlotRange -> All,
   DisplayFunction -> Identity];

(* Create Selected Eigengenes Graph Display *)

p = Table[0, {5}];
color = {RGBColor[1, 0, 0], RGBColor[0, 0, 1], RGBColor[0, 0.5, 0],
   RGBColor[1, 0.5, 0], RGBColor[0.75, 0, 1]};
xlabels = {"(c) Arrays", "", "", "(d) Arrays", ""};
ylabels = {{" ", "Expression Level"}, {" ", " "}, {" ", " "}, {" ", " "}, {" ", " "}};
framex = Table[{a - 1, arraynames[[1, a]]}, {a, 1, arrays}];
framey = {-1, -0.5, 0, 0.5, 1};
flag = 0;
```

```
Do[{
  labelx = ColumnForm[{xlabels[[n]]}, Center],
  labely = ColumnForm[ylabels[[n]], Center],
  coordinates = Table[{a - 1, eigengenes[[n, a]]}, {a, 1, arrays}],
  points = Table[Point[coordinates[[a]]], {a, 1, arrays}],
  line = Line[coordinates],
  If[n == 4, flag = 0],
  g = Show[{
     Graphics[{color[[n]], PointSize[0.022], points}],
     Graphics[{color[[n]], line}]},
    If[flag == 0,
     {FrameLabel -> {None, labely, labelx, None},
      FrameTicks -> {None, framey, framex, None}},
     {FrameLabel -> {None, labely, labelx, None},
      FrameTicks -> {None, None, None, None}}],
    Frame -> True,
    GridLines -> {None, {{0, RGBColor[0, 0, 0]}}},
    PlotRange -> {-1.05, 1.05},
    DisplayFunction -> Identity],
  g = FullGraphics[g],
  g[[1, 2]] = g[[1, 2]] /.
    Text[labely, {b_, c_}, {1., 0.}] ->
     Text[labely, {b - 5.4, c}, {0, 0}, {0, 1}],
  g[[1, 2]] = g[[1, 2]] /.
    Text[labelx, {b_, c_}, {0., -1.}] ->
     Text[labelx, {b, c + 0.625}, {0, -1}, {1, 0}],
  g[[1, 2]] = g[[1, 2]] /.
    Text[a_, {b_, c_}, {0., -1.}] ->
     Text[a, {b, c}, {0, -1}, {0, 1}],
  flag = flag + 1,
  p[[n]] = Show[g,
    AspectRatio -> 1.05,
    PlotRange -> All,
    DisplayFunction -> Identity]},
 {n, 1, 5}]

(* Display Selected Eigengenes *)

g3 = Show[{p[[1]], p[[2]], p[[3]]}, DisplayFunction -> Identity];
g4 = Show[{p[[4]], p[[5]]}, DisplayFunction -> Identity];

(* Display Eigengenes, Fractions and Selected Eigengenes *)

Show[GraphicsArray[{g1, g2, g3, g4}],
  GraphicsSpacing -> -0.15];
```

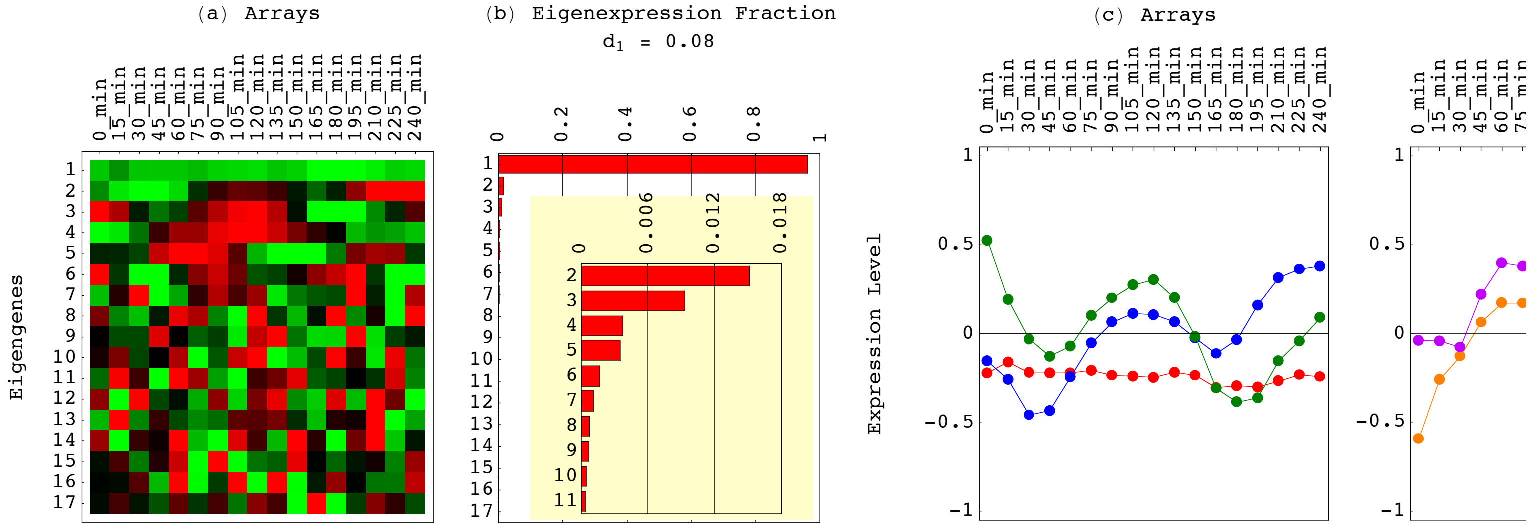

```
(* Estimate S. cerevisiae Missing Cell Cycle Data Using SVD *)

(* Read S. cerevisiae Data *)

stream = "Desktop/HO_GSVD/Data/S_cerevisiae.txt";
matrix = Import[stream, "Table"];
{genes, arrays} = Dimensions[matrix] - {1, 3}
Clear[stream];

{4772, 17}

genenames = TakeRows[
   TakeColumns[matrix, {1, 3}],
   {2, genes + 1}];
arraynames = TakeColumns[
   TakeRows[matrix, {1, 1}],
   {4, arrays + 3}];
matrix = TakeColumns[
   TakeRows[matrix, {2, genes + 1}],
   {4, arrays + 3}];
matrix = ToExpression[matrix];

(* Count Null Data *)

counter = Table[Dimensions[Position[matrix[[a]], Null]][[1]], {a, 1, genes}];

(* Locate Gene Position of Null Data *)

Clear[positions];
positions = Table[0, {a, 1, arrays + 1}];
Do[
  positions[[a]] = Flatten[
    Position[Flatten[counter], a - 1]],
  {a, 1, arrays + 1}];
numbers = Flatten[
   Table[
    Dimensions[positions[[a]]],
    {a, 1, Round[arrays * 0.25]}]];

(* Create Display Of Gene Position Of Null Data *)

framex = Table[{a, a - 1}, {a, 1, Round[arrays * 0.25]}];
framey = {500, 1000, 1500, 2000, 2500, 3000};
labelx = ColumnForm[{"Number of Arrays"}, Center];
labely = ColumnForm[{"Number of Genes"}, Center];
g = BarChart[numbers,
   Frame -> True,
   Axes -> False,
   FrameLabel -> {labelx, labely, None, None},
   FrameTicks -> {framex, framey, None, None},
   GridLines -> {None, None},
   PlotRange -> {{0.5, Round[arrays * 0.25] + 0.5}, {0, 3000}},
   DisplayFunction -> Identity];
g = FullGraphics[g];
g[[1, 2]] = g[[1, 2]] /.
   Text[labely, {b_, c_}, {1., 0.}] ->
    Text[labely, {b - 0.75, c}, {0, 0}, {0, 1}];
g[[1, 2]] = g[[1, 2]] /.
   Text[labelx, {b_, c_}, {0., 1.}] ->
    Text[labelx, {b, c - 400}, {0, 1}, {1, 0}];
```

```
(* Display Gene Position Of Null Data *)

Show[g, PlotRange -> All];
```

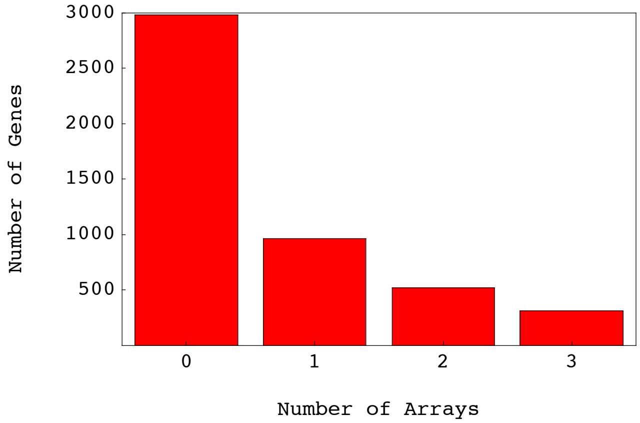


```
(* Select Genes by Number of Missing Data Points *)

matrix = AppendRows[Table[{counter[[a]]}, {a, 1, genes}], genenames, matrix];
matrix = Sort[matrix, OrderedQ[{#1, #2}] &];
fullgenenames = TakeColumns[
   TakeRows[matrix, {1, numbers[[1]]}],
   {2, 4}];
fullmatrix = TakeColumns[
   TakeRows[matrix, {1, numbers[[1]]}],
   {5, arrays + 4}];
missinggenenames1 = TakeColumns[
   TakeRows[matrix, {numbers[[1]] + 1, numbers[[1]] + numbers[[2]]}],
   {2, 4}];
missingmatrix1 = TakeColumns[
   TakeRows[matrix, {numbers[[1]] + 1, numbers[[1]] + numbers[[2]]}],
   {5, arrays + 4}];
missinggenenames2 = TakeColumns[
   TakeRows[matrix,
    {numbers[[1]] + numbers[[2]] + 1,
     numbers[[1]] + numbers[[2]] + numbers[[3]]}],
   {2, 4}];
missingmatrix2 = TakeColumns[
   TakeRows[matrix,
    {numbers[[1]] + numbers[[2]] + 1,
     numbers[[1]] + numbers[[2]] + numbers[[3]]}],
   {5, arrays + 4}];
missinggenenames3 = TakeColumns[
   TakeRows[matrix,
    {numbers[[1]] + numbers[[2]] + numbers[[3]] + 1,
     numbers[[1]] + numbers[[2]] + numbers[[3]] + numbers[[4]]}],
   {2, 4}];
missingmatrix3 = TakeColumns[
   TakeRows[matrix,
    {numbers[[1]] + numbers[[2]] + numbers[[3]] + 1,
     numbers[[1]] + numbers[[2]] + numbers[[3]] + numbers[[4]]}],
   {5, arrays + 4}];
```

```
(* Locate Array Position of Null Data *)

locator1 = Table[0, {numbers[[2]]}];
Do[
  locator1[[a]] = locator1[[a]] + Flatten[Position[missingmatrix1[[a]], Null]],
  {a, 1, numbers[[2]]}];
locator2 = Table[0, {numbers[[3]]}];
Do[
  locator2[[a]] = locator2[[a]] + Flatten[Position[missingmatrix2[[a]], Null]],
  {a, 1, numbers[[3]]}];
locator3 = Table[0, {numbers[[4]]}];
Do[
  locator3[[a]] = locator3[[a]] + Flatten[Position[missingmatrix3[[a]], Null]],
  {a, 1, numbers[[4]]}];

(* Sort Raw Data According to the Position of Missing Data Points for Each Gene *)

missingmatrix1 = AppendRows[locator1, missinggenenames1, missingmatrix1];
missingmatrix1 = Sort[missingmatrix1, OrderedQ[{#1, #2}] &];
locator1 = TakeColumns[missingmatrix1, {1, 1}];
missinggenenames1 = TakeColumns[missingmatrix1, {2, 4}];
missingmatrix1 = TakeColumns[missingmatrix1, {5, arrays + 4}];
missingmatrix2 = AppendRows[locator2, missinggenenames2, missingmatrix2];
missingmatrix2 = Sort[missingmatrix2, OrderedQ[{#1, #2}] &];
locator2 = TakeColumns[missingmatrix2, {1, 2}];
missinggenenames2 = TakeColumns[missingmatrix2, {3, 5}];
missingmatrix2 = TakeColumns[missingmatrix2, {6, arrays + 5}];
missingmatrix3 = AppendRows[locator3, missinggenenames3, missingmatrix3];
missingmatrix3 = Sort[missingmatrix3, OrderedQ[{#1, #2}] &];
locator3 = TakeColumns[missingmatrix3, {1, 3}];
missinggenenames3 = TakeColumns[missingmatrix3, {4, 6}];
missingmatrix3 = TakeColumns[missingmatrix3, {7, arrays + 6}];

(* Examine Subset of Genes with Full Data *)

(* Calculate SVD *)

{eigenarrays, eigenexpressions, eigengenes} = SingularValues[fullmatrix];
eigenarrays = Transpose[eigenarrays];
fractions = eigenexpressions^2 / Sum[eigenexpressions[[a]]^2, {a, 1, arrays}];
entropy = -N[Sum[fractions[[a]] * Log[fractions[[a]]], {a, 1, arrays}] / Log[arrays]];
entropy = N[Round[100 * entropy] / 100]

0.19

(* Create Fractions Bar Charts Displays *)

fractions[[3]]

0.0100742
```

```
limit = 0.012;
Clear[gridx, framex, framey];
gridx = Table[a, {a, 0, limit, N[limit / 3]}];
framex = gridx;
framex = Table[{gridx[[a]], framex[[a]]}, {a, 1, 4}];
gridx = Table[{gridx[[a]], RGBColor[0, 0, 0]}, {a, 1, 4}];
framey = Table[{a + 1, arrays - a - 6}, {a, 0, 12 - 3}];
table = Table[fractions[[arrays - a]], {a, 6, arrays - 3}];
g = BarChart[table,
   BarOrientation -> Horizontal,
   PlotRange -> {{0, limit * 1.0001}, {0.5, 11 - 2 + 0.5}},
   AspectRatio -> 1,
   Axes -> False,
   Frame -> True,
   FrameTicks -> {None, framey, framex, None},
   FrameLabel -> {None, None, None, None},
   GridLines -> {gridx, None},
   DisplayFunction -> Identity];
g = FullGraphics[g];
g[[1, 2]] = g[[1, 2]] /.
   Text[a_, {b_, c_}, {0., -1.}] ->
    Text[a, {b, c}, {0, -1}, {0, 1}];
g1 = Show[g,
   AspectRatio -> 1.25,
   PlotRange -> All,
   DisplayFunction -> Identity];

gridx = Table[a, {a, 0, 1, 0.2}];
framex = gridx;
framex = Table[{gridx[[a]], framex[[a]]}, {a, 1, 6}];
framex[[6]] = {1, "1"};
gridx = Table[{gridx[[a]], RGBColor[0, 0, 0]}, {a, 1, 6}];
framey = Table[{a + 1, arrays - a}, {a, 0, arrays - 1}];
labelx = ColumnForm[
   {"(b) Eigenexpression Fraction", StringJoin["d′2 = ", ToString[entropy]], " "},
   Center];
g = BarChart[
   Table[fractions[[arrays - a]], {a, 0, arrays - 1}],
   BarOrientation -> Horizontal,
   PlotRange -> {{0, 1.0001}, {0.5, arrays + 0.5}},
   AspectRatio -> 1,
   Axes -> False,
   Frame -> True,
   FrameTicks -> {None, framey, framex, None},
   FrameLabel -> {None, None, labelx, None},
   GridLines -> {gridx, None},
   DisplayFunction -> Identity];
g = FullGraphics[g];
g[[1, 2]] = g[[1, 2]] /.
   Text[labelx, {b_, c_}, {0., -1.}] ->
    Text[labelx, {b, c + 3}, {0, -1}, {1, 0}];
g[[1, 2]] = g[[1, 2]] /.
   Text[a_, {b_, c_}, {0., -1.}] ->
    Text[a, {b, c}, {0, -1}, {0, 1}];
g2 = Show[{g,
    Graphics[{RGBColor[1, 1, 0.8], Rectangle[{0.15, 0.6}, {0.98, 15.5}]}],
    Graphics[{Rectangle[{0.1, 0.6}, {0.98, 15.5}, g1]}]},
   AspectRatio -> 1.35,
   PlotRange -> All,
   DisplayFunction -> Identity];
```

```
(* Create Eigengenes 2 D Red & Green Raster Display *)

contrast = 3.5;
displaying = Table[
   If[contrast * eigengenes[[i, j]] > 0,
    If[contrast * eigengenes[[i, j]] < 1, {contrast * eigengenes[[i, j]], 0}, {1, 0}],
    If[contrast * eigengenes[[i, j]] > -1, {0, -contrast * eigengenes[[i, j]]}, {0, 1}]],
   {i, 1, arrays}, {j, 1, arrays}];
framex = Table[{a - 0.5, arraynames[[1, a]]}, {a, 1, arrays}];
framey = Table[{a + 1 - 0.5, arrays - a}, {a, 0, arrays - 1}];
labely = "Eigengenes";
labelx = ColumnForm[{"(a) Arrays", " ", " "}, Center];
g = Show[
   Graphics[
    RasterArray[
     Table[
      RGBColor[displaying[[i, j, 1]], displaying[[i, j, 2]], 0],
      {i, arrays, 1, -1}, {j, 1, arrays}]]],
   AspectRatio -> 1,
   Frame -> True,
   FrameTicks -> {None, framey, framex, None},
   FrameLabel -> {None, labely, labelx, None},
   DisplayFunction -> Identity];
g = FullGraphics[g];
g[[1, 2]] = g[[1, 2]] /.
   Text[labely, {b_, c_}, {1., 0.}] ->
    Text[labely, {b - 3, c}, {0, 0}, {0, 1}];
g[[1, 2]] = g[[1, 2]] /.
   Text[labelx, {b_, c_}, {0., -1.}] ->
    Text[labelx, {b, c + 3}, {0, -1}, {1, 0}];
g[[1, 2]] = g[[1, 2]] /.
   Text[a_, {b_, c_}, {0., -1.}] ->
    Text[a, {b, c}, {0, -1}, {0, 1}];
g1 = Show[g,
   AspectRatio -> 1.05,
   PlotRange -> All,
   DisplayFunction -> Identity];

(* Create Selected Eigengenes Graph Display *)

p = Table[0, {5}];
color = {RGBColor[1, 0, 0], RGBColor[0, 0, 1], RGBColor[0, 0.5, 0],
   RGBColor[1, 0.5, 0], RGBColor[0.75, 0, 1]};
xlabels = {"(c) Arrays", " ", " ", "(d) Arrays", " "};
ylabels = {{" ", "Expression Level"}, {" ", " "}, {" ", " "}, {" ", " "}, {" ", " "}};
framex = Table[{a - 1, arraynames[[1, a]]}, {a, 1, arrays}];
framey = {-1, -0.5, 0, 0.5, 1};
flag = 0;
```

```
Do[{
  labelx = ColumnForm[{xlabels[[n]]}, Center],
  labely = ColumnForm[ylabels[[n]], Center],
  coordinates = Table[{a - 1, eigengenes[[n, a]]}, {a, 1, arrays}],
  points = Table[Point[coordinates[[a]]], {a, 1, arrays}],
  line = Line[coordinates],
  If[n == 4, flag = 0],
  g = Show[{
     Graphics[{color[[n]], PointSize[0.022], points}],
     Graphics[{color[[n]], line}]},
    If[flag == 0,
     {FrameLabel -> {None, labely, labelx, None},
      FrameTicks -> {None, framey, framex, None}},
     {FrameLabel -> {None, labely, labelx, None},
      FrameTicks -> {None, None, None, None}}],
    Frame -> True,
    GridLines -> {None, {{0, RGBColor[0, 0, 0]}}},
    PlotRange -> {-1.05, 1.05},
    DisplayFunction -> Identity],
  g = FullGraphics[g],
  g[[1, 2]] = g[[1, 2]] /.
    Text[labely, {b_, c_}, {1., 0.}] ->
     Text[labely, {b - 5.4, c}, {0, 0}, {0, 1}],
  g[[1, 2]] = g[[1, 2]] /.
    Text[labelx, {b_, c_}, {0., -1.}] ->
     Text[labelx, {b, c + 0.625}, {0, -1}, {1, 0}],
  g[[1, 2]] = g[[1, 2]] /.
    Text[a_, {b_, c_}, {0., -1.}] ->
     Text[a, {b, c}, {0, -1}, {0, 1}],
  flag = flag + 1,
  p[[n]] = Show[g,
    AspectRatio -> 1.05,
    PlotRange -> All,
    DisplayFunction -> Identity]},
 {n, 1, 5}]

(* Display Selected Eigengenes *)

g3 = Show[{p[[1]], p[[2]], p[[3]]}, DisplayFunction -> Identity];
g4 = Show[{p[[4]], p[[5]]}, DisplayFunction -> Identity];

(* Display Eigengenes, Fractions and Selected Eigengenes *)

Show[GraphicsArray[{g1, g2, g3, g4}],
  GraphicsSpacing -> -0.15];
```

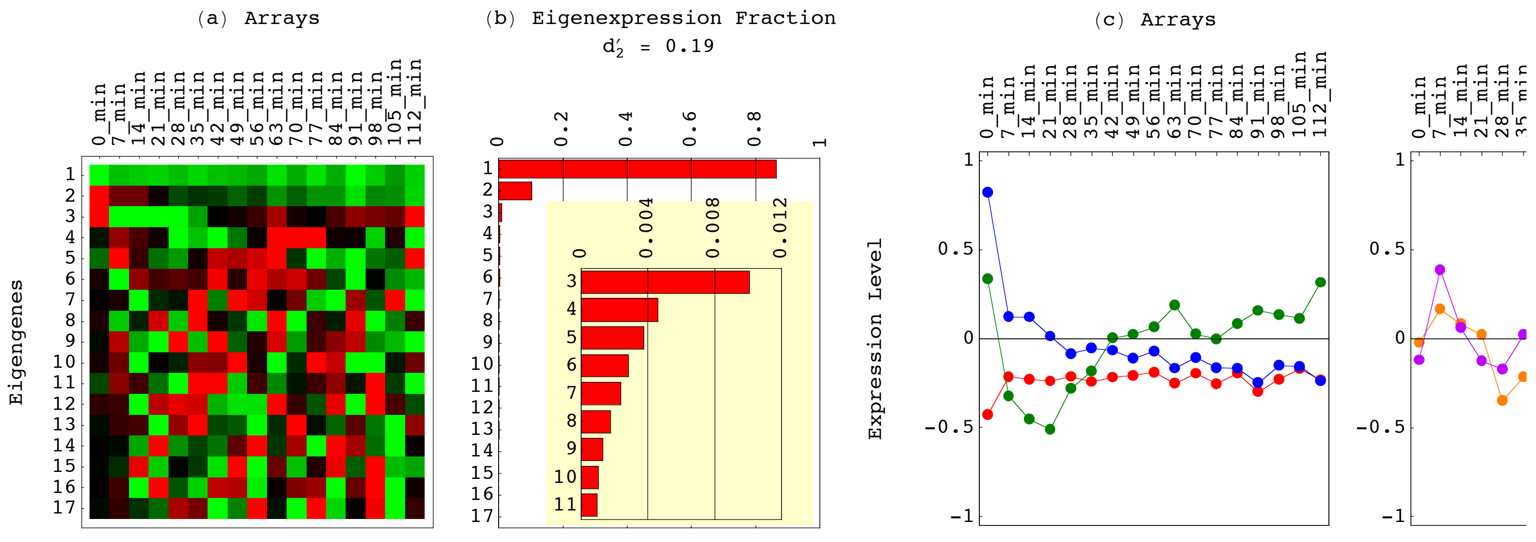

```
(* Choose Subset of Eigengenes for Estimation *)

eigengenes = TakeRows[eigengenes, {1, 5}];

(* Estimate Missing Data *)

Do[
 missingmatrix1[[a, locator1[[a, 1]]]] =
  N[Round[Flatten[Dot[Dot[
          Transpose[Drop[
            Transpose[{missingmatrix1[[a]]}],
            {locator1[[a, 1]]}]],
          PseudoInverse[Transpose[Drop[
             Transpose[eigengenes],
             {locator1[[a, 1]]}]]]],
         eigengenes]][[locator1[[a, 1]]]] * 100] / 100],
 {a, 1, numbers[[2]]}]

Do[Do[
  missingmatrix2[[a, locator2[[a, b]]]] =
   N[Round[Flatten[Dot[Dot[
          Transpose[Drop[Drop[
             Transpose[{missingmatrix2[[a]]}],
             {locator2[[a, 2]]}], {locator2[[a, 1]]}]],
          PseudoInverse[Transpose[Drop[Drop[
              Transpose[eigengenes],
              {locator2[[a, 2]]}], {locator2[[a, 1]]}]]]],
         eigengenes]][[locator2[[a, b]]]] * 100] / 100],
  {b, 1, 2}],
 {a, 1, numbers[[3]]}]

Do[Do[
  missingmatrix3[[a, locator3[[a, b]]]] =
   N[Round[Flatten[Dot[Dot[
          Transpose[Drop[Drop[Drop[
              Transpose[{missingmatrix3[[a]]}],
              {locator3[[a, 3]]}], {locator3[[a, 2]]}], {locator3[[a, 1]]}]],
          PseudoInverse[Transpose[Drop[Drop[Drop[
               Transpose[eigengenes],
               {locator3[[a, 3]]}], {locator3[[a, 2]]}], {locator3[[a, 1]]}]]]],
         eigengenes]][[locator3[[a, b]]]] * 100] / 100],
  {b, 1, 3}],
 {a, 1, numbers[[4]]}]

genenames = AppendColumns[
   fullgenenames,
   missinggenenames1,
   missinggenenames2,
   missinggenenames3];

matrix = AppendColumns[
   fullmatrix,
   missingmatrix1,
   missingmatrix2,
   missingmatrix3];
{genes, arrays} = Dimensions[matrix];
matrix2 = matrix;
genenames2 = genenames;
arraynames2 = arraynames;
{genes2, arrays2} = Dimensions[matrix2]

{4772, 17}
```

```
(* Examine S. cerevisiae Data After Missing Data Estimation *)

(* Calculate SVD *)

{eigenarrays, eigenexpressions, eigengenes} = SingularValues[matrix];
eigengenes[[4]] = -eigengenes[[4]];
eigenarrays = Transpose[eigenarrays];
fractions = eigenexpressions ^ 2 / Sum[eigenexpressions[[a]] ^ 2, {a, 1, arrays}];
entropy = -N[Sum[fractions[[a]] * Log[fractions[[a]]], {a, 1, arrays}] / Log[arrays]];
entropy = N[Round[100 * entropy] / 100]

0.19

(* Create Fractions Bar Charts Displays *)

fractions[[3]]

0.0104045

limit = 0.012;
Clear[gridx, framex, framey];
gridx = Table[a, {a, 0, limit, N[limit / 3]}];
framex = gridx;
framex = Table[{gridx[[a]], framex[[a]]}, {a, 1, 4}];
gridx = Table[{gridx[[a]], RGBColor[0, 0, 0]}, {a, 1, 4}];
framey = Table[{a + 1, arrays - a - 6}, {a, 0, 12 - 3}];
table = Table[fractions[[arrays - a]], {a, 6, arrays - 3}];
g = BarChart[table,
   BarOrientation -> Horizontal,
   PlotRange -> {{0, limit * 1.0001}, {0.5, 11 - 2 + 0.5}},
   AspectRatio -> 1,
   Axes -> False,
   Frame -> True,
   FrameTicks -> {None, framey, framex, None},
   FrameLabel -> {None, None, None, None},
   GridLines -> {gridx, None},
   DisplayFunction -> Identity];
g = FullGraphics[g];
g[[1, 2]] = g[[1, 2]] /.
   Text[a_, {b_, c_}, {0., -1.}] →
    Text[a, {b, c}, {0, -1}, {0, 1}];
g1 = Show[g,
   AspectRatio -> 1.25,
   PlotRange -> All,
   DisplayFunction -> Identity];

gridx = Table[a, {a, 0, 1, 0.2}];
framex = gridx;
framex = Table[{gridx[[a]], framex[[a]]}, {a, 1, 6}];
framex[[6]] = {1, "1"};
gridx = Table[{gridx[[a]], RGBColor[0, 0, 0]}, {a, 1, 6}];
framey = Table[{a + 1, arrays - a}, {a, 0, arrays - 1}];
labelx = ColumnForm[
   {"(b) Eigenexpression Fraction", StringJoin["d₂ = ", ToString[entropy]], " "},
   Center];
g = BarChart[
   Table[fractions[[arrays - a]], {a, 0, arrays - 1}],
   BarOrientation -> Horizontal,
   PlotRange -> {{0, 1.0001}, {0.5, arrays + 0.5}},
   AspectRatio -> 1,
   Axes -> False,
   Frame -> True,
   FrameTicks -> {None, framey, framex, None},
   FrameLabel -> {None, None, labelx, None},
   GridLines -> {gridx, None},
   DisplayFunction -> Identity];
```

```
g = FullGraphics[g];
g[[1, 2]] = g[[1, 2]] /.
   Text[labelx, {b_, c_}, {0., -1.}] ->
    Text[labelx, {b, c + 3}, {0, -1}, {1, 0}];
g[[1, 2]] = g[[1, 2]] /.
   Text[a_, {b_, c_}, {0., -1.}] ->
    Text[a, {b, c}, {0, -1}, {0, 1}];
g2 = Show[{g,
    Graphics[{RGBColor[1, 1, 0.8], Rectangle[{0.15, 0.6}, {0.98, 15.5}]}],
    Graphics[{Rectangle[{0.1, 0.6}, {0.98, 15.5}, g1]}]},
   AspectRatio -> 1.35,
   PlotRange -> All,
   DisplayFunction -> Identity];

(* Create Eigengenes 2 D Red & Green Raster Display *)

contrast = 3.5;
displaying = Table[
   If[contrast * eigengenes[[i, j]] > 0,
    If[contrast * eigengenes[[i, j]] < 1, {contrast * eigengenes[[i, j]], 0}, {1, 0}],
    If[contrast * eigengenes[[i, j]] > -1, {0, -contrast * eigengenes[[i, j]]}, {0, 1}]],
   {i, 1, arrays}, {j, 1, arrays}];
framex = Table[{a - 0.5, arraynames[[1, a]]}, {a, 1, arrays}];
framey = Table[{a + 1 - 0.5, arrays - a}, {a, 0, arrays - 1}];
labely = "Eigengenes";
labelx = ColumnForm[{"(a) Arrays", " ", " "}, Center];
g = Show[
   Graphics[
    RasterArray[
     Table[
      RGBColor[displaying[[i, j, 1]], displaying[[i, j, 2]], 0],
      {i, arrays, 1, -1}, {j, 1, arrays}]]],
   AspectRatio -> 1,
   Frame -> True,
   FrameTicks -> {None, framey, framex, None},
   FrameLabel -> {None, labely, labelx, None},
   DisplayFunction -> Identity];
g = FullGraphics[g];
g[[1, 2]] = g[[1, 2]] /.
   Text[labely, {b_, c_}, {1., 0.}] ->
    Text[labely, {b - 3, c}, {0, 0}, {0, 1}];
g[[1, 2]] = g[[1, 2]] /.
   Text[labelx, {b_, c_}, {0., -1.}] ->
    Text[labelx, {b, c + 3}, {0, -1}, {1, 0}];
g[[1, 2]] = g[[1, 2]] /.
   Text[a_, {b_, c_}, {0., -1.}] ->
    Text[a, {b, c}, {0, -1}, {0, 1}];
g1 = Show[g,
   AspectRatio -> 1.05,
   PlotRange -> All,
   DisplayFunction -> Identity];

(* Create Selected Eigengenes Graph Display *)

p = Table[0, {5}];
color = {RGBColor[1, 0, 0], RGBColor[0, 0, 1], RGBColor[0, 0.5, 0],
   RGBColor[1, 0.5, 0], RGBColor[0.75, 0, 1]};
xlabels = {"(c) Arrays", " ", " ", "(d) Arrays", " "};
ylabels = {{" ", "Expression Level"}, {" ", " "}, {" ", " "}, {" ", " "}, {" ", " "}};
framex = Table[{a - 1, arraynames[[1, a]]}, {a, 1, arrays}];
framey = {-1, -0.5, 0, 0.5, 1};
flag = 0;
```

```
Do[{
  labelx = ColumnForm[{xlabels[[n]]}, Center],
  labely = ColumnForm[ylabels[[n]], Center],
  coordinates = Table[{a - 1, eigengenes[[n, a]]}, {a, 1, arrays}],
  points = Table[Point[coordinates[[a]]], {a, 1, arrays}],
  line = Line[coordinates],
  If[n == 4, flag = 0],
  g = Show[{
     Graphics[{color[[n]], PointSize[0.022], points}],
     Graphics[{color[[n]], line}]},
    If[flag == 0,
     {FrameLabel -> {None, labely, labelx, None},
      FrameTicks -> {None, framey, framex, None}},
     {FrameLabel -> {None, labely, labelx, None},
      FrameTicks -> {None, None, None, None}}],
    Frame -> True,
    GridLines -> {None, {{0, RGBColor[0, 0, 0]}}},
    PlotRange -> {-1.05, 1.05},
    DisplayFunction -> Identity],
  g = FullGraphics[g],
  g[[1, 2]] = g[[1, 2]] /.
    Text[labely, {b_, c_}, {1., 0.}] ->
     Text[labely, {b - 5.4, c}, {0, 0}, {0, 1}],
  g[[1, 2]] = g[[1, 2]] /.
    Text[labelx, {b_, c_}, {0., -1.}] ->
     Text[labelx, {b, c + 0.625}, {0, -1}, {1, 0}],
  g[[1, 2]] = g[[1, 2]] /.
    Text[a_, {b_, c_}, {0., -1.}] ->
     Text[a, {b, c}, {0, -1}, {0, 1}],
  flag = flag + 1,
  p[[n]] = Show[g,
    AspectRatio -> 1.05,
    PlotRange -> All,
    DisplayFunction -> Identity]},
 {n, 1, 5}]

(* Display Selected Eigengenes *)

g3 = Show[{p[[1]], p[[2]], p[[3]]}, DisplayFunction -> Identity];
g4 = Show[{p[[4]], p[[5]]}, DisplayFunction -> Identity];

(* Display Eigengenes, Fractions and Selected Eigengenes *)

Show[GraphicsArray[{g1, g2, g3, g4}],
  GraphicsSpacing -> -0.15];
```

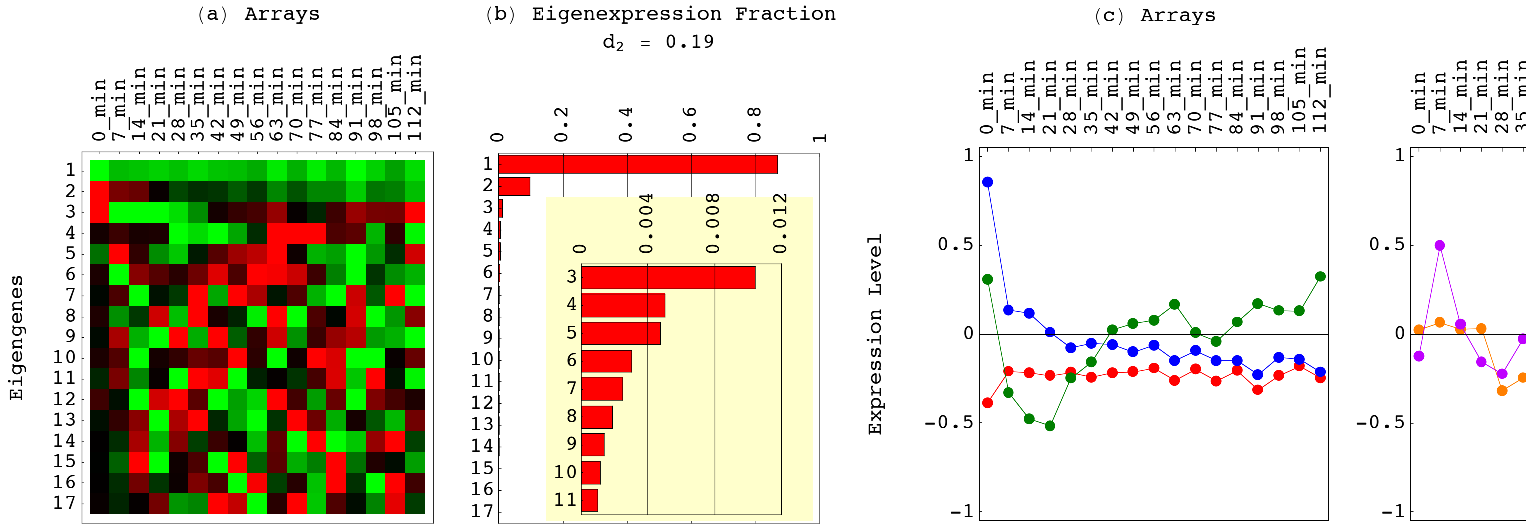

```
(* Estimate Missing Human Cell Cycle Data Using SVD *)
```

```
(* Read Human Data *)

stream = "Desktop/HO_GSVD/Data/Human.txt";
matrix = Import[stream, "Table"];
{genes, arrays} = Dimensions[matrix] - {1, 3}
Clear[stream];
```

```
{13068, 17}
```

```
genenames = TakeRows[
   TakeColumns[matrix, {1, 3}],
   {2, genes + 1}];
arraynames = TakeColumns[
   TakeRows[matrix, {1, 1}],
   {4, arrays + 3}];
matrix = TakeColumns[
   TakeRows[matrix, {2, genes + 1}],
   {4, arrays + 3}];
matrix = ToExpression[matrix];

(* Count Null Data *)

counter = Table[Dimensions[Position[matrix[[a]], Null]][[1]], {a, 1, genes}];

(* Locate Gene Position of Null Data *)

Clear[positions];
positions = Table[0, {a, 1, arrays + 1}];
Do[
  positions[[a]] = Flatten[
    Position[Flatten[counter], a - 1]],
  {a, 1, arrays + 1}];
numbers = Flatten[
   Table[
    Dimensions[positions[[a]]],
    {a, 1, Round[arrays * 0.25]}]];

(* Create Display Of Gene Position Of Null Data *)

framex = Table[{a, a - 1}, {a, 1, Round[arrays * 0.25]}];
framey = {1000, 2000, 3000, 4000, 5000};
labelx = ColumnForm[{"Number of Arrays"}, Center];
labely = ColumnForm[{"Number of Genes"}, Center];
g = BarChart[numbers,
    Frame -> True,
    Axes -> False,
    FrameLabel -> {labelx, labely, None, None},
    FrameTicks -> {framex, framey, None, None},
    GridLines -> {None, None},
    PlotRange -> {{0.5, Round[arrays * 0.25] + 0.5}, {0, 5000}},
    DisplayFunction -> Identity];
g = FullGraphics[g];
g[[1, 2]] = g[[1, 2]] /.
        Text[labely, {b_, c_}, {1., 0.}] ->
            Text[labely, {b - 0.75, c}, {0, 0}, {0, 1}];
g[[1, 2]] = g[[1, 2]] /.
        Text[labelx, {b_, c_}, {0., 1.}] ->
            Text[labelx, {b, c - 600}, {0, 1}, {1, 0}];
```

```
(* Display Gene Position Of Null Data *)

Show[g, PlotRange -> All];
```

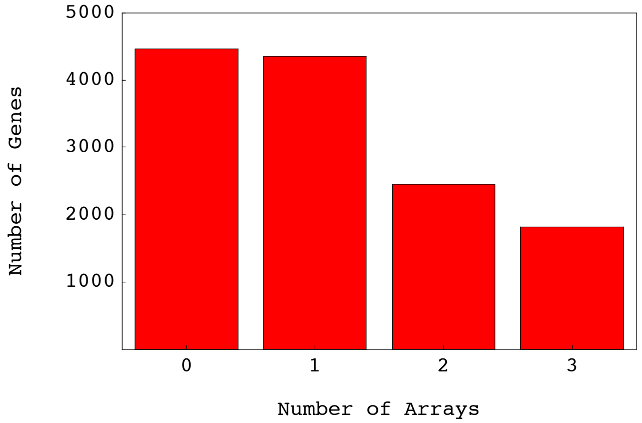


```
(* Select Genes by Number of Missing Data Points *)

matrix = AppendRows[Table[{counter[[a]]}, {a, 1, genes}], genenames, matrix];
matrix = Sort[matrix, OrderedQ[{#1, #2}] &];
fullgenenames = TakeColumns[
   TakeRows[matrix, {1, numbers[[1]]}],
   {2, 4}];
fullmatrix = TakeColumns[
   TakeRows[matrix, {1, numbers[[1]]}],
   {5, arrays + 4}];
missinggenenames1 = TakeColumns[
   TakeRows[matrix, {numbers[[1]] + 1, numbers[[1]] + numbers[[2]]}],
   {2, 4}];
missingmatrix1 = TakeColumns[
   TakeRows[matrix, {numbers[[1]] + 1, numbers[[1]] + numbers[[2]]}],
   {5, arrays + 4}];
missinggenenames2 = TakeColumns[
   TakeRows[matrix,
    {numbers[[1]] + numbers[[2]] + 1,
     numbers[[1]] + numbers[[2]] + numbers[[3]]}],
   {2, 4}];
missingmatrix2 = TakeColumns[
   TakeRows[matrix,
    {numbers[[1]] + numbers[[2]] + 1,
     numbers[[1]] + numbers[[2]] + numbers[[3]]}],
   {5, arrays + 4}];
missinggenenames3 = TakeColumns[
   TakeRows[matrix,
    {numbers[[1]] + numbers[[2]] + numbers[[3]] + 1,
     numbers[[1]] + numbers[[2]] + numbers[[3]] + numbers[[4]]}],
   {2, 4}];
missingmatrix3 = TakeColumns[
   TakeRows[matrix,
    {numbers[[1]] + numbers[[2]] + numbers[[3]] + 1,
     numbers[[1]] + numbers[[2]] + numbers[[3]] + numbers[[4]]}],
   {5, arrays + 4}];
```

```
(* Locate Array Position of Null Data *)

locator1 = Table[0, {numbers[[2]]}];
Do[
  locator1[[a]] = locator1[[a]] + Flatten[Position[missingmatrix1[[a]], Null]],
  {a, 1, numbers[[2]]}];
locator2 = Table[0, {numbers[[3]]}];
Do[
  locator2[[a]] = locator2[[a]] + Flatten[Position[missingmatrix2[[a]], Null]],
  {a, 1, numbers[[3]]}];
locator3 = Table[0, {numbers[[4]]}];
Do[
  locator3[[a]] = locator3[[a]] + Flatten[Position[missingmatrix3[[a]], Null]],
  {a, 1, numbers[[4]]}];

(* Sort Raw Data According to the Position of Missing Data Points for Each Gene *)

missingmatrix1 = AppendRows[locator1, missinggenenames1, missingmatrix1];
missingmatrix1 = Sort[missingmatrix1, OrderedQ[{#1, #2}] &];
locator1 = TakeColumns[missingmatrix1, {1, 1}];
missinggenenames1 = TakeColumns[missingmatrix1, {2, 4}];
missingmatrix1 = TakeColumns[missingmatrix1, {5, arrays + 4}];
missingmatrix2 = AppendRows[locator2, missinggenenames2, missingmatrix2];
missingmatrix2 = Sort[missingmatrix2, OrderedQ[{#1, #2}] &];
locator2 = TakeColumns[missingmatrix2, {1, 2}];
missinggenenames2 = TakeColumns[missingmatrix2, {3, 5}];
missingmatrix2 = TakeColumns[missingmatrix2, {6, arrays + 5}];
missingmatrix3 = AppendRows[locator3, missinggenenames3, missingmatrix3];
missingmatrix3 = Sort[missingmatrix3, OrderedQ[{#1, #2}] &];
locator3 = TakeColumns[missingmatrix3, {1, 3}];
missinggenenames3 = TakeColumns[missingmatrix3, {4, 6}];
missingmatrix3 = TakeColumns[missingmatrix3, {7, arrays + 6}];

(* Examine Subset of Genes with Full Data *)

(* Calculate SVD *)

{eigenarrays, eigenexpressions, eigengenes} = SingularValues[fullmatrix];
eigenarrays = Transpose[eigenarrays];
fractions = eigenexpressions^2 / Sum[eigenexpressions[[a]]^2, {a, 1, arrays}];
entropy = -N[Sum[fractions[[a]] * Log[fractions[[a]]], {a, 1, arrays}] / Log[arrays]];
entropy = N[Round[100 * entropy] / 100]

0.05

(* Create Fractions Bar Charts Displays *)

fractions[[2]]

0.00599627
```

```
limit = 0.006;
Clear[gridx, framex, framey];
gridx = Table[a, {a, 0, limit, N[limit / 3]}];
framex = gridx;
framex = Table[{gridx[[a]], framex[[a]]}, {a, 1, 4}];
gridx = Table[{gridx[[a]], RGBColor[0, 0, 0]}, {a, 1, 4}];
framey = Table[{a + 1, arrays - a - 6}, {a, 0, 12 - 3}];
table = Table[fractions[[arrays - a]], {a, 6, arrays - 2}];
g = BarChart[table,
   BarOrientation -> Horizontal,
   PlotRange -> {{0, limit * 1.0001}, {0.5, 12 - 2 + 0.5}},
   AspectRatio -> 1,
   Axes -> False,
   Frame -> True,
   FrameTicks -> {None, framey, framex, None},
   FrameLabel -> {None, None, None, None},
   GridLines -> {gridx, None},
   DisplayFunction -> Identity];
g = FullGraphics[g];
g[[1, 2]] = g[[1, 2]] /.
   Text[a_, {b_, c_}, {0., -1.}] →
    Text[a, {b, c}, {0, -1}, {0, 1}];
g1 = Show[g,
   AspectRatio -> 1.25,
   PlotRange -> All,
   DisplayFunction -> Identity];

gridx = Table[a, {a, 0, 1, 0.2}];
framex = gridx;
framex = Table[{gridx[[a]], framex[[a]]}, {a, 1, 6}];
framex[[6]] = {1, "1"};
gridx = Table[{gridx[[a]], RGBColor[0, 0, 0]}, {a, 1, 6}];
framey = Table[{a + 1, arrays - a}, {a, 0, arrays - 1}];
labelx = ColumnForm[
   {"(b) Eigenexpression Fraction", StringJoin["d'_3 = ", ToString[entropy]], " "},
   Center];
g = BarChart[
   Table[fractions[[arrays - a]], {a, 0, arrays - 1}],
   BarOrientation -> Horizontal,
   PlotRange -> {{0, 1.0001}, {0.5, arrays + 0.5}},
   AspectRatio -> 1,
   Axes -> False,
   Frame -> True,
   FrameTicks -> {None, framey, framex, None},
   FrameLabel -> {None, None, labelx, None},
   GridLines -> {gridx, None},
   DisplayFunction -> Identity];
g = FullGraphics[g];
g[[1, 2]] = g[[1, 2]] /.
   Text[labelx, {b_, c_}, {0., -1.}] →
    Text[labelx, {b, c + 3}, {0, -1}, {1, 0}];
g[[1, 2]] = g[[1, 2]] /.
   Text[a_, {b_, c_}, {0., -1.}] →
    Text[a, {b, c}, {0, -1}, {0, 1}];
g2 = Show[{g,
    Graphics[{RGBColor[1, 1, 0.8], Rectangle[{0.1, 0.6}, {0.98, 15.5}]}],
    Graphics[{Rectangle[{0.1, 0.6}, {0.98, 15.5}, g1]}]}],
   AspectRatio -> 1.35,
   PlotRange -> All,
   DisplayFunction -> Identity];
```

```
(* Create Eigengenes 2 D Red & Green Raster Display *)

contrast = 3.5;
displaying = Table[
   If[contrast * eigengenes[[i, j]] > 0,
    If[contrast * eigengenes[[i, j]] < 1, {contrast * eigengenes[[i, j]], 0}, {1, 0}],
    If[contrast * eigengenes[[i, j]] > -1, {0, -contrast * eigengenes[[i, j]]}, {0, 1}]],
   {i, 1, arrays}, {j, 1, arrays}];
framex = Table[{a - 0.5, arraynames[[1, a]]}, {a, 1, arrays}];
framey = Table[{a + 1 - 0.5, arrays - a}, {a, 0, arrays - 1}];
labely = "Eigengenes";
labelx = ColumnForm[{"(a) Arrays", " ", " "}, Center];
g = Show[
   Graphics[
    RasterArray[
     Table[
      RGBColor[displaying[[i, j, 1]], displaying[[i, j, 2]], 0],
      {i, arrays, 1, -1}, {j, 1, arrays}]]],
   AspectRatio -> 1,
   Frame -> True,
   FrameTicks -> {None, framey, framex, None},
   FrameLabel -> {None, labely, labelx, None},
   DisplayFunction -> Identity];
g = FullGraphics[g];
g[[1, 2]] = g[[1, 2]] /.
   Text[labely, {b_, c_}, {1., 0.}] →
    Text[labely, {b - 3, c}, {0, 0}, {0, 1}];
g[[1, 2]] = g[[1, 2]] /.
   Text[labelx, {b_, c_}, {0., -1.}] →
    Text[labelx, {b, c + 3}, {0, -1}, {1, 0}];

g[[1, 2]] = g[[1, 2]] /.
   Text[a_, {b_, c_}, {0., -1.}] →
    Text[a, {b, c}, {0, -1}, {0, 1}];
g1 = Show[g,
   AspectRatio -> 1.05,
   PlotRange -> All,
   DisplayFunction -> Identity];

(* Create Selected Eigengenes Graph Display *)

p = Table[0, {5}];
color =
  {RGBColor[1, 0, 0], RGBColor[0, 0, 1], RGBColor[0, 0.5, 0], RGBColor[1, 0.5, 0], RGBColor[0.75, 0, 1]};
xlabels = {"(c) Arrays", " ", " ", "(d) Arrays", " "};
ylabels = {{" ", "Expression Level"}, {" ", " "}, {" ", " "}, {" ", " "}, {" ", " "}};
framex = Table[{a - 1, arraynames[[1, a]]}, {a, 1, arrays}];
framey = {-1, -0.5, 0, 0.5, 1};
flag = 0;
```

```
Do[{
  labelx = ColumnForm[{xlabels[[n]]}, Center],
  labely = ColumnForm[ylabels[[n]], Center],
  coordinates = Table[{a - 1, eigengenes[[n, a]]}, {a, 1, arrays}],
  points = Table[Point[coordinates[[a]]], {a, 1, arrays}],
  line = Line[coordinates],
  If[n == 4, flag = 0],
  g = Show[{
     Graphics[{color[[n]], PointSize[0.022], points}],
     Graphics[{color[[n]], line}]},
    If[flag == 0,
     {FrameLabel -> {None, labely, labelx, None},
      FrameTicks -> {None, framey, framex, None}},
     {FrameLabel -> {None, labely, labelx, None},
      FrameTicks -> {None, None, None, None}}],
    Frame -> True,
    GridLines -> {None, {{0, RGBColor[0, 0, 0]}}},
    PlotRange -> {-1.05, 1.05},
    DisplayFunction -> Identity],
  g = FullGraphics[g],
  g[[1, 2]] = g[[1, 2]] /.
    Text[labely, {b_, c_}, {1., 0.}] ->
     Text[labely, {b - 5.4, c}, {0, 0}, {0, 1}],
  g[[1, 2]] = g[[1, 2]] /.
    Text[labelx, {b_, c_}, {0., -1.}] ->
     Text[labelx, {b, c + 0.625}, {0, -1}, {1, 0}],
  g[[1, 2]] = g[[1, 2]] /.
    Text[a_, {b_, c_}, {0., -1.}] ->
     Text[a, {b, c}, {0, -1}, {0, 1}],
  flag = flag + 1,
  p[[n]] = Show[g,
    AspectRatio -> 1.05,
    PlotRange -> All,
    DisplayFunction -> Identity]},
 {n, 1, 5}]

(* Display Selected Eigengenes *)

g3 = Show[{p[[1]], p[[2]], p[[3]]}, DisplayFunction -> Identity];
g4 = Show[{p[[4]], p[[5]]}, DisplayFunction -> Identity];

(* Display Eigengenes, Fractions and Selected Eigengenes *)

Show[GraphicsArray[{g1, g2, g3, g4}],
  GraphicsSpacing -> -0.15];
```

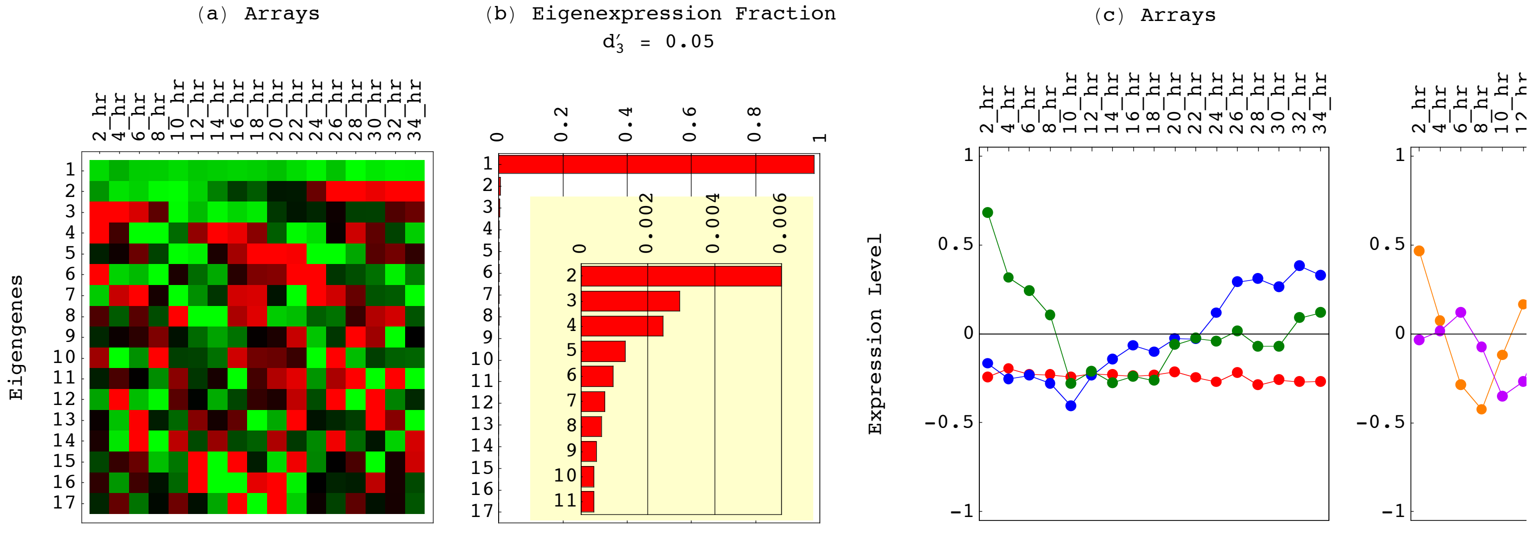

```
(* Choose Subset of Eigengenes for Estimation *)

eigengenes = TakeRows[eigengenes, {1, 5}];

(* Estimate Missing Data *)

Do[
 missingmatrix1[[a, locator1[[a, 1]]]] =
  N[Round[Flatten[Dot[Dot[
        Transpose[Drop[
          Transpose[{missingmatrix1[[a]]}],
          {locator1[[a, 1]]}]],
        PseudoInverse[Transpose[Drop[
           Transpose[eigengenes],
           {locator1[[a, 1]]}]]]],
       eigengenes]][[locator1[[a, 1]]]] * 100] / 100],
 {a, 1, numbers[[2]]}]

Do[Do[
  missingmatrix2[[a, locator2[[a, b]]]] =
   N[Round[Flatten[Dot[Dot[
        Transpose[Drop[Drop[
           Transpose[{missingmatrix2[[a]]}],
           {locator2[[a, 2]]}], {locator2[[a, 1]]}]],
        PseudoInverse[Transpose[Drop[Drop[
            Transpose[eigengenes],
            {locator2[[a, 2]]}], {locator2[[a, 1]]}]]]],
       eigengenes]][[locator2[[a, b]]]] * 100] / 100],
  {b, 1, 2}],
 {a, 1, numbers[[3]]}]

Do[Do[
  missingmatrix3[[a, locator3[[a, b]]]] =
   N[Round[Flatten[Dot[Dot[
        Transpose[Drop[Drop[Drop[
            Transpose[{missingmatrix3[[a]]}],
            {locator3[[a, 3]]}], {locator3[[a, 2]]}], {locator3[[a, 1]]}]],
        PseudoInverse[Transpose[Drop[Drop[Drop[
             Transpose[eigengenes],
             {locator3[[a, 3]]}], {locator3[[a, 2]]}], {locator3[[a, 1]]}]]]],
       eigengenes]][[locator3[[a, b]]]] * 100] / 100],
  {b, 1, 3}],
 {a, 1, numbers[[4]]}]

genenames = AppendColumns[
   fullgenenames,
   missinggenenames1,
   missinggenenames2,
   missinggenenames3];
matrix = AppendColumns[
   fullmatrix,
   missingmatrix1,
   missingmatrix2,
   missingmatrix3];
{genes, arrays} = Dimensions[matrix];
matrix3 = matrix;
genenames3 = genenames;
arraynames3 = arraynames;
{genes3, arrays3} = Dimensions[matrix3]

{13068, 17}
```

```
(* Examine Human Data After Missing Data Estimation *)

(* Calculate SVD *)

{eigenarrays, eigenexpressions, eigengenes} = SingularValues[matrix];
eigenarrays = Transpose[eigenarrays];
fractions = eigenexpressions^2 / Sum[eigenexpressions[[a]]^2, {a, 1, arrays}];
entropy = -N[Sum[fractions[[a]] * Log[fractions[[a]]], {a, 1, arrays}] / Log[arrays]];
entropy = N[Round[100 * entropy] / 100]

0.04

(* Create Fractions Bar Charts Displays *)

fractions[[2]]

0.00538975

limit = 0.006;
Clear[gridx, framex, framey];
gridx = Table[a, {a, 0, limit, N[limit / 3]}];
framex = gridx;
framex = Table[{gridx[[a]], framex[[a]]}, {a, 1, 4}];
gridx = Table[{gridx[[a]], RGBColor[0, 0, 0]}, {a, 1, 4}];
framey = Table[{a + 1, arrays - a - 6}, {a, 0, 12 - 3}];
table = Table[fractions[[arrays - a]], {a, 6, arrays - 2}];
g = BarChart[table,
   BarOrientation -> Horizontal,
   PlotRange -> {{0, limit * 1.0001}, {0.5, 12 - 2 + 0.5}},
   AspectRatio -> 1,
   Axes -> False,
   Frame -> True,
   FrameTicks -> {None, framey, framex, None},
   FrameLabel -> {None, None, None, None},
   GridLines -> {gridx, None},
   DisplayFunction -> Identity];
g = FullGraphics[g];
g[[1, 2]] = g[[1, 2]] /.
   Text[a_, {b_, c_}, {0., -1.}] ->
    Text[a, {b, c}, {0, -1}, {0, 1}];
g1 = Show[g,
   AspectRatio -> 1.25,
   PlotRange -> All,
   DisplayFunction -> Identity];

gridx = Table[a, {a, 0, 1, 0.2}];
framex = gridx;
framex = Table[{gridx[[a]], framex[[a]]}, {a, 1, 6}];
framex[[6]] = {1, "1"};
gridx = Table[{gridx[[a]], RGBColor[0, 0, 0]}, {a, 1, 6}];
framey = Table[{a + 1, arrays - a}, {a, 0, arrays - 1}];
labelx = ColumnForm[
   {"(b) Eigenexpression Fraction", StringJoin["d₃ = ", ToString[entropy]], " "},
   Center];
g = BarChart[
   Table[fractions[[arrays - a]], {a, 0, arrays - 1}],
   BarOrientation -> Horizontal,
   PlotRange -> {{0, 1.0001}, {0.5, arrays + 0.5}},
   AspectRatio -> 1,
   Axes -> False,
   Frame -> True,
   FrameTicks -> {None, framey, framex, None},
   FrameLabel -> {None, None, labelx, None},
   GridLines -> {gridx, None},
   DisplayFunction -> Identity];
```

```
g = FullGraphics[g];
g[[1, 2]] = g[[1, 2]] /.
   Text[labelx, {b_, c_}, {0., -1.}] ->
    Text[labelx, {b, c + 3}, {0, -1}, {1, 0}];
g[[1, 2]] = g[[1, 2]] /.
   Text[a_, {b_, c_}, {0., -1.}] ->
    Text[a, {b, c}, {0, -1}, {0, 1}];
g2 = Show[{g,
    Graphics[{RGBColor[1, 1, 0.8], Rectangle[{0.1, 0.6}, {0.98, 15.5}]}],
    Graphics[{Rectangle[{0.1, 0.6}, {0.98, 15.5}, g1]}]},
   AspectRatio -> 1.35,
   PlotRange -> All,
   DisplayFunction -> Identity];

(* Create Eigengenes 2 D Red & Green Raster Display *)

contrast = 3.5;
displaying = Table[
   If[contrast * eigengenes[[i, j]] > 0,
    If[contrast * eigengenes[[i, j]] < 1, {contrast * eigengenes[[i, j]], 0}, {1, 0}],
    If[contrast * eigengenes[[i, j]] > -1, {0, -contrast * eigengenes[[i, j]]}, {0, 1}]],
   {i, 1, arrays}, {j, 1, arrays}];
framex = Table[{a - 0.5, arraynames[[1, a]]}, {a, 1, arrays}];
framey = Table[{a + 1 - 0.5, arrays - a}, {a, 0, arrays - 1}];
labely = "Eigengenes";
labelx = ColumnForm[{"(a) Arrays", " ", " "}, Center];
g = Show[
   Graphics[
    RasterArray[
     Table[
      RGBColor[displaying[[i, j, 1]], displaying[[i, j, 2]], 0],
      {i, arrays, 1, -1}, {j, 1, arrays}]]],
   AspectRatio -> 1,
   Frame -> True,
   FrameTicks -> {None, framey, framex, None},
   FrameLabel -> {None, labely, labelx, None},
   DisplayFunction -> Identity];
g = FullGraphics[g];
g[[1, 2]] = g[[1, 2]] /.
   Text[labely, {b_, c_}, {1., 0.}] ->
    Text[labely, {b - 3, c}, {0, 0}, {0, 1}];
g[[1, 2]] = g[[1, 2]] /.
   Text[labelx, {b_, c_}, {0., -1.}] ->
    Text[labelx, {b, c + 3}, {0, -1}, {1, 0}];
g[[1, 2]] = g[[1, 2]] /.
   Text[a_, {b_, c_}, {0., -1.}] ->
    Text[a, {b, c}, {0, -1}, {0, 1}];
g1 = Show[g,
   AspectRatio -> 1.05,
   PlotRange -> All,
   DisplayFunction -> Identity];

(* Create Selected Eigengenes Graph Display *)

p = Table[0, {5}];
color =
  {RGBColor[1, 0, 0], RGBColor[0, 0, 1], RGBColor[0, 0.5, 0], RGBColor[1, 0.5, 0], RGBColor[0.75, 0, 1]};
xlabels = {"(c) Arrays", " ", " ", "(d) Arrays", " "};
ylabels = {{" ", "Expression Level"}, {" ", " "}, {" ", " "}, {" ", " "}, {" ", " "}};
framex = Table[{a - 1, arraynames[[1, a]]}, {a, 1, arrays}];
framey = {-1, -0.5, 0, 0.5, 1};
flag = 0;
```

```
Do[{
  labelx = ColumnForm[{xlabels[[n]]}, Center];
  labely = ColumnForm[ylabels[[n]], Center];
  coordinates = Table[{a - 1, eigengenes[[n, a]]}, {a, 1, arrays}];
  points = Table[Point[coordinates[[a]]], {a, 1, arrays}];
  line = Line[coordinates];
  If[n == 4, flag = 0];
  g = Show[{
     Graphics[{color[[n]], PointSize[0.022], points}],
     Graphics[{color[[n]], line}]},
    If[flag == 0,
     {FrameLabel -> {None, labely, labelx, None},
      FrameTicks -> {None, framey, framex, None}},
     {FrameLabel -> {None, labely, labelx, None},
      FrameTicks -> {None, None, None, None}}],
    Frame -> True,
    GridLines -> {None, {{0, RGBColor[0, 0, 0]}}},
    PlotRange -> {-1.05, 1.05},
    DisplayFunction -> Identity],
  g = FullGraphics[g],
  g[[1, 2]] = g[[1, 2]] /.
    Text[labely, {b_, c_}, {1., 0.}] ->
     Text[labely, {b - 5.4, c}, {0, 0}, {0, 1}],
  g[[1, 2]] = g[[1, 2]] /.
    Text[labelx, {b_, c_}, {0., -1.}] ->
     Text[labelx, {b, c + 0.625}, {0, -1}, {1, 0}],
  g[[1, 2]] = g[[1, 2]] /.
    Text[a_, {b_, c_}, {0., -1.}] ->
     Text[a, {b, c}, {0, -1}, {0, 1}],
  flag = flag + 1,
  p[[n]] = Show[g,
    AspectRatio -> 1.05,
    PlotRange -> All,
    DisplayFunction -> Identity]},
 {n, 1, 5}]

(* Display Selected Eigengenes *)

g3 = Show[{p[[1]], p[[2]], p[[3]]}, DisplayFunction -> Identity];
g4 = Show[{p[[4]], p[[5]]}, DisplayFunction -> Identity];

(* Display Eigengenes, Fractions and Selected Eigengenes *)

Show[GraphicsArray[{g1, g2, g3, g4}],
  GraphicsSpacing -> -0.15];
```

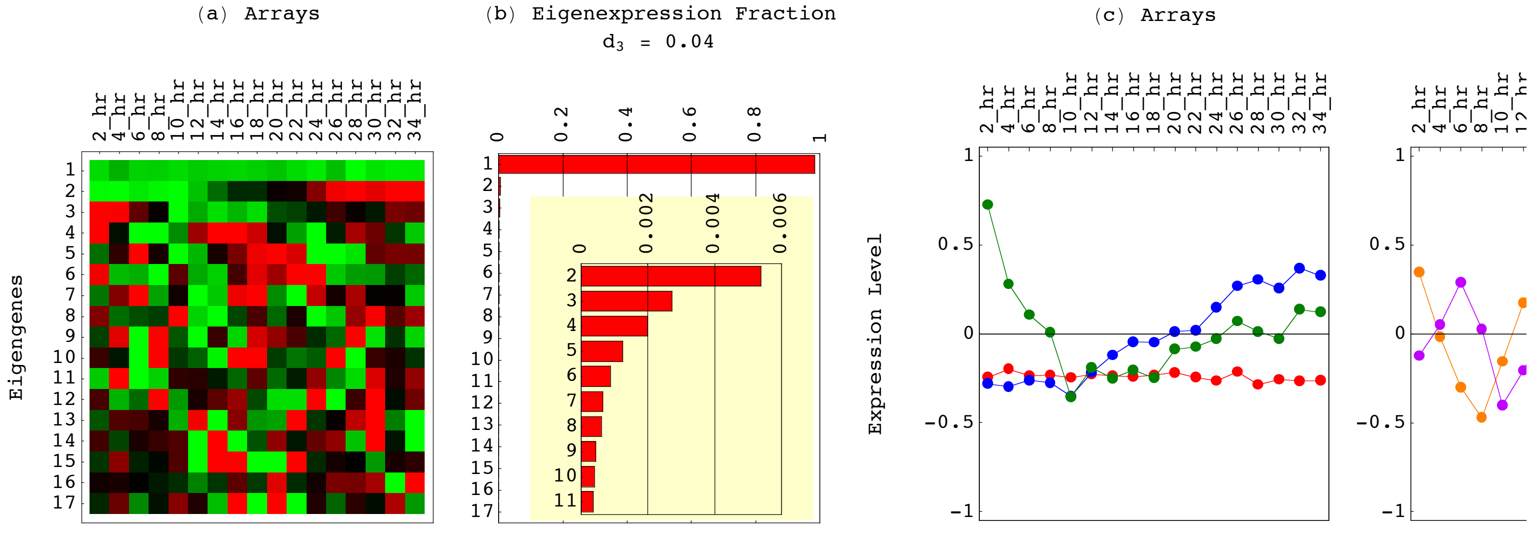

```
(* HO GSVD of S. pombe, S. cerevisiae and Human Cell Cycle Datasets *)

(* Calculate HO GSVD of S. pombe, S. cerevisiae and Human Cell Cycle Datasets *)

cor1 = Dot[Transpose[matrix1], matrix1];
cor2 = Dot[Transpose[matrix2], matrix2];
cor3 = Dot[Transpose[matrix3], matrix3];
cor = Dot[cor1, Inverse[cor2]] + Dot[cor2, Inverse[cor1]] + Dot[cor2, Inverse[cor3]] +
   Dot[cor3, Inverse[cor2]] + Dot[cor1, Inverse[cor3]] + Dot[cor3, Inverse[cor1]];
{values, vectors} = Eigensystem[cor];
values = values / 6;
genelets = vectors;
Do[genelets[[a]] = genelets[[a]] / Sqrt[Dot[genelets[[a]], genelets[[a]]]], {a, 1, arrays}];

arraylets1 = Dot[matrix1, Inverse[genelets]];
arraylets2 = Dot[matrix2, Inverse[genelets]];
arraylets3 = Dot[matrix3, Inverse[genelets]];
arraylets1 = Transpose[arraylets1];
Do[arraylets1[[a]] = arraylets1[[a]] / Sqrt[Dot[arraylets1[[a]], arraylets1[[a]]]], {a, 1, arrays}];
arraylets1 = Transpose[arraylets1];
arraylets2 = Transpose[arraylets2];
Do[arraylets2[[a]] = arraylets2[[a]] / Sqrt[Dot[arraylets2[[a]], arraylets2[[a]]]], {a, 1, arrays}];
arraylets2 = Transpose[arraylets2];
arraylets3 = Transpose[arraylets3];
Do[arraylets3[[a]] = arraylets3[[a]] / Sqrt[Dot[arraylets3[[a]], arraylets3[[a]]]], {a, 1, arrays}];
arraylets3 = Transpose[arraylets3];
d1 = Chop[Dot[PseudoInverse[arraylets1], matrix1, Inverse[genelets]]];
d2 = Chop[Dot[PseudoInverse[arraylets2], matrix2, Inverse[genelets]]];
d3 = Chop[Dot[PseudoInverse[arraylets3], matrix3, Inverse[genelets]]];

(* Create Arraylets Correlations 2 D Red & Green Raster Displays *)

C1 = Chop[Dot[Transpose[arraylets1], arraylets1], 0.33];
C2 = Chop[Dot[Transpose[arraylets2], arraylets2], 0.33];
C3 = Chop[Dot[Transpose[arraylets3], arraylets3], 0.33];
Ctensor = {C1, C2, C3};

p = Table[0, {5}];
arraynames = Table[a, {a, 1, arrays}];
contrast = 3;
framex = Table[{a - 0.5, ToString[a]}, {a, 1, arrays}];
framey = Table[{a + 1 - 0.5, arrays - a}, {a, 0, arrays - 1}];
ylabels = {"Arraylets", " ", " "};
xlabels = {{"(a) S. pombe Arraylets"},
   {"(b) S. cerevisiae Arraylets"},
   {"(c) Human Arraylets"}};
```

```
Do[{
  displaying = Table[
    If[contrast * Ctensor[[n]][[i, j]] > 0,
     If[contrast * Ctensor[[n]][[i, j]] < 1, {contrast * Ctensor[[n]][[i, j]], 0}, {1, 0}],
     If[contrast * Ctensor[[n]][[i, j]] > -1, {0, -contrast * Ctensor[[n]][[i, j]]}, {0, 1}]],
    {i, 1, arrays}, {j, 1, arrays}],
  labelx = ColumnForm[xlabels[[n]], Center],
  labely = ylabels[[n]],
  g = Show[
    Graphics[
     RasterArray[
      Table[
       RGBColor[displaying[[i, j, 1]], displaying[[i, j, 2]], 0],
       {i, arrays, 1, -1}, {j, 1, arrays}]]],
    AspectRatio -> 1,
    Frame -> True,
    FrameTicks -> {None, framey, framex, None},
    FrameLabel -> {None, labely, labelx, None},
    GridLines -> {{{12, {RGBColor[1, 1, 1]}}}, {{arrays - 12, {RGBColor[1, 1, 1]}}}},
    DisplayFunction -> Identity],
  g = FullGraphics[g],
  g[[1, 2]] = g[[1, 2]] /.
    Text[labely, {b_, c_}, {1., 0.}] ->
     Text[labely, {b - 2.5, c}, {0, 0}, {0, 1}],
  g[[1, 2]] = g[[1, 2]] /.
    Text[labelx, {b_, c_}, {0., -1.}] ->
     Text[labelx, {b, c + 2}, {0, -1}, {1, 0}],
  g[[1, 2]] = g[[1, 2]] /.
    Text[a_, {b_, c_}, {0., -1.}] ->
     Text[a, {b, c}, {0, -1}, {0, 1}],
  p[[n]] = Show[g,
    AspectRatio -> 1.05,
    PlotRange -> All,
    DisplayFunction -> Identity]},
 {n, 1, 3}]

(* Display Arraylets Correlations *)

Show[GraphicsArray[{p[[1]], p[[2]], p[[3]]}],
  GraphicsSpacing -> -0.11];
```

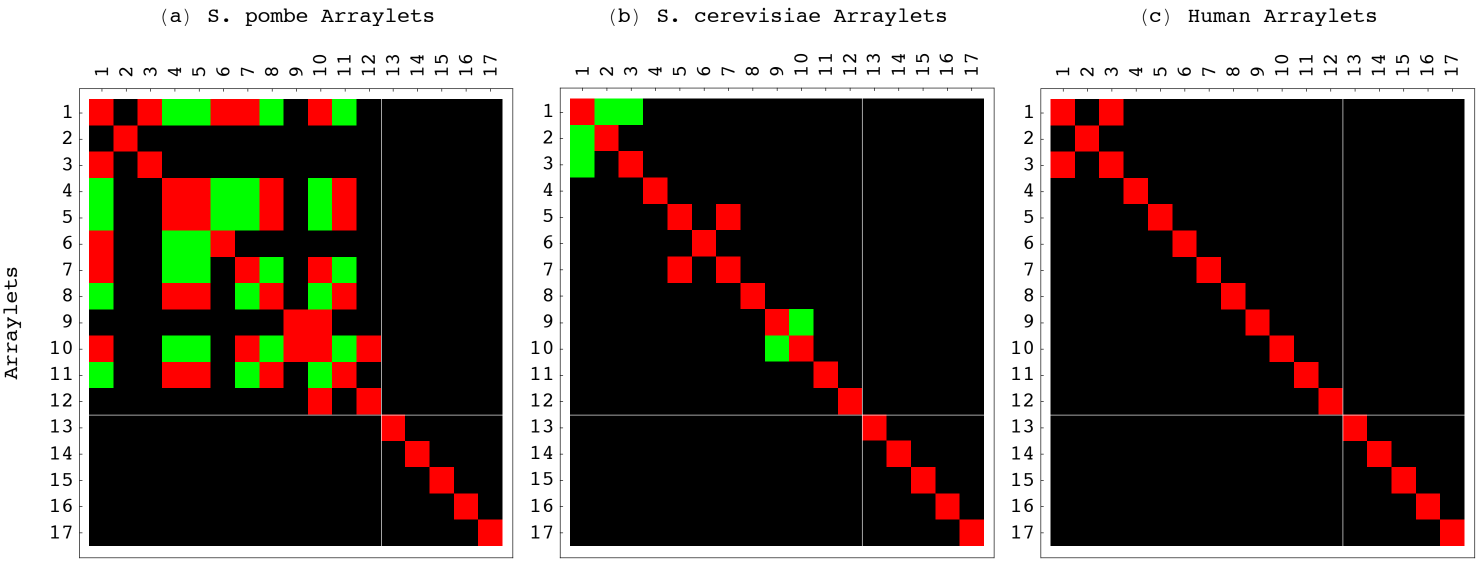

```
(* Create Genelets 2 D Red & Green Raster Display *)

(* Center Genelets *)

average = Table[1, {a, 1, arrays}];
average = N[average / Sqrt[Dot[average, average]]];
centergenelets = genelets - N[Outer[Times, Dot[genelets, average], average]];

arraynames = Table[a, {a, 1, arrays}];
contrast = 3;
displaying = Table[
   If[contrast * centergenelets[[i, j]] > 0,
    If[contrast * centergenelets[[i, j]] < 1, {contrast * centergenelets[[i, j]], 0}, {1, 0}],
    If[contrast * centergenelets[[i, j]] > -1, {0, -contrast * centergenelets[[i, j]]}, {0, 1}]],
   {i, 1, arrays}, {j, 1, arrays}];
framex = Table[{a - 0.5, ToString[a]}, {a, 1, arrays}];
framey = Table[{a + 1 - 0.5, arrays - a}, {a, 0, arrays - 1}];
labely = "Genelets";
labelx = ColumnForm[{"(a)  Arrays", "  "}, Center];
g = Show[
   Graphics[
    RasterArray[
     Table[
      RGBColor[displaying[[i, j, 1]], displaying[[i, j, 2]], 0],
      {i, arrays, 1, -1}, {j, 1, arrays}]]],
   AspectRatio -> 1,
   Frame -> True,
   FrameTicks -> {None, framey, framex, None},
   FrameLabel -> {None, labely, labelx, None},
   DisplayFunction -> Identity];
g = FullGraphics[g];
g[[1, 2]] = g[[1, 2]] /.
   Text[labely, {b_, c_}, {1., 0.}] ->
    Text[labely, {b - 3, c}, {0, 0}, {0, 1}];
g[[1, 2]] = g[[1, 2]] /.
   Text[labelx, {b_, c_}, {0., -1.}] ->
    Text[labelx, {b, c + 2}, {0, -1}, {1, 0}];
g[[1, 2]] = g[[1, 2]] /.
   Text[a_, {b_, c_}, {0., -1.}] ->
    Text[a, {b, c}, {0, -1}, {0, 1}];
g1 = Show[g,
   AspectRatio -> 1.05,
   PlotRange -> All,
   DisplayFunction -> Identity];

(* Create Fractions Bar Charts Displays *)

fractions = 1 / values;
Clear[gridx, framex, framey];
gridx = Table[a, {a, 0, limit, N[limit / 5]}];
framex = gridx;
framex = Table[{gridx[[a]], framex[[a]]}, {a, 1, 6}];
gridx = Table[{gridx[[a]], RGBColor[0, 0, 0]}, {a, 1, 6}];
framey = Table[{a + 1, arrays - a - 6}, {a, 0, 12 - 3}];
table = Table[fractions[[arrays - a]], {a, 6, arrays - 3}];
gridx = Table[a, {a, 0, 1, 0.2}];
framex = gridx;
framex = Table[{gridx[[a]], framex[[a]]}, {a, 1, 6}];
framex[[6]] = {1, "1"};
gridx = Table[{gridx[[a]], RGBColor[0, 0, 0]}, {a, 1, 6}];
framey = Table[{a + 1, arrays - a}, {a, 0, arrays - 1}];
labelx = ColumnForm[
   {"(b)  Inverse Eigenvalues λk^-1", StringJoin["  ", "  "]},
   Center];
```

```
g = BarChart[
   Table[fractions[[arrays - a]], {a, 0, arrays - 1}],
   BarOrientation -> Horizontal,
   PlotRange -> {{0, 1.0001}, {0.5, arrays + 0.5}},
   AspectRatio -> 1,
   Axes -> False,
   Frame -> True,
   FrameTicks -> {None, framey, framex, None},
   FrameLabel -> {None, None, labelx, None},
   GridLines -> {{gridx[[1]], gridx[[2]], gridx[[3]], gridx[[4]], gridx[[5]],
      {0.5, {RGBColor[0, 0, 0], Dashing[{0.03, 0.02}]}}}, {{0, RGBColor[0, 0, 0]}}},
   DisplayFunction -> Identity];
g = FullGraphics[g];
g[[1, 2]] = g[[1, 2]] /.
   Text[labelx, {b_, c_}, {0., -1.}] ->
    Text[labelx, {b, c + 2}, {0, -1}, {1, 0}];
g[[1, 2]] = g[[1, 2]] /.
   Text[a_, {b_, c_}, {0., -1.}] ->
    Text[a, {b, c}, {0, -1}, {0, 1}];
g2 = Show[{g},
   AspectRatio -> 1.35,
   PlotRange -> All,
   DisplayFunction -> Identity];

(* Create Selected Projected Genelets Graph Display *)

{u, w, v} = SingularValueDecomposition[
   {genelets[[13]], genelets[[14]], genelets[[15]], genelets[[16]], genelets[[17]]}];
w[[2, 2]] = 0;
w[[4, 4]] = 0;
w[[5, 5]] = 0;
Pgenelets = Dot[u, w, Transpose[v]];
average = Table[1, {a, 1, arrays}];
average = N[average / Sqrt[Dot[average, average]]];
Pgenelets = Pgenelets - N[Outer[Times, Dot[Pgenelets, average], average]];
Do[Pgenelets[[a]] = Pgenelets[[a]] / Sqrt[Dot[Pgenelets[[a]], Pgenelets[[a]]]], {a, 1, 5}]
Pgenelets13 =  Sqrt[2 / 17.] * Cos[4 * Pi * t / 17 + 9 * Pi / 16];
Pgenelets14 = Sqrt[2 / 17.] * Cos[4 * Pi * t / 17 - Pi / 2];
Pgenelets15 = Sqrt[2 / 17.] * Cos[4 * Pi * t / 17 -  Pi / 4];
Pgenelets16 = Sqrt[2 / 17.] * Cos[4 * Pi * t / 17 + Pi / 2];
Pgenelets17 = Sqrt[2 / 17.] * Cos[4 * Pi * t / 17];

p = Table[0, {5}];
color = {RGBColor[1, 0, 0], RGBColor[0, 0, 1], RGBColor[0, 0.5, 0],
   RGBColor[1, 0.5, 0], RGBColor[0.75, 0, 1]};
fittedPgenelets = {Pgenelets13, Pgenelets14, Pgenelets15, Pgenelets16, Pgenelets17};
```

texts = {"$\sqrt{\frac{2}{T}}\cos(\frac{4\pi t}{T}+\frac{\pi}{4}+\frac{\pi}{16})$", "$\sqrt{\frac{2}{T}}\cos(\frac{4\pi t}{T}-\frac{\pi}{2})$", "$\sqrt{\frac{2}{T}}\cos(\frac{4\pi t}{T}-\frac{\pi}{4})$",
   "$\sqrt{\frac{2}{T}}\cos(\frac{4\pi t}{T}+\frac{\pi}{2})$", "$\sqrt{\frac{2}{T}}\cos(\frac{4\pi t}{T})$"};

```
textpositions = {{6.5, 1.0}, {8.5, -0.675}, {10.5, 0.675}, {8.5, 0.675}, {8.5, -0.675}};
xlabels = {"(c) Arrays", " ", " ", "(d) Arrays", " "};
ylabels = {{"Expression Level"}, {" "}, {" "}, {" "}, {" "}};
framex = Table[{a - 1, ToString[a]}, {a, 1, arrays}];
framey = {-1, -0.5, 0, 0.5, 1};
flag = 0;
```

```
Do[{
  graph = Plot[fittedPgenelets[[n]],
    {t, 0, arrays - 1},
    PlotStyle -> {color[[n]], Dashing[{0.03, 0.02}]},
    DisplayFunction -> Identity],
  labelx = ColumnForm[{xlabels[[n]]}, Center],
  labely = ColumnForm[ylabels[[n]], Center],
  coordinates = Table[{a - 1, Pgenelets[[n, a]]}, {a, 1, arrays}],
  points = Table[Point[coordinates[[a]]], {a, 1, arrays}],
  line = Line[coordinates],
  If[n == 4, flag = 0],
  g = Show[{
     Graphics[{color[[n]], PointSize[0.022], points}],
     Graphics[{color[[n]], line}],
     Graphics[{color[[n]], Text[texts[[n]], textpositions[[n]]]}],
     graph},
    If[flag == 0,
     {FrameLabel -> {None, labely, labelx, None},
      FrameTicks -> {None, framey, framex, None}},
     {FrameLabel -> {None, labely, labelx, None},
      FrameTicks -> {None, None, None, None}}],
    Frame -> True,
    GridLines -> {None, {{0, RGBColor[0, 0, 0]}}},
    PlotRange -> {-0.85, 1.25},
    DisplayFunction -> Identity],
  g = FullGraphics[g],
  g[[1, 2]] = g[[1, 2]] /. Text[labely, {b_, c_}, {1., 0.}] ->
     Text[labely, {b - 3, c}, {0, 0}, {0, 1}],
  g[[1, 2]] = g[[1, 2]] /. Text[labelx, {b_, c_}, {0., -1.}] ->
     Text[labelx, {b, c + 0.4}, {0, -1}, {1, 0}],
  g[[1, 2]] = g[[1, 2]] /. Text[a_, {b_, c_}, {0., -1.}] ->
     Text[a, {b, c}, {0, -1}, {0, 1}],
  flag = flag + 1,
  p[[n]] = Show[g,
    AspectRatio -> 1.05,
    PlotRange -> All,
    DisplayFunction -> Identity]},
 {n, 1, 5}]

(* Display Genelets and Selected Genelets *)

g3 = Show[{p[[1]], p[[2]], p[[3]]}, DisplayFunction -> Identity];
g4 = Show[{p[[4]], p[[5]]}, DisplayFunction -> Identity];
Show[GraphicsArray[{g1, g2, g3, g4}], GraphicsSpacing -> -0.095];
```

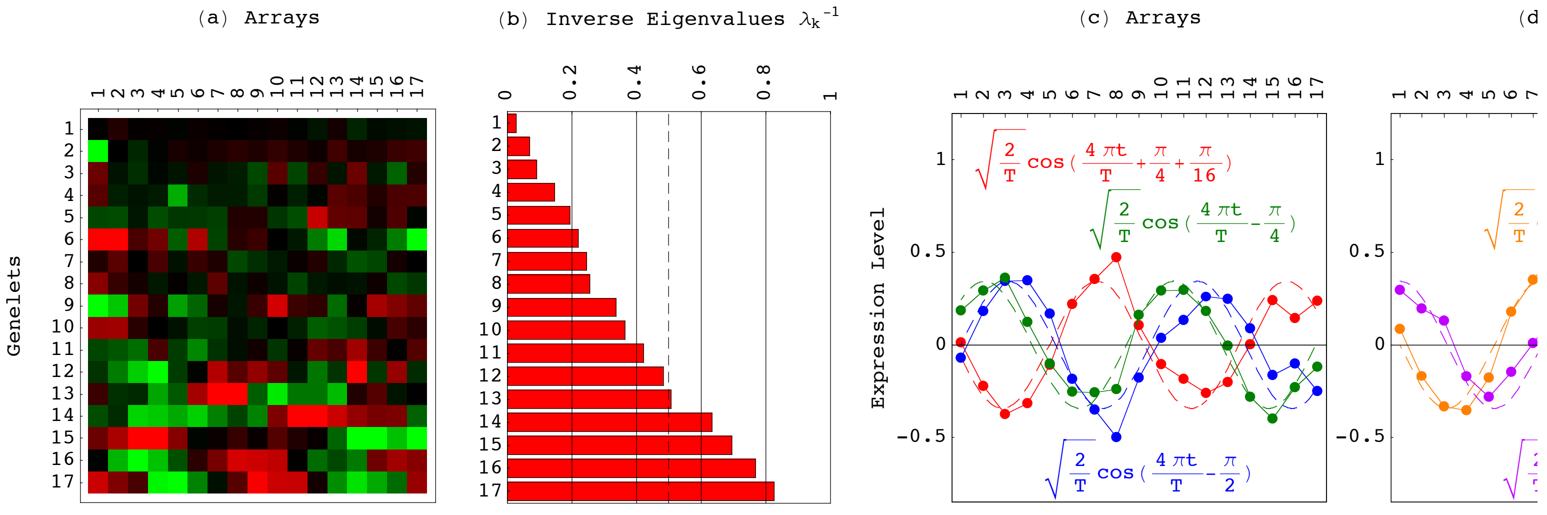

```
(* Estimate Significance of Association of Arraylets with the Cell Cycle *)

(* Calculate Significance of Cell Cycle Associations in S. pombe *)

cutoff = 100;
genes = genes1;
arraynames = arraynames1;
arraylets = arraylets1;

(* Use Rustici et al. or Oliva et al. Classifications of S. pombe Genes *)

genenames = TakeColumns[genenames1, {4}];
stages = {"M", "G1", "S", "G2", "None"};
numbers = Flatten[Table[{Count[Flatten[genenames], stages[[a]]]}, {a, 1, 5}]];
genelet = {{13}, {14}, {15}, {16}, {17}};
probability = Table[{0}, {a, 13, arrays}];
parallelannotation = Table[{0}, {a, 13, arrays}];
parallelprobability = Table[{0}, {a, 13, arrays}];
antiannotation = Table[{0}, {a, 13, arrays}];
antiprobability = Table[{0}, {a, 13, arrays}];

Do[{
  arraylet = TakeColumns[Sort[
     AppendRows[TakeColumns[arraylets, genelet[[c]]], genenames],
     OrderedQ[{{#2}, {#1}}] &], {2}],
  table = Table[{
     stages[[a]],
     numbers[[a]],
     Count[Flatten[TakeRows[arraylet, {1, cutoff}]], stages[[a]]]},
    {a, 1, 5}],
  probability = Table[{
     Sum[N[Binomial[table[[a, 2]], b] * Binomial[genes - table[[a, 2]], cutoff - b] /
         Binomial[genes, cutoff]], {b, table[[a, 3]], cutoff}],
     stages[[a]]},
    {a, 1, 5}],
  parallelannotation[[c]] = {Sort[probability, OrderedQ[{{#1}, {#2}}] &][[1, 2]]},
  parallelprobability[[c]] = {ScientificForm[Sort[probability, OrderedQ[{{#1}, {#2}}] &][[1, 1]], 2]},
  table = Table[{
     stages[[a]],
     numbers[[a]],
     Count[Flatten[TakeRows[arraylet, {genes - (cutoff - 1), genes}]], stages[[a]]]},
    {a, 1, 5}],
  probability = Table[{
     Sum[N[Binomial[table[[a, 2]], b] * Binomial[genes - table[[a, 2]], cutoff - b] /
         Binomial[genes, cutoff]], {b, table[[a, 3]], cutoff}],
     stages[[a]]},
    {a, 1, 5}],
  antiannotation[[c]] = {Sort[probability, OrderedQ[{{#1}, {#2}}] &][[1, 2]]},
  antiprobability[[c]] = {ScientificForm[Sort[probability, OrderedQ[{{#1}, {#2}}] &][[1, 1]], 2]}},
 {c, 1, Dimensions[genelet][[1]]}]

table1 = AppendRows[
   genelet,
   parallelannotation,
   parallelprobability,
   antiannotation,
   antiprobability];
```

```
(* Calculate Significance of Cell Cycle Associations in S. cerevisiae *)

genes = genes2;
arraynames = arraynames2;
arraylets = arraylets2;

(* Use Spellman et al. Classification of S. cerevisiae Genes *)

genenames = TakeColumns[genenames2, {3}];
stages = {"M/G1", "G1", "S", "S/G2", "G2/M", "None"};
numbers = Flatten[Table[{Count[Flatten[genenames], stages[[a]]]}, {a, 1, 6}]];
genelet = {{13}, {14}, {15}, {16}, {17}};
probability = Table[{0}, {a, 13, arrays}];
parallelannotation = Table[{0}, {a, 13, arrays}];
parallelprobability = Table[{0}, {a, 13, arrays}];
antiannotation = Table[{0}, {a, 13, arrays}];
antiprobability = Table[{0}, {a, 13, arrays}];

Do[{
  arraylet = TakeColumns[Sort[
     AppendRows[TakeColumns[arraylets, genelet[[c]]], genenames],
     OrderedQ[{{#2}, {#1}}] &], {2}],
  table = Table[{
     stages[[a]],
     numbers[[a]],
     Count[Flatten[TakeRows[arraylet, {1, cutoff}]], stages[[a]]]},
    {a, 1, 6}],
  probability = Table[{
     Sum[N[Binomial[table[[a, 2]], b] * Binomial[genes - table[[a, 2]], cutoff - b] /
         Binomial[genes, cutoff]], {b, table[[a, 3]], cutoff}],
     stages[[a]]},
    {a, 1, 6}],
  parallelannotation[[c]] = {Sort[probability, OrderedQ[{{#1}, {#2}}] &][[1, 2]]},
  parallelprobability[[c]] = {ScientificForm[Sort[probability, OrderedQ[{{#1}, {#2}}] &][[1, 1]], 2]},
  table = Table[{
     stages[[a]],
     numbers[[a]],
     Count[Flatten[TakeRows[arraylet, {genes - (cutoff - 1), genes}]], stages[[a]]]},
    {a, 1, 6}],
  probability = Table[{
     Sum[N[Binomial[table[[a, 2]], b] * Binomial[genes - table[[a, 2]], cutoff - b] /
         Binomial[genes, cutoff]], {b, table[[a, 3]], cutoff}],
     stages[[a]]},
    {a, 1, 6}],
  antiannotation[[c]] = {Sort[probability, OrderedQ[{{#1}, {#2}}] &][[1, 2]]},
  antiprobability[[c]] = {ScientificForm[Sort[probability, OrderedQ[{{#1}, {#2}}] &][[1, 1]], 2]}},
 {c, 1, Dimensions[genelet][[1]]}]

table2 = AppendRows[
   genelet,
   parallelannotation,
   parallelprobability,
   antiannotation,
   antiprobability];
```

```
(* Calculate Significance of Cell Cycle Associations in Human *)

genes = genes3;
arraynames = arraynames3;
arraylets = arraylets3;

(* Use Whitfield et al. Classification of Human Genes *)

genenames = TakeColumns[genenames3, {3}];
stages = {"M/G1", "G1/S", "S", "G2", "G2/M", "None"};
numbers = Flatten[Table[{Count[Flatten[genenames], stages[[a]]]}, {a, 1, 6}]];
genelet = {{13}, {14}, {15}, {16}, {17}};
probability = Table[{0}, {a, 13, arrays}];
parallelannotation = Table[{0}, {a, 13, arrays}];
parallelprobability = Table[{0}, {a, 13, arrays}];
antiannotation = Table[{0}, {a, 13, arrays}];
antiprobability = Table[{0}, {a, 13, arrays}];

Do[{
  arraylet = TakeColumns[Sort[
     AppendRows[TakeColumns[arraylets, genelet[[c]]], genenames],
     OrderedQ[{{#2}, {#1}}] &], {2}],
  table = Table[{
     stages[[a]],
     numbers[[a]],
     Count[Flatten[TakeRows[arraylet, {1, cutoff}]], stages[[a]]]},
    {a, 1, 6}],
  probability = Table[{
     Sum[N[Binomial[table[[a, 2]], b] * Binomial[genes - table[[a, 2]], cutoff - b] /
         Binomial[genes, cutoff]], {b, table[[a, 3]], cutoff}],
     stages[[a]]},
    {a, 1, 6}],
  parallelannotation[[c]] = {Sort[probability, OrderedQ[{{#1}, {#2}}] &][[1, 2]]},
  parallelprobability[[c]] = {ScientificForm[Sort[probability, OrderedQ[{{#1}, {#2}}] &][[1, 1]], 2]},
  table = Table[{
     stages[[a]],
     numbers[[a]],
     Count[Flatten[TakeRows[arraylet, {genes - (cutoff - 1), genes}]], stages[[a]]]},
    {a, 1, 6}],
  probability = Table[{
     Sum[N[Binomial[table[[a, 2]], b] * Binomial[genes - table[[a, 2]], cutoff - b] /
         Binomial[genes, cutoff]], {b, table[[a, 3]], cutoff}],
     stages[[a]]},
    {a, 1, 6}],
  antiannotation[[c]] = {Sort[probability, OrderedQ[{{#1}, {#2}}] &][[1, 2]]},
  antiprobability[[c]] = {ScientificForm[Sort[probability, OrderedQ[{{#1}, {#2}}] &][[1, 1]], 2]}},
 {c, 1, Dimensions[genelet][[1]]}]

table3 = AppendRows[
   genelet,
   parallelannotation,
   parallelprobability,
   antiannotation,
   antiprobability];
```

```
(* Display Significance of Association of Arraylets with the Cell Cycle *)

headerx = {{
    ColumnForm[{" ", "Dataset"}, Left],
    ColumnForm[{" ", "Arraylet"}, Left],
    ColumnForm[{"Overexpression", "Annotation"}, Left],
    ColumnForm[{"Overexpression", "P-Value"}, Left],
    ColumnForm[{"Underexpression", "Annotation"}, Left],
    ColumnForm[{"Underexpression", "P-Value"}, Left]},
   {" ", " ", " ", " ", " ", " "}};
spacerx = {{" ", " ", " ", " ", " "}};
headery = Table[" ", {a, 1, 17}, {b, 1, 1}];
headery[[1]] = {"S. pombe"};
headery[[7]] = {"S. cerevisiae"};
headery[[13]] = {"Human"};
association =
  AppendColumns[headerx,
   AppendRows[headery,
    AppendColumns[table1, spacerx, table2, spacerx, table3]]];
TableForm[association, TableSpacing -> {1, 1}]
```

Dataset	Arraylet	Overexpression Annotation	Overexpression P-Value	Underexpression Annotation	Underexpression P-Value
S. pombe	13	G2	2.4×10^{-10}	G1	$1.\times10^{-15}$
	14	M	2.2×10^{-21}	G2	1.3×10^{-9}
	15	M	4.1×10^{-13}	S	1.6×10^{-17}
	16	G2	5.2×10^{-18}	G1	1.2×10^{-26}
	17	G2	2.4×10^{-10}	S	5.3×10^{-35}
S. cerevisiae	13	S/G2	4.3×10^{-15}	M/G1	1.4×10^{-9}
	14	M/G1	4.9×10^{-26}	G2/M	2.2×10^{-12}
	15	G1	7.7×10^{-17}	S	1.3×10^{-8}
	16	G2/M	2.3×10^{-38}	G1	$2.\times10^{-32}$
	17	G2/M	2.3×10^{-41}	G1	2.6×10^{-40}
Human	13	G1/S	1.1×10^{-33}	G2	2.4×10^{-44}
	14	M/G1	5.7×10^{-3}	G2	4.7×10^{-2}
	15	G2	9.8×10^{-24}	None	1.4×10^{-1}
	16	G1/S	9.8×10^{-13}	G2	4.1×10^{-4}
	17	G2	9.3×10^{-33}	M/G1	2.7×10^{-2}

```
(* Sort S. pombe, S. cerevisiae and Human Data in 5 D Common Cell Cycle Subspace *)

(* Least-Squares Approximate 5 D Subspace of Genelets with 2 D Subspace Using SVD *)

{u, w, v} = SingularValues[
   {genelets[[13]], genelets[[14]], genelets[[15]], genelets[[16]], genelets[[17]]}];
v[[3]] = -v[[3]];

(* Define 2 D Subspace {x,y} ≡  {v[[3]],v[[1]]} *)

x = v[[3]];
y = v[[1]];

(* Create v[[1]], v[[2]] and v[[3]] Graph Displays *)

average = Table[1, {a, 1, arrays}];
average = N[average / Sqrt[Dot[average, average]]];
v = v - N[Outer[Times, Dot[v, average], average]];

v1 = Sqrt[2 / 17.] * Cos[4 * Pi * t / 17 - Pi / 2];
v2 = Sqrt[2 / 17.] * Cos[2 * Pi * t / 17 - Pi / 3];
v3 = Sqrt[2 / 17.] * Cos[4 * Pi * t / 17];

p = Table[0, {3}];
color = {RGBColor[1, 0, 0], RGBColor[0, 0, 1], RGBColor[0, 0.5, 0]};
fittedv = {v1, v2, v3};
texts = {"Sqrt[2/T] cos(4 πt/T - π/2)", "Sqrt[2/T] cos(2 πt/T - π/3)", "Sqrt[2/T] cos(4 πt/T)"};
textpositions = {{6.5, 1.0}, {8.5, -0.675}, {10.5, 0.675}};
framex = Table[{a - 1, ToString[a]}, {a, 1, arrays}];
framey = {-1, -0.5, 0, 0.5, 1};
labelx = ColumnForm[{"Arrays"}, Center];
labely = ColumnForm[{" ", "Expression Level"}, Center];
flag = 0;
```

```
Do[{
  graph = Plot[fittedv[[n]],
    {t, 0, arrays - 1},
    PlotStyle -> {color[[n]], Dashing[{0.03, 0.02}]},
    DisplayFunction -> Identity],
  coordinates = Table[{a - 1, v[[n, a]]}, {a, 1, arrays}],
  points = Table[Point[coordinates[[a]]], {a, 1, arrays}],
  line = Line[coordinates],
  g = Show[{
     Graphics[{color[[n]], PointSize[0.022], points}],
     Graphics[{color[[n]], line}],
     Graphics[{color[[n]], Text[texts[[n]], textpositions[[n]]]}],
     graph},
    If[flag == 0,
     {FrameLabel -> {None, labely, labelx, None},
      FrameTicks -> {None, framey, framex, None}},
     {FrameLabel -> {None, None, None, None},
      FrameTicks -> {None, None, None, None}}],
    Frame -> True,
    GridLines -> {None, {{0, RGBColor[0, 0, 0]}}},
    PlotRange -> {-0.85, 1.25},
    DisplayFunction -> Identity],
  g = FullGraphics[g],
  g[[1, 2]] = g[[1, 2]] /.
    Text[labely, {b_, c_}, {1., 0.}] ->
     Text[labely, {b - 3, c}, {0, 0}, {0, 1}],
  g[[1, 2]] = g[[1, 2]] /.
    Text[labelx, {b_, c_}, {0., -1.}] ->
     Text[labelx, {b, c + 0.225}, {0, -1}, {1, 0}],
  g[[1, 2]] = g[[1, 2]] /.
    Text[a_, {b_, c_}, {0., -1.}] ->
     Text[a, {b, c}, {0, -1}, {0, 1}],
  flag = flag + 1,
  p[[n]] = Show[g,
    AspectRatio -> 0.95,
    PlotRange -> All,
    DisplayFunction -> Identity]},
 {n, 1, 3}]

(* Display v[[1]], v[[2]] and v[[3]] *)

g = Show[{p[[3]], p[[2]], p[[1]]},
   DisplayFunction -> $DisplayFunction];
```

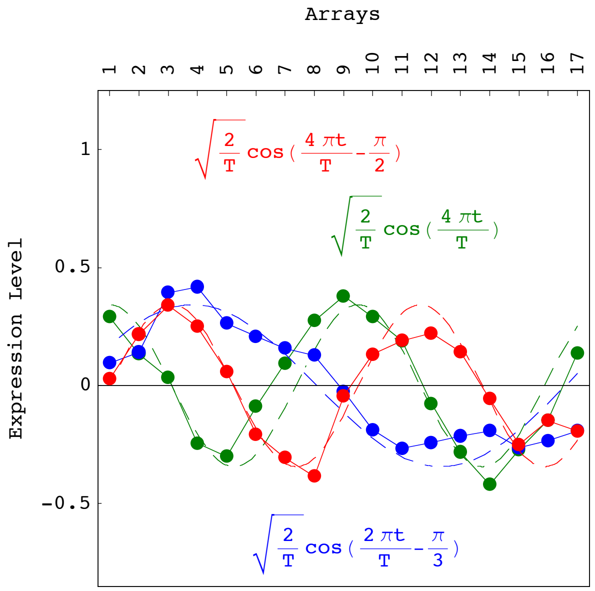

```
(* Calculate Amplitudes of the Projections of the Genelets *)

a13 = Sqrt[Dot[genelets[[13]], x]^2 + Dot[genelets[[13]], y]^2];
a14 = Sqrt[Dot[genelets[[14]], x]^2 + Dot[genelets[[14]], y]^2];
a15 = Sqrt[Dot[genelets[[15]], x]^2 + Dot[genelets[[15]], y]^2];
a16 = Sqrt[Dot[genelets[[16]], x]^2 + Dot[genelets[[16]], y]^2];
a17 = Sqrt[Dot[genelets[[17]], x]^2 + Dot[genelets[[17]], y]^2];

(* Calculate Angular Directions of the Projections of Genelets *)

c13 = ArcTan[Dot[genelets[[13]], x], Dot[genelets[[13]], y]];
c14 = ArcTan[Dot[genelets[[14]], x], Dot[genelets[[14]], y]];
c15 = ArcTan[Dot[genelets[[15]], x], Dot[genelets[[15]], y]];
c16 = ArcTan[Dot[genelets[[16]], x], Dot[genelets[[16]], y]];
c17 = ArcTan[Dot[genelets[[17]], x], Dot[genelets[[17]], y]];

c1314 = 2 * Abs[Cos[c13 - c14]];
c1315 = 2 * Abs[Cos[c13 - c15]];
c1316 = 2 * Abs[Cos[c13 - c16]];
c1317 = 2 * Abs[Cos[c13 - c17]];
c1415 = 2 * Abs[Cos[c14 - c15]];
c1416 = 2 * Abs[Cos[c14 - c16]];
c1417 = 2 * Abs[Cos[c14 - c17]];
c1516 = 2 * Abs[Cos[c15 - c16]];
c1517 = 2 * Abs[Cos[c15 - c17]];
c1617 = 2 * Abs[Cos[c16 - c17]];

(* Sort S. pombe Data *)

genes = genes1;
arraynames = arraynames1;
genenames = genenames1;

(* Sort S. pombe Arrays *)

(* Center Genelets and Calculate Contributions of Arraylets to Arrays *)

average = Table[1, {a, 1, arrays}];
average = N[average / Sqrt[Dot[average, average]]];

arraycontributions13 =
  (genelets[[13]] - N[Outer[Times, Dot[genelets, average], average]][[13]]) * d1[[13, 13]];
arraycontributions14 =
  (genelets[[14]] - N[Outer[Times, Dot[genelets, average], average]][[14]]) * d1[[14, 14]];
arraycontributions15 =
  (genelets[[15]] - N[Outer[Times, Dot[genelets, average], average]][[15]]) * d1[[15, 15]];
arraycontributions16 =
  (genelets[[16]] - N[Outer[Times, Dot[genelets, average], average]][[16]]) * d1[[16, 16]];
arraycontributions17 =
  (genelets[[17]] - N[Outer[Times, Dot[genelets, average], average]][[17]]) * d1[[17, 17]];
```

```
(* Project Arrays from 5 D Arraylets Subspace Onto 2 D Subspace  *)

coordinates = Table[{
    (Dot[genelets[[13]], x] * arraycontributions13[[a]] +
       Dot[genelets[[14]], x] * arraycontributions14[[a]] +
       Dot[genelets[[15]], x] * arraycontributions15[[a]] +
       Dot[genelets[[16]], x] * arraycontributions16[[a]] +
       Dot[genelets[[17]], x] * arraycontributions17[[a]]) /
     Sqrt[(a13 * arraycontributions13[[a]]) ^ 2 + (a14 * arraycontributions14[[a]]) ^ 2 +
       (a15 * arraycontributions15[[a]]) ^ 2 + (a16 * arraycontributions16[[a]]) ^ 2 +
       (a17 * arraycontributions17[[a]]) ^ 2 +
       c1314 * Abs[(a13 * arraycontributions13[[a]]) * (a14 * arraycontributions14[[a]])] +
       c1315 * Abs[(a13 * arraycontributions13[[a]]) * (a15 * arraycontributions15[[a]])] +
       c1316 * Abs[(a13 * arraycontributions13[[a]]) * (a16 * arraycontributions16[[a]])] +
       c1317 * Abs[(a13 * arraycontributions13[[a]]) * (a17 * arraycontributions17[[a]])] +
       c1415 * Abs[(a14 * arraycontributions14[[a]]) * (a15 * arraycontributions15[[a]])] +
       c1416 * Abs[(a14 * arraycontributions14[[a]]) * (a16 * arraycontributions16[[a]])] +
       c1417 * Abs[(a14 * arraycontributions14[[a]]) * (a17 * arraycontributions17[[a]])] +
       c1516 * Abs[(a15 * arraycontributions15[[a]]) * (a16 * arraycontributions16[[a]])] +
       c1517 * Abs[(a15 * arraycontributions15[[a]]) * (a17 * arraycontributions17[[a]])] +
       c1617 * Abs[(a16 * arraycontributions16[[a]]) * (a17 * arraycontributions17[[a]])]],
    (Dot[genelets[[13]], y] * arraycontributions13[[a]] +
       Dot[genelets[[14]], y] * arraycontributions14[[a]] +
       Dot[genelets[[15]], y] * arraycontributions15[[a]] +
       Dot[genelets[[16]], y] * arraycontributions16[[a]] +
       Dot[genelets[[17]], y] * arraycontributions17[[a]]) /
     Sqrt[(a13 * arraycontributions13[[a]]) ^ 2 + (a14 * arraycontributions14[[a]]) ^ 2 +
       (a15 * arraycontributions15[[a]]) ^ 2 + (a16 * arraycontributions16[[a]]) ^ 2 +
       (a17 * arraycontributions17[[a]]) ^ 2 +
       c1314 * Abs[(a13 * arraycontributions13[[a]]) * (a14 * arraycontributions14[[a]])] +
       c1315 * Abs[(a13 * arraycontributions13[[a]]) * (a15 * arraycontributions15[[a]])] +
       c1316 * Abs[(a13 * arraycontributions13[[a]]) * (a16 * arraycontributions16[[a]])] +
       c1317 * Abs[(a13 * arraycontributions13[[a]]) * (a17 * arraycontributions17[[a]])] +
       c1415 * Abs[(a14 * arraycontributions14[[a]]) * (a15 * arraycontributions15[[a]])] +
       c1416 * Abs[(a14 * arraycontributions14[[a]]) * (a16 * arraycontributions16[[a]])] +
       c1417 * Abs[(a14 * arraycontributions14[[a]]) * (a17 * arraycontributions17[[a]])] +
       c1516 * Abs[(a15 * arraycontributions15[[a]]) * (a16 * arraycontributions16[[a]])] +
       c1517 * Abs[(a15 * arraycontributions15[[a]]) * (a17 * arraycontributions17[[a]])] +
       c1617 * Abs[(a16 * arraycontributions16[[a]]) * (a17 * arraycontributions17[[a]])]]},
   {a, 1, arrays}];

(* Create Parametric Plot of S. pombe Arrays Projected Onto 2 D Subspace *)

Clear[points];
points1 = {Point[coordinates[[7]]], Point[coordinates[[8]]], Point[coordinates[[9]]],
   Point[coordinates[[10]]], Point[coordinates[[16]]], Point[coordinates[[17]]]};
points2 = {Point[coordinates[[2]]], Point[coordinates[[11]]]};
points3 = {Point[coordinates[[1]]], Point[coordinates[[3]]], Point[coordinates[[4]]],
   Point[coordinates[[5]]], Point[coordinates[[12]]], Point[coordinates[[13]]]};
points4 = {Point[coordinates[[6]]], Point[coordinates[[14]]], Point[coordinates[[15]]]};
textcoordinates = coordinates;
Do[
  textcoordinates[[a, 1]] = If[
    textcoordinates[[a, 1]] > 0,
    textcoordinates[[a, 1]] + 0.095,
    textcoordinates[[a, 1]] - 0.1],
  {a, 1, 9}];
Do[
  textcoordinates[[a, 1]] =
   If[textcoordinates[[a, 1]] > 0,
    textcoordinates[[a, 1]] + 0.11,
    textcoordinates[[a, 1]] - 0.12],
  {a, 10, arrays}];
```

```
textcoordinates[[1]] = textcoordinates[[1]] - {0.18, 0};
textcoordinates[[6]] = textcoordinates[[6]] + {0.2, 0};
textcoordinates[[11]] = textcoordinates[[11]] - {0.22, 0};
textcoordinates[[12]] = textcoordinates[[12]] + {0.12, -0.12};
textcoordinates[[15]] = textcoordinates[[15]] + {0, 0.05};
textcoordinates[[16]] = textcoordinates[[16]] + {0.24, 0};
textcoordinates[[17]] = textcoordinates[[17]] + {0, 0.1};

texts = Table[Text[a, textcoordinates[[a]]], {a, 1, arrays}];
radius = Sqrt[coordinates[[2, 1]]^2 + coordinates[[2, 2]]^2];
p = Show[{
    Graphics[{RGBColor[0, 0, 0], Text["(a)", {-1.22, 1.15}, {-1, 0}]}],
    Graphics[{RGBColor[0, 0, 0], Text["x", {1.22, -0.075}, {1, 0}]}],
    Graphics[{RGBColor[0, 0, 0], Text["y", {-0.065, 1.22}, {0, 1}]}],
    Graphics[{RGBColor[1, 0, 0], Text["G2", {1.01, -0.54}]}],
    Graphics[{RGBColor[0, 0, 0], Text["M", {0.25, 1.07}]}],
    Graphics[{RGBColor[0, 0.5, 0], Text["G1", {-1, 0.525}]}],
    Graphics[{RGBColor[0, 0, 1], Text["S", {-0.878, -0.71}]}],
    Graphics[{RGBColor[1, 0, 0], PointSize[0.035], points1}],
    Graphics[{RGBColor[1, 1, 0], PointSize[0.035], points2}],
    Graphics[{RGBColor[0, 0.5, 0], PointSize[0.035], points3}],
    Graphics[{RGBColor[0, 0, 1], PointSize[0.035], points4}],
    Graphics[{RGBColor[0, 0, 0], Text["u1,17", {1.235, 0.5}, {1, 0}]}],
    Graphics[{RGBColor[0, 0, 0], Text["u1,16", {0.5, -1.12}, {-1, 0}]}],
    Graphics[{RGBColor[0, 0, 0], Text["u1,15", {0.7, 1.12}, {-1, 0}]}],
    Graphics[{RGBColor[0, 0, 0], Text["u1,14", {-0.45, 1.12}, {1, 0}]}],
    Graphics[{RGBColor[0, 0, 0], Text["u1,13", {-0.025, -1.12}, {1, 0}]}],
    Graphics[texts],
    Graphics[{RGBColor[0, 0, 0], Dashing[{0.03, 0.02}], Circle[{0, 0}, 0.5]}],
    Graphics[{RGBColor[0, 0, 0], Dashing[{0.03, 0.02}], Circle[{0, 0}, 1]}],
    Graphics[{RGBColor[0, 0, 0], Dashing[{0.03, 0.02}], Line[{{0, 1.25}, {0, -1.25}}]}],
    Graphics[{RGBColor[0, 0, 0], Dashing[{0.03, 0.02}], Line[{{1.25, 0}, {-1.25, 0}}]}],
    Graphics[{RGBColor[0, 0, 0], Arrow[{1.25 / Tan[c13], 1.25}, {-1.25 / Tan[c13], -1.25},
       HeadCenter -> 0.5, HeadLength -> 0.035, HeadWidth -> 0.75]}],
    Graphics[{RGBColor[0, 0, 0], Arrow[{-1.25 / Tan[c14 - Pi], -1.25}, {1.25 / Tan[c14 - Pi], 1.25},
       HeadCenter -> 0.5, HeadLength -> 0.035, HeadWidth -> 0.75]}],
    Graphics[{RGBColor[0, 0, 0], Arrow[{-1.25 / Tan[c15], -1.25}, {1.25 / Tan[c15], 1.25},
       HeadCenter -> 0.5, HeadLength -> 0.035, HeadWidth -> 0.75]}],
    Graphics[{RGBColor[0, 0, 0], Arrow[{1.25 / Tan[c16], 1.25}, {-1.25 / Tan[c16], -1.25},
       HeadCenter -> 0.5, HeadLength -> 0.035, HeadWidth -> 0.75]}],
    Graphics[{RGBColor[0, 0, 0], Arrow[{-1.25, -1.25 * Tan[c17]}, {1.25, 1.25 * Tan[c17]},
       HeadCenter -> 0.5, HeadLength -> 0.035, HeadWidth -> 0.75]}]},
   AspectRatio -> 1,
   PlotRange -> {{-1.25, 1.25}, {-1.25, 1.25}},
   Frame -> True,
   FrameTicks -> False,
   FrameLabel -> {None, None, None, None},
   GridLines -> {None, None},
   DisplayFunction -> Identity];

p = FullGraphics[p];
p[[1, 2]] = p[[1, 2]] /.
   Text[labely, {b_, c_}, {1., 0.}] ->
    Text[labely, {-1.18, 0}, {0, 0}, {0, 1}];
g1 = Show[p,
   AspectRatio -> 1,
   PlotRange -> All,
   DisplayFunction -> Identity];
```

```
(* Sort S. pombe Genes *)

(* Center Arraylets and Calculate Contributions of Genelets to Genes *)

centerarraylets = Transpose[arraylets1];
average = Table[1, {a, 1, genes}];
average = N[average / Sqrt[Dot[average, average]]];
centerarraylets = centerarraylets - N[Outer[Times, Dot[centerarraylets, average], average]];
centerarraylets = Transpose[centerarraylets];
genecontributions = Transpose[Dot[centerarraylets, d1]];

(* Project Genes from 5 D Genelets Subspace Onto 2 D Subspace  *)

coordinates = Table[{
    (Dot[genelets[[13]], x] * genecontributions[[13, a]] +
       Dot[genelets[[14]], x] * genecontributions[[14, a]] +
       Dot[genelets[[15]], x] * genecontributions[[15, a]] +
       Dot[genelets[[16]], x] * genecontributions[[16, a]] +
       Dot[genelets[[17]], x] * genecontributions[[17, a]]) /
     Sqrt[(a13 * genecontributions[[13, a]])^2 + (a14 * genecontributions[[14, a]])^2 +
       (a15 * genecontributions[[15, a]])^2 + (a16 * genecontributions[[16, a]])^2 +
       (a17 * genecontributions[[17, a]])^2 +
       c1314 * Abs[(a13 * genecontributions[[13, a]]) * (a14 * genecontributions[[14, a]])] +
       c1315 * Abs[(a13 * genecontributions[[13, a]]) * (a15 * genecontributions[[15, a]])] +
       c1316 * Abs[(a13 * genecontributions[[13, a]]) * (a16 * genecontributions[[16, a]])] +
       c1317 * Abs[(a13 * genecontributions[[13, a]]) * (a17 * genecontributions[[17, a]])] +
       c1415 * Abs[(a14 * genecontributions[[14, a]]) * (a15 * genecontributions[[15, a]])] +
       c1416 * Abs[(a14 * genecontributions[[14, a]]) * (a16 * genecontributions[[16, a]])] +
       c1417 * Abs[(a14 * genecontributions[[14, a]]) * (a17 * genecontributions[[17, a]])] +
       c1516 * Abs[(a15 * genecontributions[[15, a]]) * (a16 * genecontributions[[16, a]])] +
       c1517 * Abs[(a15 * genecontributions[[15, a]]) * (a17 * genecontributions[[17, a]])] +
       c1617 * Abs[(a16 * genecontributions[[16, a]]) * (a17 * genecontributions[[17, a]])]],
    (Dot[genelets[[13]], y] * genecontributions[[13, a]] +
       Dot[genelets[[14]], y] * genecontributions[[14, a]] +
       Dot[genelets[[15]], y] * genecontributions[[15, a]] +
       Dot[genelets[[16]], y] * genecontributions[[16, a]] +
       Dot[genelets[[17]], y] * genecontributions[[17, a]]) /
     Sqrt[(a13 * genecontributions[[13, a]])^2 + (a14 * genecontributions[[14, a]])^2 +
       (a15 * genecontributions[[15, a]])^2 + (a16 * genecontributions[[16, a]])^2 +
       (a17 * genecontributions[[17, a]])^2 +
       c1314 * Abs[(a13 * genecontributions[[13, a]]) * (a14 * genecontributions[[14, a]])] +
       c1315 * Abs[(a13 * genecontributions[[13, a]]) * (a15 * genecontributions[[15, a]])] +
       c1316 * Abs[(a13 * genecontributions[[13, a]]) * (a16 * genecontributions[[16, a]])] +
       c1317 * Abs[(a13 * genecontributions[[13, a]]) * (a17 * genecontributions[[17, a]])] +
       c1415 * Abs[(a14 * genecontributions[[14, a]]) * (a15 * genecontributions[[15, a]])] +
       c1416 * Abs[(a14 * genecontributions[[14, a]]) * (a16 * genecontributions[[16, a]])] +
       c1417 * Abs[(a14 * genecontributions[[14, a]]) * (a17 * genecontributions[[17, a]])] +
       c1516 * Abs[(a15 * genecontributions[[15, a]]) * (a16 * genecontributions[[16, a]])] +
       c1517 * Abs[(a15 * genecontributions[[15, a]]) * (a17 * genecontributions[[17, a]])] +
       c1617 * Abs[(a16 * genecontributions[[16, a]]) * (a17 * genecontributions[[17, a]])]]},
   {a, 1, genes}];

(* Create Parametric Plot of S. pombe Cell Cycle Genes Projected Onto 2 D Subspace *)

list = AppendRows[TakeColumns[genenames, {1, 1}], TakeColumns[genenames, {4, 4}]];
stages = {"G2", "M", "G1", "S"};
points = {points1, points2, points3, points4};
radii = {radii1, radii2, radii3, radii4};
```

```
Do[{
  position = Position[list, stages[[b]]],
  points[[b]] = Table[Point[coordinates[[position[[a]][[1]]]]], {a, 1, Dimensions[position][[1]]}],
  radii[[b]] =
   Table[Sqrt[coordinates[[position[[a]][[1]]]][[1]]^2 + coordinates[[position[[a]][[1]]]][[2]]^2],
    {a, 1, Dimensions[position][[1]]}]},
 {b, 1, Dimensions[stages][[1]]}]
radii = Sort[Flatten[radii]];
N[Round[radii[[37]] * 100] / 100]
N[Round[radii[[38]] * 100] / 100]
```

```
0.49
```

```
0.5
```

```
p = Show[{
    Graphics[{RGBColor[0, 0, 0], Text["(d)", {-1.22, 1.15}, {-1, 0}]}],
    Graphics[{RGBColor[0, 0, 0], Text["x", {1.22, -0.075}, {1, 0}]}],
    Graphics[{RGBColor[0, 0, 0], Text["y", {-0.065, 1.22}, {0, 1}]}],
    Graphics[{RGBColor[1, 0, 0], Text["G2", {1.01, -0.54}]}],
    Graphics[{RGBColor[0, 0, 0], Text["M", {0.25, 1.07}]}],
    Graphics[{RGBColor[0, 0.5, 0], Text["G1", {-1, 0.525}]}],
    Graphics[{RGBColor[0, 0, 1], Text["S", {-0.878, -0.71}]}],
    Graphics[{RGBColor[1, 0, 0], PointSize[0.02], points[[1]]}],
    Graphics[{RGBColor[1, 1, 0], PointSize[0.02], points[[2]]}],
    Graphics[{RGBColor[0, 0, 1], PointSize[0.02], points[[4]]}],
    Graphics[{RGBColor[0, 0.5, 0], PointSize[0.02], points[[3]]}],
    Graphics[{RGBColor[0, 0, 0], Text["v17", {1.235, 0.5}, {1, 0}]}],
    Graphics[{RGBColor[0, 0, 0], Text["v16", {0.5, -1.12}, {-1, 0}]}],
    Graphics[{RGBColor[0, 0, 0], Text["v15", {0.7, 1.12}, {-1, 0}]}],
    Graphics[{RGBColor[0, 0, 0], Text["v14", {-0.45, 1.12}, {1, 0}]}],
    Graphics[{RGBColor[0, 0, 0], Text["v13", {-0.025, -1.12}, {1, 0}]}],
    Graphics[{RGBColor[0, 0, 0], Dashing[{0.03, 0.02}], Circle[{0, 0}, 0.5, {0 * Pi, 2 * Pi}]}],
    Graphics[{RGBColor[0, 0, 0], Dashing[{0.03, 0.02}], Circle[{0, 0}, 1]}],
    Graphics[{RGBColor[0, 0, 0], Dashing[{0.03, 0.02}], Line[{{0, 1.25}, {0, -1.25}}]}],
    Graphics[{RGBColor[0, 0, 0], Dashing[{0.03, 0.02}], Line[{{1.25, 0}, {-1.25, 0}}]}],
    Graphics[{RGBColor[0, 0, 0], Arrow[{1.25 / Tan[c13], 1.25}, {-1.25 / Tan[c13], -1.25},
       HeadCenter -> 0.5, HeadLength -> 0.035, HeadWidth -> 0.75]}],
    Graphics[{RGBColor[0, 0, 0], Arrow[{-1.25 / Tan[c14 - Pi], -1.25}, {1.25 / Tan[c14 - Pi], 1.25},
       HeadCenter -> 0.5, HeadLength -> 0.035, HeadWidth -> 0.75]}],
    Graphics[{RGBColor[0, 0, 0], Arrow[{-1.25 / Tan[c15], -1.25}, {1.25 / Tan[c15], 1.25},
       HeadCenter -> 0.5, HeadLength -> 0.035, HeadWidth -> 0.75]}],
    Graphics[{RGBColor[0, 0, 0], Arrow[{1.25 / Tan[c16], 1.25}, {-1.25 / Tan[c16], -1.25},
       HeadCenter -> 0.5, HeadLength -> 0.035, HeadWidth -> 0.75]}],
    Graphics[{RGBColor[0, 0, 0], Arrow[{-1.25, -1.25 * Tan[c17]}, {1.25, 1.25 * Tan[c17]},
       HeadCenter -> 0.5, HeadLength -> 0.035, HeadWidth -> 0.75]}]},
   AspectRatio -> 1,
   PlotRange -> {{-1.25, 1.25}, {-1.25, 1.25}},
   Frame -> True,
   FrameTicks -> False,
   FrameLabel -> {None, None, None, None},
   GridLines -> {None, None},
   DisplayFunction -> Identity];
p = FullGraphics[p];
p[[1, 2]] = p[[1, 2]] /.
   Text[labely, {b_, c_}, {1., 0.}] ->
    Text[labely, {-1.18, 0}, {0, 0}, {0, 1}];
g2 = Show[p,
   AspectRatio -> 1,
   PlotRange -> All,
   DisplayFunction -> Identity];
```

```
R[theta_] = {{Cos[theta], Sin[theta]}, {-Sin[theta], Cos[theta]}};
z = Transpose[{{0.4, 0}, {1.1, 0}, {0.75, 0.25}}];
polypoints[theta_] = Transpose[Dot[R[theta], z]];
p = Show[{
    Graphics[{RGBColor[0, 0, 0], Text["(g)", {-1.22, 1.15}, {-1, 0}]}],
    Graphics[{RGBColor[0, 0, 0], Text["x", {1.22, -0.075}, {1, 0}]}],
    Graphics[{RGBColor[0, 0, 0], Text["y", {-0.065, 1.22}, {0, 1}]}],
    Graphics[{RGBColor[1, 0, 0], Text["G2", {1.01, -0.54}]}],
    Graphics[{RGBColor[0, 0, 0], Text["M", {0.25, 1.07}]}],
    Graphics[{RGBColor[0, 0.5, 0], Text["G1", {-1, 0.525}]}],
    Graphics[{RGBColor[0, 0, 1], Text["S", {-0.878, -0.71}]}],
    Graphics[{RGBColor[0, 0, 0], Text["17", {1.235, 0.5}, {1, 0}]}],
    Graphics[{RGBColor[0, 0, 0], Text["16", {0.5, -1.12}, {-1, 0}]}],
    Graphics[{RGBColor[0, 0, 0], Text["15", {0.7, 1.12}, {-1, 0}]}],
    Graphics[{RGBColor[0, 0, 0], Text["14", {-0.45, 1.12}, {1, 0}]}],
    Graphics[{RGBColor[0, 0, 0], Text["13", {-0.025, -1.12}, {1, 0}]}],
    Graphics[{RGBColor[1, 0, 0], Disk[{0, 0}, 1, {0, c15}]}],
    Graphics[{RGBColor[1, 0, 0], Disk[{0, 0}, 1, {c15 + Pi, 2 * Pi}]}],
    Graphics[{RGBColor[1, 1, 0], Disk[{0, 0}, 1, {c15, c14}]}],
    Graphics[{RGBColor[0, 0.5, 0], Disk[{0, 0}, 1, {c14, Pi + c17}]}],
    Graphics[{RGBColor[0, 0, 1], Disk[{0, 0}, 1, {Pi + c17, c15 + Pi}]}],
    Graphics[{RGBColor[1, 1, 1], Disk[{0, 0}, 0.5]}],
    Graphics[{RGBColor[1, 0, 0], Polygon[polypoints[2 * Pi - c15]]}],
    Graphics[{RGBColor[1, 1, 0], Polygon[polypoints[2 * Pi - c14]]}],
    Graphics[{RGBColor[0, 0.5, 0], Polygon[polypoints[Pi - c17]]}],
    Graphics[{RGBColor[0, 0, 1], Polygon[polypoints[Pi - c15]]}],
    Graphics[{RGBColor[0, 0, 0], Dashing[{0.03, 0.02}], Line[{{0, 1.25}, {0, -1.25}}]}],
    Graphics[{RGBColor[0, 0, 0], Dashing[{0.03, 0.02}], Line[{{1.25, 0}, {-1.25, 0}}]}],
    Graphics[{RGBColor[0, 0, 0], Arrow[{1.25 / Tan[c13], 1.25}, {-1.25 / Tan[c13], -1.25},
       HeadCenter -> 0.5, HeadLength -> 0.035, HeadWidth -> 0.75]}],
    Graphics[{RGBColor[0, 0, 0], Arrow[{-1.25 / Tan[c14 - Pi], -1.25}, {1.25 / Tan[c14 - Pi], 1.25},
       HeadCenter -> 0.5, HeadLength -> 0.035, HeadWidth -> 0.75]}],
    Graphics[{RGBColor[0, 0, 0], Arrow[{-1.25 / Tan[c15], -1.25}, {1.25 / Tan[c15], 1.25},
       HeadCenter -> 0.5, HeadLength -> 0.035, HeadWidth -> 0.75]}],
    Graphics[{RGBColor[0, 0, 0], Arrow[{1.25 / Tan[c16], 1.25}, {-1.25 / Tan[c16], -1.25},
       HeadCenter -> 0.5, HeadLength -> 0.035, HeadWidth -> 0.75]}],
    Graphics[{RGBColor[0, 0, 0], Arrow[{-1.25, -1.25 * Tan[c17]}, {1.25, 1.25 * Tan[c17]},
       HeadCenter -> 0.5, HeadLength -> 0.035, HeadWidth -> 0.75]}]},
   AspectRatio -> 1,
   PlotRange -> {{-1.25, 1.25}, {-1.25, 1.25}},
   Frame -> True,
   FrameTicks -> False,
   FrameLabel -> {None, None, None, None},
   GridLines -> {None, None},
   DisplayFunction -> Identity];
p = FullGraphics[p];
p[[1, 2]] = p[[1, 2]] /.
   Text[labely, {b_, c_}, {1., 0.}] ->
    Text[labely, {-1.18, 0}, {0, 0}, {0, 1}];
g3 = Show[p,
   AspectRatio -> 1,
   PlotRange -> All,
   DisplayFunction -> Identity];
Clear[z];
```

```
(* BFR1 Projected Onto 2 D Subspace *)

stages = {"G2", "M", "G1", "S"};
BFR1a = coordinates[[Position[genenames, "SPCC18B5.01C"][[1, 1]]]];
BFR1b = coordinates[[Position[genenames, "SPCC18B5.01C"][[2, 1]]]];

p = Show[{
    Graphics[{RGBColor[0, 0, 0], Text["(a)", {-1, 1.05}]}],
    Graphics[{RGBColor[0, 0, 0], Text["S. pombe", {1.1, -1.05}, {1, 0}]}],
    Graphics[{RGBColor[0, 0, 0], Text["x", {1.13, -0.07}, {1, 0}]}],
    Graphics[{RGBColor[0, 0, 0], Text["y", {-0.055, 1.145}, {0, 1}]}],
    Graphics[{RGBColor[1, 0, 0], Text["G2", {1.01, -0.54}]}],
    Graphics[{RGBColor[0, 0, 0], Text["M", {0.25, 1.07}]}],
    Graphics[{RGBColor[0, 0.5, 0], Text["G1", {-1, 0.525}]}],
    Graphics[{RGBColor[0, 0, 1], Text["S", {-0.878, -0.71}]}],
    Graphics[{RGBColor[1, 0, 0], Dashing[{0.03, 0.02}],
      {Thickness[0.009], Circle[{0, 0}, 1, {0, c15}]}}],
    Graphics[
     {RGBColor[1, 0, 0], Dashing[{0.03, 0.02}], {Thickness[0.009], Circle[{0, 0}, 1, {c15 + Pi, 2 * Pi}]}}],
    Graphics[{RGBColor[1, 1, 0], Dashing[{0.03, 0.02}],
      {Thickness[0.009], Circle[{0, 0}, 1, {c15, c14}]}}],
    Graphics[
     {RGBColor[0, 0.5, 0], Dashing[{0.03, 0.02}], {Thickness[0.009], Circle[{0, 0}, 1, {c14, Pi + c17}]}}],
    Graphics[{RGBColor[0, 0, 1], Dashing[{0.03, 0.02}],
      {Thickness[0.009], Circle[{0, 0}, 1, {Pi + c17, c15 + Pi}]}}],
    Graphics[{RGBColor[1, 0, 0], Dashing[{0.03, 0.02}],
      {Thickness[0.009], Circle[{0, 0}, 0.5, {0, c15}]}}],
    Graphics[
     {RGBColor[1, 0, 0], Dashing[{0.03, 0.02}], {Thickness[0.009], Circle[{0, 0}, 0.5, {c15 + Pi, 2 * Pi}]}}],
    Graphics[{RGBColor[1, 1, 0], Dashing[{0.03, 0.02}],
      {Thickness[0.009], Circle[{0, 0}, 0.5, {c15, c14}]}}],
    Graphics[{RGBColor[0, 0.5, 0], Dashing[{0.03, 0.02}],
      {Thickness[0.009], Circle[{0, 0}, 0.5, {c14, Pi + c17}]}}],
    Graphics[{RGBColor[0, 0, 1], Dashing[{0.03, 0.02}],
      {Thickness[0.009], Circle[{0, 0}, 0.5, {Pi + c17, c15 + Pi}]}}],
    Graphics[{RGBColor[1, 0, 0], PointSize[0.03], Point[BFR1a]}],
    Graphics[{RGBColor[1, 0, 0], PointSize[0.03], Point[BFR1b]}],
    Graphics[{RGBColor[1, 0, 0], Text["BFR1", BFR1a + {-0.05, -0.12}]}],
    Graphics[{RGBColor[1, 0, 0], Text["BFR1", BFR1b + {-0.17, 0}]}],
    Graphics[{RGBColor[0, 0, 0], Dashing[{0.03, 0.02}], Line[{{0, 1.15}, {0, -1.15}}]}],
    Graphics[{RGBColor[0, 0, 0], Dashing[{0.03, 0.02}], Line[{{1.15, 0}, {-1.15, 0}}]}]},
   AspectRatio -> 1,
   PlotRange -> {{-1.15, 1.15}, {-1.15, 1.15}},
   Frame -> True,
   FrameTicks -> False,
   FrameLabel -> {None, None, None, None},
   GridLines -> {None, None},
   DisplayFunction -> Identity];
p = FullGraphics[p];
p[[1, 2]] = p[[1, 2]] /.
   Text[labely, {b_, c_}, {1., 0.}] ->
    Text[labely, {-1.18, 0}, {0, 0}, {0, 1}];
O1 = Show[p,
   AspectRatio -> 1,
   PlotRange -> All,
   DisplayFunction -> Identity];
```

```
(* SPAC1786 .02 and SPAC977 .09 c Projected Onto 2 D Subspace *)

stages = {"G2", "M", "G1", "S"};
C1786a = coordinates[[Position[genenames, "SPAC1786.02"][[1, 1]]]];
C1786b = coordinates[[Position[genenames, "SPAC1786.02"][[2, 1]]]];
C977 = coordinates[[Position[genenames, "SPAC977.09C"][[1, 1]]]];

p = Show[{
    Graphics[{RGBColor[0, 0, 0], Text["(c)", {-1, 1.05}]}],
    Graphics[{RGBColor[0, 0, 0], Text["S. pombe", {1.1, -1.05}, {1, 0}]}],
    Graphics[{RGBColor[0, 0, 0], Text["x", {1.13, -0.07}, {1, 0}]}],
    Graphics[{RGBColor[0, 0, 0], Text["y", {-0.055, 1.145}, {0, 1}]}],
    Graphics[{RGBColor[1, 0, 0], Text["G2", {1.01, -0.54}]}],
    Graphics[{RGBColor[0, 0, 0], Text["M", {0.25, 1.07}]}],
    Graphics[{RGBColor[0, 0.5, 0], Text["G1", {-1, 0.525}]}],
    Graphics[{RGBColor[0, 0, 1], Text["S", {-0.878, -0.71}]}],
    Graphics[{RGBColor[1, 0, 0], Dashing[{0.03, 0.02}],
      {Thickness[0.009], Circle[{0, 0}, 1, {0, c15}]}}],
    Graphics[{RGBColor[1, 0, 0], Dashing[{0.03, 0.02}],
      {Thickness[0.009], Circle[{0, 0}, 1, {c15 + Pi, 2 * Pi}]}}],
    Graphics[{RGBColor[1, 1, 0], Dashing[{0.03, 0.02}],
      {Thickness[0.009], Circle[{0, 0}, 1, {c15, c14}]}}],
    Graphics[{RGBColor[0, 0.5, 0], Dashing[{0.03, 0.02}],
      {Thickness[0.009], Circle[{0, 0}, 1, {c14, Pi + c17}]}}],
    Graphics[{RGBColor[0, 0, 1], Dashing[{0.03, 0.02}],
      {Thickness[0.009], Circle[{0, 0}, 1, {Pi + c17, c15 + Pi}]}}],
    Graphics[{RGBColor[1, 0, 0], Dashing[{0.03, 0.02}],
      {Thickness[0.009], Circle[{0, 0}, 0.5, {0, c15}]}}],
    Graphics[{RGBColor[1, 0, 0], Dashing[{0.03, 0.02}],
      {Thickness[0.009], Circle[{0, 0}, 0.5, {c15 + Pi, 2 * Pi}]}}],
    Graphics[{RGBColor[1, 1, 0], Dashing[{0.03, 0.02}],
      {Thickness[0.009], Circle[{0, 0}, 0.5, {c15, c14}]}}],
    Graphics[{RGBColor[0, 0.5, 0], Dashing[{0.03, 0.02}],
      {Thickness[0.009], Circle[{0, 0}, 0.5, {c14, Pi + c17}]}}],
    Graphics[{RGBColor[0, 0, 1], Dashing[{0.03, 0.02}],
      {Thickness[0.009], Circle[{0, 0}, 0.5, {Pi + c17, c15 + Pi}]}}],
    Graphics[{RGBColor[1, 0, 0], PointSize[0.03], Point[C1786a]}],
    Graphics[{RGBColor[1, 0, 0], PointSize[0.03], Point[C1786b]}],
    Graphics[{RGBColor[1, 0, 0], Text["C1786.02", C1786a + {-0.05, -0.1}]}],
    Graphics[{RGBColor[1, 0, 0], Text["C1786.02", C1786b + {-0.03, -0.1}]}],
    Graphics[{RGBColor[0, 0, 1], PointSize[0.03], Point[C977]}],
    Graphics[{RGBColor[0, 0, 1], Text["C977.09C", C977 + {0.35, 0}]}],
    Graphics[{RGBColor[0, 0, 0], Dashing[{0.03, 0.02}], Line[{{0, 1.15}, {0, -1.15}}]}],
    Graphics[{RGBColor[0, 0, 0], Dashing[{0.03, 0.02}], Line[{{1.15, 0}, {-1.15, 0}}]}]},
   AspectRatio -> 1,
   PlotRange -> {{-1.15, 1.15}, {-1.15, 1.15}},
   Frame -> True,
   FrameTicks -> False,
   FrameLabel -> {None, None, None, None},
   GridLines -> {None, None},
   DisplayFunction -> Identity];
p = FullGraphics[p];
p[[1, 2]] = p[[1, 2]] /.
   Text[labely, {b_, c_}, {1., 0.}] ->
    Text[labely, {-1.18, 0}, {0, 0}, {0, 1}];
R1 = Show[p,
   AspectRatio -> 1,
   PlotRange -> All,
   DisplayFunction -> Identity];
```

```
(* CIG2 Projected Onto 2 D Subspace *)

stages = {"G2", "M", "G1", "S"};
cig2 = coordinates[[Position[genenames, "SPAPB2B4.03"][[1, 1]]]];
cdc13a = coordinates[[Position[genenames, "SPBC582.03"][[1, 1]]]];
cdc13b = coordinates[[Position[genenames, "SPBC582.03"][[2, 1]]]];

p = Show[{
    Graphics[{RGBColor[0, 0, 0], Text["(e)", {-1, 1.05}]}],
    Graphics[{RGBColor[0, 0, 0], Text["S. pombe", {1.1, -1.05}, {1, 0}]}],
    Graphics[{RGBColor[0, 0, 0], Text["x", {1.13, -0.07}, {1, 0}]}],
    Graphics[{RGBColor[0, 0, 0], Text["y", {-0.055, 1.145}, {0, 1}]}],
    Graphics[{RGBColor[1, 0, 0], Text["G2", {1.01, -0.54}]}],
    Graphics[{RGBColor[0, 0, 0], Text["M", {0.25, 1.07}]}],
    Graphics[{RGBColor[0, 0.5, 0], Text["G1", {-1, 0.525}]}],
    Graphics[{RGBColor[0, 0, 1], Text["S", {-0.878, -0.71}]}],
    Graphics[{RGBColor[1, 0, 0], Dashing[{0.03, 0.02}],
      {Thickness[0.009], Circle[{0, 0}, 1, {0, c15}]}}],
    Graphics[{RGBColor[1, 0, 0], Dashing[{0.03, 0.02}],
      {Thickness[0.009], Circle[{0, 0}, 1, {c15 + Pi, 2 * Pi}]}}],
    Graphics[{RGBColor[1, 1, 0], Dashing[{0.03, 0.02}],
      {Thickness[0.009], Circle[{0, 0}, 1, {c15, c14}]}}],
    Graphics[{RGBColor[0, 0.5, 0], Dashing[{0.03, 0.02}],
      {Thickness[0.009], Circle[{0, 0}, 1, {c14, Pi + c17}]}}],
    Graphics[{RGBColor[0, 0, 1], Dashing[{0.03, 0.02}],
      {Thickness[0.009], Circle[{0, 0}, 1, {Pi + c17, c15 + Pi}]}}],
    Graphics[{RGBColor[1, 0, 0], Dashing[{0.03, 0.02}],
      {Thickness[0.009], Circle[{0, 0}, 0.5, {0, c15}]}}],
    Graphics[{RGBColor[1, 0, 0], Dashing[{0.03, 0.02}],
      {Thickness[0.009], Circle[{0, 0}, 0.5, {c15 + Pi, 2 * Pi}]}}],
    Graphics[{RGBColor[1, 1, 0], Dashing[{0.03, 0.02}],
      {Thickness[0.009], Circle[{0, 0}, 0.5, {c15, c14}]}}],
    Graphics[{RGBColor[0, 0.5, 0], Dashing[{0.03, 0.02}],
      {Thickness[0.009], Circle[{0, 0}, 0.5, {c14, Pi + c17}]}}],
    Graphics[{RGBColor[0, 0, 1], Dashing[{0.03, 0.02}],
      {Thickness[0.009], Circle[{0, 0}, 0.5, {Pi + c17, c15 + Pi}]}}],
    Graphics[{RGBColor[0, 0.5, 0], PointSize[0.03], Point[cig2]}],
    Graphics[{RGBColor[0, 0.5, 0], Text["CIG2", cig2 + {0.03, -0.11}]}],
    Graphics[{RGBColor[0, 0, 0], PointSize[0.03], Point[cdc13a]}],
    Graphics[{RGBColor[0, 0, 0], PointSize[0.03], Point[cdc13b]}],
    Graphics[{RGBColor[0, 0, 0], Text["CDC13", cdc13a + {0.29, -0.08}]}],
    Graphics[{RGBColor[0, 0, 0], Text["CDC13", cdc13b + {-0.1, -0.1}]}],
    Graphics[{RGBColor[0, 0, 0], Dashing[{0.03, 0.02}], Line[{{0, 1.15}, {0, -1.15}}]}],
    Graphics[{RGBColor[0, 0, 0], Dashing[{0.03, 0.02}], Line[{{1.15, 0}, {-1.15, 0}}]}]},
   AspectRatio -> 1,
   PlotRange -> {{-1.15, 1.15}, {-1.15, 1.15}},
   Frame -> True,
   FrameTicks -> False,
   FrameLabel -> {None, None, None, None},
   GridLines -> {None, None},
   DisplayFunction -> Identity];
p = FullGraphics[p];
p[[1, 2]] = p[[1, 2]] /.
   Text[labely, {b_, c_}, {1., 0.}] ->
    Text[labely, {-1.18, 0}, {0, 0}, {0, 1}];
C1 = Show[p,
   AspectRatio -> 1,
   PlotRange -> All,
   DisplayFunction -> Identity];
```

```
(* Sort S. cerevisiae Data *)

genes = genes2;
arraynames = arraynames2;
genenames = genenames2;

(* Sort S. cerevisiae Arrays *)

average = Table[1, {a, 1, arrays}];
average = N[average / Sqrt[Dot[average, average]]];

arraycontributions13 =
  (genelets[[13]] - N[Outer[Times, Dot[genelets, average], average]][[13]]) * d2[[13, 13]];
arraycontributions14 =
  (genelets[[14]] - N[Outer[Times, Dot[genelets, average], average]][[14]]) * d2[[14, 14]];
arraycontributions15 =
  (genelets[[15]] - N[Outer[Times, Dot[genelets, average], average]][[15]]) * d2[[15, 15]];
arraycontributions16 =
  (genelets[[16]] - N[Outer[Times, Dot[genelets, average], average]][[16]]) * d2[[16, 16]];
arraycontributions17 =
  (genelets[[17]] - N[Outer[Times, Dot[genelets, average], average]][[17]]) * d2[[17, 17]];

(* Project Arrays from 5 D Arraylets Subspace Onto 2 D Subspace  *)

coordinates = Table[{
     (Dot[genelets[[13]], x] * arraycontributions13[[a]] +
        Dot[genelets[[14]], x] * arraycontributions14[[a]] +
        Dot[genelets[[15]], x] * arraycontributions15[[a]] +
        Dot[genelets[[16]], x] * arraycontributions16[[a]] +
        Dot[genelets[[17]], x] * arraycontributions17[[a]]) /
      Sqrt[(a13 * arraycontributions13[[a]])^2 + (a14 * arraycontributions14[[a]])^2 +
        (a15 * arraycontributions15[[a]])^2 + (a16 * arraycontributions16[[a]])^2 +
        (a17 * arraycontributions17[[a]])^2 +
        c1314 * Abs[(a13 * arraycontributions13[[a]]) * (a14 * arraycontributions14[[a]])] +
        c1315 * Abs[(a13 * arraycontributions13[[a]]) * (a15 * arraycontributions15[[a]])] +
        c1316 * Abs[(a13 * arraycontributions13[[a]]) * (a16 * arraycontributions16[[a]])] +
        c1317 * Abs[(a13 * arraycontributions13[[a]]) * (a17 * arraycontributions17[[a]])] +
        c1415 * Abs[(a14 * arraycontributions14[[a]]) * (a15 * arraycontributions15[[a]])] +
        c1416 * Abs[(a14 * arraycontributions14[[a]]) * (a16 * arraycontributions16[[a]])] +
        c1417 * Abs[(a14 * arraycontributions14[[a]]) * (a17 * arraycontributions17[[a]])] +
        c1516 * Abs[(a15 * arraycontributions15[[a]]) * (a16 * arraycontributions16[[a]])] +
        c1517 * Abs[(a15 * arraycontributions15[[a]]) * (a17 * arraycontributions17[[a]])] +
        c1617 * Abs[(a16 * arraycontributions16[[a]]) * (a17 * arraycontributions17[[a]])]],
     (Dot[genelets[[13]], y] * arraycontributions13[[a]] +
        Dot[genelets[[14]], y] * arraycontributions14[[a]] +
        Dot[genelets[[15]], y] * arraycontributions15[[a]] +
        Dot[genelets[[16]], y] * arraycontributions16[[a]] +
        Dot[genelets[[17]], y] * arraycontributions17[[a]]) /
      Sqrt[(a13 * arraycontributions13[[a]])^2 + (a14 * arraycontributions14[[a]])^2 +
        (a15 * arraycontributions15[[a]])^2 + (a16 * arraycontributions16[[a]])^2 +
        (a17 * arraycontributions17[[a]])^2 +
        c1314 * Abs[(a13 * arraycontributions13[[a]]) * (a14 * arraycontributions14[[a]])] +
        c1315 * Abs[(a13 * arraycontributions13[[a]]) * (a15 * arraycontributions15[[a]])] +
        c1316 * Abs[(a13 * arraycontributions13[[a]]) * (a16 * arraycontributions16[[a]])] +
        c1317 * Abs[(a13 * arraycontributions13[[a]]) * (a17 * arraycontributions17[[a]])] +
        c1415 * Abs[(a14 * arraycontributions14[[a]]) * (a15 * arraycontributions15[[a]])] +
        c1416 * Abs[(a14 * arraycontributions14[[a]]) * (a16 * arraycontributions16[[a]])] +
        c1417 * Abs[(a14 * arraycontributions14[[a]]) * (a17 * arraycontributions17[[a]])] +
        c1516 * Abs[(a15 * arraycontributions15[[a]]) * (a16 * arraycontributions16[[a]])] +
        c1517 * Abs[(a15 * arraycontributions15[[a]]) * (a17 * arraycontributions17[[a]])] +
        c1617 * Abs[(a16 * arraycontributions16[[a]]) * (a17 * arraycontributions17[[a]])]]},
    {a, 1, arrays}];
```

```
(* Create Parametric Plot of S. cerevisiae Arrays Projected Onto 2 D Subspace *)

Clear[points];
points1 = {Point[coordinates[[1]]], Point[coordinates[[2]]], Point[coordinates[[11]]]};
points2 = {Point[coordinates[[3]]], Point[coordinates[[4]]],
   Point[coordinates[[5]]], Point[coordinates[[12]]], Point[coordinates[[13]]]};
points3 = {Point[coordinates[[6]]], Point[coordinates[[14]]], Point[coordinates[[15]]]};
points4 = {Point[coordinates[[7]]]};
points5 = {Point[coordinates[[8]]], Point[coordinates[[9]]],
   Point[coordinates[[10]]], Point[coordinates[[16]]],
   Point[coordinates[[17]]]};
textcoordinates = coordinates;
Do[
  textcoordinates[[a, 1]] = If[
    textcoordinates[[a, 1]] > 0,
    textcoordinates[[a, 1]] + 0.095,
    textcoordinates[[a, 1]] - 0.1],
  {a, 1, 9}];

Do[
  textcoordinates[[a, 1]] =
   If[textcoordinates[[a, 1]] > 0,
    textcoordinates[[a, 1]] + 0.11,
    textcoordinates[[a, 1]] - 0.12],
  {a, 10, arrays}];
textcoordinates[[1]] = textcoordinates[[1]] - {0.095, -0.095};
textcoordinates[[6]] = textcoordinates[[6]] + {0.2, 0};
textcoordinates[[10]] = textcoordinates[[10]] - {0.1, -0.095};
textcoordinates[[11]] = textcoordinates[[11]] - {0.11, 0.1};
textcoordinates[[12]] = textcoordinates[[12]] + {0.12, -0.12};
textcoordinates[[15]] = textcoordinates[[15]] + {0, 0.05};
textcoordinates[[16]] = textcoordinates[[16]] + {0.24, 0};
textcoordinates[[17]] = textcoordinates[[17]] + {0, 0.1};
```

```
texts = Table[Text[a, textcoordinates[[a]]], {a, 1, arrays}];
radius = Sqrt[coordinates[[2, 1]]^2 + coordinates[[2, 2]]^2];
p = Show[{
    Graphics[{RGBColor[0, 0, 0], Text["(b)", {-1.22, 1.15}, {-1, 0}]}],
    Graphics[{RGBColor[0, 0, 0], Text["x", {1.22, -0.075}, {1, 0}]}],
    Graphics[{RGBColor[0, 0, 0], Text["y", {-0.065, 1.22}, {0, 1}]}],
    Graphics[{RGBColor[0, 0, 0], Text["M/G1", {0.825, 0.83}]}],
    Graphics[{RGBColor[0, 0.5, 0], Text["G1", {-1, 0.525}]}],
    Graphics[{RGBColor[0, 0, 1], Text["S", {-0.878, -0.71}]}],
    Graphics[{RGBColor[1, 0, 0], Text["S/G2", {0.57, -1}]}],
    Graphics[{RGBColor[1, 0.5, 0], Text["G2/M", {1.07, -0.52}]}],
    Graphics[{RGBColor[1, 1, 0], PointSize[0.035], points1}],
    Graphics[{RGBColor[0, 0.5, 0], PointSize[0.035], points2}],
    Graphics[{RGBColor[0, 0, 1], PointSize[0.035], points3}],
    Graphics[{RGBColor[1, 0, 0], PointSize[0.035], points4}],
    Graphics[{RGBColor[1, 0.5, 0], PointSize[0.035], points5}],
    Graphics[{RGBColor[0, 0, 0], Text["u2,17", {1.235, 0.5}, {1, 0}]}],
    Graphics[{RGBColor[0, 0, 0], Text["u2,16", {0.5, -1.12}, {-1, 0}]}],
    Graphics[{RGBColor[0, 0, 0], Text["u2,15", {0.7, 1.12}, {-1, 0}]}],
    Graphics[{RGBColor[0, 0, 0], Text["u2,14", {-0.45, 1.12}, {1, 0}]}],
    Graphics[{RGBColor[0, 0, 0], Text["u2,13", {-0.025, -1.12}, {1, 0}]}],
    Graphics[texts],
    Graphics[{RGBColor[0, 0, 0], Dashing[{0.03, 0.02}], Circle[{0, 0}, 0.5]}],
    Graphics[{RGBColor[0, 0, 0], Dashing[{0.03, 0.02}], Circle[{0, 0}, 1]}],
    Graphics[{RGBColor[0, 0, 0], Dashing[{0.03, 0.02}], Line[{{0, 1.25}, {0, -1.25}}]}],
    Graphics[{RGBColor[0, 0, 0], Dashing[{0.03, 0.02}], Line[{{1.25, 0}, {-1.25, 0}}]}],
    Graphics[{RGBColor[0, 0, 0], Arrow[{1.25 / Tan[c13], 1.25}, {-1.25 / Tan[c13], -1.25},
       HeadCenter -> 0.5, HeadLength -> 0.035, HeadWidth -> 0.75]}],
    Graphics[{RGBColor[0, 0, 0], Arrow[{-1.25 / Tan[c14 - Pi], -1.25}, {1.25 / Tan[c14 - Pi], 1.25},
       HeadCenter -> 0.5, HeadLength -> 0.035, HeadWidth -> 0.75]}],
    Graphics[{RGBColor[0, 0, 0], Arrow[{-1.25 / Tan[c15], -1.25}, {1.25 / Tan[c15], 1.25},
       HeadCenter -> 0.5, HeadLength -> 0.035, HeadWidth -> 0.75]}],
    Graphics[{RGBColor[0, 0, 0], Arrow[{1.25 / Tan[c16], 1.25}, {-1.25 / Tan[c16], -1.25},
       HeadCenter -> 0.5, HeadLength -> 0.035, HeadWidth -> 0.75]}],
    Graphics[{RGBColor[0, 0, 0], Arrow[{-1.25, -1.25 * Tan[c17]}, {1.25, 1.25 * Tan[c17]},
       HeadCenter -> 0.5, HeadLength -> 0.035, HeadWidth -> 0.75]}]},
   AspectRatio -> 1,
   PlotRange -> {{-1.25, 1.25}, {-1.25, 1.25}},
   Frame -> True,
   FrameTicks -> False,
   FrameLabel -> {None, None, None, None},
   GridLines -> {None, None},
   DisplayFunction -> Identity];
p = FullGraphics[p];
p[[1, 2]] = p[[1, 2]] /.
   Text[labely, {b_, c_}, {1., 0.}] ->
    Text[labely, {-1.18, 0}, {0, 0}, {0, 1}];
t1 = Show[p,
   AspectRatio -> 1,
   PlotRange -> All,
   DisplayFunction -> Identity];
```

```
(* Sort S. cerevisiae Genes *)

(* Center Arraylets and Calculate Contributions of Genelets to Genes *)

centerarraylets = Transpose[arraylets2];
average = Table[1, {a, 1, genes}];
average = N[average / Sqrt[Dot[average, average]]];
centerarraylets = centerarraylets - N[Outer[Times, Dot[centerarraylets, average], average]];
centerarraylets = Transpose[centerarraylets];
genecontributions = Transpose[Dot[centerarraylets, d2]];

(* Project Genes from 5 D Genelets Subspace Onto 2 D Subspace  *)

coordinates = Table[{
    (Dot[genelets[[13]], x] * genecontributions[[13, a]] +
       Dot[genelets[[14]], x] * genecontributions[[14, a]] +
       Dot[genelets[[15]], x] * genecontributions[[15, a]] +
       Dot[genelets[[16]], x] * genecontributions[[16, a]] +
       Dot[genelets[[17]], x] * genecontributions[[17, a]]) /
     Sqrt[(a13 * genecontributions[[13, a]])^2 + (a14 * genecontributions[[14, a]])^2 +
       (a15 * genecontributions[[15, a]])^2 + (a16 * genecontributions[[16, a]])^2 +
       (a17 * genecontributions[[17, a]])^2 +
       c1314 * Abs[(a13 * genecontributions[[13, a]]) * (a14 * genecontributions[[14, a]])] +
       c1315 * Abs[(a13 * genecontributions[[13, a]]) * (a15 * genecontributions[[15, a]])] +
       c1316 * Abs[(a13 * genecontributions[[13, a]]) * (a16 * genecontributions[[16, a]])] +
       c1317 * Abs[(a13 * genecontributions[[13, a]]) * (a17 * genecontributions[[17, a]])] +
       c1415 * Abs[(a14 * genecontributions[[14, a]]) * (a15 * genecontributions[[15, a]])] +
       c1416 * Abs[(a14 * genecontributions[[14, a]]) * (a16 * genecontributions[[16, a]])] +
       c1417 * Abs[(a14 * genecontributions[[14, a]]) * (a17 * genecontributions[[17, a]])] +
       c1516 * Abs[(a15 * genecontributions[[15, a]]) * (a16 * genecontributions[[16, a]])] +
       c1517 * Abs[(a15 * genecontributions[[15, a]]) * (a17 * genecontributions[[17, a]])] +
       c1617 * Abs[(a16 * genecontributions[[16, a]]) * (a17 * genecontributions[[17, a]])]],
    (Dot[genelets[[13]], y] * genecontributions[[13, a]] +
       Dot[genelets[[14]], y] * genecontributions[[14, a]] +
       Dot[genelets[[15]], y] * genecontributions[[15, a]] +
       Dot[genelets[[16]], y] * genecontributions[[16, a]] +
       Dot[genelets[[17]], y] * genecontributions[[17, a]]) /
     Sqrt[(a13 * genecontributions[[13, a]])^2 + (a14 * genecontributions[[14, a]])^2 +
       (a15 * genecontributions[[15, a]])^2 + (a16 * genecontributions[[16, a]])^2 +
       (a17 * genecontributions[[17, a]])^2 +
       c1314 * Abs[(a13 * genecontributions[[13, a]]) * (a14 * genecontributions[[14, a]])] +
       c1315 * Abs[(a13 * genecontributions[[13, a]]) * (a15 * genecontributions[[15, a]])] +
       c1316 * Abs[(a13 * genecontributions[[13, a]]) * (a16 * genecontributions[[16, a]])] +
       c1317 * Abs[(a13 * genecontributions[[13, a]]) * (a17 * genecontributions[[17, a]])] +
       c1415 * Abs[(a14 * genecontributions[[14, a]]) * (a15 * genecontributions[[15, a]])] +
       c1416 * Abs[(a14 * genecontributions[[14, a]]) * (a16 * genecontributions[[16, a]])] +
       c1417 * Abs[(a14 * genecontributions[[14, a]]) * (a17 * genecontributions[[17, a]])] +
       c1516 * Abs[(a15 * genecontributions[[15, a]]) * (a16 * genecontributions[[16, a]])] +
       c1517 * Abs[(a15 * genecontributions[[15, a]]) * (a17 * genecontributions[[17, a]])] +
       c1617 * Abs[(a16 * genecontributions[[16, a]]) * (a17 * genecontributions[[17, a]])]]},
   {a, 1, genes}];

(* Create Parametric Plot of S. cerevisiae Cell Cycle Genes Projected Onto 2 D Subspace *)

list = AppendRows[TakeColumns[genenames, {1, 1}], TakeColumns[genenames, {3, 3}]];
stages = {"M/G1", "G1", "S", "S/G2", "G2/M"};
points = {points1, points2, points3, points4, points5};
radii = {radii1, radii2, radii3, radii4, radii5};
```

```
Do[{
  position = Position[list, stages[[b]]],
  points[[b]] = Table[Point[coordinates[[position[[a]][[1]]]]], {a, 1, Dimensions[position][[1]]}],
  radii[[b]] =
   Table[Sqrt[coordinates[[position[[a]][[1]]]][[1]]^2 + coordinates[[position[[a]][[1]]]][[2]]^2],
    {a, 1, Dimensions[position][[1]]}]},
 {b, 1, Dimensions[stages][[1]]}]
radii = Sort[Flatten[radii]];
N[Round[radii[[87]] * 100] / 100]
N[Round[radii[[88]] * 100] / 100]
```

```
0.49
```

```
0.5
```

```
p = Show[{
    Graphics[{RGBColor[0, 0, 0], Text["(e)", {-1.22, 1.15}, {-1, 0}]}],
    Graphics[{RGBColor[0, 0, 0], Text["x", {1.22, -0.075}, {1, 0}]}],
    Graphics[{RGBColor[0, 0, 0], Text["y", {-0.065, 1.22}, {0, 1}]}],
    Graphics[{RGBColor[0, 0, 0], Text["M/G1", {0.825, 0.83}]}],
    Graphics[{RGBColor[0, 0.5, 0], Text["G1", {-1, 0.525}]}],
    Graphics[{RGBColor[0, 0, 1], Text["S", {-0.878, -0.71}]}],
    Graphics[{RGBColor[1, 0, 0], Text["S/G2", {0.57, -1}]}],
    Graphics[{RGBColor[1, 0.5, 0], Text["G2/M", {1.07, -0.52}]}],
    Graphics[{RGBColor[1, 0.5, 0], PointSize[0.02], points[[5]]}],
    Graphics[{RGBColor[1, 1, 0], PointSize[0.02], points[[1]]}],
    Graphics[{RGBColor[1, 0, 0], PointSize[0.02], points[[4]]}],
    Graphics[{RGBColor[0, 0.5, 0], PointSize[0.02], points[[2]]}],
    Graphics[{RGBColor[0, 0, 0], Text["v17", {1.235, 0.5}, {1, 0}]}],
    Graphics[{RGBColor[0, 0, 0], Text["v16", {0.5, -1.12}, {-1, 0}]}],
    Graphics[{RGBColor[0, 0, 0], Text["v15", {0.7, 1.12}, {-1, 0}]}],
    Graphics[{RGBColor[0, 0, 0], Text["v14", {-0.45, 1.12}, {1, 0}]}],
    Graphics[{RGBColor[0, 0, 0], Text["v13", {-0.025, -1.12}, {1, 0}]}],
    Graphics[{RGBColor[0, 0, 0], Dashing[{0.03, 0.02}], Circle[{0, 0}, 0.5]}],
    Graphics[{RGBColor[0, 0, 0], Dashing[{0.03, 0.02}], Circle[{0, 0}, 1]}],
    Graphics[{RGBColor[0, 0, 0], Dashing[{0.03, 0.02}], Line[{{0, 1.25}, {0, -1.25}}]}],
    Graphics[{RGBColor[0, 0, 0], Dashing[{0.03, 0.02}], Line[{{1.25, 0}, {-1.25, 0}}]}],
    Graphics[{RGBColor[0, 0, 0], Arrow[{1.25 / Tan[c13], 1.25}, {-1.25 / Tan[c13], -1.25},
       HeadCenter -> 0.5, HeadLength -> 0.035, HeadWidth -> 0.75]}],
    Graphics[{RGBColor[0, 0, 0], Arrow[{-1.25 / Tan[c14 - Pi], -1.25}, {1.25 / Tan[c14 - Pi], 1.25},
       HeadCenter -> 0.5, HeadLength -> 0.035, HeadWidth -> 0.75]}],
    Graphics[{RGBColor[0, 0, 0], Arrow[{-1.25 / Tan[c15], -1.25}, {1.25 / Tan[c15], 1.25},
       HeadCenter -> 0.5, HeadLength -> 0.035, HeadWidth -> 0.75]}],
    Graphics[{RGBColor[0, 0, 0], Arrow[{1.25 / Tan[c16], 1.25}, {-1.25 / Tan[c16], -1.25},
       HeadCenter -> 0.5, HeadLength -> 0.035, HeadWidth -> 0.75]}],
    Graphics[{RGBColor[0, 0, 0], Arrow[{-1.25, -1.25 * Tan[c17]}, {1.25, 1.25 * Tan[c17]},
       HeadCenter -> 0.5, HeadLength -> 0.035, HeadWidth -> 0.75]}]},
   AspectRatio -> 1,
   PlotRange -> {{-1.25, 1.25}, {-1.25, 1.25}},
   Frame -> True,
   FrameTicks -> False,
   FrameLabel -> {None, None, None, None},
   GridLines -> {None, None},
   DisplayFunction -> Identity];
p = FullGraphics[p];
p[[1, 2]] = p[[1, 2]] /.
   Text[labely, {b_, c_}, {1., 0.}] ->
    Text[labely, {-1.18, 0}, {0, 0}, {0, 1}];
t2 = Show[p,
   AspectRatio -> 1,
   PlotRange -> All,
   DisplayFunction -> Identity];
```

```
R[theta_] = {{Cos[theta], Sin[theta]}, {-Sin[theta], Cos[theta]}};
z = Transpose[{{0.4, 0}, {1.1, 0}, {0.75, 0.25}}];
polypoints[theta_] = Transpose[Dot[R[theta], z]];
p = Show[{
    Graphics[{RGBColor[0, 0, 0], Text["(h)", {-1.22, 1.15}, {-1, 0}]}],
    Graphics[{RGBColor[0, 0, 0], Text["x", {1.22, -0.075}, {1, 0}]}],
    Graphics[{RGBColor[0, 0, 0], Text["y", {-0.065, 1.22}, {0, 1}]}],
    Graphics[{RGBColor[0, 0, 0], Text["M/G1", {0.825, 0.83}]}],
    Graphics[{RGBColor[0, 0.5, 0], Text["G1", {-1, 0.525}]}],
    Graphics[{RGBColor[0, 0, 1], Text["S", {-0.878, -0.71}]}],
    Graphics[{RGBColor[1, 0, 0], Text["S/G2", {0.57, -1}]}],
    Graphics[{RGBColor[1, 0.5, 0], Text["G2/M", {1.07, -0.52}]}],
    Graphics[{RGBColor[0, 0, 0], Text["17", {1.235, 0.5}, {1, 0}]}],
    Graphics[{RGBColor[0, 0, 0], Text["16", {0.5, -1.12}, {-1, 0}]}],
    Graphics[{RGBColor[0, 0, 0], Text["15", {0.7, 1.12}, {-1, 0}]}],
    Graphics[{RGBColor[0, 0, 0], Text["14", {-0.45, 1.12}, {1, 0}]}],
    Graphics[{RGBColor[0, 0, 0], Text["13", {-0.025, -1.12}, {1, 0}]}],
    Graphics[{RGBColor[1, 0.5, 0], Disk[{0, 0}, 1, {Pi + c16, 2 * Pi}]}],
    Graphics[{RGBColor[1, 0.5, 0], Disk[{0, 0}, 1, {0, c17}]}],
    Graphics[{RGBColor[1, 1, 0], Disk[{0, 0}, 1, {c17, c14}]}],
    Graphics[{RGBColor[0, 0.5, 0], Disk[{0, 0}, 1, {c14, Pi + c17}]}],
    Graphics[{RGBColor[0, 0, 1], Disk[{0, 0}, 1, {Pi + c17, Pi + c15}]}],
    Graphics[{RGBColor[1, 0, 0], Disk[{0, 0}, 1, {Pi + c15, 2 * Pi + c16}]}],
    Graphics[{RGBColor[1, 1, 1], Disk[{0, 0}, 0.5]}],
    Graphics[{RGBColor[1, 0.5, 0], Polygon[polypoints[2 * Pi - c17]]}],
    Graphics[{RGBColor[1, 1, 0], Polygon[polypoints[2 * Pi - c14]]}],
    Graphics[{RGBColor[0, 0.5, 0], Polygon[polypoints[Pi - c17]]}],
    Graphics[{RGBColor[0, 0, 1], Polygon[polypoints[Pi - c15]]}],
    Graphics[{RGBColor[1, 0, 0], Polygon[polypoints[2 * Pi - c16]]}],
    Graphics[{RGBColor[0, 0, 0], Dashing[{0.03, 0.02}], Line[{{0, 1.25}, {0, -1.25}}]}],
    Graphics[{RGBColor[0, 0, 0], Dashing[{0.03, 0.02}], Line[{{1.25, 0}, {-1.25, 0}}]}],
    Graphics[{RGBColor[0, 0, 0], Arrow[{1.25 / Tan[c13], 1.25}, {-1.25 / Tan[c13], -1.25},
       HeadCenter -> 0.5, HeadLength -> 0.035, HeadWidth -> 0.75]}],
    Graphics[{RGBColor[0, 0, 0], Arrow[{-1.25 / Tan[c14 - Pi], -1.25}, {1.25 / Tan[c14 - Pi], 1.25},
       HeadCenter -> 0.5, HeadLength -> 0.035, HeadWidth -> 0.75]}],
    Graphics[{RGBColor[0, 0, 0], Arrow[{-1.25 / Tan[c15], -1.25}, {1.25 / Tan[c15], 1.25},
       HeadCenter -> 0.5, HeadLength -> 0.035, HeadWidth -> 0.75]}],
    Graphics[{RGBColor[0, 0, 0], Arrow[{1.25 / Tan[c16], 1.25}, {-1.25 / Tan[c16], -1.25},
       HeadCenter -> 0.5, HeadLength -> 0.035, HeadWidth -> 0.75]}],
    Graphics[{RGBColor[0, 0, 0], Arrow[{-1.25, -1.25 * Tan[c17]}, {1.25, 1.25 * Tan[c17]},
       HeadCenter -> 0.5, HeadLength -> 0.035, HeadWidth -> 0.75]}]},
   AspectRatio -> 1,
   PlotRange -> {{-1.25, 1.25}, {-1.25, 1.25}},
   Frame -> True,
   FrameTicks -> False,
   FrameLabel -> {None, None, None, None},
   GridLines -> {None, None},
   DisplayFunction -> Identity];
p = FullGraphics[p];
p[[1, 2]] = p[[1, 2]] /.
   Text[labely, {b_, c_}, {1., 0.}] ->
    Text[labely, {-1.18, 0}, {0, 0}, {0, 1}];
t3 = Show[p,
   AspectRatio -> 1,
   PlotRange -> All,
   DisplayFunction -> Identity];
Clear[z];

(* SNQ2, PDR5, PDR10, PDR15, PDR1 Projected Onto 2 D Subspace *)

stages = {"M/G1", "G1", "S", "S/G2", "G2/M"};
PDR1 = coordinates[[Position[genenames, "YGL013C"][[1, 1]]]];
SNQ2 = coordinates[[Position[genenames, "YDR011W"][[1, 1]]]];
PDR10 = coordinates[[Position[genenames, "YOR328W"][[1, 1]]]];
PDR15 = coordinates[[Position[genenames, "YDR406W"][[1, 1]]]];
PDR5 = coordinates[[Position[genenames, "YOR153W"][[1, 1]]]];
```

```
p = Show[{
    Graphics[{RGBColor[0, 0, 0], Text["(b)", {-1, 1.05}]}],
    Graphics[{RGBColor[0, 0, 0], Text["S. cerevisiae", {1.1, -1.05}, {1, 0}]}],
    Graphics[{RGBColor[0, 0, 0], Text["x", {1.13, -0.07}, {1, 0}]}],
    Graphics[{RGBColor[0, 0, 0], Text["y", {-0.055, 1.145}, {0, 1}]}],
    Graphics[{RGBColor[0, 0, 0], Text["M/G1", {0.825, 0.83}]}],
    Graphics[{RGBColor[0, 0.5, 0], Text["G1", {-1, 0.525}]}],
    Graphics[{RGBColor[0, 0, 1], Text["S", {-0.878, -0.71}]}],
    Graphics[{RGBColor[1, 0, 0], Text["S/G2", {-0.2, -1.08}]}],
    Graphics[{RGBColor[1, 0.5, 0], Text["G2/M", {1, -0.6}]}],
    Graphics[{RGBColor[1, 0.5, 0],
      Dashing[{0.03, 0.02}], {Thickness[0.009], Circle[{0, 0}, 1, {2 * Pi + c16, 2 * Pi}]}}],
    Graphics[{RGBColor[1, 0.5, 0], Dashing[{0.03, 0.02}],
      {Thickness[0.009], Circle[{0, 0}, 1, {0, c17}]}}],
    Graphics[{RGBColor[1, 1, 0], Dashing[{0.03, 0.02}], {Thickness[0.009], Circle[{0, 0}, 1, {c17, c14}]}}],
    Graphics[
     {RGBColor[0, 0.5, 0], Dashing[{0.03, 0.02}], {Thickness[0.009], Circle[{0, 0}, 1, {c14, Pi + c17}]}}],
    Graphics[{RGBColor[0, 0, 1], Dashing[{0.03, 0.02}],
      {Thickness[0.009], Circle[{0, 0}, 1, {Pi + c17, Pi + c15}]}}],
    Graphics[{RGBColor[1, 0, 0], Dashing[{0.03, 0.02}],
      {Thickness[0.009], Circle[{0, 0}, 1, {Pi + c15, 2 * Pi + c16}]}}],
    Graphics[{RGBColor[1, 0.5, 0], Dashing[{0.03, 0.02}],
      {Thickness[0.009], Circle[{0, 0}, 0.5, {2 * Pi + c16, 2 * Pi}]}}],
    Graphics[{RGBColor[1, 0.5, 0], Dashing[{0.03, 0.02}],
      {Thickness[0.009], Circle[{0, 0}, 0.5, {0, c17}]}}],
    Graphics[{RGBColor[1, 1, 0], Dashing[{0.03, 0.02}],
      {Thickness[0.009], Circle[{0, 0}, 0.5, {c17, c14}]}}],
    Graphics[{RGBColor[0, 0.5, 0], Dashing[{0.03, 0.02}],
      {Thickness[0.009], Circle[{0, 0}, 0.5, {c14, Pi + c17}]}}],
    Graphics[{RGBColor[0, 0, 1], Dashing[{0.03, 0.02}],
      {Thickness[0.009], Circle[{0, 0}, 0.5, {Pi + c17, Pi + c15}]}}],
    Graphics[{RGBColor[1, 0, 0], Dashing[{0.03, 0.02}],
      {Thickness[0.009], Circle[{0, 0}, 0.5, {Pi + c15, 2 * Pi + c16}]}}],
    Graphics[{RGBColor[1, 0, 0], PointSize[0.03], Point[SNQ2]}],
    Graphics[{RGBColor[1, 0, 0], Text["SNQ2", SNQ2 + {-0.25, -0.07}]}],
    Graphics[{RGBColor[0, 0, 0], PointSize[0.03], Point[PDR10]}],
    Graphics[{RGBColor[0, 0, 0], Text["PDR10", PDR10 + {0.25, 0}]}],
    Graphics[{RGBColor[0, 0, 0], PointSize[0.03], Point[PDR15]}],
    Graphics[{RGBColor[0, 0, 0], Text["PDR15", PDR15 + {-0.065, -0.125}]}],
    Graphics[{RGBColor[1, 0.5, 0], PointSize[0.03], Point[PDR5]}],
    Graphics[{RGBColor[1, 0.5, 0], Text["PDR5", PDR5 + {0.1, 0.1}]}],
    Graphics[{RGBColor[1, 0, 0], PointSize[0.03], Point[PDR1]}],
    Graphics[{RGBColor[1, 0, 0], Text["PDR1", PDR1 + {0, 0.1}]}],
    Graphics[{RGBColor[0, 0, 0], Dashing[{0.03, 0.02}], Line[{{0, 1.15}, {0, -1.15}}]}],
    Graphics[{RGBColor[0, 0, 0], Dashing[{0.03, 0.02}], Line[{{1.15, 0}, {-1.15, 0}}]}]},
   AspectRatio -> 1,
   PlotRange -> {{-1.15, 1.15}, {-1.15, 1.15}},
   Frame -> True,
   FrameTicks -> False,
   FrameLabel -> {None, None, None, None},
   GridLines -> {None, None},
   DisplayFunction -> Identity];
p = FullGraphics[p];
p[[1, 2]] = p[[1, 2]] /.
   Text[labely, {b_, c_}, {1., 0.}] ->
    Text[labely, {-1.18, 0}, {0, 0}, {0, 1}];
O2 = Show[p,
   AspectRatio -> 1,
   PlotRange -> All,
   DisplayFunction -> Identity];
```

```
(* PLB1 and PLB2 Projected Onto 2 D Subspace *)

stages = {"M/G1", "G1", "S", "S/G2", "G2/M"};
PLB1 = coordinates[[Position[genenames, "YMR008C"][[1, 1]]]];
PLB3 = coordinates[[Position[genenames, "YOL011W"][[1, 1]]]];

p = Show[{
    Graphics[{RGBColor[0, 0, 0], Text["(d)", {-1, 1.05}]}],
    Graphics[{RGBColor[0, 0, 0], Text["S. cerevisiae", {1.1, -1.05}, {1, 0}]}],
    Graphics[{RGBColor[0, 0, 0], Text["x", {1.13, -0.07}, {1, 0}]}],
    Graphics[{RGBColor[0, 0, 0], Text["y", {-0.055, 1.145}, {0, 1}]}],
    Graphics[{RGBColor[0, 0, 0], Text["M/G1", {0.825, 0.83}]}],
    Graphics[{RGBColor[0, 0.5, 0], Text["G1", {-1, 0.525}]}],
    Graphics[{RGBColor[0, 0, 1], Text["S", {-0.878, -0.71}]}],
    Graphics[{RGBColor[1, 0, 0], Text["S/G2", {-0.2, -1.08}]}],
    Graphics[{RGBColor[1, 0.5, 0], Text["G2/M", {1, -0.6}]}],
    Graphics[{RGBColor[1, 0.5, 0],
      Dashing[{0.03, 0.02}], {Thickness[0.009], Circle[{0, 0}, 1, {2 * Pi + c16, 2 * Pi}]}}],
    Graphics[{RGBColor[1, 0.5, 0], Dashing[{0.03, 0.02}],
      {Thickness[0.009], Circle[{0, 0}, 1, {0, c17}]}}],
    Graphics[{RGBColor[1, 1, 0], Dashing[{0.03, 0.02}], {Thickness[0.009], Circle[{0, 0}, 1, {c17, c14}]}}],
    Graphics[
     {RGBColor[0, 0.5, 0], Dashing[{0.03, 0.02}], {Thickness[0.009], Circle[{0, 0}, 1, {c14, Pi + c17}]}}],
    Graphics[{RGBColor[0, 0, 1], Dashing[{0.03, 0.02}],
      {Thickness[0.009], Circle[{0, 0}, 1, {Pi + c17, Pi + c15}]}}],
    Graphics[{RGBColor[1, 0, 0], Dashing[{0.03, 0.02}],
      {Thickness[0.009], Circle[{0, 0}, 1, {Pi + c15, 2 * Pi + c16}]}}],
    Graphics[{RGBColor[1, 0.5, 0], Dashing[{0.03, 0.02}],
      {Thickness[0.009], Circle[{0, 0}, 0.5, {2 * Pi + c16, 2 * Pi}]}}],
    Graphics[{RGBColor[1, 0.5, 0], Dashing[{0.03, 0.02}],
      {Thickness[0.009], Circle[{0, 0}, 0.5, {0, c17}]}}],
    Graphics[{RGBColor[1, 1, 0], Dashing[{0.03, 0.02}],
      {Thickness[0.009], Circle[{0, 0}, 0.5, {c17, c14}]}}],
    Graphics[{RGBColor[0, 0.5, 0], Dashing[{0.03, 0.02}],
      {Thickness[0.009], Circle[{0, 0}, 0.5, {c14, Pi + c17}]}}],
    Graphics[{RGBColor[0, 0, 1], Dashing[{0.03, 0.02}],
      {Thickness[0.009], Circle[{0, 0}, 0.5, {Pi + c17, Pi + c15}]}}],
    Graphics[{RGBColor[1, 0, 0], Dashing[{0.03, 0.02}],
      {Thickness[0.009], Circle[{0, 0}, 0.5, {Pi + c15, 2 * Pi + c16}]}}],
    Graphics[{RGBColor[0, 0, 0], PointSize[0.03], Point[PLB1]}],
    Graphics[{RGBColor[0, 0, 0], Text["PLB1", PLB1 + {-0.15, -0.1}]}],
    Graphics[{RGBColor[0, 0, 0], PointSize[0.03], Point[PLB3]}],
    Graphics[{RGBColor[0, 0, 0], Text["PLB3", PLB3 + {0.2, 0}]}],
    Graphics[{RGBColor[0, 0, 0], Dashing[{0.03, 0.02}], Line[{{0, 1.15}, {0, -1.15}}]}],
    Graphics[{RGBColor[0, 0, 0], Dashing[{0.03, 0.02}], Line[{{1.15, 0}, {-1.15, 0}}]}]},
   AspectRatio -> 1,
   PlotRange -> {{-1.15, 1.15}, {-1.15, 1.15}},
   Frame -> True,
   FrameTicks -> False,
   FrameLabel -> {None, None, None, None},
   GridLines -> {None, None},
   DisplayFunction -> Identity];
p = FullGraphics[p];
p[[1, 2]] = p[[1, 2]] /.
   Text[labely, {b_, c_}, {1., 0.}] ->
    Text[labely, {-1.18, 0}, {0, 0}, {0, 1}];
R2 = Show[p,
   AspectRatio -> 1,
   PlotRange -> All,
   DisplayFunction -> Identity];
```

```
(* CLB1, CLB2, CLB3, CLB4, CLB4, CLN1, CLN2 and CLN3  Projected Onto 2 D Subspace *)

stages = {"M/G1", "G1", "S", "S/G2", "G2/M"};
CLN1 = coordinates[[Position[genenames, "YMR199W"][[1, 1]]]];
CLN2 = coordinates[[Position[genenames, "YPL256C"][[1, 1]]]];
CLN3 = coordinates[[Position[genenames, "YAL040C"][[1, 1]]]];
CLB1 = coordinates[[Position[genenames, "YGR108W"][[1, 1]]]];
CLB2 = coordinates[[Position[genenames, "YPR119W"][[1, 1]]]];
CLB3 = coordinates[[Position[genenames, "YDL155W"][[1, 1]]]];
CLB4 = coordinates[[Position[genenames, "YLR210W"][[1, 1]]]];
CLB5 = coordinates[[Position[genenames, "YPR120C"][[1, 1]]]];
```

```
p = Show[{
    Graphics[{RGBColor[0, 0, 0], Text["(f)", {-1, 1.05}]}],
    Graphics[{RGBColor[0, 0, 0], Text["S. cerevisiae", {1.1, -1.05}, {1, 0}]}],
    Graphics[{RGBColor[0, 0, 0], Text["x", {1.13, -0.07}, {1, 0}]}],
    Graphics[{RGBColor[0, 0, 0], Text["y", {-0.055, 1.145}, {0, 1}]}],
    Graphics[{RGBColor[0, 0, 0], Text["M/G1", {0.825, 0.83}]}],
    Graphics[{RGBColor[0, 0.5, 0], Text["G1", {-1, 0.525}]}],
    Graphics[{RGBColor[0, 0, 1], Text["S", {-0.878, -0.71}]}],
    Graphics[{RGBColor[1, 0, 0], Text["S/G2", {-0.2, -1.08}]}],
    Graphics[{RGBColor[1, 0.5, 0], Text["G2/M", {1, -0.6}]}],
    Graphics[{RGBColor[1, 0.5, 0], Dashing[{0.03, 0.02}],
      {Thickness[0.009], Circle[{0, 0}, 1, {2 * Pi + c16, 2 * Pi}]}}],
    Graphics[{RGBColor[1, 0.5, 0], Dashing[{0.03, 0.02}],
      {Thickness[0.009], Circle[{0, 0}, 1, {0, c17}]}}],
    Graphics[{RGBColor[1, 1, 0], Dashing[{0.03, 0.02}],
      {Thickness[0.009], Circle[{0, 0}, 1, {c17, c14}]}}],
    Graphics[{RGBColor[0, 0.5, 0], Dashing[{0.03, 0.02}],
      {Thickness[0.009], Circle[{0, 0}, 1, {c14, Pi + c17}]}}],
    Graphics[{RGBColor[0, 0, 1], Dashing[{0.03, 0.02}],
      {Thickness[0.009], Circle[{0, 0}, 1, {Pi + c17, Pi + c15}]}}],
    Graphics[{RGBColor[1, 0, 0], Dashing[{0.03, 0.02}],
      {Thickness[0.009], Circle[{0, 0}, 1, {Pi + c15, 2 * Pi + c16}]}}],
    Graphics[{RGBColor[1, 0.5, 0], Dashing[{0.03, 0.02}],
      {Thickness[0.009], Circle[{0, 0}, 0.5, {2 * Pi + c16, 2 * Pi}]}}],
    Graphics[{RGBColor[1, 0.5, 0], Dashing[{0.03, 0.02}],
      {Thickness[0.009], Circle[{0, 0}, 0.5, {0, c17}]}}],
    Graphics[{RGBColor[1, 1, 0], Dashing[{0.03, 0.02}],
      {Thickness[0.009], Circle[{0, 0}, 0.5, {c17, c14}]}}],
    Graphics[{RGBColor[0, 0.5, 0], Dashing[{0.03, 0.02}],
      {Thickness[0.009], Circle[{0, 0}, 0.5, {c14, Pi + c17}]}}],
    Graphics[{RGBColor[0, 0, 1], Dashing[{0.03, 0.02}],
      {Thickness[0.009], Circle[{0, 0}, 0.5, {Pi + c17, Pi + c15}]}}],
    Graphics[{RGBColor[1, 0, 0], Dashing[{0.03, 0.02}],
      {Thickness[0.009], Circle[{0, 0}, 0.5, {Pi + c15, 2 * Pi + c16}]}}],
    Graphics[{RGBColor[0, 0.5, 0], PointSize[0.03], Point[CLN1]}],
    Graphics[{RGBColor[0, 0.5, 0], Text["CLN1", CLN1 + {0.2, 0}]}],
    Graphics[{RGBColor[0, 0.5, 0], PointSize[0.03], Point[CLN2]}],
    Graphics[{RGBColor[0, 0.5, 0], Text["CLN2", CLN2 + {0.2, 0}]}],
    Graphics[{RGBColor[0, 0, 0], PointSize[0.03], Point[CLN3]}],
    Graphics[{RGBColor[0, 0, 0], Text["CLN3", CLN3 + {-0.2, 0}]}],
    Graphics[{RGBColor[1, 0.5, 0], PointSize[0.03], Point[CLB1]}],
    Graphics[{RGBColor[1, 0.5, 0], Text["CLB1", CLB1 + {-0.18, 0}]}],
    Graphics[{RGBColor[1, 0.5, 0], PointSize[0.03], Point[CLB2]}],
    Graphics[{RGBColor[1, 0.5, 0], Text["CLB2", CLB2 + {-0.18, 0}]}],
    Graphics[{RGBColor[0, 0, 1], PointSize[0.03], Point[CLB3]}],
    Graphics[{RGBColor[0, 0, 1], Text["CLB3", CLB3 + {-0.2, 0}]}],
    Graphics[{RGBColor[0, 0, 1], PointSize[0.03], Point[CLB4]}],
    Graphics[{RGBColor[0, 0, 1], Text["CLB4", CLB4 + {0.2, 0}]}],
    Graphics[{RGBColor[0, 0.5, 0], PointSize[0.03], Point[CLB5]}],
    Graphics[{RGBColor[0, 0.5, 0], Text["CLB5", CLB5 + {0, 0.1}]}],
    Graphics[{RGBColor[0, 0, 0], Dashing[{0.03, 0.02}], Line[{{0, 1.15}, {0, -1.15}}]}],
    Graphics[{RGBColor[0, 0, 0], Dashing[{0.03, 0.02}], Line[{{1.15, 0}, {-1.15, 0}}]}]},
   AspectRatio -> 1,
   PlotRange -> {{-1.15, 1.15}, {-1.15, 1.15}},
   Frame -> True,
   FrameTicks -> False,
   FrameLabel -> {None, None, None, None},
   GridLines -> {None, None},
   DisplayFunction -> Identity];
p = FullGraphics[p];
p[[1, 2]] = p[[1, 2]] /. Text[labely, {b_, c_}, {1., 0.}] ->
    Text[labely, {-1.18, 0}, {0, 0}, {0, 1}];
C2 = Show[p,
   AspectRatio -> 1,
   PlotRange -> All,
   DisplayFunction -> Identity];
```

```
(* Sort Human Data *)

genes = genes3;
arraynames = arraynames3;
genenames = TakeColumns[genenames3, {1, 3}];

(* Sort Human Arrays *)

(* Center Genelets and Calculate Contributions of Arraylets to Arrays *)

average = Table[1, {a, 1, arrays}];
average = N[average / Sqrt[Dot[average, average]]];

arraycontributions13 =
  (genelets[[13]] - N[Outer[Times, Dot[genelets, average], average]][[13]]) * d3[[13, 13]];
arraycontributions14 =
  (genelets[[14]] - N[Outer[Times, Dot[genelets, average], average]][[14]]) * d3[[14, 14]];
arraycontributions15 =
  (genelets[[15]] - N[Outer[Times, Dot[genelets, average], average]][[15]]) * d3[[15, 15]];
arraycontributions16 =
  (genelets[[16]] - N[Outer[Times, Dot[genelets, average], average]][[16]]) * d3[[16, 16]];
arraycontributions17 =
  (genelets[[17]] - N[Outer[Times, Dot[genelets, average], average]][[17]]) * d3[[17, 17]];


(* Project Arrays from 5 D Arraylets Subspace Onto 2 D Subspace  *)

coordinates = Table[{
    (Dot[genelets[[13]], x] * arraycontributions13[[a]] +
       Dot[genelets[[14]], x] * arraycontributions14[[a]] +
       Dot[genelets[[15]], x] * arraycontributions15[[a]] +
       Dot[genelets[[16]], x] * arraycontributions16[[a]] +
       Dot[genelets[[17]], x] * arraycontributions17[[a]]) /
     Sqrt[(a13 * arraycontributions13[[a]])^2 + (a14 * arraycontributions14[[a]])^2 +
       (a15 * arraycontributions15[[a]])^2 + (a16 * arraycontributions16[[a]])^2 +
       (a17 * arraycontributions17[[a]])^2 +
       c1314 * Abs[(a13 * arraycontributions13[[a]]) * (a14 * arraycontributions14[[a]])] +
       c1315 * Abs[(a13 * arraycontributions13[[a]]) * (a15 * arraycontributions15[[a]])] +
       c1316 * Abs[(a13 * arraycontributions13[[a]]) * (a16 * arraycontributions16[[a]])] +
       c1317 * Abs[(a13 * arraycontributions13[[a]]) * (a17 * arraycontributions17[[a]])] +
       c1415 * Abs[(a14 * arraycontributions14[[a]]) * (a15 * arraycontributions15[[a]])] +
       c1416 * Abs[(a14 * arraycontributions14[[a]]) * (a16 * arraycontributions16[[a]])] +
       c1417 * Abs[(a14 * arraycontributions14[[a]]) * (a17 * arraycontributions17[[a]])] +
       c1516 * Abs[(a15 * arraycontributions15[[a]]) * (a16 * arraycontributions16[[a]])] +
       c1517 * Abs[(a15 * arraycontributions15[[a]]) * (a17 * arraycontributions17[[a]])] +
       c1617 * Abs[(a16 * arraycontributions16[[a]]) * (a17 * arraycontributions17[[a]])]],
    (Dot[genelets[[13]], y] * arraycontributions13[[a]] +
       Dot[genelets[[14]], y] * arraycontributions14[[a]] +
       Dot[genelets[[15]], y] * arraycontributions15[[a]] +
       Dot[genelets[[16]], y] * arraycontributions16[[a]] +
       Dot[genelets[[17]], y] * arraycontributions17[[a]]) /
     Sqrt[(a13 * arraycontributions13[[a]])^2 + (a14 * arraycontributions14[[a]])^2 +
       (a15 * arraycontributions15[[a]])^2 + (a16 * arraycontributions16[[a]])^2 +
       (a17 * arraycontributions17[[a]])^2 +
       c1314 * Abs[(a13 * arraycontributions13[[a]]) * (a14 * arraycontributions14[[a]])] +
       c1315 * Abs[(a13 * arraycontributions13[[a]]) * (a15 * arraycontributions15[[a]])] +
       c1316 * Abs[(a13 * arraycontributions13[[a]]) * (a16 * arraycontributions16[[a]])] +
       c1317 * Abs[(a13 * arraycontributions13[[a]]) * (a17 * arraycontributions17[[a]])] +
       c1415 * Abs[(a14 * arraycontributions14[[a]]) * (a15 * arraycontributions15[[a]])] +
       c1416 * Abs[(a14 * arraycontributions14[[a]]) * (a16 * arraycontributions16[[a]])] +
       c1417 * Abs[(a14 * arraycontributions14[[a]]) * (a17 * arraycontributions17[[a]])] +
       c1516 * Abs[(a15 * arraycontributions15[[a]]) * (a16 * arraycontributions16[[a]])] +
       c1517 * Abs[(a15 * arraycontributions15[[a]]) * (a17 * arraycontributions17[[a]])] +
       c1617 * Abs[(a16 * arraycontributions16[[a]]) * (a17 * arraycontributions17[[a]])]]},
   {a, 1, arrays}];
```

```
(* Create Parametric Plot of Human Arrays Projected Onto 2 D Subspace *)

Clear[points];
points1 = {Point[coordinates[[5]]], Point[coordinates[[6]]],
   Point[coordinates[[12]]], Point[coordinates[[13]]]};
points2 = {Point[coordinates[[7]]], Point[coordinates[[8]]],
   Point[coordinates[[15]]], Point[coordinates[[14]]], Point[coordinates[[16]]]};
points3 = {Point[coordinates[[1]]], Point[coordinates[[2]]], Point[coordinates[[9]]]};
points4 = {Point[coordinates[[3]]], Point[coordinates[[10]]], Point[coordinates[[17]]]};
points5 = {Point[coordinates[[4]]], Point[coordinates[[11]]]};
textcoordinates = coordinates;
Do[
  textcoordinates[[a, 1]] = If[
    textcoordinates[[a, 1]] > 0,
    textcoordinates[[a, 1]] + 0.095,
    textcoordinates[[a, 1]] - 0.1],
  {a, 1, 9}];
Do[textcoordinates[[a, 1]] =
   If[
    textcoordinates[[a, 1]] > 0,
    textcoordinates[[a, 1]] + 0.11,
    textcoordinates[[a, 1]] - 0.12],
  {a, 10, arrays}];
textcoordinates[[1]] = textcoordinates[[1]] - {0.095, -0.095};
textcoordinates[[6]] = textcoordinates[[6]] + {0.2, 0};
textcoordinates[[10]] = textcoordinates[[10]] - {0.1, -0.095};
textcoordinates[[11]] = textcoordinates[[11]] - {0.11, 0.1};
textcoordinates[[12]] = textcoordinates[[12]] + {0.12, -0.12};
textcoordinates[[14]] = textcoordinates[[14]] + {0.11, 0.1};
textcoordinates[[15]] = textcoordinates[[15]] + {0, 0.05};
textcoordinates[[16]] = textcoordinates[[16]] + {0.24, 0};
textcoordinates[[17]] = textcoordinates[[17]] + {-0.25, 0};

texts = Table[Text[a, textcoordinates[[a]]], {a, 1, arrays}];
zerophase = N[ArcTan[coordinates[[1, 2]] / (coordinates[[1, 1]])]];
radius = Sqrt[coordinates[[2, 1]]^2 + coordinates[[2, 2]]^2];
```

```
p = Show[{
    Graphics[{RGBColor[0, 0, 0], Text["(c)", {-1.22, 1.15}, {-1, 0}]}],
    Graphics[{RGBColor[0, 0, 0], Text["x", {1.22, -0.075}, {1, 0}]}],
    Graphics[{RGBColor[0, 0, 0], Text["y", {-0.065, 1.22}, {0, 1}]}],
    Graphics[{RGBColor[0, 0, 1], Text["S", {0.97, -0.52}]}],
    Graphics[{RGBColor[1, 0, 0], Text["G2", {0.88, 0.7}]}],
    Graphics[{RGBColor[1, 0.5, 0], Text["G2/M", {0.3, 1.08}]}],
    Graphics[{RGBColor[0, 0, 0], Text["M/G1", {-1.02, 0.55}]}],
    Graphics[{RGBColor[0, 0.5, 0], Text["G1/S", {-0.878, -0.81}]}],
    Graphics[{RGBColor[1, 1, 0], PointSize[0.035], points1}],
    Graphics[{RGBColor[0, 0.5, 0], PointSize[0.035], points2}],
    Graphics[{RGBColor[0, 0, 1], PointSize[0.035], points3}],
    Graphics[{RGBColor[1, 0, 0], PointSize[0.035], points4}],
    Graphics[{RGBColor[1, 0.5, 0], PointSize[0.035], points5}],
    Graphics[{RGBColor[0, 0, 0], Text["u3,17", {1.235, 0.5}, {1, 0}]}],
    Graphics[{RGBColor[0, 0, 0], Text["u3,16", {0.5, -1.12}, {-1, 0}]}],
    Graphics[{RGBColor[0, 0, 0], Text["u3,15", {0.7, 1.12}, {-1, 0}]}],
    Graphics[{RGBColor[0, 0, 0], Text["u3,14", {-0.45, 1.12}, {1, 0}]}],
    Graphics[{RGBColor[0, 0, 0], Text["u3,13", {-0.025, -1.12}, {1, 0}]}],
    Graphics[texts],
    Graphics[{RGBColor[0, 0, 0], Dashing[{0.03, 0.02}], Circle[{0, 0}, 0.5]}],
    Graphics[{RGBColor[0, 0, 0], Dashing[{0.03, 0.02}], Circle[{0, 0}, 1]}],
    Graphics[{RGBColor[0, 0, 0], Dashing[{0.03, 0.02}], Line[{{0, 1.25}, {0, -1.25}}]}],
    Graphics[{RGBColor[0, 0, 0], Dashing[{0.03, 0.02}], Line[{{1.25, 0}, {-1.25, 0}}]}],
    Graphics[{RGBColor[0, 0, 0], Arrow[{1.25 / Tan[c13], 1.25}, {-1.25 / Tan[c13], -1.25},
       HeadCenter -> 0.5, HeadLength -> 0.035, HeadWidth -> 0.75]}],
    Graphics[{RGBColor[0, 0, 0], Arrow[{-1.25 / Tan[c14 - Pi], -1.25}, {1.25 / Tan[c14 - Pi], 1.25},
       HeadCenter -> 0.5, HeadLength -> 0.035, HeadWidth -> 0.75]}],
    Graphics[{RGBColor[0, 0, 0], Arrow[{-1.25 / Tan[c15], -1.25}, {1.25 / Tan[c15], 1.25},
       HeadCenter -> 0.5, HeadLength -> 0.035, HeadWidth -> 0.75]}],
    Graphics[{RGBColor[0, 0, 0], Arrow[{1.25 / Tan[c16], 1.25}, {-1.25 / Tan[c16], -1.25},
       HeadCenter -> 0.5, HeadLength -> 0.035, HeadWidth -> 0.75]}],
    Graphics[{RGBColor[0, 0, 0], Arrow[{-1.25, -1.25 * Tan[c17]}, {1.25, 1.25 * Tan[c17]},
       HeadCenter -> 0.5, HeadLength -> 0.035, HeadWidth -> 0.75]}]},
   AspectRatio -> 1,
   PlotRange -> {{-1.25, 1.25}, {-1.25, 1.25}},
   Frame -> True,
   FrameTicks -> False,
   FrameLabel -> {None, None, None, None},
   GridLines -> {None, None},
   DisplayFunction -> Identity];
p = FullGraphics[p];
p[[1, 2]] = p[[1, 2]] /.
   Text[labely, {b_, c_}, {1., 0.}] ->
    Text[labely, {-1.18, 0}, {0, 0}, {0, 1}];
p4 = Show[p,
   AspectRatio -> 1,
   PlotRange -> All,
   DisplayFunction -> Identity];
```

```
(* Sort Human Genes *)

(* Center Arraylets and Calculate Contributions of Genelets to Genes *)

centerarraylets = Transpose[arraylets3];
average = Table[1, {a, 1, genes}];
average = N[average / Sqrt[Dot[average, average]]];
centerarraylets = centerarraylets - N[Outer[Times, Dot[centerarraylets, average], average]];
centerarraylets = Transpose[centerarraylets];
genecontributions = Transpose[Dot[centerarraylets, d3]];

(* Project Genes from 5 D Genelets Subspace Onto 2 D Subspace  *)

coordinates = Table[{
    (Dot[genelets[[13]], x] * genecontributions[[13, a]] +
       Dot[genelets[[14]], x] * genecontributions[[14, a]] +
       Dot[genelets[[15]], x] * genecontributions[[15, a]] +
       Dot[genelets[[16]], x] * genecontributions[[16, a]] +
       Dot[genelets[[17]], x] * genecontributions[[17, a]]) /
     Sqrt[(a13 * genecontributions[[13, a]])^2 + (a14 * genecontributions[[14, a]])^2 +
       (a15 * genecontributions[[15, a]])^2 + (a16 * genecontributions[[16, a]])^2 +
       (a17 * genecontributions[[17, a]])^2 +
       c1314 * Abs[(a13 * genecontributions[[13, a]]) * (a14 * genecontributions[[14, a]])] +
       c1315 * Abs[(a13 * genecontributions[[13, a]]) * (a15 * genecontributions[[15, a]])] +
       c1316 * Abs[(a13 * genecontributions[[13, a]]) * (a16 * genecontributions[[16, a]])] +
       c1317 * Abs[(a13 * genecontributions[[13, a]]) * (a17 * genecontributions[[17, a]])] +
       c1415 * Abs[(a14 * genecontributions[[14, a]]) * (a15 * genecontributions[[15, a]])] +
       c1416 * Abs[(a14 * genecontributions[[14, a]]) * (a16 * genecontributions[[16, a]])] +
       c1417 * Abs[(a14 * genecontributions[[14, a]]) * (a17 * genecontributions[[17, a]])] +
       c1516 * Abs[(a15 * genecontributions[[15, a]]) * (a16 * genecontributions[[16, a]])] +
       c1517 * Abs[(a15 * genecontributions[[15, a]]) * (a17 * genecontributions[[17, a]])] +
       c1617 * Abs[(a16 * genecontributions[[16, a]]) * (a17 * genecontributions[[17, a]])]],
    (Dot[genelets[[13]], y] * genecontributions[[13, a]] +
       Dot[genelets[[14]], y] * genecontributions[[14, a]] +
       Dot[genelets[[15]], y] * genecontributions[[15, a]] +
       Dot[genelets[[16]], y] * genecontributions[[16, a]] +
       Dot[genelets[[17]], y] * genecontributions[[17, a]]) /
     Sqrt[(a13 * genecontributions[[13, a]])^2 + (a14 * genecontributions[[14, a]])^2 +
       (a15 * genecontributions[[15, a]])^2 + (a16 * genecontributions[[16, a]])^2 +
       (a17 * genecontributions[[17, a]])^2 +
       c1314 * Abs[(a13 * genecontributions[[13, a]]) * (a14 * genecontributions[[14, a]])] +
       c1315 * Abs[(a13 * genecontributions[[13, a]]) * (a15 * genecontributions[[15, a]])] +
       c1316 * Abs[(a13 * genecontributions[[13, a]]) * (a16 * genecontributions[[16, a]])] +
       c1317 * Abs[(a13 * genecontributions[[13, a]]) * (a17 * genecontributions[[17, a]])] +
       c1415 * Abs[(a14 * genecontributions[[14, a]]) * (a15 * genecontributions[[15, a]])] +
       c1416 * Abs[(a14 * genecontributions[[14, a]]) * (a16 * genecontributions[[16, a]])] +
       c1417 * Abs[(a14 * genecontributions[[14, a]]) * (a17 * genecontributions[[17, a]])] +
       c1516 * Abs[(a15 * genecontributions[[15, a]]) * (a16 * genecontributions[[16, a]])] +
       c1517 * Abs[(a15 * genecontributions[[15, a]]) * (a17 * genecontributions[[17, a]])] +
       c1617 * Abs[(a16 * genecontributions[[16, a]]) * (a17 * genecontributions[[17, a]])]]},
   {a, 1, genes}];

(* Create Parametric Plot of Cell Cycle Genes Projected Onto 2 D Subspace *)

list = AppendRows[TakeColumns[genenames, {1, 1}], TakeColumns[genenames, {3, 3}]];
stages = {"M/G1", "G1/S", "S", "G2", "G2/M"};
points = {points1, points2, points3, points4, points5};
radii = {radii1, radii2, radii3, radii4, radii5};
```

```
Do[{
  position = Position[list, stages[[b]]],
  points[[b]] = Table[Point[coordinates[[position[[a]][[1]]]]], {a, 1, Dimensions[position][[1]]}],
  radii[[b]] =
   Table[Sqrt[coordinates[[position[[a]][[1]]]][[1]]^2 + coordinates[[position[[a]][[1]]]][[2]]^2],
    {a, 1, Dimensions[position][[1]]}]},
 {b, 1, Dimensions[stages][[1]]}]
radii = Sort[Flatten[radii]];
N[Round[radii[[155]] * 100] / 100]
N[Round[radii[[156]] * 100] / 100]
```

```
0.49
```

```
0.5
```

```
p = Show[{
    Graphics[{RGBColor[0, 0, 0], Text["(f)", {-1.22, 1.15}, {-1, 0}]}],
    Graphics[{RGBColor[0, 0, 0], Text["x", {1.22, -0.075}, {1, 0}]}],
    Graphics[{RGBColor[0, 0, 0], Text["y", {-0.065, 1.22}, {0, 1}]}],
    Graphics[{RGBColor[0, 0, 1], Text["S", {0.97, -0.52}]}],
    Graphics[{RGBColor[1, 0, 0], Text["G2", {0.88, 0.7}]}],
    Graphics[{RGBColor[1, 0.5, 0], Text["G2/M", {0.3, 1.08}]}],
    Graphics[{RGBColor[0, 0, 0], Text["M/G1", {-1.02, 0.55}]}],
    Graphics[{RGBColor[0, 0.5, 0], Text["G1/S", {-0.878, -0.81}]}],
    Graphics[{RGBColor[1, 0.5, 0], PointSize[0.02], points[[5]]}],
    Graphics[{RGBColor[1, 1, 0], PointSize[0.02], points[[1]]}],
    Graphics[{RGBColor[1, 0, 0], PointSize[0.02], points[[4]]}],
    Graphics[{RGBColor[0, 0.5, 0], PointSize[0.02], points[[2]]}],
    Graphics[{RGBColor[0, 0, 1], PointSize[0.02], points[[3]]}],
    Graphics[{RGBColor[0, 0, 0], Text["v17", {1.235, 0.5}, {1, 0}]}],
    Graphics[{RGBColor[0, 0, 0], Text["v16", {0.5, -1.12}, {-1, 0}]}],
    Graphics[{RGBColor[0, 0, 0], Text["v15", {0.7, 1.12}, {-1, 0}]}],
    Graphics[{RGBColor[0, 0, 0], Text["v14", {-0.45, 1.12}, {1, 0}]}],
    Graphics[{RGBColor[0, 0, 0], Text["v13", {-0.025, -1.12}, {1, 0}]}],
    Graphics[{RGBColor[0, 0, 0], Dashing[{0.03, 0.02}], Circle[{0, 0}, 0.5]}],
    Graphics[{RGBColor[0, 0, 0], Dashing[{0.03, 0.02}], Circle[{0, 0}, 1]}],
    Graphics[{RGBColor[0, 0, 0], Dashing[{0.03, 0.02}], Line[{{0, 1.25}, {0, -1.25}}]}],
    Graphics[{RGBColor[0, 0, 0], Dashing[{0.03, 0.02}], Line[{{1.25, 0}, {-1.25, 0}}]}],
    Graphics[{RGBColor[0, 0, 0], Arrow[{1.25 / Tan[c13], 1.25}, {-1.25 / Tan[c13], -1.25},
       HeadCenter -> 0.5, HeadLength -> 0.035, HeadWidth -> 0.75]}],
    Graphics[{RGBColor[0, 0, 0], Arrow[{-1.25 / Tan[c14 - Pi], -1.25}, {1.25 / Tan[c14 - Pi], 1.25},
       HeadCenter -> 0.5, HeadLength -> 0.035, HeadWidth -> 0.75]}],
    Graphics[{RGBColor[0, 0, 0], Arrow[{-1.25 / Tan[c15], -1.25}, {1.25 / Tan[c15], 1.25},
       HeadCenter -> 0.5, HeadLength -> 0.035, HeadWidth -> 0.75]}],
    Graphics[{RGBColor[0, 0, 0], Arrow[{1.25 / Tan[c16], 1.25}, {-1.25 / Tan[c16], -1.25},
       HeadCenter -> 0.5, HeadLength -> 0.035, HeadWidth -> 0.75]}],
    Graphics[{RGBColor[0, 0, 0], Arrow[{-1.25, -1.25 * Tan[c17]}, {1.25, 1.25 * Tan[c17]},
       HeadCenter -> 0.5, HeadLength -> 0.035, HeadWidth -> 0.75]}]},
   AspectRatio -> 1,
   PlotRange -> {{-1.25, 1.25}, {-1.25, 1.25}},
   Frame -> True,
   FrameTicks -> False,
   FrameLabel -> {None, None, None, None},
   GridLines -> {None, None},
   DisplayFunction -> Identity];
p = FullGraphics[p];
p[[1, 2]] = p[[1, 2]] /.
   Text[labely, {b_, c_}, {1., 0.}] ->
    Text[labely, {-1.18, 0}, {0, 0}, {0, 1}];
p5 = Show[p,
   AspectRatio -> 1,
   PlotRange -> All,
   DisplayFunction -> Identity];
```

```
R[theta_] = {{Cos[theta], Sin[theta]}, {-Sin[theta], Cos[theta]}};
z = Transpose[{{0.4, 0}, {1.1, 0}, {0.75, 0.25}}];
polypoints[theta_] = Transpose[Dot[R[theta], z]];
p = Show[{
    Graphics[{RGBColor[0, 0, 0], Text["(i)", {-1.22, 1.15}, {-1, 0}]}],
    Graphics[{RGBColor[0, 0, 0], Text["x", {1.22, -0.075}, {1, 0}]}],
    Graphics[{RGBColor[0, 0, 0], Text["y", {-0.065, 1.22}, {0, 1}]}],
    Graphics[{RGBColor[0, 0, 1], Text["S", {0.97, -0.52}]}],
    Graphics[{RGBColor[1, 0, 0], Text["G2", {0.88, 0.7}]}],
    Graphics[{RGBColor[1, 0.5, 0], Text["G2/M", {0.3, 1.08}]}],
    Graphics[{RGBColor[0, 0, 0], Text["M/G1", {-1.02, 0.55}]}],
    Graphics[{RGBColor[0, 0.5, 0], Text["G1/S", {-0.878, -0.81}]}],
    Graphics[{RGBColor[0, 0, 0], Text["17", {1.235, 0.5}, {1, 0}]}],
    Graphics[{RGBColor[0, 0, 0], Text["16", {0.5, -1.12}, {-1, 0}]}],
    Graphics[{RGBColor[0, 0, 0], Text["15", {0.7, 1.12}, {-1, 0}]}],
    Graphics[{RGBColor[0, 0, 0], Text["14", {-0.45, 1.12}, {1, 0}]}],
    Graphics[{RGBColor[0, 0, 0], Text["13", {-0.025, -1.12}, {1, 0}]}],
    Graphics[{RGBColor[0, 0, 1], Disk[{0, 0}, 1, {Pi + c16, 2 * Pi}]}],
    Graphics[{RGBColor[0, 0, 1], Disk[{0, 0}, 1, {0, c17}]}],
    Graphics[{RGBColor[1, 0, 0], Disk[{0, 0}, 1, {c17, c15}]}],
    Graphics[{RGBColor[1, 0.5, 0], Disk[{0, 0}, 1, {c15, c14}]}],
    Graphics[{RGBColor[1, 1, 0], Disk[{0, 0}, 1, {c14, Pi + c17}]}],
    Graphics[{RGBColor[0, 0.5, 0], Disk[{0, 0}, 1, {Pi + c17, 2 * Pi + c16}]}],
    Graphics[{RGBColor[1, 1, 1], Disk[{0, 0}, 0.5]}],
    Graphics[{RGBColor[0, 0, 1], Polygon[polypoints[2 * Pi - c17]]}],
    Graphics[{RGBColor[1, 0, 0], Polygon[polypoints[2 * Pi - c15]]}],
    Graphics[{RGBColor[1, 0.5, 0], Polygon[polypoints[2 * Pi - c14]]}],
    Graphics[{RGBColor[1, 1, 0], Polygon[polypoints[Pi - c17]]}],
    Graphics[{RGBColor[0, 0.5, 0], Polygon[polypoints[2 * Pi - c16]]}],
    Graphics[{RGBColor[0, 0, 0], Dashing[{0.03, 0.02}], Line[{{0, 1.25}, {0, -1.25}}]}],
    Graphics[{RGBColor[0, 0, 0], Dashing[{0.03, 0.02}], Line[{{1.25, 0}, {-1.25, 0}}]}],
    Graphics[{RGBColor[0, 0, 0], Arrow[{1.25 / Tan[c13], 1.25}, {-1.25 / Tan[c13], -1.25},
       HeadCenter -> 0.5, HeadLength -> 0.035, HeadWidth -> 0.75]}],
    Graphics[{RGBColor[0, 0, 0], Arrow[{-1.25 / Tan[c14 - Pi], -1.25}, {1.25 / Tan[c14 - Pi], 1.25},
       HeadCenter -> 0.5, HeadLength -> 0.035, HeadWidth -> 0.75]}],
    Graphics[{RGBColor[0, 0, 0], Arrow[{-1.25 / Tan[c15], -1.25}, {1.25 / Tan[c15], 1.25},
       HeadCenter -> 0.5, HeadLength -> 0.035, HeadWidth -> 0.75]}],
    Graphics[{RGBColor[0, 0, 0], Arrow[{1.25 / Tan[c16], 1.25}, {-1.25 / Tan[c16], -1.25},
       HeadCenter -> 0.5, HeadLength -> 0.035, HeadWidth -> 0.75]}],
    Graphics[{RGBColor[0, 0, 0], Arrow[{-1.25, -1.25 * Tan[c17]}, {1.25, 1.25 * Tan[c17]},
       HeadCenter -> 0.5, HeadLength -> 0.035, HeadWidth -> 0.75]}]},
   AspectRatio -> 1,
   PlotRange -> {{-1.25, 1.25}, {-1.25, 1.25}},
   Frame -> True,
   FrameTicks -> False,
   FrameLabel -> {None, None, None, None},
   GridLines -> {None, None},
   DisplayFunction -> Identity];
p = FullGraphics[p];
p[[1, 2]] = p[[1, 2]] /.
   Text[labely, {b_, c_}, {1., 0.}] ->
    Text[labely, {-1.18, 0}, {0, 0}, {0, 1}];
p6 = Show[p,
   AspectRatio -> 1,
   PlotRange -> All,
   DisplayFunction -> Identity];
Clear[z];
```

```
(* CCNA2 and CCNB2 Projected Onto 2 D Subspace *)

stages = {"M/G1", "G1/S", "S", "G2", "G2/M"};
CCNB2 = coordinates[[Position[genenames, "IMAGE:856289"][[1, 1]]]];
CCNA2a = coordinates[[Position[genenames, "IMAGE:950690"][[1, 1]]]];
CCNA2b = coordinates[[Position[genenames, "IMAGE:814270"][[1, 1]]]];

p = Show[{
    Graphics[{RGBColor[0, 0, 0], Text["(g)", {-1, 1.05}]}],
    Graphics[{RGBColor[0, 0, 0], Text["Human", {1.1, -1.05}, {1, 0}]}],
    Graphics[{RGBColor[0, 0, 0], Text["x", {1.13, -0.07}, {1, 0}]}],
    Graphics[{RGBColor[0, 0, 0], Text["y", {-0.055, 1.145}, {0, 1}]}],
    Graphics[{RGBColor[0, 0, 0], Text["M/G1", {-0.825, 0.825}]}],
    Graphics[{RGBColor[0, 0.5, 0], Text["G1/S", {-0.878, -0.81}]}],
    Graphics[{RGBColor[1, 0, 0], Text["G2", {0.88, 0.7}]}],
    Graphics[{RGBColor[1, 0.5, 0], Text["G2/M", {0.3, 1.08}]}],
    Graphics[{RGBColor[0, 0, 1], Text["S", {0.97, -0.52}]}],
    Graphics[
     {RGBColor[0, 0, 1], Dashing[{0.03, 0.02}], {Thickness[0.009], Circle[{0, 0}, 1, {2*Pi+c16, 2*Pi}]}}],
    Graphics[{RGBColor[0, 0, 1], Dashing[{0.03, 0.02}],
      {Thickness[0.009], Circle[{0, 0}, 1, {0, c17}]}}],
    Graphics[{RGBColor[1, 0, 0], Dashing[{0.03, 0.02}], {Thickness[0.009], Circle[{0, 0}, 1, {c17, c15}]}}],
    Graphics[
     {RGBColor[1, 0.5, 0], Dashing[{0.03, 0.02}], {Thickness[0.009], Circle[{0, 0}, 1, {c15, c14}]}}],
    Graphics[{RGBColor[1, 1, 0], Dashing[{0.03, 0.02}],
      {Thickness[0.009], Circle[{0, 0}, 1, {c14, Pi+c17}]}}],
    Graphics[{RGBColor[0, 0.5, 0], Dashing[{0.03, 0.02}],
      {Thickness[0.009], Circle[{0, 0}, 1, {Pi+c17, 2*Pi+c16}]}}],
    Graphics[{RGBColor[0, 0, 1], Dashing[{0.03, 0.02}],
      {Thickness[0.009], Circle[{0, 0}, 0.5, {2*Pi+c16, 2*Pi}]}}],
    Graphics[{RGBColor[0, 0, 1], Dashing[{0.03, 0.02}],
      {Thickness[0.009], Circle[{0, 0}, 0.5, {0, c17}]}}],
    Graphics[
     {RGBColor[1, 0, 0], Dashing[{0.03, 0.02}], {Thickness[0.009], Circle[{0, 0}, 0.5, {c17, c15}]}}],
    Graphics[{RGBColor[1, 0.5, 0], Dashing[{0.03, 0.02}],
      {Thickness[0.009], Circle[{0, 0}, 0.5, {c15, c14}]}}],
    Graphics[{RGBColor[1, 1, 0], Dashing[{0.03, 0.02}],
      {Thickness[0.009], Circle[{0, 0}, 0.5, {c14, Pi+c17}]}}],
    Graphics[{RGBColor[0, 0.5, 0], Dashing[{0.03, 0.02}],
      {Thickness[0.009], Circle[{0, 0}, 0.5, {Pi+c17, 2*Pi+c16}]}}],
    Graphics[{RGBColor[1, 0.5, 0], PointSize[0.03], Point[CCNB2]}],
    Graphics[{RGBColor[1, 0.5, 0], Text["CCNB2", CCNB2+{0.02, -0.15}]}],
    Graphics[{RGBColor[1, 0, 0], PointSize[0.03], Point[CCNA2a]}],
    Graphics[{RGBColor[1, 0, 0], PointSize[0.03], Point[CCNA2b]}],
    Graphics[{RGBColor[1, 0, 0], Text["CCNA2", CCNA2a+{0.23, 0.02}]}],
    Graphics[{RGBColor[1, 0, 0], Text["CCNA2", CCNA2b+{0, 0.1}]}],
    Graphics[{RGBColor[0, 0, 0], Dashing[{0.03, 0.02}], Line[{{0, 1.15}, {0, -1.15}}]}],
    Graphics[{RGBColor[0, 0, 0], Dashing[{0.03, 0.02}], Line[{{1.15, 0}, {-1.15, 0}}]}]},
   AspectRatio -> 1,
   PlotRange -> {{-1.15, 1.15}, {-1.15, 1.15}},
   Frame -> True,
   FrameTicks -> False,
   FrameLabel -> {None, None, None, None},
   GridLines -> {None, None},
   DisplayFunction -> Identity];
p = FullGraphics[p];
p[[1, 2]] = p[[1, 2]] /.
   Text[labely, {b_, c_}, {1., 0.}] →
    Text[labely, {-1.18, 0}, {0, 0}, {0, 1}];
C3 = Show[p,
   AspectRatio → 1,
   PlotRange -> All,
   DisplayFunction → Identity];
```

```
(* Display S. pombe, S. cerevisiae and Human Arrays & Genes Parametric Plots *)

Show[GraphicsArray[{{g1, g2, g3}, {t1, t2, t3}, {p4, p5, p6}}],
    GraphicsSpacing -> 0];
```

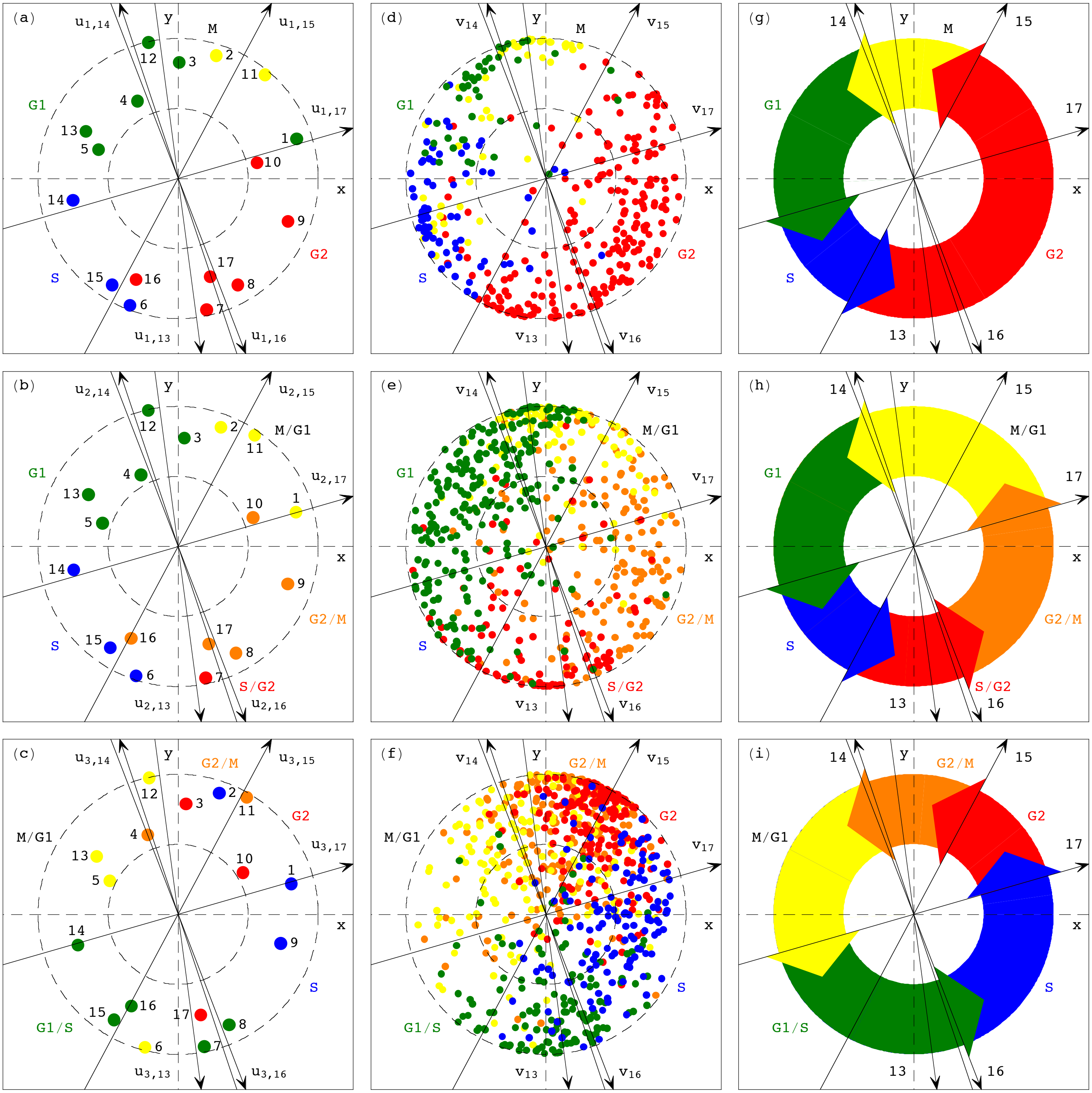

```
(* Display Ortholog Projection Parameter Graphs *)

Show[GraphicsArray[{{O1, O2}, {R1, R2}, {C1, C2, C3}}],
    GraphicsSpacing -> 0];
```

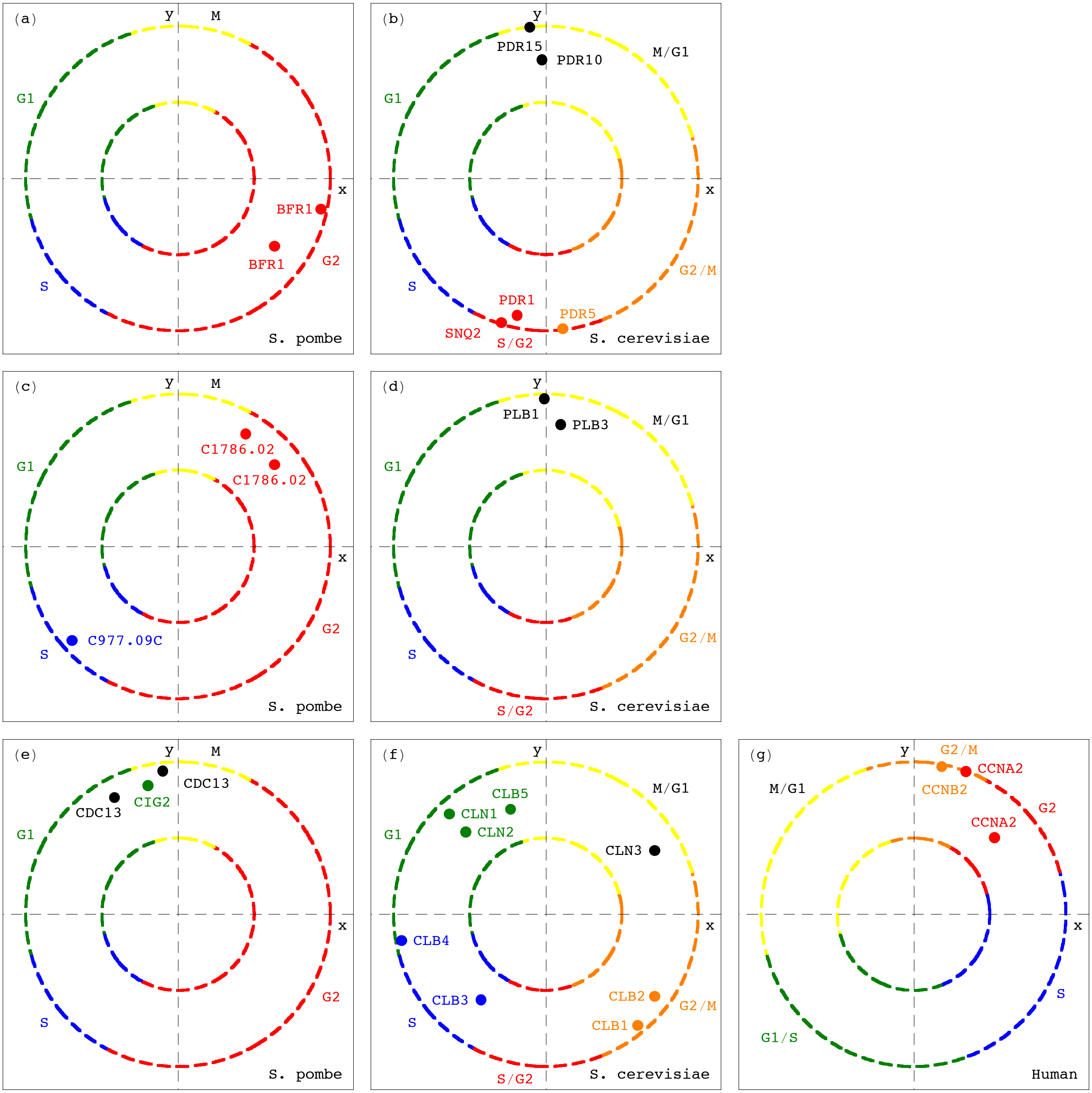

```
(* Reconstruct S. pombe, S. cerevisiae and Human Data in 5 D Common Cell Cycle Subspace *)

(* Sort S. pombe Genes *)

matrix = matrix1;
genes = genes1;
arraynames = arraynames1;
genenames = genenames1;

(* Center Arraylets and Calculate Contributions of Genelets to Genes *)

centerarraylets = Transpose[arraylets1];
average = Table[1, {a, 1, genes}];
average = N[average / Sqrt[Dot[average, average]]];
centerarraylets = centerarraylets - N[Outer[Times, Dot[centerarraylets, average], average]];
centerarraylets = Transpose[centerarraylets];
genecontributions = Transpose[Dot[centerarraylets, d1]];

(* Project Genes from 5 D Genelets Subspace Onto 2 D Subspace  *)

coordinates = Table[{(Dot[genelets[[13]], x] * genecontributions[[13, a]] +
        Dot[genelets[[14]], x] * genecontributions[[14, a]] +
        Dot[genelets[[15]], x] * genecontributions[[15, a]] +
        Dot[genelets[[16]], x] * genecontributions[[16, a]] +
        Dot[genelets[[17]], x] * genecontributions[[17, a]]) /
      Sqrt[(a13 * genecontributions[[13, a]]) ^ 2 + (a14 * genecontributions[[14, a]]) ^ 2 +
        (a15 * genecontributions[[15, a]]) ^ 2 + (a16 * genecontributions[[16, a]]) ^ 2 +
        (a17 * genecontributions[[17, a]]) ^ 2 +
        c1314 * Abs[(a13 * genecontributions[[13, a]]) * (a14 * genecontributions[[14, a]])] +
        c1315 * Abs[(a13 * genecontributions[[13, a]]) * (a15 * genecontributions[[15, a]])] +
        c1316 * Abs[(a13 * genecontributions[[13, a]]) * (a16 * genecontributions[[16, a]])] +
        c1317 * Abs[(a13 * genecontributions[[13, a]]) * (a17 * genecontributions[[17, a]])] +
        c1415 * Abs[(a14 * genecontributions[[14, a]]) * (a15 * genecontributions[[15, a]])] +
        c1416 * Abs[(a14 * genecontributions[[14, a]]) * (a16 * genecontributions[[16, a]])] +
        c1417 * Abs[(a14 * genecontributions[[14, a]]) * (a17 * genecontributions[[17, a]])] +
        c1516 * Abs[(a15 * genecontributions[[15, a]]) * (a16 * genecontributions[[16, a]])] +
        c1517 * Abs[(a15 * genecontributions[[15, a]]) * (a17 * genecontributions[[17, a]])] +
        c1617 * Abs[(a16 * genecontributions[[16, a]]) * (a17 * genecontributions[[17, a]])]],
     (Dot[genelets[[13]], y] * genecontributions[[13, a]] +
        Dot[genelets[[14]], y] * genecontributions[[14, a]] +
        Dot[genelets[[15]], y] * genecontributions[[15, a]] +
        Dot[genelets[[16]], y] * genecontributions[[16, a]] +
        Dot[genelets[[17]], y] * genecontributions[[17, a]]) /
      Sqrt[(a13 * genecontributions[[13, a]]) ^ 2 + (a14 * genecontributions[[14, a]]) ^ 2 +
        (a15 * genecontributions[[15, a]]) ^ 2 + (a16 * genecontributions[[16, a]]) ^ 2 +
        (a17 * genecontributions[[17, a]]) ^ 2 +
        c1314 * Abs[(a13 * genecontributions[[13, a]]) * (a14 * genecontributions[[14, a]])] +
        c1315 * Abs[(a13 * genecontributions[[13, a]]) * (a15 * genecontributions[[15, a]])] +
        c1316 * Abs[(a13 * genecontributions[[13, a]]) * (a16 * genecontributions[[16, a]])] +
        c1317 * Abs[(a13 * genecontributions[[13, a]]) * (a17 * genecontributions[[17, a]])] +
        c1415 * Abs[(a14 * genecontributions[[14, a]]) * (a15 * genecontributions[[15, a]])] +
        c1416 * Abs[(a14 * genecontributions[[14, a]]) * (a16 * genecontributions[[16, a]])] +
        c1417 * Abs[(a14 * genecontributions[[14, a]]) * (a17 * genecontributions[[17, a]])] +
        c1516 * Abs[(a15 * genecontributions[[15, a]]) * (a16 * genecontributions[[16, a]])] +
        c1517 * Abs[(a15 * genecontributions[[15, a]]) * (a17 * genecontributions[[17, a]])] +
        c1617 * Abs[(a16 * genecontributions[[16, a]]) * (a17 * genecontributions[[17, a]])]]},
   {a, 1, genes}];
```

```
(* Define the Initial Phase *)

phase = ArcTan[TakeColumns[coordinates, {1}], TakeColumns[coordinates, {2}]] / Degree;
Do[If[Negative[phase[[a]][[1]]], phase[[a]] = phase[[a]] + 360], {a, 1, genes}]

(* Sort Genes Accodring to Phases in 2 D Subspace *)

sortmatrix = AppendRows[phase, genenames, matrix];
sortmatrix = Sort[sortmatrix, OrderedQ[{{#1}, {#2}}] &];

(* Sorted S. pombe data *)

phase1 = TakeColumns[sortmatrix, {1, 1}];
matrix1 = TakeColumns[sortmatrix, {6, arrays + 5}];
genenames1 = TakeColumns[sortmatrix, {2, 5}];

(* Sort S. cerevisiae Genes *)

matrix = matrix2;
genes = genes2;
arraynames = arraynames2;
genenames = genenames2;

(* Center Arraylets and Calculate Contributions of Genelets to Genes *)

centerarraylets = Transpose[arraylets2];
average = Table[1, {a, 1, genes}];
average = N[average / Sqrt[Dot[average, average]]];
centerarraylets = centerarraylets - N[Outer[Times, Dot[centerarraylets, average], average]];
centerarraylets = Transpose[centerarraylets];
genecontributions = Transpose[Dot[centerarraylets, d2]];
```

```
(* Project Genes from 5 D Genelets Subspace Onto 2 D Subspace  *)

coordinates = Table[{(Dot[genelets[[13]], x] * genecontributions[[13, a]] +
       Dot[genelets[[14]], x] * genecontributions[[14, a]] +
       Dot[genelets[[15]], x] * genecontributions[[15, a]] +
       Dot[genelets[[16]], x] * genecontributions[[16, a]] +
       Dot[genelets[[17]], x] * genecontributions[[17, a]]) /
     Sqrt[(a13 * genecontributions[[13, a]]) ^ 2 + (a14 * genecontributions[[14, a]]) ^ 2 +
       (a15 * genecontributions[[15, a]]) ^ 2 + (a16 * genecontributions[[16, a]]) ^ 2 +
       (a17 * genecontributions[[17, a]]) ^ 2 +
       c1314 * Abs[(a13 * genecontributions[[13, a]]) * (a14 * genecontributions[[14, a]])] +
       c1315 * Abs[(a13 * genecontributions[[13, a]]) * (a15 * genecontributions[[15, a]])] +
       c1316 * Abs[(a13 * genecontributions[[13, a]]) * (a16 * genecontributions[[16, a]])] +
       c1317 * Abs[(a13 * genecontributions[[13, a]]) * (a17 * genecontributions[[17, a]])] +
       c1415 * Abs[(a14 * genecontributions[[14, a]]) * (a15 * genecontributions[[15, a]])] +
       c1416 * Abs[(a14 * genecontributions[[14, a]]) * (a16 * genecontributions[[16, a]])] +
       c1417 * Abs[(a14 * genecontributions[[14, a]]) * (a17 * genecontributions[[17, a]])] +
       c1516 * Abs[(a15 * genecontributions[[15, a]]) * (a16 * genecontributions[[16, a]])] +
       c1517 * Abs[(a15 * genecontributions[[15, a]]) * (a17 * genecontributions[[17, a]])] +
       c1617 * Abs[(a16 * genecontributions[[16, a]]) * (a17 * genecontributions[[17, a]])]],
    (Dot[genelets[[13]], y] * genecontributions[[13, a]] +
       Dot[genelets[[14]], y] * genecontributions[[14, a]] +
       Dot[genelets[[15]], y] * genecontributions[[15, a]] +
       Dot[genelets[[16]], y] * genecontributions[[16, a]] +
       Dot[genelets[[17]], y] * genecontributions[[17, a]]) /
     Sqrt[(a13 * genecontributions[[13, a]]) ^ 2 + (a14 * genecontributions[[14, a]]) ^ 2 +
       (a15 * genecontributions[[15, a]]) ^ 2 + (a16 * genecontributions[[16, a]]) ^ 2 +
       (a17 * genecontributions[[17, a]]) ^ 2 +
       c1314 * Abs[(a13 * genecontributions[[13, a]]) * (a14 * genecontributions[[14, a]])] +
       c1315 * Abs[(a13 * genecontributions[[13, a]]) * (a15 * genecontributions[[15, a]])] +
       c1316 * Abs[(a13 * genecontributions[[13, a]]) * (a16 * genecontributions[[16, a]])] +
       c1317 * Abs[(a13 * genecontributions[[13, a]]) * (a17 * genecontributions[[17, a]])] +
       c1415 * Abs[(a14 * genecontributions[[14, a]]) * (a15 * genecontributions[[15, a]])] +
       c1416 * Abs[(a14 * genecontributions[[14, a]]) * (a16 * genecontributions[[16, a]])] +
       c1417 * Abs[(a14 * genecontributions[[14, a]]) * (a17 * genecontributions[[17, a]])] +
       c1516 * Abs[(a15 * genecontributions[[15, a]]) * (a16 * genecontributions[[16, a]])] +
       c1517 * Abs[(a15 * genecontributions[[15, a]]) * (a17 * genecontributions[[17, a]])] +
       c1617 * Abs[(a16 * genecontributions[[16, a]]) * (a17 * genecontributions[[17, a]])]]},
   {a, 1, genes}];

(* Define the Initial Phase *)

phase = ArcTan[TakeColumns[coordinates, {1}], TakeColumns[coordinates, {2}]] / Degree;
Do[If[Negative[phase[[a]][[1]]], phase[[a]] = phase[[a]] + 360], {a, 1, genes}]

(* Sort Genes Accodring to Phases in 2 D Subspace *)

sortmatrix = AppendRows[phase, genenames, matrix];
sortmatrix = Sort[sortmatrix, OrderedQ[{{#1}, {#2}}] &];

(* Sorted S. cerevisiae data *)

phase2 = TakeColumns[sortmatrix, {1, 1}];
matrix2 = TakeColumns[sortmatrix, {5, arrays + 4}];
genenames2 = TakeColumns[sortmatrix, {2, 4}];
```

```
(* Sort Human Genes *)

matrix = matrix3;
genes = genes3;
arraynames = arraynames3;
genenames = genenames3;

(* Center Arraylets and Calculate Contributions of Genelets to Genes *)

centerarraylets = Transpose[arraylets3];
average = Table[1, {a, 1, genes}];
average = N[average / Sqrt[Dot[average, average]]];
centerarraylets = centerarraylets - N[Outer[Times, Dot[centerarraylets, average], average]];
centerarraylets = Transpose[centerarraylets];
genecontributions = Transpose[Dot[centerarraylets, d3]];

(* Project Genes from 5 D Genelets Subspace Onto 2 D Subspace  *)

coordinates = Table[{(Dot[genelets[[13]], x] * genecontributions[[13, a]] +
        Dot[genelets[[14]], x] * genecontributions[[14, a]] +
        Dot[genelets[[15]], x] * genecontributions[[15, a]] +
        Dot[genelets[[16]], x] * genecontributions[[16, a]] +
        Dot[genelets[[17]], x] * genecontributions[[17, a]]) /
      Sqrt[(a13 * genecontributions[[13, a]])^2 + (a14 * genecontributions[[14, a]])^2 +
        (a15 * genecontributions[[15, a]])^2 + (a16 * genecontributions[[16, a]])^2 +
        (a17 * genecontributions[[17, a]])^2 +
        c1314 * Abs[(a13 * genecontributions[[13, a]]) * (a14 * genecontributions[[14, a]])] +
        c1315 * Abs[(a13 * genecontributions[[13, a]]) * (a15 * genecontributions[[15, a]])] +
        c1316 * Abs[(a13 * genecontributions[[13, a]]) * (a16 * genecontributions[[16, a]])] +
        c1317 * Abs[(a13 * genecontributions[[13, a]]) * (a17 * genecontributions[[17, a]])] +
        c1415 * Abs[(a14 * genecontributions[[14, a]]) * (a15 * genecontributions[[15, a]])] +
        c1416 * Abs[(a14 * genecontributions[[14, a]]) * (a16 * genecontributions[[16, a]])] +
        c1417 * Abs[(a14 * genecontributions[[14, a]]) * (a17 * genecontributions[[17, a]])] +
        c1516 * Abs[(a15 * genecontributions[[15, a]]) * (a16 * genecontributions[[16, a]])] +
        c1517 * Abs[(a15 * genecontributions[[15, a]]) * (a17 * genecontributions[[17, a]])] +
        c1617 * Abs[(a16 * genecontributions[[16, a]]) * (a17 * genecontributions[[17, a]])]],
     (Dot[genelets[[13]], y] * genecontributions[[13, a]] +
        Dot[genelets[[14]], y] * genecontributions[[14, a]] +
        Dot[genelets[[15]], y] * genecontributions[[15, a]] +
        Dot[genelets[[16]], y] * genecontributions[[16, a]] +
        Dot[genelets[[17]], y] * genecontributions[[17, a]]) /
      Sqrt[(a13 * genecontributions[[13, a]])^2 + (a14 * genecontributions[[14, a]])^2 +
        (a15 * genecontributions[[15, a]])^2 + (a16 * genecontributions[[16, a]])^2 +
        (a17 * genecontributions[[17, a]])^2 +
        c1314 * Abs[(a13 * genecontributions[[13, a]]) * (a14 * genecontributions[[14, a]])] +
        c1315 * Abs[(a13 * genecontributions[[13, a]]) * (a15 * genecontributions[[15, a]])] +
        c1316 * Abs[(a13 * genecontributions[[13, a]]) * (a16 * genecontributions[[16, a]])] +
        c1317 * Abs[(a13 * genecontributions[[13, a]]) * (a17 * genecontributions[[17, a]])] +
        c1415 * Abs[(a14 * genecontributions[[14, a]]) * (a15 * genecontributions[[15, a]])] +
        c1416 * Abs[(a14 * genecontributions[[14, a]]) * (a16 * genecontributions[[16, a]])] +
        c1417 * Abs[(a14 * genecontributions[[14, a]]) * (a17 * genecontributions[[17, a]])] +
        c1516 * Abs[(a15 * genecontributions[[15, a]]) * (a16 * genecontributions[[16, a]])] +
        c1517 * Abs[(a15 * genecontributions[[15, a]]) * (a17 * genecontributions[[17, a]])] +
        c1617 * Abs[(a16 * genecontributions[[16, a]]) * (a17 * genecontributions[[17, a]])]]},
    {a, 1, genes}];
```

```
(* Define the Initial Phase *)

phase = ArcTan[TakeColumns[coordinates, {1}], TakeColumns[coordinates, {2}]] / Degree;
Do[If[Negative[phase[[a]][[1]]], phase[[a]] = phase[[a]] + 360], {a, 1, genes}]

(* Sort Genes Accodring to Phases in 2 D Subspace *)

sortmatrix = AppendRows[phase, genenames, matrix];
sortmatrix = Sort[sortmatrix, OrderedQ[{{#1}, {#2}}] &];

(* Sorted Human data *)

phase3 = TakeColumns[sortmatrix, {1, 1}];
matrix3 = TakeColumns[sortmatrix, {5, arrays + 4}];
genenames3 = TakeColumns[sortmatrix, {2, 4}];

(* Calculate HO GSVD of S. pombe, S. cerevisiae and Human Sorted Data *)

cor1 = Dot[Transpose[matrix1], matrix1];
cor2 = Dot[Transpose[matrix2], matrix2];
cor3 = Dot[Transpose[matrix3], matrix3];
cor = Dot[cor1, Inverse[cor2]] + Dot[cor2, Inverse[cor1]] + Dot[cor2, Inverse[cor3]] +
   Dot[cor3, Inverse[cor2]] + Dot[cor1, Inverse[cor3]] + Dot[cor3, Inverse[cor1]];
{values, vectors} = Eigensystem[cor];
genelets = vectors;
Do[genelets[[a]] = genelets[[a]] / Sqrt[Dot[genelets[[a]], genelets[[a]]]], {a, 1, arrays}];

arraylets1 = Dot[matrix1, Inverse[genelets]];
arraylets2 = Dot[matrix2, Inverse[genelets]];
arraylets3 = Dot[matrix3, Inverse[genelets]];
arraylets1 = Transpose[arraylets1];
Do[arraylets1[[a]] = arraylets1[[a]] / Sqrt[Dot[arraylets1[[a]], arraylets1[[a]]]], {a, 1, arrays}];
arraylets1 = Transpose[arraylets1];
arraylets2 = Transpose[arraylets2];
Do[arraylets2[[a]] = arraylets2[[a]] / Sqrt[Dot[arraylets2[[a]], arraylets2[[a]]]], {a, 1, arrays}];
arraylets2 = Transpose[arraylets2];
arraylets3 = Transpose[arraylets3];
Do[arraylets3[[a]] = arraylets3[[a]] / Sqrt[Dot[arraylets3[[a]], arraylets3[[a]]]], {a, 1, arrays}];
arraylets3 = Transpose[arraylets3];
d1 = Chop[Dot[PseudoInverse[arraylets1], matrix1, Inverse[genelets]]];
d2 = Chop[Dot[PseudoInverse[arraylets2], matrix2, Inverse[genelets]]];
d3 = Chop[Dot[PseudoInverse[arraylets3], matrix3, Inverse[genelets]]];
genelets13 = Chop[TrigFit[Drop[genelets[[13]], {1}], 2, {t - 1, arrays - 1}], 0.1];
genelets14 = Chop[TrigFit[Drop[genelets[[14]], {1}], 2, {t - 1, arrays - 1}], 0.1];
genelets15 = Chop[TrigFit[Drop[genelets[[15]], {1}], 2, {t - 1, arrays - 1}], 0.1];
genelets16 = Chop[TrigFit[Drop[genelets[[16]], {1}], 2, {t - 1, arrays - 1}], 0];
genelets17 = Chop[TrigFit[Drop[genelets[[17]], {1}], 2, {t - 1, arrays - 1}], 0];
```

```
(* Display Reconstructed and Sorted S. pombe Data *)

genes = genes1;
genenames = genenames1;
arraynames = arraynames1;

(* Reconstruct Sorted S. pombe Data *)

Do[d1[[a, a]] = 0, {a, 1, 12}];
matrix = Dot[arraylets1, d1, genelets];

(* Center Reconstructed Sorted S. pombe Data *)

average = Table[1, {a, 1, arrays}];
average = N[average / Sqrt[Dot[average, average]]];
matrix = matrix - N[Outer[Times, Dot[matrix, average], average]];
matrix = Transpose[matrix];
average = Table[1, {a, 1, genes}];
average = N[average / Sqrt[Dot[average, average]]];
matrix = matrix - N[Outer[Times, Dot[matrix, average], average]];
matrix = Transpose[matrix];

(* Classify Gene Phases into Cell Cycle Phases *)

Do[ If[phase1[[a]][[1]] > c15 / Degree, {endph1 = a - 1; Break[]}], {a, 1, genes}]
Do[ If[phase1[[a]][[1]] > c14 / Degree, {endph2 = a - 1; Break[]}], {a, 1, genes}]
Do[ If[phase1[[a]][[1]] > c17 / Degree + 180, {endph3 = a - 1; Break[]}], {a, 1, genes}]
Do[ If[phase1[[a]][[1]] > c15 / Degree + 180, {endph4 = a - 1; Break[]}], {a, 1, genes}]
endph5 = genes;

endph1
endph2
endph3
endph4
endph5
```

```
507

917

1590

2019

3167
```

```
(* Create Recosntructed Sorted S. pombe Data 2 D Red & Green Raster Display *)

contrast = 10 * 1.5;
displaying = Table[
   If[contrast * matrix[[i, j]] > 0,
    If[contrast * matrix[[i, j]] < 1, {contrast * matrix[[i, j]], 0}, {1, 0}],
    If[contrast * matrix[[i, j]] > -1, {0, -contrast * matrix[[i, j]]}, {0, 1}]],
   {i, 1, genes}, {j, 1, arrays}];
framex = Table[{a - 0.5, arraynames[[1, a]]}, {a, 1, arrays}];
framey = {
   {genes - endph1 / 2, "G2"},
   {genes - endph1 - (endph2 - endph1) / 2, "M"},
   {genes - endph2 - (endph3 - endph2) / 2, "G1"},
   {genes - endph3 - (endph4 - endph3) / 2, "S"},
   {genes - endph4 - (endph5 - endph4) / 2, "G2"}};
gridy = {
   {genes - endph1 + 0.5, {RGBColor[0, 0, 0]}},
   {genes - endph2 + 0.5, {RGBColor[0, 0, 0]}},
   {genes - endph3 + 0.5, {RGBColor[0, 0, 0]}},
   {genes - endph4 + 0.5, {RGBColor[0, 0, 0]}},
   {genes - endph5 + 0.5, {RGBColor[0, 0, 0]}}};
labelx = "(a)  Arrays";
labely = ColumnForm[{" ", "Genes", " ", " ", " ", " ", " ", " "}, Center];
g = Show[
   Graphics[
    RasterArray[
     Table[
      RGBColor[displaying[[i, j, 1]], displaying[[i, j, 2]], 0],
      {i, genes, 1, -1}, {j, 1, arrays}]]],
   AspectRatio -> 1,
   Frame -> True,
   FrameTicks -> {None, framey, framex, None},
   FrameLabel -> {None, labely, labelx, None},
   GridLines -> {None, gridy},
   DisplayFunction -> Identity];
g = FullGraphics[g];
g[[1, 2]] = g[[1, 2]] /.
   Text[labely, {b_, c_}, {1., 0.}] →
    Text[labely, {b, c}, {0, 0}, {0, 1}];
g[[1, 2]] = g[[1, 2]] /.
   Text[a_, {b_, c_}, {1., 0.}] →
    Text[a, {b - 1.5, c}, {0, 0}, {0, 1}];
g[[1, 2]] = g[[1, 2]] /.
   Text[labelx, {b_, c_}, {0., -1.}] →
    Text[labelx, {b, c + 500}, {0, -1}, {1, 0}];
g[[1, 2]] = g[[1, 2]] /.
   Text[a_, {b_, c_}, {0., -1.}] →
    Text[a, {b, c}, {0, -1}, {0, 1}];
g1 = Show[g,
   AspectRatio -> GoldenRatio * 1.2,
   PlotRange -> All,
   DisplayFunction → Identity];

(* Center Sorted S. pombe Arraylets *)

arraylets = Transpose[arraylets1];
average = Table[1, {a, 1, genes}];
average = N[average / Sqrt[Dot[average, average]]];
arraylets = arraylets - N[Outer[Times, Dot[arraylets, average], average]];
arraylets = Transpose[arraylets];
```

```
(* Create Sorted S. pombe Arraylets 2 D Red & Green Raster Display *)

contrast = 75 * 1.5;
displaying = Table[
   If[contrast * arraylets[[i, j]] > 0,
    If[contrast * arraylets[[i, j]] < 1, {contrast * arraylets[[i, j]], 0}, {1, 0}],
    If[contrast * arraylets[[i, j]] > -1, {0, -contrast * arraylets[[i, j]]}, {0, 1}]],
   {i, 1, genes}, {j, 1, arrays}];
labelx = "(b) Arraylets";
labely = ColumnForm[{" ", " ", " ", " ", " ", " ", " ", " "}, Center];
framex = Table[{a - 0.5, ToString[a]}, {a, 1, arrays}];
framey = {
   {genes - endph1 / 2, " "},
   {genes - endph1 - (endph2 - endph1) / 2, " "},
   {genes - endph2 - (endph3 - endph2) / 2, " "},
   {genes - endph3 - (endph4 - endph3) / 2, " "},
   {genes - endph4 - (endph5 - endph4) / 2, " "}};
gridy = {
   {genes - endph1 + 0.5, {RGBColor[0, 0, 0]}},
   {genes - endph2 + 0.5, {RGBColor[0, 0, 0]}},
   {genes - endph3 + 0.5, {RGBColor[0, 0, 0]}},
   {genes - endph4 + 0.5, {RGBColor[0, 0, 0]}},
   {genes - endph5 + 0.5, {RGBColor[0, 0, 0]}}};
g = Show[
   Graphics[
    RasterArray[
     Table[
      RGBColor[displaying[[i, j, 1]], displaying[[i, j, 2]], 0],
      {i, genes, 1, -1}, {j, 1, arrays}
     ]]],
   AspectRatio -> 1,
   Frame -> True,
   FrameTicks -> {None, framey, framex, None},
   FrameLabel -> {None, labely, labelx, None},
   GridLines -> {None, gridy},
   DisplayFunction -> Identity];
g = FullGraphics[g];
g[[1, 2]] = g[[1, 2]] /.
   Text[labely, {b_, c_}, {1., 0.}] →
    Text[labely, {b, c}, {0, 0}, {0, 1}];
g[[1, 2]] = g[[1, 2]] /.
   Text[a_, {b_, c_}, {1., 0.}] ->
    Text[a, {b - 1.5, c}, {0, 0}, {0, 1}];
g[[1, 2]] = g[[1, 2]] /.
   Text[labelx, {b_, c_}, {0., -1.}] ->
    Text[labelx, {b, c + 500}, {0, -1}, {1, 0}];
g[[1, 2]] = g[[1, 2]] /.
   Text[a_, {b_, c_}, {0., -1.}] ->
    Text[a, {b, c}, {0, -1}, {0, 1}];
g2 = Show[g,
   AspectRatio -> GoldenRatio * 1.2,
   PlotRange -> All,
   DisplayFunction → Identity];

(* Create Selected Sorted S. pombe Arraylets Graph Display *)

arraylets = Transpose[arraylets1];
arraylets13 = Chop[TrigFit[arraylets[[13]], 1, {t, genes - 1}], 0.001];
arraylets14 = Chop[TrigFit[arraylets[[14]], 1, {t, genes - 1}], 0.001];
arraylets15 = Chop[TrigFit[arraylets[[15]], 1, {t, genes - 1}], 0.001];
arraylets16 = Chop[TrigFit[arraylets[[16]], 1, {t, genes - 1}], 0.001];
arraylets17 = Chop[TrigFit[arraylets[[17]], 1, {t, genes - 1}], 0.001];
```

```
p = Table[0, {5}];
color =
  {RGBColor[1, 0, 0], RGBColor[0, 0, 1], RGBColor[0, 0.5, 0], RGBColor[1, 0.5, 0], RGBColor[0.75, 0, 1]};
framex = {{0, "0       "}, {0.06, "0.06    "}, {0.12, "0.12    "}, {0.18, "0.18    "}, {0.24, "0.24    "}};
labelx = "(c) Expression Level";
labely = ColumnForm[{" ", " ", " ", " ", " ", " ", " ", " "}, Center];
flag = 0;
FittedArraylets = {arraylets13, arraylets14, arraylets15, arraylets16, arraylets17};
offset = {0, 0.025, 0.05, 0.075, 0.1};

Do[{
  graph = ParametricPlot[{FittedArraylets[[n]] + offset[[n]] * 2.4, -t},
    {t, 0, genes - 1},
    PlotStyle -> {RGBColor[0, 0, 0], Thickness[0.016]},
    DisplayFunction -> Identity],
  coordinates = Table[
    If[arraylets[[12 + n, a]] + offset[[n]] * 2.4 > 0.125 * 2.4, 0.125 * 2.4,
     If[arraylets[[12 + n, a]] + offset[[n]] * 2.4 < -0.025 * 2.4, -0.025 * 2.4,
      arraylets[[12 + n, a]] + offset[[n]] * 2.4]],
    {a, 1, genes}],
  coordinates = Table[{coordinates[[a]], -a + 1}, {a, 1, genes}],
  line = Line[coordinates],
  g = Show[{
     Graphics[{color[[n]], line}],
     graph},
    If[flag == 0,
     {FrameLabel -> {None, labely, labelx, None},
      FrameTicks -> {None, framey, framex, None}},
     {FrameLabel -> {None, None, None, None},
      FrameTicks -> {None, None, None, None}}],
    Frame -> True,
    GridLines -> {{{offset[[n]] * 2.4, RGBColor[0, 0, 0]}}, None},
    AspectRatio -> GoldenRatio * 1.15,
    PlotRange -> {{-0.025 * 2.4, 0.125 * 2.4}, {85, -genes + 1 - 85}},
    DisplayFunction -> Identity],
  g = FullGraphics[g],
  g[[1, 2]] = g[[1, 2]] /.
    Text[labely, {b_, c_}, {1., 0.}] ->
     Text[labely, {b, c}, {0, 0}, {0, 1}],
  g[[1, 2]] = g[[1, 2]] /.
    Text[a_, {b_, c_}, {1., 0.}] ->
     Text[a, {b - 0.01, c}, {0, 0}, {0, 1}],
  g[[1, 2]] = g[[1, 2]] /.
    Text[labelx, {b_, c_}, {0., -1.}] ->
     Text[labelx, {b, c + 525}, {0, -1}, {1, 0}],
  g[[1, 2]] = g[[1, 2]] /.
    Text[a_, {b_, c_}, {0., -1.}] ->
     Text[a, {b, c}, {0, -1}, {0, 1}],
  flag = flag + 1,
  p[[n]] = Show[g,
    AspectRatio -> GoldenRatio * 1.29,
    PlotRange -> All,
    DisplayFunction -> Identity]},
 {n, 1, 5}]

(* Display Selected Sorted S. pombe Arraylets *)

g3 = Show[{p[[5]], p[[4]], p[[3]], p[[2]], p[[1]]},
   DisplayFunction -> Identity];
```

```
(* Display Reconstructed Sorted S. pombe Data, Arraylets and Selected Arraylets *)

Show[GraphicsArray[{g1, g2, g3}],
    GraphicsSpacing -> -0.2];
```

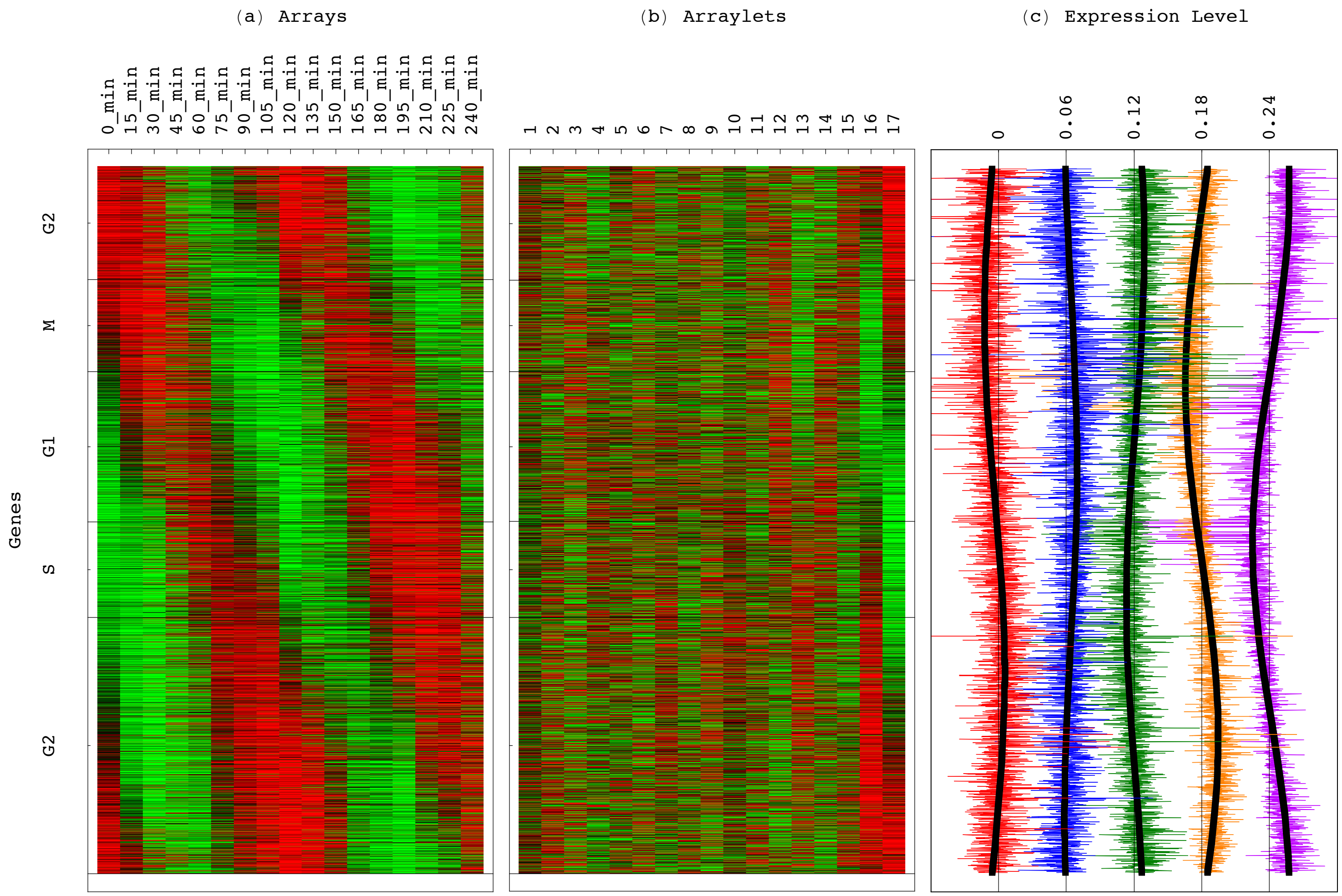

```
(* Display Reconstructed and Sorted S. cerevisiae Data *)

genes = genes2;
genenames = genenames2;
arraynames = arraynames2;

(* Reconstruct Sorted S. cerevisiae Data *)

Do[d2[[a, a]] = 0, {a, 1, 12}];
matrix = Dot[arraylets2, d2, genelets];

(* Center Reconstructed Sorted S. cerevisiae Data *)

average = Table[1, {a, 1, arrays}];
average = N[average / Sqrt[Dot[average, average]]];
matrix = matrix - N[Outer[Times, Dot[matrix, average], average]];
matrix = Transpose[matrix];
average = Table[1, {a, 1, genes}];
average = N[average / Sqrt[Dot[average, average]]];
matrix = matrix - N[Outer[Times, Dot[matrix, average], average]];
matrix = Transpose[matrix];

(* Classify Gene Phases into Cell Cycle Phases *)

Do[ If[phase2[[a]][[1]] > c17 / Degree, {endph1 = a - 1; Break[]}], {a, 1, genes}]
Do[ If[phase2[[a]][[1]] > c14 / Degree, {endph2 = a - 1; Break[]}], {a, 1, genes}]
Do[ If[phase2[[a]][[1]] > c17 / Degree + 180, {endph3 = a - 1; Break[]}], {a, 1, genes}]
Do[ If[phase2[[a]][[1]] > c15 / Degree + 180, {endph4 = a - 1; Break[]}], {a, 1, genes}]
Do[ If[phase2[[a]][[1]] > c16 / Degree + 360, {endph5 = a - 1; Break[]}], {a, 1, genes}]
endph6 = genes;

endph1
endph2
endph3
endph4
endph5
endph6
```

```
151

1393

2354

2883

3696

4772
```

```
(* Create Reconstructed Sorted S. cerevisiae Data 2 D Red & Green Raster Display *)

contrast = 10 * 1.5;
displaying = Table[
   If[contrast * matrix[[i, j]] > 0,
    If[contrast * matrix[[i, j]] < 1, {contrast * matrix[[i, j]], 0}, {1, 0}],
    If[contrast * matrix[[i, j]] > -1, {0, -contrast * matrix[[i, j]]}, {0, 1}]],
   {i, 1, genes}, {j, 1, arrays}];
framex = Table[{a - 0.5, arraynames[[1, a]]}, {a, 1, arrays}];
framey = {
   {genes - endph1 / 2, "G2/M"},
   {genes - endph1 - (endph2 - endph1) / 2, "M/G1"},
   {genes - endph2 - (endph3 - endph2) / 2, "G1"},
   {genes - endph3 - (endph4 - endph3) / 2, "S"},
   {genes - endph4 - (endph5 - endph4) / 2, "S/G2"},
   {genes - endph5 - (endph6 - endph5) / 2, "G2/M"}};
gridy = {
   {genes - endph1 + 0.5, {RGBColor[0, 0, 0]}},
   {genes - endph2 + 0.5, {RGBColor[0, 0, 0]}},
   {genes - endph3 + 0.5, {RGBColor[0, 0, 0]}},
   {genes - endph4 + 0.5, {RGBColor[0, 0, 0]}},
   {genes - endph5 + 0.5, {RGBColor[0, 0, 0]}},
   {genes - endph6 + 0.5, {RGBColor[0, 0, 0]}}};
labelx = "(a)  Arrays";
labely = ColumnForm[{" ", "Genes", " ", " ", " ", " ", " ", " "}, Center];
g = Show[
   Graphics[
    RasterArray[
     Table[
      RGBColor[displaying[[i, j, 1]], displaying[[i, j, 2]], 0],
      {i, genes, 1, -1}, {j, 1, arrays}]]],
   AspectRatio -> 1,
   Frame -> True,
   FrameTicks -> {None, framey, framex, None},
   FrameLabel -> {None, labely, labelx, None},
   GridLines -> {None, gridy},
   DisplayFunction -> Identity];
g = FullGraphics[g];
g[[1, 2]] = g[[1, 2]] /.
   Text[labely, {b_, c_}, {1., 0.}] ->
    Text[labely, {b, c}, {0, 0}, {0, 1}];
g[[1, 2]] = g[[1, 2]] /.
   Text[a_, {b_, c_}, {1., 0.}] ->
    Text[a, {b - 1.5, c}, {0, 0}, {0, 1}];
g[[1, 2]] = g[[1, 2]] /.
   Text[labelx, {b_, c_}, {0., -1.}] ->
    Text[labelx, {b, c + 600}, {0, -1}, {1, 0}];
g[[1, 2]] = g[[1, 2]] /.
   Text[a_, {b_, c_}, {0., -1.}] ->
    Text[a, {b, c}, {0, -1}, {0, 1}];
g4 = Show[g,
   AspectRatio -> GoldenRatio * 1.4,
   PlotRange -> All,
   DisplayFunction → Identity];

(* Center Sorted S. cerevisiae Arraylets *)

arraylets = Transpose[arraylets2];
average = Table[1, {a, 1, genes}];
average = N[average / Sqrt[Dot[average, average]]];
arraylets = arraylets - N[Outer[Times, Dot[arraylets, average], average]];
arraylets = Transpose[arraylets];
```

```
(* Create Sorted S. cerevisiae Arraylets 2 D Red & Green Raster Display *)

contrast = 75 * 1.5;
displaying = Table[
   If[contrast * arraylets[[i, j]] > 0,
    If[contrast * arraylets[[i, j]] < 1, {contrast * arraylets[[i, j]], 0}, {1, 0}],
    If[contrast * arraylets[[i, j]] > -1, {0, -contrast * arraylets[[i, j]]}, {0, 1}]],
   {i, 1, genes}, {j, 1, arrays}];
labelx = "(b) Arraylets";
labely = ColumnForm[{" ", " ", " ", " ", " ", " ", " ", " "}, Center];
framey = {
   {genes - endph1 / 2, " "},
   {genes - endph1 - (endph2 - endph1) / 2, " "},
   {genes - endph2 - (endph3 - endph2) / 2, " "},
   {genes - endph3 - (endph4 - endph3) / 2, " "},
   {genes - endph4 - (endph5 - endph4) / 2, " "},
   {genes - endph5 - (endph6 - endph5) / 2, " "}};
gridy = {
   {genes - endph1 + 0.5, {RGBColor[0, 0, 0]}},
   {genes - endph2 + 0.5, {RGBColor[0, 0, 0]}},
   {genes - endph3 + 0.5, {RGBColor[0, 0, 0]}},
   {genes - endph4 + 0.5, {RGBColor[0, 0, 0]}},
   {genes - endph5 + 0.5, {RGBColor[0, 0, 0]}},
   {genes - endph6 + 0.5, {RGBColor[0, 0, 0]}}};
framex = Table[{a - 0.5, ToString[a]}, {a, 1, arrays}];
g = Show[
   Graphics[
    RasterArray[
     Table[
      RGBColor[displaying[[i, j, 1]], displaying[[i, j, 2]], 0],
      {i, genes, 1, -1}, {j, 1, arrays}
     ]]],
   AspectRatio -> 1,
   Frame -> True,
   FrameTicks -> {None, framey, framex, None},
   FrameLabel -> {None, labely, labelx, None},
   GridLines -> {None, gridy},
   DisplayFunction -> Identity];
g = FullGraphics[g];
g[[1, 2]] = g[[1, 2]] /.
   Text[labely, {b_, c_}, {1., 0.}] ->
    Text[labely, {b, c}, {0, 0}, {0, 1}];
g[[1, 2]] = g[[1, 2]] /.
   Text[a_, {b_, c_}, {1., 0.}] ->
    Text[a, {b - 1.5, c}, {0, 0}, {0, 1}];
g[[1, 2]] = g[[1, 2]] /.
   Text[labelx, {b_, c_}, {0., -1.}] ->
    Text[labelx, {b, c + 600}, {0, -1}, {1, 0}];
g[[1, 2]] = g[[1, 2]] /.
   Text[a_, {b_, c_}, {0., -1.}] ->
    Text[a, {b, c}, {0, -1}, {0, 1}];
g5 = Show[g,
   AspectRatio -> GoldenRatio * 1.4,
   PlotRange -> All,
   DisplayFunction -> Identity];

(* Create Selected Sorted S. cerevisiae Arraylets Graph Display *)

arraylets = Transpose[arraylets2];
arraylets13 = Chop[TrigFit[arraylets[[13]], 1, {t, genes - 1}], 0.001];
arraylets14 = Chop[TrigFit[arraylets[[14]], 1, {t, genes - 1}], 0.001];
arraylets15 = Chop[TrigFit[arraylets[[15]], 1, {t, genes - 1}], 0.001];
arraylets16 = Chop[TrigFit[arraylets[[16]], 1, {t, genes - 1}], 0.001];
arraylets17 = Chop[TrigFit[arraylets[[17]], 1, {t, genes - 1}], 0.001];
```

```
p = Table[0, {5}];
color =
  {RGBColor[1, 0, 0], RGBColor[0, 0, 1], RGBColor[0, 0.5, 0], RGBColor[1, 0.5, 0], RGBColor[0.75, 0, 1]};
framex = {{0, "0       "}, {0.05, "0.05    "}, {0.10, "0.10    "}, {0.15, "0.15    "}, {0.20, "0.20    "}};
labelx = "(c) Expression Level";
labely = ColumnForm[{" ", " ", " ", " ", " ", " ", " ", " "}, Center];
flag = 0;
FittedArraylets = {arraylets13, arraylets14, arraylets15, arraylets16, arraylets17};
offset = {0, 0.025, 0.05, 0.075, 0.1};

Do[{
  graph = ParametricPlot[{FittedArraylets[[n]] + offset[[n]] * 2.0, -t},
    {t, 0, genes - 1},
    PlotStyle -> {RGBColor[0, 0, 0], Thickness[0.016]},
    DisplayFunction -> Identity],
  coordinates = Table[
    If[arraylets[[12 + n, a]] + offset[[n]] * 2.0 > 0.125 * 2.0, 0.125 * 2.0,
     If[arraylets[[12 + n, a]] + offset[[n]] * 2.0 < -0.025 * 2.0,
      -0.025 * 2.0, arraylets[[12 + n, a]] + offset[[n]] * 2.0]],
    {a, 1, genes}],
  coordinates = Table[{coordinates[[a]], -a + 1}, {a, 1, genes}],
  line = Line[coordinates],
  g = Show[{
     Graphics[{color[[n]], line}],
     graph},
    If[flag == 0,
     {FrameLabel -> {None, labely, labelx, None},
      FrameTicks -> {None, framey, framex, None}},
     {FrameLabel -> {None, None, None, None},
      FrameTicks -> {None, None, None, None}}],
    Frame -> True,
    GridLines -> {{{offset[[n]] * 2.0, RGBColor[0, 0, 0]}}, None},
    AspectRatio -> GoldenRatio * 1.15,
    PlotRange -> {{-0.025 * 2.0, 0.125 * 2.0}, {135, -genes + 1 - 130}},
    DisplayFunction -> Identity],
  g = FullGraphics[g],
  g[[1, 2]] = g[[1, 2]] /.
    Text[labely, {b_, c_}, {1., 0.}] ->
     Text[labely, {b, c}, {0, 0}, {0, 1}],
  g[[1, 2]] = g[[1, 2]] /.
    Text[a_, {b_, c_}, {1., 0.}] ->
     Text[a, {b - 0.01, c}, {0, 0}, {0, 1}],
  g[[1, 2]] = g[[1, 2]] /.
    Text[labelx, {b_, c_}, {0., -1.}] ->
     Text[labelx, {b, c + 635}, {0, -1}, {1, 0}],
  g[[1, 2]] = g[[1, 2]] /.
    Text[a_, {b_, c_}, {0., -1.}] ->
     Text[a, {b, c}, {0, -1}, {0, 1}],
  flag = flag + 1,
  p[[n]] = Show[g,
    AspectRatio -> GoldenRatio * 1.5025,
    PlotRange -> All,
    DisplayFunction -> Identity]},
 {n, 1, 5}]

(* Display Selected Sorted S. cerevisiae Arraylets *)

g6 = Show[{p[[5]], p[[4]], p[[3]], p[[2]], p[[1]]},
   DisplayFunction -> Identity];
```

```
(* Display Reconstructed Sorted S. cerevisiae Data, Arraylets and Selected Arraylets *)

Show[GraphicsArray[{g4, g5, g6}],
    GraphicsSpacing -> -0.2];
```

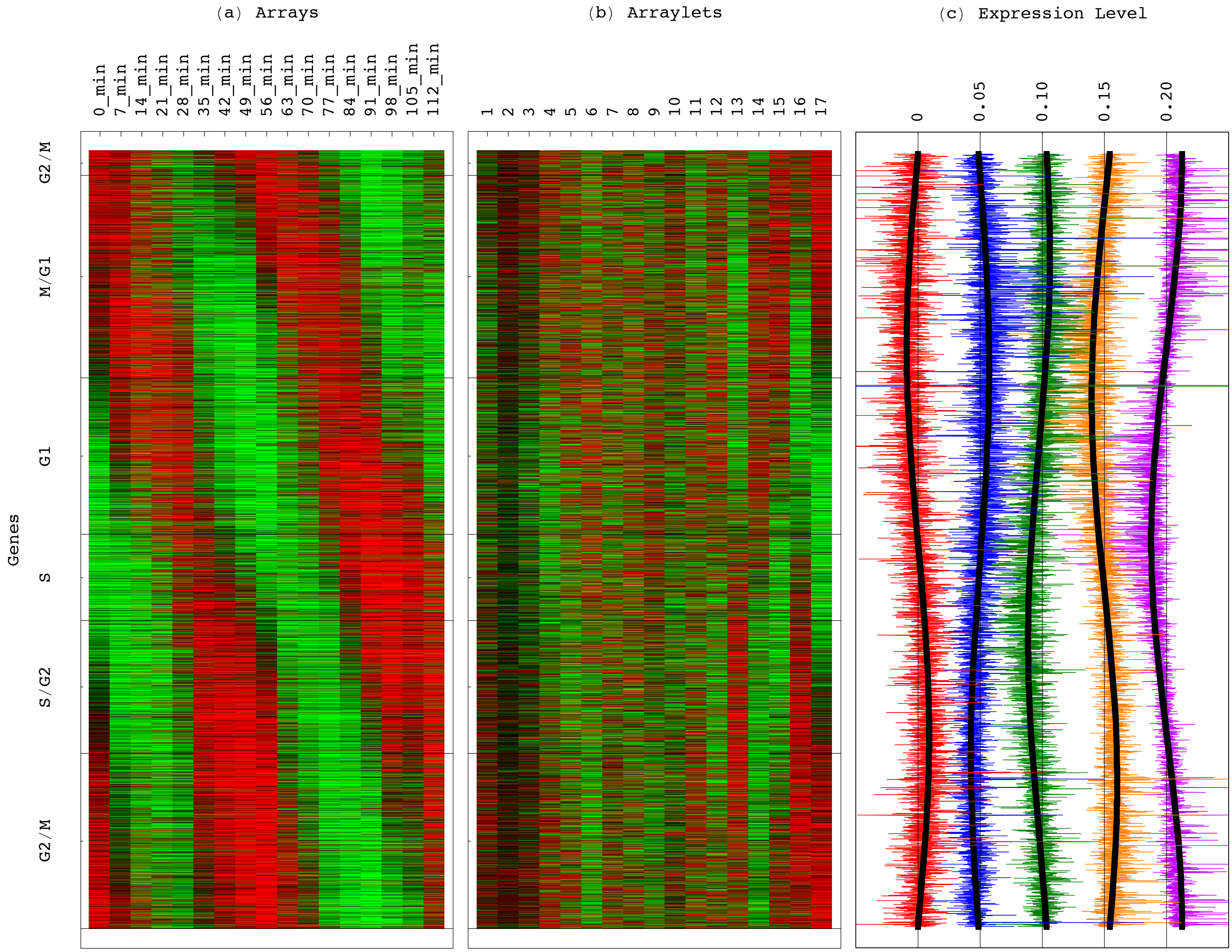

```
(* Display Reconstructed and Sorted Human Data *)

genes = genes3;
genenames = genenames3;
arraynames = arraynames3;

(* Reconstruct Sorted Human Data *)

Do[d3[[a, a]] = 0, {a, 1, 12}];
matrix = Dot[arraylets3, d3, genelets];

(* Center Reconstructed Sorted Human Data *)

average = Table[1, {a, 1, arrays}];
average = N[average / Sqrt[Dot[average, average]]];
matrix = matrix - N[Outer[Times, Dot[matrix, average], average]];
matrix = Transpose[matrix];
average = Table[1, {a, 1, genes}];
average = N[average / Sqrt[Dot[average, average]]];
matrix = matrix - N[Outer[Times, Dot[matrix, average], average]];
matrix = Transpose[matrix];

(* Classify Gene Phases into Cell Cycle Phases *)

Do[ If[phase3[[a]][[1]] > c17 / Degree, {endph1 = a - 1; Break[]}], {a, 1, genes}]
Do[ If[phase3[[a]][[1]] > c15 / Degree, {endph2 = a - 1; Break[]}], {a, 1, genes}]
Do[ If[phase3[[a]][[1]] > c14 / Degree, {endph3 = a - 1; Break[]}], {a, 1, genes}]
Do[ If[phase3[[a]][[1]] > c17 / Degree + 180, {endph4 = a - 1; Break[]}], {a, 1, genes}]
Do[ If[phase3[[a]][[1]] > c16 / Degree + 360, {endph5 = a - 1; Break[]}], {a, 1, genes}]
endph6 = genes;

endph1
endph2
endph3
endph4
endph5
endph6

473

2070

3977

6661

10763

13068
```

```
(* Create Reconstructed Sorted Human Data 2 D Red & Green Raster Display *)

contrast = 10 * 1.5;
displaying = Table[
   If[contrast * matrix[[i, j]] > 0,
    If[contrast * matrix[[i, j]] < 1, {contrast * matrix[[i, j]], 0}, {1, 0}],
    If[contrast * matrix[[i, j]] > -1, {0, -contrast * matrix[[i, j]]}, {0, 1}]],
   {i, 1, genes}, {j, 1, arrays}];
framex = Table[{a - 0.5, arraynames[[1, a]]}, {a, 1, arrays}];
framey = {
   {genes - endph1 / 2, "S"},
   {genes - endph1 - (endph2 - endph1) / 2, "G2"},
   {genes - endph2 - (endph3 - endph2) / 2, "G2/M"},
   {genes - endph3 - (endph4 - endph3) / 2, "M/G1"},
   {genes - endph4 - (endph5 - endph4) / 2, "G1/S"},
   {genes - endph5 - (endph6 - endph5) / 2, "S"}};
gridy = {
   {genes - endph1 + 0.5, {RGBColor[0, 0, 0]}},
   {genes - endph2 + 0.5, {RGBColor[0, 0, 0]}},
   {genes - endph3 + 0.5, {RGBColor[0, 0, 0]}},
   {genes - endph4 + 0.5, {RGBColor[0, 0, 0]}},
   {genes - endph5 + 0.5, {RGBColor[0, 0, 0]}},
   {genes - endph6 + 0.5, {RGBColor[0, 0, 0]}}};
labelx = "(a)  Arrays";
labely = ColumnForm[{" ", "Genes", " ", " ", " ", " ", " ", " "}, Center];
g = Show[
   Graphics[
    RasterArray[
     Table[
      RGBColor[displaying[[i, j, 1]], displaying[[i, j, 2]], 0],
      {i, genes, 1, -1}, {j, 1, arrays}]]],
   AspectRatio -> 1,
   Frame -> True,
   FrameTicks -> {None, framey, framex, None},
   FrameLabel -> {None, labely, labelx, None},
   GridLines -> {None, gridy},
   DisplayFunction -> Identity];
g = FullGraphics[g];
g[[1, 2]] = g[[1, 2]] /.
   Text[labely, {b_, c_}, {1., 0.}] ->
    Text[labely, {b, c}, {0, 0}, {0, 1}];
g[[1, 2]] = g[[1, 2]] /.
   Text[a_, {b_, c_}, {1., 0.}] ->
    Text[a, {b - 1.5, c}, {0, 0}, {0, 1}];
g[[1, 2]] = g[[1, 2]] /.
   Text[labelx, {b_, c_}, {0., -1.}] ->
    Text[labelx, {b, c + 1000}, {0, -1}, {1, 0}];
g[[1, 2]] = g[[1, 2]] /.
   Text[a_, {b_, c_}, {0., -1.}] ->
    Text[a, {b, c}, {0, -1}, {0, 1}];
g7 = Show[g,
   AspectRatio -> GoldenRatio * 2,
   PlotRange -> All,
   DisplayFunction -> Identity];

(* Center Sorted Human Arraylets *)

arraylets = Transpose[arraylets3];
average = Table[1, {a, 1, genes}];
average = N[average / Sqrt[Dot[average, average]]];
arraylets = arraylets - N[Outer[Times, Dot[arraylets, average], average]];
arraylets = Transpose[arraylets];
```

```
(* Create Sorted Human Arraylets 2 D Red & Green Raster Display *)

contrast = 75 * 1.5;
displaying = Table[
   If[contrast * arraylets[[i, j]] > 0,
    If[contrast * arraylets[[i, j]] < 1, {contrast * arraylets[[i, j]], 0}, {1, 0}],
    If[contrast * arraylets[[i, j]] > -1, {0, -contrast * arraylets[[i, j]]}, {0, 1}]],
   {i, 1, genes}, {j, 1, arrays}];
labelx = "(b) Arraylets";
labely = ColumnForm[{" ", " ", " ", " ", " ", " ", " ", " "}, Center];
framey = {
   {genes - endph1 / 2, " "},
   {genes - endph1 - (endph2 - endph1) / 2, " "},
   {genes - endph2 - (endph3 - endph2) / 2, " "},
   {genes - endph3 - (endph4 - endph3) / 2, " "},
   {genes - endph4 - (endph5 - endph4) / 2, " "},
   {genes - endph5 - (endph6 - endph5) / 2, " "}};
gridy = {
   {genes - endph1 + 0.5, {RGBColor[0, 0, 0]}},
   {genes - endph2 + 0.5, {RGBColor[0, 0, 0]}},
   {genes - endph3 + 0.5, {RGBColor[0, 0, 0]}},
   {genes - endph4 + 0.5, {RGBColor[0, 0, 0]}},
   {genes - endph5 + 0.5, {RGBColor[0, 0, 0]}},
   {genes - endph6 + 0.5, {RGBColor[0, 0, 0]}}};
framex = Table[{a - 0.5, ToString[a]}, {a, 1, arrays}];
g = Show[
   Graphics[
    RasterArray[
     Table[
      RGBColor[displaying[[i, j, 1]], displaying[[i, j, 2]], 0],
      {i, genes, 1, -1}, {j, 1, arrays}
     ]]],
   AspectRatio -> 1,
   Frame -> True,
   FrameTicks -> {None, framey, framex, None},
   FrameLabel -> {None, labely, labelx, None},
   GridLines -> {None, gridy},
   DisplayFunction -> Identity];
g = FullGraphics[g];
g[[1, 2]] = g[[1, 2]] /.
   Text[labely, {b_, c_}, {1., 0.}] ->
    Text[labely, {b, c}, {0, 0}, {0, 1}];
g[[1, 2]] = g[[1, 2]] /.
   Text[a_, {b_, c_}, {1., 0.}] ->
    Text[a, {b - 1.5, c}, {0, 0}, {0, 1}];
g[[1, 2]] = g[[1, 2]] /.
   Text[labelx, {b_, c_}, {0., -1.}] ->
    Text[labelx, {b, c + 1000}, {0, -1}, {1, 0}];
g[[1, 2]] = g[[1, 2]] /.
   Text[a_, {b_, c_}, {0., -1.}] ->
    Text[a, {b, c}, {0, -1}, {0, 1}];
g8 = Show[g,
   AspectRatio -> GoldenRatio * 2,
   PlotRange -> All,
   DisplayFunction -> Identity];

(* Create Selected Sorted Human Arraylets Graph Display *)

arraylets = Transpose[arraylets3];
arraylets13 = Chop[TrigFit[arraylets[[13]], 1, {t, genes - 1}], 0.001];
arraylets14 = Chop[TrigFit[arraylets[[14]], 1, {t, genes - 1}], 0.001];
arraylets15 = Chop[TrigFit[arraylets[[15]], 1, {t, genes - 1}], 0.001];
arraylets16 = Chop[TrigFit[arraylets[[16]], 1, {t, genes - 1}], 0.001];
arraylets17 = Chop[TrigFit[arraylets[[17]], 1, {t, genes - 1}], 0.001];
```

```
p = Table[0, {5}];
color =
  {RGBColor[1, 0, 0], RGBColor[0, 0, 1], RGBColor[0, 0.5, 0], RGBColor[1, 0.5, 0], RGBColor[0.75, 0, 1]};
framex = {{0, "0       "}, {0.035, "0.035   "}, {0.07, "0.070   "}, {0.105, "0.105   "}, {0.14, "0.140   "}};
labelx = "(c) Expression Level";
labely = ColumnForm[{" ", " ", " ", " ", " ", " ", " ", " "}, Center];
flag = 0;
FittedArraylets = {arraylets13, arraylets14, arraylets15, arraylets16, arraylets17};
offset = {0, 0.025, 0.05, 0.075, 0.1};

Do[{
  graph = ParametricPlot[{FittedArraylets[[n]] + offset[[n]] * 1.4, -t},
    {t, 0, genes - 1},
    PlotStyle -> {RGBColor[0, 0, 0], Thickness[0.016]},
    DisplayFunction -> Identity],
  coordinates = Table[
    If[arraylets[[12 + n, a]] + offset[[n]] * 1.4 > 0.125 * 1.4, 0.125 * 1.4,
     If[arraylets[[12 + n, a]] + offset[[n]] * 1.4 < -0.025 * 1.4, -0.025 * 1.4,
      arraylets[[12 + n, a]] + offset[[n]] * 1.4]],
    {a, 1, genes}],
  coordinates = Table[{coordinates[[a]], -a + 1}, {a, 1, genes}],
  line = Line[coordinates],
  g = Show[{
     Graphics[{color[[n]], line}],
     graph},
    If[flag == 0,
     {FrameLabel -> {None, labely, labelx, None},
      FrameTicks -> {None, framey, framex, None}},
     {FrameLabel -> {None, None, None, None},
      FrameTicks -> {None, None, None, None}}],
    Frame -> True,
    GridLines -> {{{offset[[n]] * 1.4, RGBColor[0, 0, 0]}}, None},
    AspectRatio -> GoldenRatio * 1.15,
    PlotRange -> {{-0.025 * 1.4, 0.125 * 1.4}, {365, -genes + 1 - 380}},
    DisplayFunction -> Identity],
  g = FullGraphics[g],
  g[[1, 2]] = g[[1, 2]] /.
    Text[labely, {b_, c_}, {1., 0.}] ->
     Text[labely, {b, c}, {0, 0}, {0, 1}],
  g[[1, 2]] = g[[1, 2]] /.
    Text[a_, {b_, c_}, {1., 0.}] ->
     Text[a, {b - 0.01, c}, {0, 0}, {0, 1}],
  g[[1, 2]] = g[[1, 2]] /.
    Text[labelx, {b_, c_}, {0., -1.}] ->
     Text[labelx, {b, c + 1090}, {0, -1}, {1, 0}],
  g[[1, 2]] = g[[1, 2]] /.
    Text[a_, {b_, c_}, {0., -1.}] ->
     Text[a, {b, c}, {0, -1}, {0, 1}],
  flag = flag + 1,
  p[[n]] = Show[g,
    AspectRatio -> GoldenRatio * 2.15,
    PlotRange -> All,
    DisplayFunction -> Identity]},
 {n, 1, 5}]

(* Display Selected Sorted Human Arraylets *)

g9 = Show[{p[[5]], p[[4]], p[[3]], p[[2]], p[[1]]}, DisplayFunction -> Identity];

(* Display Reconstructed Sorted Human Data, Arraylets and Selected Arraylets *)

Show[GraphicsArray[{g7, g8, g9}], GraphicsSpacing -> -0.2];
```

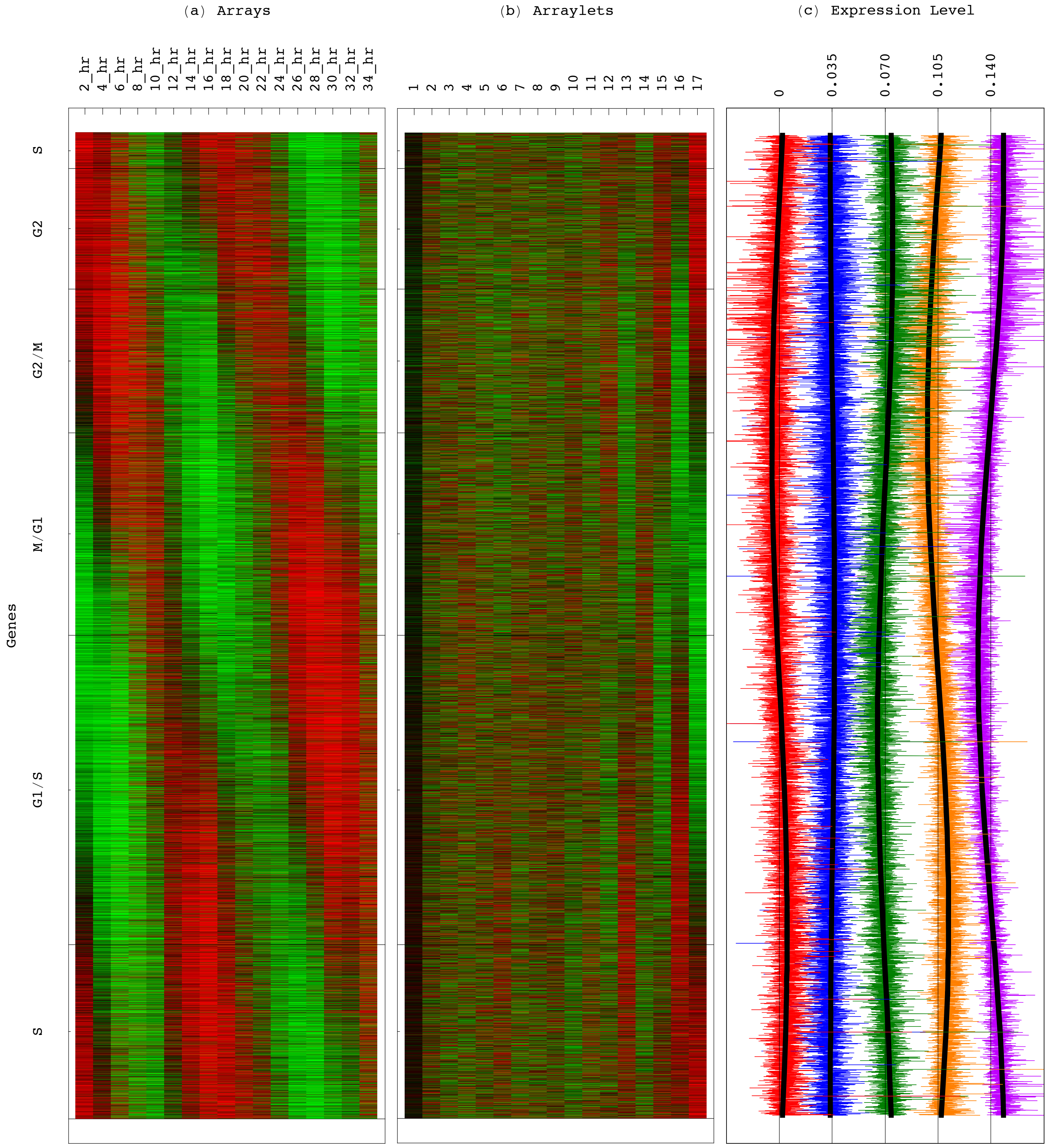
(a) Arrays
(b) Arraylets
(c) Expression Level
2_hr
4_hr
6_hr
8_hr
10_hr
12_hr
14_hr
16_hr
18_hr
20_hr
22_hr
24_hr
26_hr
28_hr
30_hr
32_hr
34_hr
1
2
3
4
5
6
7
8
9
10
11
12
13
14
15
16
17
0
0.035
0.070
0.105
0.140
Genes
S
G2
G2/M
M/G1
G1/S
S

(* Display HO GSVD of Sorted S. pombe, S. cerevisiae and Human Data *)

(* Recalculate HO GSVD of Sorted Original S. pombe, S. cerevisiae and Human Data *)

```
cor1 = Dot[Transpose[matrix1], matrix1];
cor2 = Dot[Transpose[matrix2], matrix2];
cor3 = Dot[Transpose[matrix3], matrix3];
cor = Dot[cor1, Inverse[cor2]] + Dot[cor2, Inverse[cor1]] + Dot[cor2, Inverse[cor3]] +
   Dot[cor3, Inverse[cor2]] + Dot[cor1, Inverse[cor3]] + Dot[cor3, Inverse[cor1]];
{values, vectors} = Eigensystem[cor];
genelets = vectors;
Do[genelets[[a]] = genelets[[a]] / Sqrt[Dot[genelets[[a]], genelets[[a]]]], {a, 1, arrays}]
arraylets1 = Dot[matrix1, Inverse[genelets]];
arraylets2 = Dot[matrix2, Inverse[genelets]];
arraylets3 = Dot[matrix3, Inverse[genelets]];
Projection1 = arraylets1;
Projection2 = arraylets2;
Projection3 = arraylets3;
arraylets1 = Transpose[arraylets1];
Do[arraylets1[[a]] = arraylets1[[a]] / Sqrt[Dot[arraylets1[[a]], arraylets1[[a]]]], {a, 1, arrays}];
arraylets1 = Transpose[arraylets1];
arraylets2 = Transpose[arraylets2];
Do[arraylets2[[a]] = arraylets2[[a]] / Sqrt[Dot[arraylets2[[a]], arraylets2[[a]]]], {a, 1, arrays}];
arraylets2 = Transpose[arraylets2];
arraylets3 = Transpose[arraylets3];
Do[arraylets3[[a]] = arraylets3[[a]] / Sqrt[Dot[arraylets3[[a]], arraylets3[[a]]]], {a, 1, arrays}];
arraylets3 = Transpose[arraylets3];
d1 = Dot[PseudoInverse[arraylets1], matrix1, Inverse[genelets]];
d2 = Dot[PseudoInverse[arraylets2], matrix2, Inverse[genelets]];
d3 = Dot[PseudoInverse[arraylets3], matrix3, Inverse[genelets]];
```

(* Display Sorted S. pombe Data *)

```
genes = genes1;
genenames = genenames1;
arraynames = arraynames1;
matrix = matrix1;
```

(* Center Sorted S. pombe Data *)

```
average = Table[1, {a, 1, arrays}];
average = N[average / Sqrt[Dot[average, average]]];
matrix = matrix - N[Outer[Times, Dot[matrix, average], average]];
matrix = Transpose[matrix];
average = Table[1, {a, 1, genes}];
average = N[average / Sqrt[Dot[average, average]]];
matrix = matrix - N[Outer[Times, Dot[matrix, average], average]];
matrix = Transpose[matrix];
```

(* Create Sorted S. pombe Data 2 D Red & Green Raster Display *)

```
contrast = 10 * 1.5;
displaying = Table[
   If[contrast * matrix[[i, j]] > 0,
    If[contrast * matrix[[i, j]] < 1, {contrast * matrix[[i, j]], 0}, {1, 0}],
    If[contrast * matrix[[i, j]] > -1, {0, -contrast * matrix[[i, j]]}, {0, 1}]],
   {i, 1, genes}, {j, 1, arrays}];
T1 = Table[
   RGBColor[displaying[[i, j, 1]], displaying[[i, j, 2]], 0],
   {i, genes, 1, -1}, {j, 1, arrays}];
```

```
(* Center Sorted S. pombe Arraylets *)

arraylets = Transpose[arraylets1];
average = Table[1, {a, 1, genes}];
average = N[average / Sqrt[Dot[average, average]]];
arraylets = arraylets - N[Outer[Times, Dot[arraylets, average], average]];
arraylets = Transpose[arraylets];

(* Create Sorted S. pombe Arraylets 2 D Red & Green Raster Display *)

contrast = 75 * 1.5;
displaying = Table[
   If[contrast * arraylets[[i, j]] > 0,
    If[contrast * arraylets[[i, j]] < 1, {contrast * arraylets[[i, j]], 0}, {1, 0}],
    If[contrast * arraylets[[i, j]] > -1, {0, -contrast * arraylets[[i, j]]}, {0, 1}]],
   {i, 1, genes}, {j, 1, arrays}];
U1 = Table[
   RGBColor[displaying[[i, j, 1]], displaying[[i, j, 2]], 0],
   {i, genes, 1, -1}, {j, 1, arrays}];

(* Create S. pombe Expression Fractions Red & Green Raster Display *)

contrast = 0.03;
displaying = Table[
   If[contrast * d1[[i, j]] > 0,
    If[contrast * d1[[i, j]] < 1, {contrast * d1[[i, j]], 0}, {1, 0}],
    If[contrast * d1[[i, j]] > -1, {0, -contrast * d1[[i, j]]}, {0, 1}]],
   {i, 1, arrays}, {j, 1, arrays}];
ue1 = Table[
   RGBColor[displaying[[i, j, 1]], displaying[[i, j, 2]], 0],
   {i, arrays, 1, -1}, {j, 1, arrays}];

(* Center Genelets *)

average = Table[1, {a, 1, arrays}];
average = N[average / Sqrt[Dot[average, average]]];
centergenelets = genelets - N[Outer[Times, Dot[genelets, average], average]];

(* Create Genelets 2 D Red & Green Raster Display *)

contrast = 3;
displaying = Table[
   If[contrast * centergenelets[[i, j]] > 0,
    If[contrast * centergenelets[[i, j]] < 1, {contrast * centergenelets[[i, j]], 0}, {1, 0}],
    If[contrast * centergenelets[[i, j]] > -1, {0, -contrast * centergenelets[[i, j]]}, {0, 1}]],
   {i, 1, arrays}, {j, 1, arrays}];
G = Table[
   RGBColor[displaying[[i, j, 1]], displaying[[i, j, 2]], 0],
   {i, arrays, 1, -1}, {j, 1, arrays}];
```

```
(* Display Sorted S. cerevisiae Data *)

genes = genes2;
genenames = genenames2;
arraynames = arraynames2;
matrix = matrix2;

(* Center Sorted S. cerevisiae Data *)

average = Table[1, {a, 1, arrays}];
average = N[average / Sqrt[Dot[average, average]]];
matrix = matrix - N[Outer[Times, Dot[matrix, average], average]];
matrix = Transpose[matrix];
average = Table[1, {a, 1, genes}];
average = N[average / Sqrt[Dot[average, average]]];
matrix = matrix - N[Outer[Times, Dot[matrix, average], average]];
matrix = Transpose[matrix];

(* Create Sorted S. cerevisiae Data 2 D Red & Green Raster Display *)

contrast = 15 * 1.5;
displaying = Table[
   If[contrast * matrix[[i, j]] > 0,
    If[contrast * matrix[[i, j]] < 1, {contrast * matrix[[i, j]], 0}, {1, 0}],
    If[contrast * matrix[[i, j]] > -1, {0, -contrast * matrix[[i, j]]}, {0, 1}]],
   {i, 1, genes}, {j, 1, arrays}];
T2 = Table[
   RGBColor[displaying[[i, j, 1]], displaying[[i, j, 2]], 0],
   {i, genes, 1, -1}, {j, 1, arrays}];

(* Center Sorted S. cerevisiae Arraylets *)

arraylets = Transpose[arraylets2];
average = Table[1, {a, 1, genes}];
average = N[average / Sqrt[Dot[average, average]]];
arraylets = arraylets - N[Outer[Times, Dot[arraylets, average], average]];
arraylets = Transpose[arraylets];

(* Create Sorted S. cerevisiae Arraylets 2 D Red & Green Raster Display *)

contrast = 125 * 1.5;
displaying = Table[
   If[contrast * arraylets[[i, j]] > 0,
    If[contrast * arraylets[[i, j]] < 1, {contrast * arraylets[[i, j]], 0}, {1, 0}],
    If[contrast * arraylets[[i, j]] > -1, {0, -contrast * arraylets[[i, j]]}, {0, 1}]],
   {i, 1, genes}, {j, 1, arrays}];
U2 = Table[
   RGBColor[displaying[[i, j, 1]], displaying[[i, j, 2]], 0],
   {i, genes, 1, -1}, {j, 1, arrays}];

(* Create S. cerevisiae Expression Fractions Red & Green Raster Display *)

contrast = 0.03;
displaying = Table[
   If[contrast * d2[[i, j]] > 0,
    If[contrast * d2[[i, j]] < 1, {contrast * d2[[i, j]], 0}, {1, 0}],
    If[contrast * d2[[i, j]] > -1, {0, -contrast * d2[[i, j]]}, {0, 1}]],
   {i, 1, arrays}, {j, 1, arrays}];
ue2 = Table[
   RGBColor[displaying[[i, j, 1]], displaying[[i, j, 2]], 0],
   {i, arrays, 1, -1}, {j, 1, arrays}];
```

```
(* Display Sorted Human Data *)

genes = genes3;
genenames = genenames3;
arraynames = arraynames3;
matrix = matrix3;

(* Center Sorted Human Data *)

average = Table[1, {a, 1, arrays}];
average = N[average / Sqrt[Dot[average, average]]];
matrix = matrix - N[Outer[Times, Dot[matrix, average], average]];
matrix = Transpose[matrix];
average = Table[1, {a, 1, genes}];
average = N[average / Sqrt[Dot[average, average]]];
matrix = matrix - N[Outer[Times, Dot[matrix, average], average]];
matrix = Transpose[matrix];

(* Create Sorted Human Data 2 D Red & Green Raster Display *)

contrast = 15 * 1.5;
displaying = Table[
   If[contrast * matrix[[i, j]] > 0,
    If[contrast * matrix[[i, j]] < 1, {contrast * matrix[[i, j]], 0}, {1, 0}],
    If[contrast * matrix[[i, j]] > -1, {0, -contrast * matrix[[i, j]]}, {0, 1}]],
   {i, 1, genes}, {j, 1, arrays}];
T3 = Table[
   RGBColor[displaying[[i, j, 1]], displaying[[i, j, 2]], 0],
   {i, genes, 1, -1}, {j, 1, arrays}];

(* Center Sorted Human Arraylets *)

arraylets = Transpose[arraylets3];
average = Table[1, {a, 1, genes}];
average = N[average / Sqrt[Dot[average, average]]];
arraylets = arraylets - N[Outer[Times, Dot[arraylets, average], average]];
arraylets = Transpose[arraylets];

(* Create Sorted Human Arraylets 2 D Red & Green Raster Display *)

contrast = 125 * 1.5;
displaying = Table[
   If[contrast * arraylets[[i, j]] > 0,
    If[contrast * arraylets[[i, j]] < 1, {contrast * arraylets[[i, j]], 0}, {1, 0}],
    If[contrast * arraylets[[i, j]] > -1, {0, -contrast * arraylets[[i, j]]}, {0, 1}]],
   {i, 1, genes}, {j, 1, arrays}];
U3 = Table[
   RGBColor[displaying[[i, j, 1]], displaying[[i, j, 2]], 0],
   {i, genes, 1, -1}, {j, 1, arrays}];

(* Create Human Expression Fractions Red & Green Raster Display *)

contrast = 0.03;
displaying = Table[
   If[contrast * d3[[i, j]] > 0,
    If[contrast * d3[[i, j]] < 1, {contrast * d3[[i, j]], 0}, {1, 0}],
    If[contrast * d3[[i, j]] > -1, {0, -contrast * d3[[i, j]]}, {0, 1}]],
   {i, 1, arrays}, {j, 1, arrays}];
ue3 = Table[
   RGBColor[displaying[[i, j, 1]], displaying[[i, j, 2]], 0],
   {i, arrays, 1, -1}, {j, 1, arrays}];
```

```
(* Display HO GSVD for N=3 Organisms *)
```

```
(* Create Data Tensor Display *)

gtensor = {
  Graphics[RasterArray[T1, {{0, -25}, {13, 25}}]],
  Graphics[RasterArray[T2, {{13, -42}, {26, 38}}]],
  Graphics[RasterArray[T3, {{26, -77}, {39, 51}}]],
  Graphics[Line[{{0, 25}, {0, -25}, {13, -25}, {13, 25}}]],
  Graphics[Line[{{13, 38}, {13, -42}, {26, -42}, {26, 38}}]],
  Graphics[Line[{{26, 51}, {26, -77}, {39, -77}, {39, 51}}]],
  Graphics[{RGBColor[1, 1, 1], Line[{{13, -25}, {13, 25}}]}],
  Graphics[{RGBColor[1, 1, 1], Line[{{26, -42}, {26, 38}}]}],
  Graphics[Line[{{0, 25}, {26, 51}, {39, 51}, {13, 25}, {0, 25}}]],
  Table[Graphics[Line[{{a, 25 + a}, {a - 4, 25 + a + 2}}]], {a, 0, 26, 13}],
  Graphics[Text[StyleForm["D₁", FontSize → 12], {-4, 25 + 2}, {1, 0}]],
  Graphics[Text[StyleForm["D₂", FontSize → 12], {13 - 4, 38 + 2}, {1, 0}]],
  Graphics[Text[StyleForm["D₃", FontSize → 12], {26 - 4, 51 + 2}, {1, 0}]],
  Graphics[Text["Organisms", {2, 42.5}, {0, 0}, {1, 1}]],
  Graphics[Text["Arrays", {32.5, 55}]],
  Graphics[Text["Genes", {-4, 18.5}, {0, 0}, {0, 1}]]};

(* Create Column Basis Vectors Display *)

space = 58.5;
gzarraylets = {
  Graphics[RasterArray[U1, {{space, -25}, {space + 13, 25}}]],
  Graphics[RasterArray[U2, {{space + 13, -42}, {space + 26, 38}}]],
  Graphics[RasterArray[U3, {{space + 26, -77}, {space + 39, 51}}]],
  Graphics[Line[{{space + 26, 51}, {space + 26, -77}, {space + 39, -77},
     {space + 39, 51}, {space + 26, 51}}]],
  Graphics[Line[{{space + 13, 38}, {space + 13, -42}, {space + 26, -42},
     {space + 26, 38}, {space + 13, 38}}]],
  Graphics[Line[{{space, 25}, {space, -25}, {space + 13, -25},
     {space + 13, 25}, {space, 25}}]],
  Graphics[{RGBColor[1, 1, 1], Line[{{space + 13, -25}, {space + 13, 25}}]}],
  Graphics[{RGBColor[1, 1, 1], Line[{{space + 26, -42}, {space + 26, 38}}]}],
  Graphics[Line[{{space, 25}, {space + 26, 51}, {space + 39, 51}, {space + 13, 25}, {space, 25}}]],
  Table[Graphics[Line[{{space + a, 25 + a}, {space + a - 4, 25 + a + 2}}]], {a, 0, 26, 13}],
  Graphics[Text[StyleForm["U₁", FontSize → 12], {space - 4, 25 + 2}, {1, 0}]],
  Graphics[Text[StyleForm["U₂", FontSize → 12], {space + 13 - 4, 38 + 2}, {1, 0}]],
  Graphics[Text[StyleForm["U₃", FontSize → 12], {space + 26 - 4, 51 + 2}, {1, 0}]],
  Graphics[Text["Organisms", {space + 2, 42.5}, {0, 0}, {1, 1}]],
  Graphics[Text["Arraylets", {space + 32.5, 55}]],
  Graphics[Text["Genes", {space - 4, 18.5}, {0, 0}, {0, 1}]]};
```

```
(* Create Diagonal Matrices Display *)

space = 113.75;
gcore = {
  Graphics[RasterArray[ue1, {{space, 12}, {space + 13, 25}}]],
  Graphics[RasterArray[ue2, {{space + 13, 25}, {space + 26, 38}}]],
  Graphics[RasterArray[ue3, {{space + 26, 38}, {space + 39, 51}}]],
  Graphics[Line[
    {{space, 25}, {space, 12}, {space + 13, 12}, {space + 13, 25}, {space, 25}}]],
  Graphics[Line[
    {{space + 13, 38}, {space + 13, 25}, {space + 26, 25}, {space + 26, 38}, {space + 13, 38}}]],
  Graphics[Line[
    {{space + 26, 51}, {space + 26, 38}, {space + 39, 38}, {space + 39, 51}, {space + 26, 51}}]],
  Graphics[Line[{{space, 25}, {space + 26, 51}}]],
  Graphics[Line[{{space + 13, 12}, {space + 39, 38}}]],
  Table[Graphics[Line[{{space + a, 25 + a}, {space + a - 4, 25 + a + 2}}]], {a, 0, 26, 13}],
  Graphics[Text[StyleForm["Σ1", FontSize → 12], {space - 4, 25 + 2}, {1, 0}]],
  Graphics[Text[StyleForm["Σ2", FontSize → 12], {space + 13 - 4, 38 + 2}, {1, 0}]],
  Graphics[Text[StyleForm["Σ3", FontSize → 12], {space + 26 - 4, 51 + 2}, {1, 0}]],
  Graphics[Text["Organisms", {space + 2, 42.5}, {0, 0}, {1, 1}]],
  Graphics[Text["Genelets", {space + 32.5, 55}]],
  Graphics[Text["Arraylets", {space - 4, 18.5}, {0, 0}, {0, 1}]]};

(* Create Row Basis Vectors Display *)

space = 156;
gxgenelets = {
   Graphics[RasterArray[G, {{space + 13, 38}, {space + 26, 51}}]],
   Graphics[Line[{{space + 13, 51}, {space + 13, 38}, {space + 26, 38}, {space + 26, 51},
      {space + 13, 51}}]],
   Graphics[Line[{{space + 13, 51}, {space + 13 - 4, 51 + 2}}]],
   Graphics[Text[StyleForm["V^T", FontSize → 12], {space + 13 - 4, 51 + 2}, {1, 0}]],
   Graphics[Text["Arrays", {space + 19.5, 55}]],
   Graphics[Text["Genelets", {space + 13 - 4, 44.5}, {0, 0}, {0, 1}]]};
```

```
(* Display HO GSVD for N=3 Organisms *)

equal = Graphics[Text[StyleForm["=", FontSize → 30], {45.5, 18.5}]];
g = Show[{gtensor, equal, gzarraylets, gcore, gxgenelets},
   PlotRange -> All,
   AspectRatio -> Automatic];
```

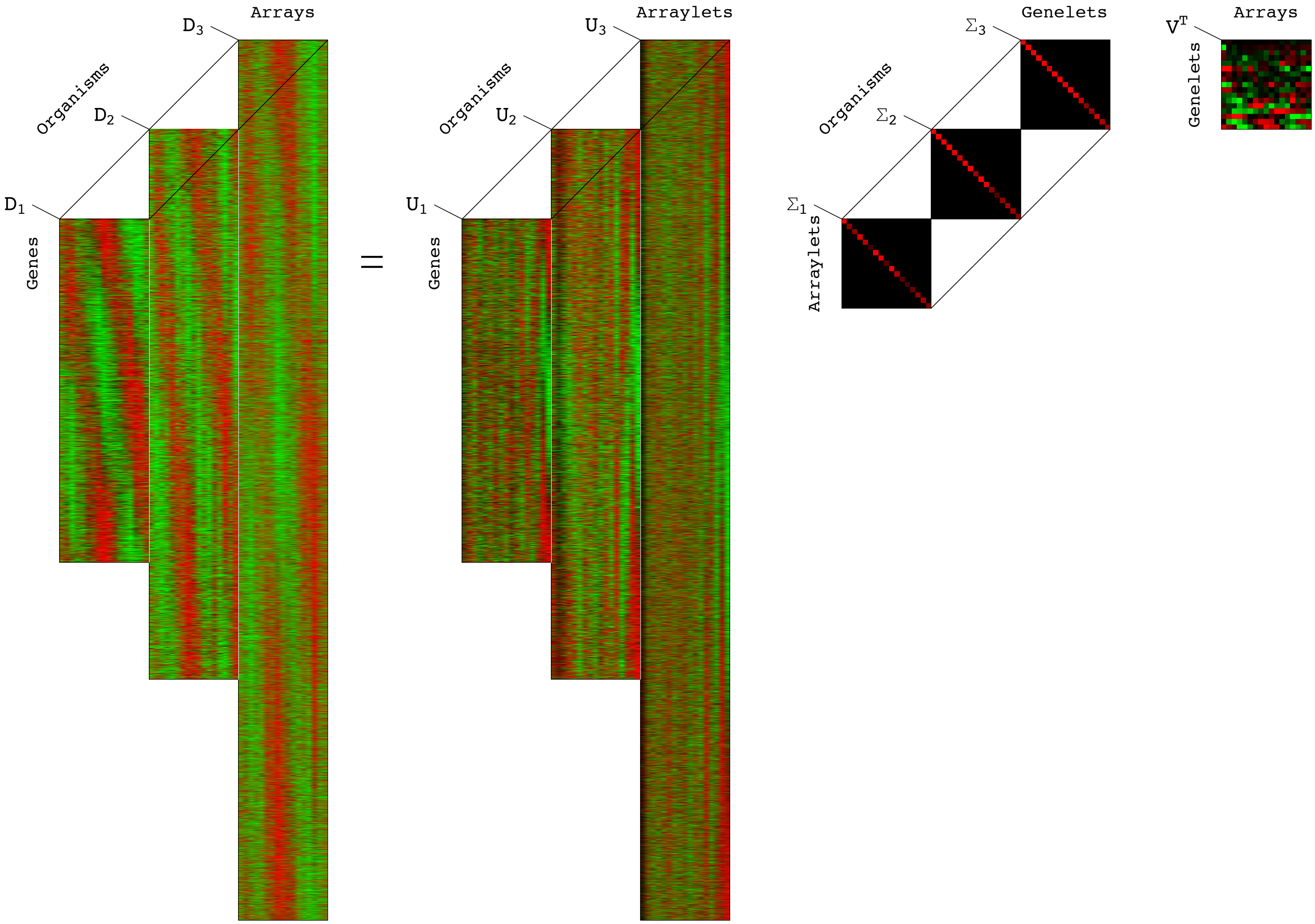

Supplement: Mathematica Notebook S2 — HO GSVD of global mRNA expression datasets from three different organisms. A PDF format file, readable by Adobe Acrobat Reader. (PDF) [file pone.0028072.s003.pdf]
